# Capturing Carbohydrate Conformations and Hydration Interactions with a Polarizable Bond Dipole Potential

**DOI:** 10.3390/molecules31030533

**Published:** 2026-02-03

**Authors:** Meng-Yao Bai, Xiao-Han Zheng, Shan-Shan Gao, Xiao-Nan Jiang, Jia-Yi Zhu, Qiang Hao, Li-Dong Gong, Lei Wang, Chang-Sheng Wang

**Affiliations:** School of Chemistry and Chemical Engineering, Liaoning Normal University, Dalian 116029, China; mengyao.bai@foxmail.com (M.-Y.B.); zhengxiaohan_cc@outlook.com (X.-H.Z.); lyric_gao@163.com (S.-S.G.); jiangxn@lnnu.edu.cn (X.-N.J.); zhujiayi940129@outlook.com (J.-Y.Z.); wanglei67@lnnu.edu.cn (L.W.)

**Keywords:** carbohydrates, polarizable potential, bond dipole model, conformational energetics, hydration interactions

## Abstract

The accurate modeling of carbohydrates is challenged by conformational flexibility, hydration, and many-body electrostatics. In this work, a polarizable bond dipole potential for carbohydrates (PBDPC25) is presented, in which C–O, O–H, and C–H bonds are represented as intrinsically polarizable dipoles. Electrostatic interactions are described through bond dipole coupling, with an orbital overlap contribution introduced to account for hydrogen bonding. For carbohydrate monomers, PBDPC25 reproduces conformational energies with a root-mean-square error (RMSE) of 2.13 kcal/mol. This accuracy exceeds that of GLYCAM06 (2.87 kcal/mol) and CHARMM36 (3.74 kcal/mol). It is also slightly better than the polarizable AMOEBA force field (2.82 kcal/mol). Optimized geometries are maintained within 0.15 Å of benchmark reference structures. This level of agreement is comparable to GLYCAM06 (0.21 Å) and close to CHARMM36 and AMOEBA (both 0.14 Å). Molecular dipole moments show excellent agreement with the reference data. Correlation coefficients exceed *R*^2^ > 0.98. For carbohydrate–water clusters, hydration energies, including many-body contributions, are predicted with an RMSE of 3.50 kcal/mol. This represents a substantial improvement over GLYCAM06, CHARMM36, and AMOEBA. These results demonstrate that PBDPC25 provides a reliable framework for modeling carbohydrate conformations and local hydration effects.

## 1. Introduction

Carbohydrates are among the most abundant and versatile biomolecules in nature. In biological systems, they serve diverse functions ranging from energy storage [1] and structural support to recognition [2] and signaling processes [3]. Their presence in glycoproteins and glycolipids [4,5] also makes them key mediators in processes such as cell adhesion, immune response, and pathogen recognition [6,7,8]. Beyond biology, carbohydrates have significant relevance in chemical and material sciences, for instance, as renewable biofuels [9,10] and as stabilizing agents in extreme environments [11,12]. Owing to this broad functional diversity, the ability to accurately describe carbohydrate conformations and their interactions with aqueous environments is of considerable importance for both fundamental understanding and practical applications.

Despite decades of investigation, modeling carbohydrates remains highly challenging. Their conformational landscapes are shaped by multiple competing factors, including the intrinsic flexibility of pyranose and furanose rings [13,14], rotations of hydroxymethyl groups, and the presence of multiple hydroxyl substituents capable of forming dynamic hydrogen-bond networks [15,16]. Solvation further amplifies this complexity, as water not only stabilizes certain conformers but also mediates cooperative hydrogen-bonding motifs [17,18]. In addition, electrostatic polarization significantly alters both intramolecular and intermolecular interactions, leading to substantial variations in dipole moments and energetics depending on the environment [19,20]. These factors together make carbohydrates prototypical systems, where the accurate treatment of conformational energetics, structural properties, and hydration interactions is essential but nontrivial.

A wide range of computational methods has been employed to address these challenges. High-level quantum chemical approaches, such as MP2 or CCSD(T), provide accurate reference data for small systems and have been widely used to benchmark carbohydrate conformations and hydration energies [21,22,23]. Density functional theory (DFT), particularly hybrid and dispersion-corrected functionals, has made carbohydrate calculations more tractable while retaining reasonable accuracy [24,25,26]. Nevertheless, both wavefunction-based and DFT methods remain limited in system size and time scale, preventing their routine application to realistic aqueous carbohydrate systems. Semi-empirical methods alleviate some computational cost but often fall short in accuracy, especially for reproducing subtle conformational preferences [27,28].

Classical force fields have therefore become indispensable for carbohydrate simulations, offering the efficiency required for long timescale molecular dynamics. Additive force fields such as CHARMM [29,30,31], GLYCAM [32,33], GROMOS [34,35,36], OPLS-AA [37,38], ESFF [39], and MM3 [40] have been parameterized for carbohydrates and successfully applied to reproduce densities, viscosities, adsorption energies, and structural properties in solution [41,42]. Their efficiency and transferability are clear strengths. However, by construction, they employ fixed atomic charges, which cannot adapt to environmental changes in electron distribution. This omission of explicit polarization can limit their accuracy in describing dielectric properties, conformational equilibria involving polar groups, and hydration energetics [33,43]. To address this limitation, polarizable force fields such as AMOEBA [44,45,46], Drude oscillator-based [47] and ABEEM [48] models have been developed. These frameworks incorporate electronic polarization explicitly and have demonstrated improved agreement with both quantum data and experiments for carbohydrate monomers and solvated systems [49,50]. Nevertheless, they require more complex parameterization and often incur higher computational costs, which may restrict their widespread use in large-scale simulations.

Recently, an alternative approach has been proposed in which chemical bonds are represented directly as dipoles. In this bond dipole framework, polar covalent bonds are treated as dipoles that can undergo polarization in response to their environment. Electrostatic energies are then described by interactions among these permanent and induced dipoles, naturally accounting for polarization and many-body effects. Compared with atomic charge or multipole-based polarizable models, this bond dipole representation offers a more compact description of electrostatics and potentially reduces computational overhead, while retaining the essential physics of polarization. Previous studies have shown that this framework can reproduce interaction energies and dipole moments of small molecules [51], amino acids [52,53], and simple aqueous systems [54], with an accuracy comparable to that of dispersion-corrected DFT methods.

In the present study, the polarizable bond dipole potential is extended to carbohydrates. Parameters for C–O, O–H, and C–H bonds within oligosaccharides are developed, and the capability of the model to reproduce key physical properties is assessed. Benchmark evaluations are carried out for the conformational energies of carbohydrate monomers, equilibrium geometries, molecular dipole moments, and hydration interaction energies of carbohydrate–water clusters. The results are compared with high-level quantum mechanical reference data and widely used carbohydrate force fields. Through this assessment, the extent to which the bond dipole framework captures the essential energetics and electrostatics of carbohydrates is examined.

## 2. Methods

The polarizable bond dipole potential for carbohydrates (PBDPC25) is formulated as a sum of bonded and non-bonded contributions and is written as

(1) $$E_{\mathrm{total}}=E_{\mathrm{bonded}}+E_{\mathrm{non-bonded}}$$

where *E*_bonded_ represents the bonded interaction terms, and *E*_non-bonded_ accounts for the non-bonded interaction terms.

### 2.1. Bonded Interactions

The bonded terms include bond stretching, angle bending, and dihedral torsions, which collectively define the intramolecular conformational landscape of carbohydrates. In this work, special attention is devoted to accurately capturing conformational energetics through a Fourier-based torsional expansion, supplemented by harmonic bond and angle potentials. These bonded contributions are parametrized against quantum chemical reference data for monosaccharides and serve as the central extension of the present model beyond our earlier study [55]. The bonded term *E*_bonded_ is written as

(2) $$\begin{array}{ll} E_{\mathrm{bonded}} & =E_{\mathrm{bond}}+E_{\mathrm{angle}}+E_{\mathrm{dihedral}}\\ & =\sum_{b}K_{b}{\left(b-b_{0}\right)}^{2}+\sum_{\theta }K_{\theta }{\left(\theta -\theta_{0}\right)}^{2}\\ & +\sum_{\varphi }V_{n}[1+\cos(n\phi_{n}-\gamma_{n})] \end{array}$$

where *b* and *b*_0_ are the instantaneous and equilibrium bond lengths, respectively. *K*_b_ is the associated force constant. *θ* and *θ*_0_ denote the actual and equilibrium bond angles, and *K*_θ_ is the angle force constant. *φ_n_* is the dihedral angle, and *V*_n_ is the Fourier coefficient. *γ*_n_ is the phase angle, and *n* is the periodicity.

### 2.2. Non-Bonded Interactions

The non-bonded term *E*_non-bonded_ can be further expressed as Equation (3), in which *E*_es_ is the electrostatics among permanent and induced bond dipoles, *E*_vdW_ is van der Waals dispersion–repulsion, and *E*_orb_ is orbital overlap contributions relevant to hydrogen bonding.

(3) $$\begin{array}{ll} E_{\mathrm{non-bonded}} & =E_{\mathrm{es}}+E_{\mathrm{vdW}}+E_{\mathrm{orb}}\\ & =\sum_{\mathrm{es}}\frac{\mu_{i0}\mu_{j0}+\mu_{i0}\delta \mu_{j}+\mu_{j0}\delta \mu_{i}+\delta \mu_{i}\delta \mu_{j}}{r_{ij}^{3}}\cdot (2\cos\alpha \cos\alpha^{\prime }+\sin\alpha \sin\alpha^{\prime }\cos\beta )\\ & +\sum_{\mathrm{vdW}}(\frac{A_{ij}}{R_{ij}^{12}}-\frac{B_{ij}}{R_{ij}^{6}})\\ & +\sum_{\mathrm{orb}}D[e^{-2a(R_{\mathrm{HB},m}-R_{0})}-2e^{-a(R_{HB,m}-R_{0})}{]}^{5}\cdot \cos(\alpha_{m}-\alpha_{0})\cos(\beta_{m1}-\beta_{0})\cos(\beta_{m2}-\beta_{0}) \end{array}$$

The electrostatics *E*_es_, van der Waals *E*_vdW_, and orbital overlap contributions *E*_orb_ have been established in our previous works [55,56], and together, they account for polarization, many-body electrostatics, and the directional nature of hydrogen bonding.

In this bond-dipole-based model, electrostatic interactions [the 1st term of Equation (3)] are evaluated between localized dipoles assigned to chemically significant polar bonds (e.g., C–O, O–H, and C–H), while C–C bonds are omitted to improve computational efficiency. Total electrostatic energy consists of three contributions: interactions between (i) permanent–permanent bond dipoles, (ii) permanent–induced bond dipoles, and (iii) induced–induced bond dipoles. Here, *μ* denotes the magnitude of the bond dipole moment, and *δμ* represents the magnitude of the corresponding induced dipole moment. Unless otherwise specified, these quantities are treated as scalar magnitudes rather than full vector quantities. The induced bond dipole moment *δμ* in the 1st term of Equation (3) is evaluated as

*δμ* = *c*(*q* − *q*_0_)*d* (4)

where *d* is the bond length, *q*_0_ denotes a fixed, atom-type-dependent reference charge, and *q* is the geometry-dependent atomic partial charge associated with the bonded atoms. The induced bond dipole *δμ* therefore reflects deviations of the instantaneous charge distribution from this reference state.

In the present implementation, *q* is evaluated for each molecular geometry using AM1 semiempirical calculations, providing a geometry-dependent polarization response. In contrast, *q*_0_ is a fixed reference quantity that characterizes the average electronic environment of a given atom type and is treated as an adjustable model parameter. This procedure yields an explicit geometry-dependent polarization response while avoiding the introduction of an additional self-consistent polarization cycle at the molecular–mechanical level. Although AM1 is employed here for charge evaluation, the bond-dipole framework itself is not tied to a specific electronic-structure method. Alternative charge–evaluation schemes or non-iterative charge–response models can be readily incorporated in future implementations without modifying the underlying formalism.

Because atomic charges derived from AM1 may exhibit systematic deviations, a correction factor *c* is introduced to compensate for such effects within the present parameterization. Accordingly, the permanent bond dipole *μ*_0_, the reference atomic charge *q*_0_, and the correction factor *c* are treated as adjustable parameters and optimized simultaneously to ensure a consistent description of polarization effects.

Orbital overlap interactions serve as a correction to electrostatics for describing the directionality and cooperativity of hydrogen bonding. This term is expressed as a function of the overlap between donor and acceptor electronic clouds. Specifically, *D* represents a scaling constant related to the electronic distribution in the sugar–water system, *a* controls the decay shape of the overlap, and *R*_0_ is the equilibrium hydrogen bond distance. For each hydrogen bond *m*, *R*_HB,*m*_ denotes the donor–acceptor distance, while the angular dependence is described by parameters (*α*_m_, *β*_m1_, *β*_m2_) relative to their ideal reference values (*α*_0_, *β*_0_). This formulation allows the model to capture both the geometric preference and the strength modulation of hydrogen bonds, beyond what is provided by dipole-based electrostatics alone.

### 2.3. Unified Intramolecular–Intermolecular Polarization Framework

The central advance of the present work lies in extending the polarizable bond dipole framework from the previous application of the framework to purely intermolecular interactions in sugar–water systems to the inclusion of intramolecular polarization within carbohydrates. In this extension, each C–O, O–H, and notably C–H bond is explicitly represented as a polarizable dipole. By incorporating both permanent and induced contributions from C-H bonds, a refined set of parameters was developed while retaining the same functional forms of the electrostatic interactions established in our earlier work [55]. This augmented representation allows the model to capture not only hydration-induced polarization but also the subtle intramolecular electrostatic effects that govern local conformational preferences, such as hydroxymethyl rotations and ring puckering. Crucially, the same polarization formalism applies seamlessly across intra- and inter-molecular levels, avoiding double-counting and preserving internal consistency.

As a result, the framework now provides a unified treatment of polarization and many-body effects, enabling carbohydrates in aqueous environments to be modeled with improved fidelity, where conformational flexibility and hydration are inherently coupled.

## 3. Parameterization Strategy

The PBDPC25 potential was parameterized by combining parameter transfer with targeted refinement against high-level quantum-chemical reference data. Carbohydrate atoms were assigned to chemically meaningful types (CT, OS, OH, HO, H1, and H2; Table S1). Bonded terms, including bond stretching, angle bending, and torsional potentials, were initialized from the GLYCAM06 force field [32], which provides a well-established baseline for carbohydrate conformational energetics. Non-bonded terms, including electrostatic interactions among permanent and induced bond dipoles, van der Waals dispersion–repulsion, and an orbital-overlap contribution, were transferred from earlier developments of the bond-dipole framework and used as initial values.

To improve the description of electrostatics within carbohydrates, the representation was extended to include explicit C-H bond dipoles, in addition to the C–O and O–H dipoles used previously. Although C–H bonds are only weakly polar, their cumulative contribution to the molecular dipole distribution and intramolecular polarization can become appreciable. Their explicit inclusion was therefore adopted to provide a more complete and internally consistent description of the electrostatic response

**Figure 1:**
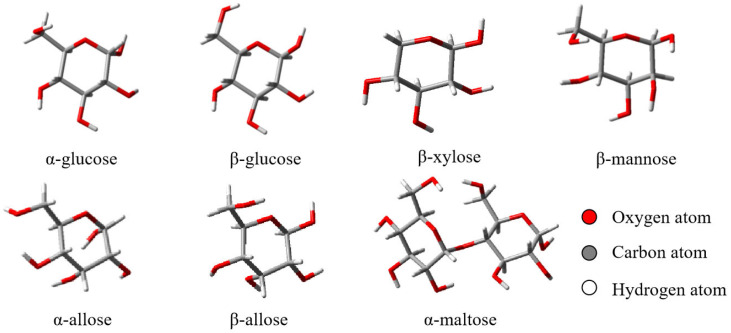
Training set I. Schematic representations of the lowest-energy conformers of α-glucose, β-glucose, α-maltose, β-xylose, β-mannose, α-allose, and β-allose employed in the parameterization of electrostatic interactions.

As shown in Figure 1, the lowest-energy conformers of α-glucose [57], β-glucose [57], α-maltose [57], β-xylose [22], β-mannose [22], α-allose [21], and β-allose [21] were selected as the training set to determine the electrostatic parameters, including the permanent bond dipole *μ*_0_, the reference atomic charge *q*_0_, and the correction factor *c* for the CT-OS, CT-OH, OH-HO, CT-H1, and CT-H2 bonds. The benchmark data of the dipole components in the XYZ directions and the total molecular dipole moment for these lowest-energy conformers were obtained at the B3LYP/aug-cc-pVTZ level, which has been shown to reproduce experimental dipoles with small mean absolute deviations [58].

The reference charge *q*_0_ for atom HO in the OH-HO bond is 0.2142 e, which is derived from the average of the AM1 partial charges on all the HO atoms of the seven lowest-energy conformers, as shown in Figure 1. The reference charge *q*_0_ for H1 or H2 is derived from the average of the partial charges on all the H1 or H2 atoms of the seven lowest-energy conformers. The induced bond dipoles of the chemical bonds inside sugar, such as *μ*_0_ (CT-OS) and *μ*_0_ (CT-OH), are not taken into account in our simulation.

The electrostatic parameters *μ*_0_ and *c* of PBDPC25 were determined by least-squares fitting of the modeled Cartesian dipole components to the reference values. In this fitting procedure, the permanent bond dipoles (*μ*_0_) were adjusted manually to minimize vectorial discrepancies across the training set, while the correction factor *c* was calibrated to account for induced contributions within the bond dipole formalism.

Table 1 reports the component-wise and magnitude comparisons for the molecular dipole moments of these representative carbohydrates. Across training set I, the optimized electrostatic parameters reproduce the reference B3LYP/aug-cc-pVTZ molecular dipole magnitudes with a root-mean-square error (RMSE) of 0.23 D. The directional RMSEs for the *x*, *y,* and *z* components are 0.22 D, 0.17 D, and 0.29 D, respectively. The component-wise errors are distributed without a clear systematic bias in sign, indicating that no global vectorial offset was introduced by the fitting procedure. The largest single deviation in *μ*_M_ (0.50 D) is observed for α-maltose and is accompanied by compensating changes in the Cartesian components.

The reported RMSE values indicate that PBDPC25 reproduces the orientation and magnitude of molecular dipoles to within sub-Debye accuracy for the chosen carbohydrate monomers. Such an agreement is consistent with the intended role of the dipole fitting: to ensure that the permanent electrostatic description captures the bulk of the molecular dipole while leaving induced contributions to be handled self-consistently by the polarizable bond dipole machinery.

The orbital interactions for hydrogen bonding between water–water molecules (OW-HW∙∙∙OW) and between water–carbohydrate molecules (OH-HO∙∙∙OW, OW-HW∙∙∙OH, and OW-HW∙∙∙OS) are considered in carbohydrate–water clusters. Parameters are taken directly from our previous work [56] without any modification. The intramolecular OH-HO∙∙∙OH or OH-HO∙∙∙OS interactions are not taken into account for the sake of efficiency and precision. The parameters for bond stretching and angle bending are taken from the GLYCAM06 force field [32] directly.

**Figure 2:**
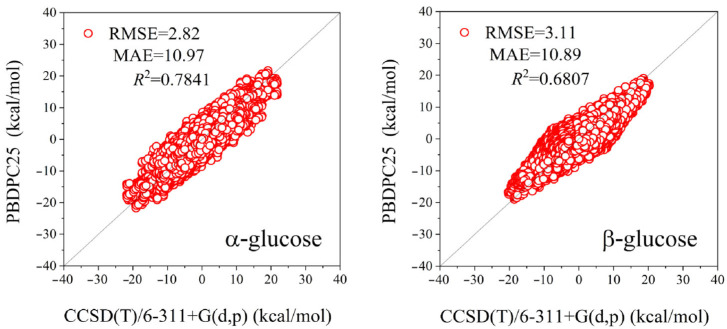
Correlation of conformational energies for 204 α-glucose and 255 β-glucose structures in training set II, used for the optimization of van der Waals and bonded parameters.

A total of 204 and 255 conformational structures of α- and β-glucose, respectively, with reference energies calculated at the CCSD(T)/6-311+G(d,p) level [22], were chosen as the training set. Van der Waals parameters for OS, HO, and H2 atoms, as well as dihedral parameters for selected representative torsions (e.g., CT–CT–CT–OS, OS–CT–CT–OS, and OH–CT–CT–OS), were subsequently refined by targeted manual fitting to the CCSD(T)/6-31++G(d,p) reference data. The fitting procedure focused on reproducing intramolecular conformational energy profiles, with all data points treated with equal weight. To maintain overall consistency with established carbohydrate force-field parameterizations, parameter adjustments were kept deliberately modest, with the final values typically differing by less than ±10% from the corresponding GLYCAM06 parameters. One exception is the H2–CT–OS–CT dihedral, for which the associated *V_n_* terms were set to 0.0, reflecting its negligible contribution to the conformational energetics considered here. Figure 2 demonstrates that the refitted potential reproduces the benchmark conformational energy profiles with good overall fidelity, yielding RMSEs of 2.82 kcal/mol for α-glucose and 3.11 kcal/mol for β-glucose, with correlation coefficients of *R*^2^ = 0.78 and *R*^2^ = 0.68, respectively.

In the treatment of intramolecular non-bonded interactions, electrostatic contributions are evaluated between pairs of bond dipoles separated by three or more covalent bonds, without the application of additional scaling factors. Intramolecular van der Waals interactions are included, starting from 1–4 atom pairs, with a scaling factor of 0.5 applied to 1–4 interactions and full (unit) weighting applied to 1–5 and longer-range interactions.

The final parameter sets for carbohydrates and water are reported in Table S2. A direct comparison with an earlier version of PBDPC25 using a test set of 78 carbohydrate–water clusters (Table S3) shows that the refined parameters lead to a reduced root-mean-square deviation for interaction energies, while the RMSE of molecular dipole moments increases slightly from 0.47 to 0.51 D. This trade-off reflects a modest rebalancing between the interaction energetics and dipole properties in the current parameter refinement.

All quantum chemical calculations were performed using Gaussian [59] and ORCA [60]. Simulations based on the polarizable bond dipole potential were carried out with our in-house PBFF code [61], and other force field calculations were performed using the TINKER 8 package [62].

## 4. Application

The potential was applied to carbohydrate monomers to predict conformational energies, equilibrium geometries, and molecular dipole moments. Subsequently, the potential was extended to carbohydrate–water clusters to evaluate the total interaction energies (*IE*_tot_) and many-body interaction contributions (*IE*_mb_). Detailed definitions, together with the computational expressions of these interaction energies, are presented in Section 2 of the Supporting Information. The accuracy of the potential was evaluated using root-mean-square error (RMSE), maximum absolute error (MAE), mean relative error (MRE), and the coefficient of determination (*R*^2^), as defined in Section 3 of the Supporting Information. Subsequent analyses focus on intramolecular conformations, dipole moments, and carbohydrate–water interaction energies. Results are compared with three established carbohydrate force fields—GLYCAM06 [32], CHARMM36 [30], and AMOEBA [46]—to assess the ability of the polarizable bond dipole potential to reproduce conformational preferences, electrostatic properties, and many-body effects in both monomeric and polymolecular systems.

### 4.1. Carbohydrates Monomers

#### 4.1.1. Conformational Energy Predictions

The performance of the polarizable bond dipole potential (PBDPC25) in describing intramolecular conformational energetics was systematically evaluated using benchmark datasets of carbohydrate conformers. Reference geometries and relative energies were taken from high-level ab initio calculations reported previously [21,22,57]. In total, 652 conformers spanning seven representative carbohydrate systems were included: 80 α-glucose, 76 β-glucose, 223 α-maltose, 90 β-xylose, 168 β-mannose, 9 α-allose, and 6 β-allose. These datasets encompass a broad range of hydroxymethyl rotamers, ring-puckering states, and intramolecular hydrogen-bonding patterns, providing a stringent test of conformational energy surfaces.

The accuracy of conformational energy predictions was quantified by comparison with vacuum quantum mechanical reference energies using three statistical measures. RMSE (0) evaluates deviations in relative energies referenced to the global minimum conformer and reflects the accuracy of energetic ordering among low-lying states. RMSE (each) and MAE (each) characterize deviations across the full conformational ensemble and are sensitive to the overall uniformity of the potential energy surface and the presence of large deviations. The formal definitions of these metrics are provided in the Supporting Information (Section 3).

As summarized in Table 2 and supported by the conformational energy correlations in Figure S1 and Tables S4–S10, PBDPC25 achieves an average RMSE (0) of 2.13 kcal/mol across all systems, outperforming GLYCAM06 (2.87 kcal/mol), CHARMM36 (3.74 kcal/mol), and AMOEBA (2.82 kcal/mol). This improvement is most pronounced for α-glucose, β-glucose, and α-maltose, where conformational energetics are strongly modulated by coupled hydroxymethyl rotations and ring deformations. For β-xylose and β-mannose, which exhibit multiple accessible ring conformations, PBDPC25 again yields lower RMSE (0) values than GLYCAM06 and CHARMM36 and comparable accuracy to AMOEBA.

Global accuracy metrics further support these trends. Averaged over all systems, PBDPC25 produces an RMSE (each) of 2.77 kcal/mol and an MAE (each) of 8.28 kcal/mol, representing substantial reductions relative to CHARMM36 (3.20/9.74 kcal/mol) and GLYCAM06 (3.49/12.31 kcal/mol). Compared with AMOEBA (3.39/10.00 kcal/mol), PBDPC25 also shows competitive or improved performance, particularly for glucose and maltose derivatives. The reduction in MAE (each) indicates that large absolute deviations are less frequent, suggesting a more uniform reproduction of energy differences across the conformational landscape rather than accuracy limited to low-energy regions.

The most significant absolute improvements are observed for α-maltose. For this disaccharide, RMSE (each) is reduced from 4.99 (CHARMM36), 5.18 (GLYCAM06), and 5.33 kcal/mol (AMOEBA) to 3.46 kcal/mol with PBDPC25, while MAE (each) decreases from 18.57, 25.19, and 20.44 kcal/mol to 12.73 kcal/mol. These reductions reflect a marked suppression of large outliers in conformer energies and imply improved reliability of Boltzmann-weighted conformational populations derived from the PBDPC25 energy surface. Comparable, though smaller, improvements are also observed for α- and β-glucose, as well as for β-xylose and β-mannose.

For structurally simpler systems such as α- and β-allose, all force fields examined achieve relatively small errors, with RMSE (each) values near or below 2 kcal/mol. In these cases, PBDPC25 performs comparably to the established additive force fields but does not exhibit a systematic advantage. For α-allose, both GLYCAM06 and CHARMM36 yield lower errors across all three metrics, whereas PBDPC25 remains within ~1–2 kcal/mol of the best-performing model. For β-allose, PBDPC25 yields RMSE (0) and RMSE (each) values lower than CHARMM36 and AMOEBA and comparable to GLYCAM06. As shown in Table S10, the CCSD(T)/CBS reference conformational energies span a limited range of ~0–4.2 kcal/mol across six conformers, with only two lying more than 2 kcal/mol above the global minimum. This compressed energetic spread limits the extent of achievable error reduction, leading to modest differences among force fields. Within this regime, PBDPC25 reproduces the overall ordering and energetic spacing of β-allose conformers, with an accuracy comparable to the best-performing additive model, although conformers separated by very small energy differences remain difficult to distinguish unambiguously.

The benchmark energies reported in Table 2 correspond to the vacuum CCSD(T) level [21,22,57] reference data. The performance of the comparison force fields should therefore be interpreted in light of their intended parameterization environments. GLYCAM06 and CHARMM36 are fixed-charge biomolecular force fields primarily designed for simulations in aqueous or condensed-phase environments. In such models, partial charges are effectively enhanced to reproduce hydration structures and condensed-phase observables in the absence of explicit electronic polarizability. When applied to gas-phase conformational energetics, particularly for hydroxyl-rich systems such as maltose, this design choice can lead to overly favorable intramolecular electrostatic interactions, accounting for the larger deviations observed in Table 2.

In contrast, the PBDPC25 model employs geometry-responsive bond dipoles that adapt to changes in molecular conformation and local electronic structure, enabling a balanced description of intramolecular electrostatics in a vacuum without reliance on environment-specific overpolarization. These considerations rationalize the systematic trends observed in Table 2 and are consistent with prior discussions in the literature [63,64] on the environment dependence of fixed-charge and polarizable force-field models.

Overall, the results demonstrate that PBDPC25 offers a balanced and reliable description of carbohydrate conformational energetics in the vacuum. Its capability to accurately capture both the relative stability of low-energy conformers and the energy differences in higher-energy regions underscores the advantages of utilizing bond-dipole-based electrostatics and orbital corrections for hydrogen bonding.

#### 4.1.2. Structural Predictions

The structural performance of PBDPC25 was evaluated by comparing optimized geometries with reference structures, using CHARMM36, GLYCAM06, and AMOEBA force fields for comparison. Hydrogen atoms were excluded from the analysis, and the root-mean-square errors (RMSEs) were calculated for all heavy atoms following superimposition.

Structural optimizations with PBDPC25 were performed using numerical gradients, with step sizes iteratively adjusted until convergence in total energy was achieved. In the present implementation, analytical gradients are not yet available, as the analytical derivatives of the induced dipole contributions are still under development and validation. For comparison, structures optimized with CHARMM36, GLYCAM06, and AMOEBA were minimized using their respective analytical-gradient implementations until the root-mean-square (RMS) gradient fell below 0.01 kcal/Å.

To assess the structural fidelity of the optimized geometries, each carbohydrate monomer was compared against reference gas-phase structures reported in prior quantum-chemical studies. These reference geometries correspond to the lowest-energy conformers identified in the original literature and were optimized at the B3LYP/6-31+G(d,p) [21], B3LYP/6-311+G(2df,p) [22], or MP2/def2-TZVPP [57] levels of theory, depending on the specific system. For consistency with the published benchmarks and to ensure an objective comparison, these structures were taken directly from the literature without further reoptimization. The reported RMSE values in Table 3, therefore, reflect deviations relative to the established reference geometries used in the corresponding studies.

The root-mean-square errors (RMSEs) of heavy-atom positions relative to these reference geometries are summarized in Table 3, providing a quantitative measure of the structural accuracy achieved by the present model across the seven stable carbohydrate monomers. PBDPC25 yields an average RMSE of 0.15 Å, which is comparable to GLYCAM06 (0.21 Å), and close to CHARMM36 (0.14 Å) and AMOEBA (0.14 Å). Individual systems demonstrate consistent accuracy: for α-glucose and β-glucose, the RMSEs are 0.14 Å and 0.09 Å for PBDPC25, comparable to or slightly larger than CHARMM36 and AMOEBA, yet smaller than GLYCAM06 for β-glucose. For α-maltose, the RMSE is 0.30 Å, showing a noticeable improvement over GLYCAM06 (0.45 Å) and CHARMM36 (0.42 Å), and close to AMOEBA (0.37 Å), indicating that PBDPC25 can reproduce disaccharide geometries with reduced deviations. Similar trends are observed for β-xylose, β-mannose, and the allose isomers, where PBDPC25 consistently provides low RMSE values, demonstrating accurate preservation of ring conformations and overall structures.

#### 4.1.3. Molecular Dipole Moments Predictions

Here, seven representative carbohydrate systems were selected as the test set, comprising 80 α-glucose conformers, 76 β-glucose conformers, 223 α-maltose conformers, 90 β-xylose conformers, 168 β-mannose conformers, 9 α-allose conformers, and 6 β-allose conformers. The B3LYP/aug-cc-pVTZ method was used as a benchmark for molecular dipole moments, while CHARMM36, GLYCAM06, and AMOEBA force fields served as comparative references.

**Figure 3:**
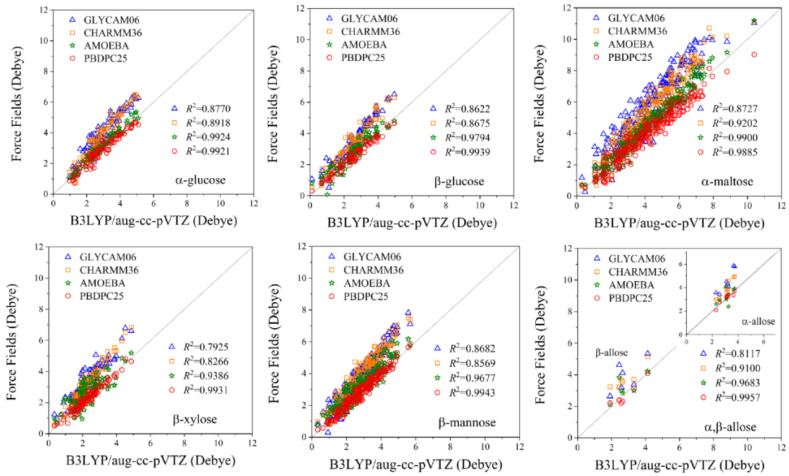
Correlation of molecular dipole moments (D) between tested force fields and the benchmark method for 652 carbohydrates.

The correlation between dipole moments predicted by the tested force fields and the benchmark method is shown in Figure 3. The results indicate that the dipole moments computed by the present potential closely follow the *y* = *x* diagonal, with a correlation coefficient of *R*^2^ > 0.98, demonstrating excellent agreement with high-level quantum chemical calculations. The accuracy of the AMOEBA force field for molecular dipole moments is close to our potential. In contrast, both CHARMM36 and GLYCAM06 yield dipole moments that are larger than the corresponding vacuum quantum mechanical reference values.

In a vacuum, both CHARMM36 and GLYCAM06 yield dipole moments that are systematically larger than the quantum mechanical reference values. This behavior is well understood and reflects the fixed-charge parameterization strategies of these force fields, which are primarily designed for simulations in aqueous or crystalline environments [30,32,33]. In such models, partial charges are effectively enhanced to reproduce condensed-phase observables in the absence of explicit electronic polarizability, which naturally leads to larger dipole moments when evaluated in the gas phase.

Overall, the results demonstrate that the present potential provides a robust and physically meaningful description of carbohydrate electrostatics, offering clear advantages over traditional additive force fields and comparable with a polarizable force field. Detailed molecular dipole moment values for all conformers are provided in Tables S11–S16 (SI).

### 4.2. Carbohydrate–Water Clusters

**Figure 4:**
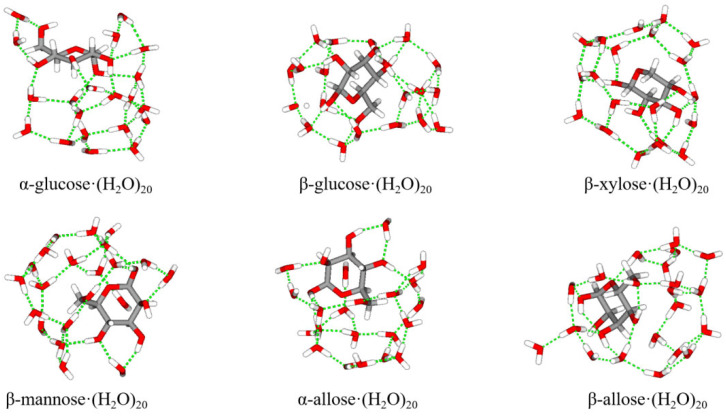
Structures of six carbohydrate–(H_2_O)_20_ clusters. (The colors red, gray and white correspond to oxygen, carbon and hydrogen respectively).

In this section, the PBDPC25 was employed to investigate the energetics of carbohydrate–water clusters (Figure 4), with particular emphasis on the total interaction energies and the many-body contributions arising from polarization effects.

For carbohydrate–water cluster calculations, PBDPC25 employs a bond-dipole-based water model consistent with the electrostatic framework of the carbohydrate potential. Specifically, the PBFF-WAT-2025 model [56] assigns permanent and geometry-responsive induced dipoles to O–H bonds within the same chemical bond dipole formalism. For the comparison force fields, standard and force-field-consistent water models were used: CHARMM36 employed the CHARMM-modified TIP3P model, and GLYCAM06 employed the TIP3P model. The DLPNO-CCSD(T)/CBS (tightPNO, tightSCF) method [65] is used as a benchmark method. Here, def2-TZVPP and def2-QZVPP basis sets in MP2 level are used for extrapolation. The detailed coordinates of six carbohydrate–(H_2_O)_20_ clusters are provided in Table S19 (SI).

#### 4.2.1. Total Interaction Energies

The total interaction energies (*IE*_tot_) of six carbohydrate–(H_2_O)_20_ clusters were evaluated using PBDPC25 and compared with the DLPNO-CCSD(T)/CBS (tightPNO) benchmark results, as summarized in Table 4. Additional comparisons were made with two density functional methods (ωB97X-D and M06-2X), two fixed-charge force field methods (CHARMM36 and GLYCAM06), and the polarizable force field methods (AMOEBA).

Overall, PBDPC25 reproduced the benchmark interaction energies, with an RMSE of 3.50 kcal/mol, which is comparable to the performance of ωB97X-D (4.79 kcal/mol) and M06-2X (3.99 kcal/mol).

The fixed-charge force fields yield larger deviations, with RMSE values of 6.20 kcal/mol and 8.29 kcal/mol, respectively. The polarizable force field AMOEBA shows a comparable level of deviation, with an RMSE of 7.09 kcal/mol. Taken together, these results indicate that PBDPC25 achieves an accuracy approaching that of density functional methods, while providing a clear improvement relative to commonly used molecular mechanics force fields for the systems examined here.

Inspection of the system-specific data reveals that PBDPC25 achieves balanced performance across different stereoisomers. For α-glucose–(H_2_O)_20_, the benchmark interaction energy of −223.52 kcal/mol was predicted as −221.31 kcal/mol, corresponding to a deviation of ~2 kcal/mol.

Similarly, β-mannose–(H_2_O)_20_ was calculated at −212.58 kcal/mol, compared with the benchmark value of −209.86 kcal/mol, resulting in a deviation of ~2 kcal/mol. For β-glucose–(H_2_O)_20_ and β-xylose–(H_2_O)_20_, the deviations were within 2–6 kcal/mol, whereas for α-allose–(H_2_O)_20_ and β-allose–(H_2_O)_20_, the difference from the benchmark was within 1–4 kcal/mol. Despite these variations, the errors remain consistently smaller than those associated with fixed-charge force fields and the polarizable force field, which systematically underestimated the interaction energies by 7–10 kcal/mol across all systems.

Taken together, these results demonstrate that PBDPC25 is capable of accurately describing the hydration interaction energies of carbohydrates, yielding predictions that closely track high-level ab initio data. The balanced accuracy across different carbohydrate stereoisomers suggests that the polarizable bond dipole framework captures the essential physics of solvation without introducing systematic bias toward overbinding or underbinding. Moreover, the near-DFT accuracy achieved at significantly reduced computational cost highlights the potential of PBDPC25 as a reliable alternative for modeling carbohydrate hydration, bridging the gap between computational efficiency and predictive accuracy.

#### 4.2.2. Many-Body Interaction Energies

The ability to reproduce many-body interaction energies is an essential test for evaluating whether a potential can properly capture polarization effects in carbohydrate–water systems [66,67,68]. The total many-body contributions (*IE*_mb_) for six carbohydrate–(H_2_O)_20_ clusters were obtained with the PBDPC25 potential and compared with the benchmark DLPNO-CCSD(T)/CBS (tightPNO) results, as reported in Table 5. Additional reference values were provided by three commonly used density functional methods (ωB97X-D and M06-2X) and the AMOEBA force field.

It can be observed that PBDPC25 reproduces the benchmark many-body interaction energies with high fidelity, yielding an RMSE of 1.44 kcal/mol, which is significantly lower than those of the tested density functional methods (3.61 kcal/mol for ωB97X-D, 2.10 kcal/mol for M06-2X, and 4.82 kcal/mol for B3LYP-D3). The accuracy of the total many-body interaction produced by the AMOEBA force field is comparable to our potential, and the RMSE is 1.59 kcal/mol. This demonstrates that both PBDPC25 and AMOEBA achieve a level of accuracy in describing many-body effects that is comparable to that of the M06-2X density functional method. Hybrid density functionals generally provide reasonable estimates for the total interaction energies dominated by two-body hydrogen bonding. However, as evidenced by the present benchmark results, their description of polarization-driven many-body contributions exhibits larger deviations from the reference data. This behavior is consistent with previous analyses indicating that many commonly used density functional approximations face challenges in accurately capturing nonadditive exchange and polarization effects in molecular trimers and hydrogen-bonded clusters [69]. By contrast, PBDPC25 explicitly accounts for geometry-responsive polarization through bond dipoles and is therefore able to capture cooperative dipole–dipole interactions within carbohydrate–water clusters, underscoring the importance of explicit polarizability at the bond dipole level for an accurate description of solvation effects.

Across the six test systems, PBDPC25 consistently provides balanced estimates of *IE*_mb_, with deviations from the benchmark values remaining within 3 kcal/mol for all clusters. This robustness suggests that the potential is transferable among different carbohydrate topologies and solvation environments. The results further highlight the importance of explicitly incorporating polarization effects into carbohydrate potentials: without such treatment, as evidenced by the density functional methods, the accuracy of many-body interactions deteriorates markedly.

#### 4.2.3. Carbohydrate–Water Interaction and Conformational Energies Under Implicit Solvent Screening

To extend the assessment beyond gas-phase clusters and to examine the behavior of the present framework under dielectric screening, additional calculations were performed using an implicit solvent representation. Rather than constituting explicit solution-phase simulations, these calculations provide a controlled test of local carbohydrate energetics and carbohydrate–water interactions referenced to quantum-chemical data obtained in a screened electrostatic environment.

**Figure 5:**
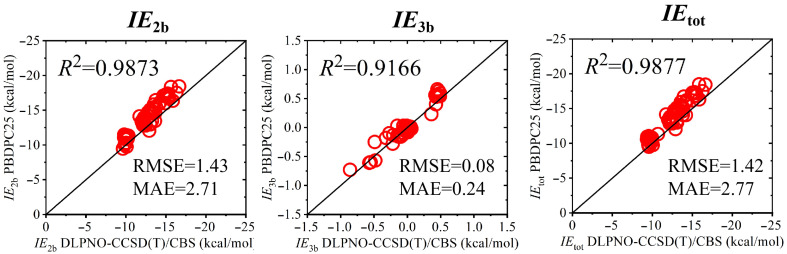
Correlation between PBDPC25 and DLPNO-CCSD(T)/CBS interaction energies for carbohydrate–water–water trimers under CPCM implicit solvation.

Six representative monosaccharides (α-glucose, β-glucose, β-mannose, β-xylose, α-allose, and β-allose) were selected. For each species, ten distinct dihydrated conformations were examined (Figure S2). For each trimer, the two-body, three-body, and total interaction energies were evaluated using the present bond dipole framework with solvent-consistent charge evaluation. In this procedure, the conformation-dependent atomic charges *q* were obtained from AM1 calculations performed under the CPCM implicit water model, thereby incorporating solvent screening effects into the electrostatic description. The resulting interaction energies were compared against DLPNO-CCSD(T)/CBS reference values computed with the same CPCM implicit solvation model. Correlation plots for the two-body, three-body, and total interaction energy components are shown in Figure 5, while detailed numerical results for both interaction and conformational energetics are provided in the Supporting Information.

The results indicate that the present model maintains a robust description of carbohydrate–water interactions under dielectric screening. In particular, the three-body contributions exhibit very small deviations relative to the reference data, consistent with prior benchmark studies on carbohydrate water clusters [55]. The corresponding RMSEs and correlation coefficients for the two-body, three-body, and total interaction energies are summarized in Table S17, and the full set of trimer geometries is provided in Table S20 (Supporting Information).

To further probe conformational energetics under dielectric screening, three representative disaccharides—lactose, maltose, and sucrose—were examined, and ten distinct conformations were considered for each species. Reference conformational energies were obtained at the RIJCOSX-DLPNO-CCSD(T)/CBS level, with solvation free energies evaluated using the SMD implicit solvation model [70]. The resulting RMSE (0) between PBDPC25 and the quantum-chemical reference values is 2.86 kcal/mol, with a corresponding correlation coefficient of *R*^2^ = 0.90. In addition, the RMSE of molecular dipole moments relative to the B3LYP/aug-cc-pVTZ results obtained using the CPCM implicit solvation model is 1.10 D, with an *R*^2^ value of 0.96 (Table S18).

Taken together, these results demonstrate that the bond dipole framework provides a robust and physically consistent description of local carbohydrate energetics and hydration effects when referenced to quantum-chemical data under dielectric screening. While these tests do not constitute a validation of bulk condensed-phase thermodynamic or dynamic properties, they extend the assessment of the present model beyond vacuum benchmarks and provide direct support for its applicability to local hydration phenomena in screened environments.

### 4.3. Computational Efficiency

Table 6 summarizes the computational efficiency of PBDPC25 and AMOEBA in single-point calculations of carbohydrate–water clusters over 1000 cycles, performed on an Intel(R) Xeon(R) E5-2650 v3 @ 2.30 GHz server using a single thread. PBDPC25 consistently requires fewer electrostatic terms (1385–1594; mean 1559) compared with AMOEBA (3045–3360; mean 3308), reflecting a more compact electrostatic representation. This reduction directly translates into improved efficiency: the average CPU time per 1000 cycles is 147 s for PBDPC25 versus 241 s for AMOEBA, respectively.

Overall, PBDPC25 demonstrates clear computational advantages in both electrostatic term scaling and timing, highlighting its efficiency for carbohydrate–water cluster simulations.

## 5. Conclusions

A polarizable bond dipole potential (PBDPC25) has been developed for carbohydrate systems with the aim of providing a physically motivated description of electronic polarization and many-body effects beyond the scope of conventional fixed-charge models. The parameterization was carried out against high-level quantum-chemical reference data, including gas-phase conformational energetics and molecular dipole moments, establishing a rigorous foundation for local intramolecular and intermolecular interactions.

Systematic benchmarks of conformational energetics, optimized geometries, dipole properties, and carbohydrate–water cluster interactions demonstrate that PBDPC25 yields a balanced and internally consistent description in vacuum and local hydration environments. For carbohydrate–water clusters, including both gas-phase and implicitly solvated trimers, the present model captures the total interaction energies and many-body contributions with accuracy comparable to M06-2X/aug-cc-pVTZ and ωB97X-D/aug-cc-pVTZ methods.

The current implementation establishes a solid methodological foundation for future developments of bond-dipole-based force fields for carbohydrates. The validated treatment of intramolecular polarization in carbohydrate monomers provides a transferable basis for extending the framework to more complex carbohydrate systems, including oligosaccharides and polysaccharides. Extension of the present carbohydrate model to large systems and long timescales will require further software optimization, including GPU acceleration, efficient polarization updates, and the incorporation of long-range electrostatics. Ongoing efforts in these directions are expected to enable future applications for carbohydrate hydration, conformational dynamics, and intermolecular interactions in aqueous environments.

## Figures and Tables

**Table 1:** Molecular dipole moments (D) for the seven stable carbohydrates used in electrostatic parameter fitting. (*μₓ*, *μᵧ*, *μ_z_* are Cartesian components of the dipole vector in the molecular-fixed frame; *μ*_M_ denotes the magnitude of the total molecular dipole moment).

Carbohydrate	B3LYP/aug-cc-pVTZ				PBDPC25				Error			
	*μ* *_x_*	*μ* *_y_*	*μ* *_z_*	*μ*_M_	*μ* *_x_*	*μ* *_y_*	*μ* *_z_*	*μ*_M_	*δμ* *_x_*	*δμ* *_y_*	*δμ* *_z_*	*δμ*_M_
α-glucose	−1.56	−3.29	0.16	3.64	−1.28	−3.32	0.35	3.58	0.28	−0.03	0.19	−0.06
β-glucose	1.99	−2.12	0.10	2.91	2.07	−2.16	0.00	2.99	0.08	−0.04	−0.10	0.08
β-xylose	−0.57	−1.63	−0.53	1.80	−0.70	−1.59	−0.20	1.75	−0.13	0.04	0.33	−0.05
β-mannose	−0.78	1.21	1.56	2.12	−1.16	1.46	1.62	2.46	−0.38	0.25	0.06	0.34
α-allose	−1.83	−2.33	0.91	3.10	−1.95	−2.38	0.60	3.14	−0.12	−0.05	−0.31	0.04
β-allose	1.81	2.54	2.07	3.74	2.03	2.55	1.76	3.70	0.22	0.01	−0.31	−0.04
α-maltose	1.38	0.30	2.82	3.15	1.55	−0.05	3.30	3.65	0.17	−0.35	0.48	0.50
RMSE									0.22	0.17	0.29	0.23

**Table 2:** RMSE and MAE of relative conformational energy (kcal/mol) for carbohydrates computed using different force fields, referenced to vacuum CCSD(T) or DLPNO-CCSD(T) benchmark data.

Carbohydrates	GLYCAM06			CHARMM36			AMOEBA			PBDPC25		
	RMSE (0)	RMSE (each)	MAE (each)	RMSE (0)	RMSE (each)	MAE (each)	RMSE (0)	RMSE (each)	MAE (each)	RMSE (0)	RMSE (each)	MAE (each)
α-glucose ^[a]^	3.69	5.09	16.00	3.68	3.84	10.48	2.69	3.41	10.30	2.50	3.37	9.56
β-glucose ^[a]^	3.58	3.80	11.85	3.90	3.07	8.36	2.39	3.37	9.70	1.94	2.45	7.29
α-maltose ^[a]^	4.29	5.18	25.19	4.60	4.99	18.57	3.94	5.33	20.44	2.69	3.46	12.73
β-xylose ^[b]^	2.71	3.17	11.08	6.00	3.56	12.45	2.11	1.96	6.46	2.17	2.93	7.95
β-mannose ^[b]^	3.60	4.59	16.81	4.89	3.77	11.95	3.54	4.10	12.72	2.48	3.34	12.15
α-allose ^[c]^	1.15	1.54	3.33	1.04	1.48	3.27	1.80	2.54	4.95	1.76	2.48	5.60
β-allose ^[c]^	1.10	1.03	1.90	2.08	1.72	3.07	3.25	3.02	5.41	1.40	1.34	2.65
Average	2.87	3.49	12.31	3.74	3.20	9.74	2.82	3.39	10.00	2.13	2.77	8.28

^[a]^ Error metrics were evaluated using relative energies, with DLPNO-CCSD(T)/CBS data from ref. [57] as reference. ^[b]^ Error metrics were evaluated using relative energies, with CCSD(T)/6-311+G(d,p) data from ref. [22] as reference. ^[c]^ Error metrics were evaluated using relative energies, with CCSD(T)/CBS data from ref. [21] as reference.

**Table 3:** RMSE (Å) of heavy atom positions for the seven most stable carbohydrates obtained from different force fields relative to reference geometries.

Carbohydrate	GLYCAM06	CHARMM36	AMOEBA	PBDPC25
α-glucose ^[a]^	0.14	0.08	0.09	0.14
β-glucose ^[a]^	0.17	0.07	0.12	0.09
α-maltose ^[a]^	0.45	0.42	0.37	0.30
β-xylose ^[b]^	0.15	0.07	0.05	0.13
β-mannose ^[b]^	0.13	0.12	0.14	0.16
α-allose ^[c]^	0.26	0.10	0.13	0.14
β-allose ^[c]^	0.20	0.09	0.10	0.12
Average	0.21	0.14	0.14	0.15

^[a]^ Reference geometries were taken from the lowest-energy conformers reported in ref. [57], optimized at the MP2/def2-TZVPP level. ^[b]^ Reference geometries were taken from the lowest-energy conformers reported in ref. [22], optimized at the B3LYP/6-311+G(2df,p) level. ^[c]^ Reference geometries were taken from the lowest-energy conformers reported in ref. [21], optimized at the B3LYP/6-31+G(d,p) level.

**Table 4:** Total interaction energies *IE*_tot_ (kcal/mol) for the six carbohydrate–(H_2_O)_20_ clusters computed using different methods.

Carbohydrate	DLPNO-CCSD(T)/CBS	ωB97X-D ^[a]^	M06-2X ^[a]^	GLYCAM06	CHARMM36	AMOEBA	PBDPC25
α-glucose–(H_2_O)_20_	−223.52	−229.23	−229.4	−221.44	−213.19	−214.81	−221.31
β-glucose–(H_2_O)_20_	−226.28	−231.68	−230.04	−216.20	−215.25	−218.88	−228.51
β-xylose–(H_2_O)_20_	−218.82	−223.83	−221.58	−213.91	−212.80	−213.32	−224.87
β-mannose–(H_2_O)_20_	−209.86	−213.46	−213.6	−206.79	−204.04	−204.15	−212.58
α-allose–(H_2_O)_20_	−198.03	−201.98	−200.84	−189.78	−189.58	−191.64	−196.24
β-allose–(H_2_O)_20_	−221.80	−226.51	−225.96	−216.99	−215.30	−213.60	−225.87
RMSE		4.79	3.99	6.20	8.29	7.09	3.50

^[a]^ with aug-cc-pVTZ basis set.

**Table 5:** Total many-body energies *IE*_mb_ (kcal/mol) for the six carbohydrate–(H_2_O)_20_ clusters computed using different methods.

Carbohydrate	DLPNO-CCSD(T)/CBS	ωB97X-D ^[a]^	M06-2X ^[a]^	AMOEBA	PBDPC25
α-glucose–(H_2_O)_20_	−43.51	−47.86	−42.40	−45.14	−46.00
β-glucose–(H_2_O)_20_	−50.02	−52.04	−46.46	−50.15	−49.49
β-xylose–(H_2_O)_20_	−49.75	−53.68	−47.96	−52.14	−51.29
β-mannose–(H_2_O)_20_	−41.07	−44.86	−39.73	−42.77	−42.20
α-allose–(H_2_O)_20_	−36.47	−39.22	−34.36	−37.03	−36.36
β-allose–(H_2_O)_20_	−46.92	−51.16	−45.16	−48.81	−48.42
RMSE		3.61	2.10	1.59	1.44

^[a]^ With aug-cc-pVTZ basis set.

**Table 6:** Electrostatic term counts and CPU time (s) for 1000 single-point energy evaluations using PBDPC25 and AMOEBA.

Carbohydrate	AMOEBA		PBDPC25	
	Elec Term	CPU Time	Elec Term	CPU Time
α-glucose–(H_2_O)_20_	3360	238	1594	149
β-glucose–(H_2_O)_20_	3360	266	1594	145
β-xylose–(H_2_O)_20_	3045	234	1385	141
β-mannose–(H_2_O)_20_	3360	239	1594	147
α-allose–(H_2_O)_20_	3360	235	1594	149
β-allose–(H_2_O)_20_	3360	235	1594	148
Average	3308	241	1559	147

## Data Availability

The original contributions presented in this study are included in the article/Supplementary Materials. Further inquiries can be directed to the corresponding author.

## Supplementary Materials

The following supporting information can be downloaded at: https://www.mdpi.com/article/10.3390/molecules31030533/s1, Figure S1: Correlation of conformational energies for carbohydrate monomers computed using PBDPC25, CHARMM36, GLYCAM06, and AMOEBA relative to CCSD(T) levelreference data; Figure S2: Representative structures of carbohydrate–water–water trimers; Table S1: Definition of atom types in the PBDPC25 model; Table S2: Optimized force field parameters for the PBDPC25 model; Table S3: Comparison of root-mean-square errors (RMSEs) for interaction energies (kcal/mol) and molecular dipole moments (D) obtained with different versions of the PBDPC25 parameter set for a test set of 78 carbohydrate–water clusters; Table S4: Relative conformational energies (RE, kcal/mol) of 80 α-glucose conformers computed using DLPNO-CCSD(T)/CBS and the tested force fields; Table S5: Relative conformational energies (RE, kcal/mol) of 76 β-glucose conformers computed using DLPNO-CCSD(T)/CBS and the tested force fields; Table S6: Relative conformational energies (RE, kcal/mol) of 223 α-maltoses conformerscomputed using DLPNO-CCSD(T)/CBS and the tested force fields; Table S7: Relative conformational energies (RE, kcal/mol)of 90 xylose conformers computed using CCSD(T)/6-311+G(d,p) and the tested force fields; Table S8: Relative conformational energies (RE, kcal/mol) of 168 mannose conformers computed using CCSD(T)/6-311+G(d,p) and the tested force fields; Table S9: Relative conformational energies (RE, kcal/mol) of 9 α-allose conformers computed using CCSD(T)/CBS and the tested force fields; Table S10: Relative conformational (RE, kcal/mol) of 6 β-allose conformers computed using CCSD(T)/CBS and the tested force fields; Table S11: Dipole moments (*μ*_M_) in Debye of 80 α-glucose conformers computed by B3LYP/aug-cc-pVTZ and tested force fields; Table S12: Dipole moments (*μ*_M_) in Debye of 76 β-glucose conformers computed by B3LYP/aug-cc-pVTZ and tested force fields; Table S13: Dipole moments (*μ*_M_) in Debye of 223 ɑ-maltose conformers computed by B3LYP/aug-cc-pVTZ and tested force fields; Table S14: Dipole momentsin Debye (*μ*_M_)of 90β-xylose conformers computed by B3LYP/aug-cc-pVTZ and tested force fields; Table S15: Dipole moments (*μ*_M_) in Debye of 168 β-mannose conformers computed by B3LYP/aug-cc-pVTZ and tested force fields; Table S16: Dipole moments (*μ*_M_ in D) of 15α/β-allose conformers computed by B3LYP/aug-cc-pVTZ and force field methods; Table S17: The interaction energies (kcal/mol) of carbohydrate-water-water trimersin solution-phase under CPCM implicit solvation; Table S18: Conformational energies (kcal/mol) and Molecular dipole moments (D) of disaccharides; Table S19: Cartesian coordinates (Å) for six carbohydrate–(H_2_O) 20 clusters; Table S20: Cartesian coordinates (Å) of 60 carbohydrate-water-water trimers.

## Abbreviations

The following abbreviations are used in this manuscript:

PBDPC25	Polarizable bond dipole potential for carbohydrates 2025
MAE	Maximum absolute error
RMSE	Root-mean-square error
MP2	Second-order Møller–Plesset perturbation theory
CCSD(T)	Coupled cluster with singles, doubles, and perturbative triples
DFT	Density functional theory
DLPNO	Domain-based local pair natural orbital
CBS	Complete basis set
CPCM	Conductor-like polarizable continuum model
