# Supplementary material for: Capturing Carbohydrate Conformations and Hydration Interactions with a Polarizable Bond Dipole Potential
Source: Molecules. 2026 Feb 3;31(3):533. doi: 10.3390/molecules31030533 (PMC12899679; doi:10.3390/molecules31030533)
Supplement: Supplementary file 1 [file molecules-31-00533-s001.zip › molecules-4121953-supplementary.pdf]

Supporting information for:

# Capturing Carbohydrate Conformations and Hydration Interactions with a Polarizable Bond Dipole Potential

**Meng-Yao Bai, Xiao-Han Zheng, Shan-Shan Gao, Xiao-Nan Jiang, Jia-Yi Zhu, Qiang Hao, Li-Dong Gong, Lei Wang, Chang-Sheng Wang**

*School of Chemistry and Chemical Engineering, Liaoning Normal University, Dalian, People's Republic of China*

# Contents

**Section 1: Force Field Parameter Definitions and Tables** .......... 3
Table S1. Definition of atom types in the PBDPC25 model .......... 3
Table S2. Optimized force field parameters for the PBDPC25 model .......... 3
Table S3. Comparison of root-mean-square errors (RMSEs) for interaction energies (kcal/mol) and molecular dipole moments (D) obtained with different versions of the PBDPC25 parameter set for a test set of 78 carbohydrate–water clusters .......... 5
**Section 2: Interaction Energy** .......... 5
**Section 3: Error Metrics and Statistical Evaluation** .......... 6
**Section 4: Result** .......... 7
Figure S1. Correlation of conformational energies for carbohydrate monomers computed using PBDPC25, CHARMM36, GLYCAM06, and AMOEBA relative to CCSD(T) level reference data. .......... 7
Table S4. Relative conformational energies (RE, kcal/mol) of 80 α-glucose conformers computed using DLPNO-CCSD(T)/CBS and the tested force fields .......... 8
Table S5. Relative conformational energies (RE, kcal/mol) of 76 β-glucose conformers computed using DLPNO-CCSD(T)/CBS and the tested force fields .......... 10
Table S6. Relative conformational energies (RE, kcal/mol) of 223 α-maltoses conformers computed using DLPNO-CCSD(T)/CBS and the tested force fields .......... 12
Table S7. Relative conformational energies (RE, kcal/mol) of 90 xylose conformers computed using CCSD(T)/6-311+G(d,p) and the tested force fields .......... 17
Table S8. Relative conformational energies (RE, kcal/mol) of 168 mannose conformers computed using CCSD(T)/6-311+G(d,p) and the tested force fields .......... 19
Table S9. Relative conformational energies (RE, kcal/mol) of 9 α-allose conformers computed using CCSD(T)/CBS and the tested force fields .......... 23
Table S10. Relative conformational (RE, kcal/mol) of 6 β-allose conformers computed using CCSD(T)/CBS and the tested force fields .......... 24
Table S11. Dipole moments ($\mu_M$) in Debye of 80 α-glucose conformers computed by B3LYP/aug-cc-pVTZ and tested force fields .......... 24
Table S12. Dipole moments ($\mu_M$) in Debye of 76 β-glucose conformers computed by B3LYP/aug-cc-pVTZ and tested force fields .......... 26
Table S13. Dipole moments ($\mu_M$) in Debye of 223 α-maltose conformers computed by B3LYP/aug-cc-pVTZ and tested force fields .......... 27
Table S14. Dipole moments in Debye ($\mu_M$) of 90 β-xylose conformers computed by B3LYP/aug-cc-pVTZ and tested force fields .......... 33
Table S15. Dipole moments ($\mu_M$) in Debye of 168 β-mannose conformers computed by B3LYP/aug-cc-pVTZ and tested force fields .......... 35
Table S16. Dipole moments ($\mu_M$ in D) of 15 α/β-allose conformers computed by B3LYP/aug-cc-pVTZ and force field methods .......... 39
Figure S2 Representative structures of carbohydrate–water–water trimers .......... 40
Table S17 The interaction energies (kcal/mol) of carbohydrate-water-water trimers in solution-phase under CPCM implicit solvation .......... 41
Table S18 Conformational energies (kcal/mol) and Molecular dipole moments (D) of disaccharides .......... 44
Table S19. Cartesian coordinates (Å) for six carbohydrate–$(H_2O)_{20}$ clusters .......... 45
Table S20. Cartesian coordinates (Å) of 60 carbohydrate-water-water trimers. .......... 51

## Section 1: Force Field Parameter Definitions and Tables

Table S1. Definition of atom types in the PBDPC25 model

| Atom type | Description |
|---|---|
| CT | $sp^3$ carbon in carbohydrate species |
| OS | $sp^3$ oxygen bonded to two CT atoms |
| OH | $sp^3$ oxygen in hydroxyl group |
| HO | hydrogen in hydroxyl group |
| H1 | hydrogen attached to a carbon bonded to one $sp^3$ oxygen |
| H2 | hydrogen attached to a carbon bonded to two $sp^3$ oxygen |
| OW | oxygen atom in water |
| HW | hydrogen atom in water |

Table S2. Optimized force field parameters for the PBDPC25 model

| Parameters for electrostatics | | | | | |
|---|---|---|---|---|---|
| Bond type | $\mu_0$ (Debye) | | $c$ | | $q_0$ (e) |
| CT-OS | **0.70**[a] | | / | | / |
| CT-OH | **0.70**[a] | | / | | / |
| OH-HO | **1.51**[a] | | **1.95**[a] | | **0.2142**[a] |
| CT-H1 | **0.70**[a] | | **1.00**[a] | | **0.0977**[a] |
| CT-H2 | **0.70**[a] | | **1.00**[a] | | **0.1483**[a] |
| OW-HW | 1.51[b] | | 1.86[b] | | 0.1942[b] |
| **Parameters for van der Waals** | | | | | |
| Atom type | $R^*$ (Å) | | $\varepsilon^*$ (kcal/mol) | | |
| CT | 1.9080[d] | | 0.1094[d] | | |
| OS | **1.6500**[a] | | **0.1000**[a] | | |
| OH | 1.7210[d] | | 0.2104[d] | | |
| HO | **0.2245**[a] | | **0.0460**[a] | | |
| H1 | 1.3870[d] | | 0.0157[d] | | |
| H2 | **1.3400**[a] | | **0.0350**[a] | | |
| OW | 1.7820[c] | | 0.1634[c] | | |
| HW | 0.0000[c] | | 0.0000[c] | | |
| **Parameters for bond stretching**[d] | | | | | |
| Bond | $K_b$(kcal.mol$^{-1}$.Å$^{-2}$) | $b_0$ (Å) | Bond | $K_b$ (kcal.mol$^{-1}$.Å$^{-2}$) | $b_0$ (Å) |
| CT-CT | 310.00 | 1.5200 | CT-H2 | 340.00 | 1.0900 |
| CT-OS | 285.00 | 1.4600 | OH-HO | 553.00 | 0.9600 |
| CT-OH | 320.00 | 1.4300 | OW-HW | 553.00 | 0.9572 |
| CT-H1 | 340.00 | 1.0900 | | | |
| **Parameters for angle bending**[d] | | | | | |
| Angle | $K_\theta$ (kcal.mol$^{-1}$.rad$^{-2}$) | $\theta_0$ (°) | Angle | $K_\theta$ (kcal.mol$^{-1}$.rad$^{-2}$) | $\theta_0$ (°) |
| CT-CT-CT | 45.00 | 109.50 | CT-OH-HO | 55.00 | 109.50 |

| | | | | | |
|---|---|---|---|---|---|
| CT-CT-H1 | 45.00 | 113.50 | CT-OS-CT | 50.00 | 111.60 |
| H1-CT-H1 | 45.00 | 111.00 | OS-CT-H1 | 60.00 | 110.00 |
| CT-CT-OS | 70.00 | 112.60 | H2-CT-CT | 45.00 | 111.00 |
| OS-CT-OH | 100.00 | 109.50 | H2-CT-OH | 60.00 | 110.00 |
| OH-CT-H1 | 60.00 | 108.50 | H2-CT-OS | 60.00 | 110.00 |
| OS-CT-OS | 100.00 | 112.00 | HW-OW-HW | 100.00 | 104.52 |
| CT-CT-OH | 70.00 | 107.50 | | | |

Parameters for dihedral torsion[e]

| Torsion | $V_n$ (kcal/mol) | $\gamma_n$ (°) | $n$ | Torsion | $V_n$ (kcal/mol) | $\gamma_n$ (°) | $n$ |
|---|---|---|---|---|---|---|---|
| CT-CT-OS-CT | 0.16 | 0.00 | 3 | CT-CT-CT-OS | **1.00**[a] | **180.0**[a] | 1 |
| CT-CT-OH-HO | 0.18 | 0.00 | 3 | OS-CT-OS-CT | **1.37** [a] | 0.00 | 3 |
| CT-CT-CT-CT | 0.45 | 0.00 | 1 | | 1.27 | 0.00 | 2 |
| HC-CT-CT-OH | 0.05 | 0.00 | 3 | | 0.30 | 0.00 | 1 |
| H1-CT-CT-CT | 0.15 | 0.00 | 3 | OS-CT-CT-OS | **0.42**[a] | 0.00 | 2 |
| H1-CT-CT-H1 | 0.17 | 0.00 | 3 | OH-CT-CT-OS | **1.10**[a] | **180.0**[a] | 1 |
| H1-CT-CT-OS | 0.05 | 0.00 | 3 | | 0.25 | 0.00 | 2 |
| OH-CT-CT-CT | 0.10 | 0.00 | 3 | OH-CT-CT-OH | **0.10**[a] | **180.0**[a] | 1 |
| OH-CT-CT-H1 | 0.05 | 0.00 | 3 | | 0.95 | 0.00 | 2 |
| H1-CT-OH-HO | 0.18 | 0.00 | 3 | | 0.55 | 0.00 | 3 |
| H1-CT-OS-CT | 0.27 | 0.00 | 3 | OH-CT-OS-CT | **1.37**[a] | 0.00 | 3 |
| H1-CT-CT-H2 | 0.17 | 0.00 | 3 | | 1.27 | 0.00 | 2 |
| H2-CT-CT-CT | 0.15 | 0.00 | 3 | | 0.30 | 0.00 | 1 |
| OH-CT-CT-H2 | 0.05 | 0.00 | 3 | H2-CT-OS-CT | 0.00 | 180.00 | 1 |
| OS-CT-OH-HO | **1.18**[a] | 0.00 | 3 | | **0.00**[a] | 0.00 | 2 |
| HO-OH-CT-H2 | 0.18 | 0.00 | 3 | | **0.00**[a] | 0.00 | 3 |

Parameters for orbital overlap[c]

| Orbital | $D$ (kcal/mol) | $a$ (Å$^{-1}$) | $R_0$ (Å) | $\alpha_0$ (°) | $\beta_0$ (°) |
|---|---|---|---|---|---|
| OW-HW ··OW<br>OH-HO ··OW<br>OW-HW ··OH<br>OW-HW ··OS | 1.65 | 1.10 | 1.944 | 180.00 | 109.47 |

Scale factors for intramolecular interactions

| | |
|---|---|
| two dipoles separated by three bonds | 1.0 |
| van der Waals interactions separated by two atoms | 0.5 |

[a] The parameters in bold numbers are determined in this work.

[b] These parameters are taken from our previous work (*J. Comput. Chem. 2023, 44, 677-686*).

[c] These parameters are taken from our previous work (*J. Chem. Theory Comput*. 2025, *21*, 11049-11069).

[d] These parameters are taken from GLYCAM06 force field (*J. Comput. Chem*. 2008, *29*, 622-655).

[e] The parameters in normal numbers are taken from GLYCAM06 force field (*J. Comput. Chem*. 2008, *29*, 622-655).

Table S3. Comparison of root-mean-square errors (RMSEs) for interaction energies (kcal/mol) and molecular dipole moments (D) obtained with different versions of the PBDPC25 parameter set for a test set of 78 carbohydrate–water clusters

| Error | $IE_{\text{tot}}$ | | $IE_{\text{2b}}$ | | $IE_{\text{3b}}$ | | $\mu_{\text{M}}$ | |
|---|---|---|---|---|---|---|---|---|
| | Previous[a] | Current | Previous[a] | Current | Previous[a] | Current | Previous[a] | Current |
| RMSE | 3.68 | 3.62 | 3.92 | 3.84 | 0.87 | 0.85 | 0.47 | 0.51 |
| MAE | 13.33 | 13.82 | 13.38 | 13.69 | 3.13 | 3.10 | 1.80 | 1.63 |
| MRE | 3.03% | 2.93% | 4.16% | 3.88% | 9.34% | 9.12% | 9.78% | 9.98% |
| $R^2$ | 0.9985 | 0.9986 | 0.9974 | 0.9975 | 0.9977 | 0.9978 | 0.9894 | 0.9872 |

[a] *Chem. Phys. Lett.* 2025, *876* , 142230

## Section 2: Interaction Energy

The many-body interaction energies of carbohydrate–water clusters were analyzed using a standard energy decomposition scheme based on the many-body expansion.

The individual two-body interaction energy between monomers *i* and *j* is defined as：

$$V_{\text{2b}}(i,j) = E(i,j) - E(i) - E(j) \tag{S1}$$

where $E(i,j)$ is the total electronic energy of the dimer composed of monomers *i* and *j*, and $E(i)$ and $E(j)$ are the corresponding monomer energies computed in isolation using the same level of theory.

The individual three-body interaction energy among monomers *i*, *j*, and *k* is defined as

$$\begin{aligned} V_{\text{3b}}(i,j,k) &= E(i,j,k) - E(i,j) - E(j,k) - E(i,k) + E(i) + E(j) + E(k) \\ &= E(i,j,k) - [E(i,j) - E(i) - E(j)] - [E(j,k) - E(j) - E(k)] \\ &\quad - [E(i,k) - E(i) - E(k)] - E(i) - E(j) - E(k) \\ &= E(i,j,k) - V_{\text{2b}}(i,j) - V_{\text{2b}}(j,k) - V_{\text{2b}}(i,k) - E(i) - E(j) - E(k) \end{aligned} \tag{S2}$$

where $E(i,j,k)$ is the total electronic energy of the trimer, and the pairwise and monomer energies are defined analogously. In these expressions, *i*, *j*, and *k* label distinct molecular monomers (carbohydrate or water) within the cluster. All energies are evaluated at consistent geometries and levels of theory to ensure a well-defined many-body decomposition. By construction, $V_{\text{3b}}$ represents the cooperative interaction that cannot be captured by a sum of pairwise contributions.

The total two-body interaction energy ($IE_{\text{2b}}$) of the system was obtained by summing up the two-body interaction energies ($V_{\text{2b}}$) within the carbohydrate-water cluster, expressed as Equation (S3). The total three-body interaction energy ($IE_{\text{3b}}$) of the system was obtained by summing up the three-body interaction energies ($V_{\text{3b}}$) within the carbohydrate-water cluster, expressed as Equation (S4).

$$IE_{\text{2b}} = \sum V_{\text{2b}}(i,j) \tag{S3}$$

$$IE_{\text{3b}} = \sum V_{\text{3b}}(i,j,k) \tag{S4}$$

The total interaction energies ($IE_{\text{tot}}$) within the carbohydrate-water systems are calculated by subtracting the total energy of the cluster from each monomer separately, and can be expressed as Equation (S5).

$$IE_{\text{tot}} = E(i,j,\cdots,n) - E(i) - E(j) - \cdots - E(n) \tag{S5}$$

The total many-body interaction energy ($IE_{\mathrm{mb}}$) of the system is calculated by the difference between the total interaction energy and the total two-body interaction energy, and can be expressed as Equation (S6)

$$IE_{\mathrm{mb}} = IE_{\mathrm{tot}} - IE_{\mathrm{2b}} \tag{S6}$$

## Section 3: Error Metrics and Statistical Evaluation

The accuracy of different method to the benchmark value is evaluated by the root-mean-square error (RMSE), maximum absolute error (MAE), mean relative error (MRE), and coefficient of determination ($R^2$), where $y_i$ denote the calculated data (including interaction energy, or molecular dipole moment, or atom position). $y_i^{\mathrm{ref}}$ correspond to the calculated or reference data at the benchmark level, with error metrics defined in Equations (S7-S10).

$$\mathrm{RMSE} = \sqrt{\frac{1}{N}\sum_{i=1}^{N}(y_i - y_i^{\mathrm{ref}})^2} \tag{S7}$$

$$\mathrm{MAE} = \max_{i \in N} | y_i - y_i^{\mathrm{ref}} | \tag{S8}$$

$$\mathrm{MRE} = \frac{1}{N}\sum_{i=1}^{N} \left| \frac{y_i - y_i^{\mathrm{ref}}}{y_i^{\mathrm{ref}}} \right| \tag{S9}$$

$$R^2 = 1 - \frac{\sum_{i=1}^{N}(y_i - y_i^{\mathrm{ref}})^2}{\sum_{i=1}^{N}(y_i^{\mathrm{ref}})^2} \tag{S10}$$

The statistical metrics RMSE(0), RMSE(each), and MAE(each) were computed as follows:

$$\mathrm{RMSE}(0) = \sqrt{\frac{1}{N-1}\sum_{i=2}^{N}[(E_i^{\mathrm{calc}} - E_{\mathrm{min}}^{\mathrm{calc}}) - (E_i^{\mathrm{ref}} - E_{\mathrm{min}}^{\mathrm{ref}})]^2} \tag{S11}$$

$$\mathrm{RMSE(each)} = \sqrt{\frac{1}{N(N\text{-}1)}\sum_{i=1}^{N}\sum_{j \neq i}^{N}[(E_j^{\mathrm{calc}} - E_i^{\mathrm{calc}}) - (E_j^{\mathrm{ref}} - E_i^{\mathrm{ref}})]^2} \tag{S12}$$

$$\mathrm{MAE(each)} = \max_{i, j>i} | (E_j^{\mathrm{calc}} - E_i^{\mathrm{calc}}) - (E_j^{\mathrm{ref}} - E_j^{\mathrm{ref}}) | \tag{S13}$$

where $E_i^{\mathrm{calc}}$ (or $E_j^{\mathrm{calc}}$) and $E_i^{\mathrm{ref}}$ (or $E_j^{\mathrm{ref}}$) denote the calculated and reference (benchmark) energies of conformer *i* (or *j*), respectively. $E_{\mathrm{min}}^{\mathrm{calc}}$ and $E_{\mathrm{min}}^{\mathrm{ref}}$ correspond to the calculated and reference energies of the lowest-energy conformer at the benchmark level.

## Section 4: Result

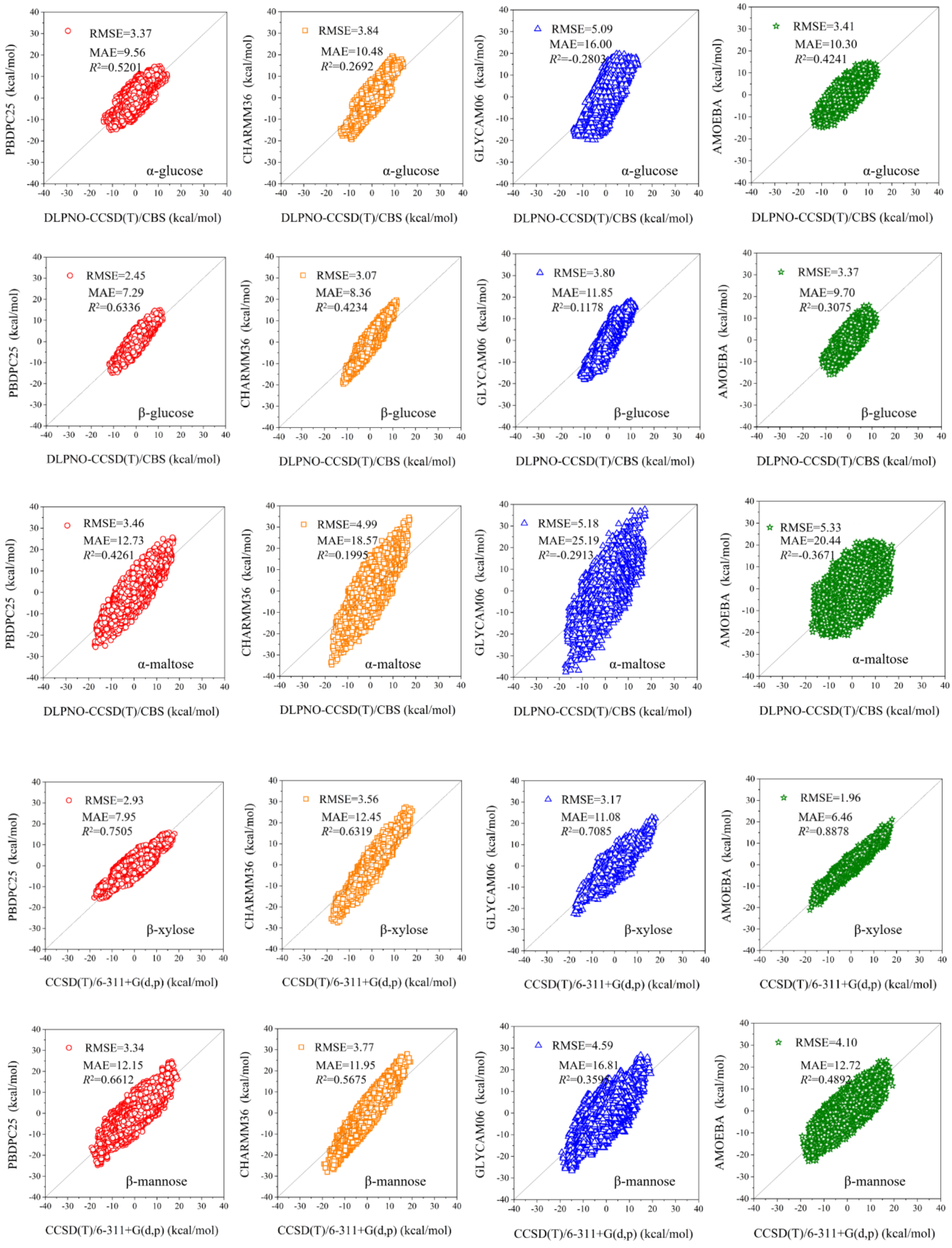


Figure S1. Correlation of conformational energies for carbohydrate monomers computed using PBDPC25, CHARMM36, GLYCAM06, and AMOEBA relative to CCSD(T) level reference data.

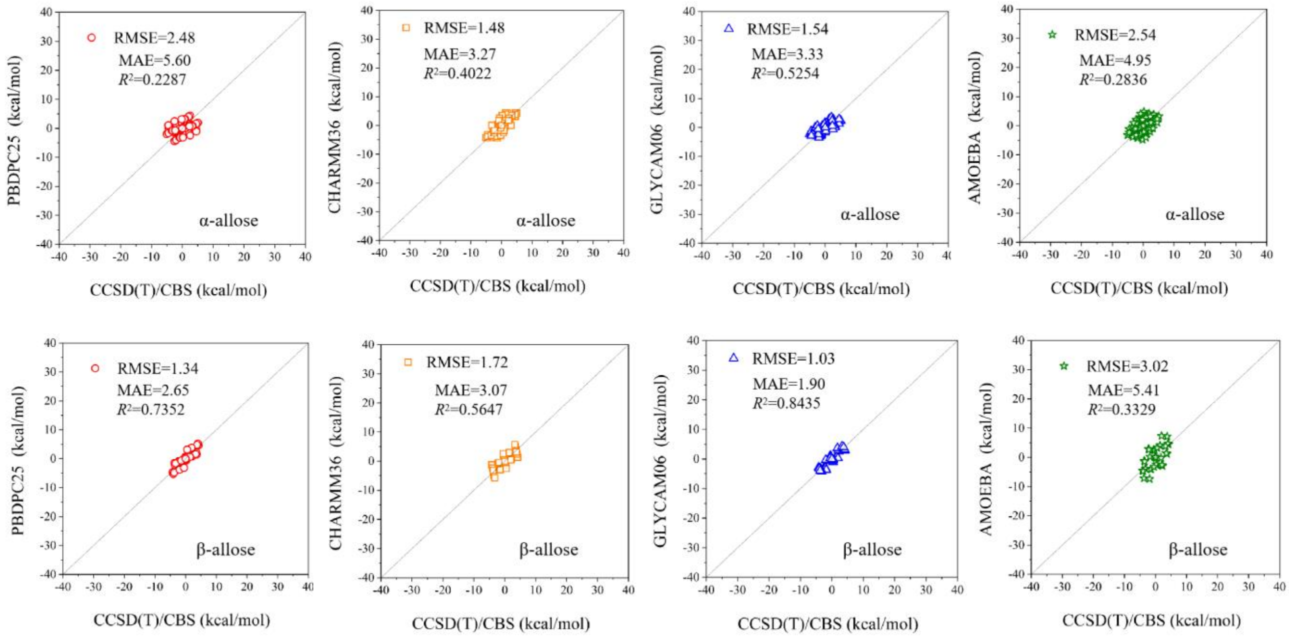


Figure S1. Continued

Table S4. Relative conformational energies (RE, kcal/mol) of 80 α-glucose conformers computed using DLPNO-CCSD(T)/CBS and the tested force fields

| Label | DLPNO-CCSD(T)/CBS | PBDPC25 | | CHARMM36 | | GLYCAM06 | | AMOEBA | |
|---|---|---|---|---|---|---|---|---|---|
| | | RE | Δ | RE | Δ | RE | Δ | RE | Δ |
| 1 | 0.23 | -0.37 | -0.60 | -1.99 | -2.22 | -1.48 | -1.71 | -1.64 | -1.87 |
| 2 | 0.00 | 0.00 | 0.00 | 0.00 | 0.00 | 0.00 | 0.00 | 0.00 | 0.00 |
| 3 | 0.10 | -0.94 | -1.04 | -1.16 | -1.26 | -0.61 | -0.71 | 0.35 | 0.25 |
| 4 | 1.14 | 0.12 | -1.02 | -0.24 | -1.38 | -0.18 | -1.32 | 2.13 | 0.99 |
| 5 | 1.28 | -0.86 | -2.14 | -0.50 | -1.78 | -0.01 | -1.29 | 1.16 | -0.12 |
| 6 | 1.41 | -0.73 | -2.14 | 0.52 | -0.89 | -0.96 | -2.37 | 0.71 | -0.70 |
| 7 | 1.90 | 1.41 | -0.49 | 0.55 | -1.35 | 1.95 | 0.05 | 0.12 | -1.78 |
| 8 | 2.80 | 0.27 | -2.53 | 2.97 | 0.17 | 3.87 | 1.07 | 4.67 | 1.87 |
| 9 | 2.87 | -0.61 | -3.48 | 1.78 | -1.09 | 2.71 | -0.16 | 5.15 | 2.28 |
| 10 | 2.48 | -0.27 | -2.75 | 1.16 | -1.32 | 2.07 | -0.41 | 0.60 | -1.88 |
| 11 | 2.54 | 0.71 | -1.83 | 3.00 | 0.46 | 2.36 | -0.18 | 5.54 | 3.00 |
| 12 | 2.41 | -0.64 | -3.05 | 2.20 | -0.21 | 1.32 | -1.09 | -1.16 | -3.57 |
| 13 | 3.28 | 2.16 | -1.12 | 3.66 | 0.38 | 5.15 | 1.87 | 2.90 | -0.38 |
| 14 | 2.46 | 0.56 | -1.90 | 3.07 | 0.61 | 2.41 | -0.05 | -0.69 | -3.15 |
| 15 | 5.65 | 8.55 | 2.90 | 12.89 | 7.24 | 13.30 | 7.65 | 8.73 | 3.08 |
| 16 | 5.31 | 8.21 | 2.90 | 11.43 | 6.12 | 12.11 | 6.80 | 7.25 | 1.94 |
| 17 | 4.91 | 8.73 | 3.82 | 10.58 | 5.67 | 11.90 | 6.99 | 5.38 | 0.47 |
| 18 | 5.33 | 2.17 | -3.16 | 5.29 | -0.04 | -2.99 | -8.32 | -0.58 | -5.91 |
| 19 | 6.53 | 6.53 | 0.00 | 12.09 | 5.56 | 10.76 | 4.23 | 4.27 | -2.26 |
| 20 | 6.27 | 8.48 | 2.21 | 13.46 | 7.19 | 13.95 | 7.68 | 10.01 | 3.74 |
| 21 | 5.30 | 3.17 | -2.13 | 7.55 | 2.25 | -0.91 | -6.21 | 1.20 | -4.10 |
| 22 | 5.93 | 3.52 | -2.41 | 7.41 | 1.48 | -0.44 | -6.37 | 2.34 | -3.59 |
| 23 | 7.12 | 8.04 | 0.92 | 9.14 | 2.02 | 6.95 | -0.17 | 5.14 | -1.98 |

| | | | | | | | | | |
|---|---|---|---|---|---|---|---|---|---|
| 24 | 6.54 | 4.20 | -2.34 | 6.08 | -0.46 | -1.41 | -7.95 | 2.08 | -4.46 |
| 25 | 7.11 | 8.68 | 1.57 | 14.78 | 7.67 | 13.70 | 6.59 | 6.03 | -1.08 |
| 26 | 7.71 | 10.69 | 2.98 | 13.57 | 5.86 | 14.84 | 7.13 | 8.64 | 0.93 |
| 27 | 6.89 | 5.08 | -1.81 | 11.90 | 5.01 | 4.70 | -2.19 | 7.81 | 0.92 |
| 28 | 8.33 | 7.64 | -0.69 | 10.18 | 1.85 | 8.08 | -0.25 | 7.49 | -0.84 |
| 29 | 6.63 | 1.89 | -4.74 | 8.64 | 2.01 | 3.58 | -3.05 | 7.90 | 1.27 |
| 30 | 8.14 | 9.43 | 1.29 | 13.00 | 4.86 | 10.31 | 2.17 | 9.05 | 0.91 |
| 31 | 6.71 | 1.22 | -5.49 | 9.10 | 2.39 | 3.89 | -2.82 | 5.22 | -1.49 |
| 32 | 6.35 | 1.84 | -4.51 | 7.07 | 0.72 | 0.20 | -6.15 | 2.80 | -3.55 |
| 33 | 6.57 | 8.44 | 1.87 | 12.35 | 5.78 | 13.00 | 6.43 | 8.97 | 2.40 |
| 34 | 8.19 | 11.99 | 3.80 | 15.83 | 7.64 | 15.10 | 6.91 | 8.28 | 0.09 |
| 35 | 7.59 | 6.93 | -0.66 | 13.38 | 5.79 | 10.12 | 2.53 | 9.06 | 1.47 |
| 36 | 9.09 | 6.12 | -2.97 | 11.31 | 2.22 | 7.60 | -1.49 | 2.68 | -6.41 |
| 37 | 7.35 | 6.94 | -0.41 | 9.62 | 2.27 | 4.36 | -2.99 | 6.72 | -0.63 |
| 38 | 8.51 | 12.38 | 3.87 | 9.41 | 0.90 | 9.50 | 0.99 | 8.57 | 0.06 |
| 39 | 7.54 | 6.51 | -1.03 | 9.83 | 2.29 | 2.67 | -4.87 | 5.59 | -1.95 |
| 40 | 8.53 | 7.05 | -1.48 | 8.91 | 0.38 | 8.58 | 0.05 | 8.25 | -0.28 |
| 41 | 8.93 | 7.93 | -1.00 | 11.12 | 2.19 | 8.22 | -0.71 | 6.64 | -2.29 |
| 42 | 9.31 | 10.09 | 0.78 | 13.02 | 3.71 | 9.29 | -0.02 | 3.73 | -5.58 |
| 43 | 9.34 | 9.22 | -0.12 | 13.84 | 4.50 | 10.61 | 1.27 | 6.24 | -3.10 |
| 44 | 9.02 | 8.68 | -0.34 | 17.13 | 8.11 | 14.52 | 5.50 | 8.35 | -0.67 |
| 45 | 9.26 | 9.35 | 0.09 | 9.64 | 0.38 | 8.39 | -0.87 | 4.81 | -4.45 |
| 46 | 7.94 | 2.28 | -5.66 | 9.21 | 1.27 | 4.57 | -3.37 | 9.53 | 1.59 |
| 47 | 9.11 | 5.94 | -3.17 | 12.02 | 2.91 | 6.05 | -3.06 | 9.28 | 0.17 |
| 48 | 9.09 | 8.01 | -1.08 | 10.00 | 0.91 | 12.72 | 3.63 | 8.86 | -0.23 |
| 49 | 7.91 | 10.23 | 2.32 | 9.45 | 1.54 | 9.38 | 1.47 | 7.32 | -0.59 |
| 50 | 8.48 | 6.85 | -1.63 | 9.74 | 1.26 | 9.80 | 1.32 | 8.28 | -0.20 |
| 51 | 8.86 | 7.40 | -1.46 | 8.62 | -0.24 | 10.19 | 1.33 | 6.40 | -2.46 |
| 52 | 9.89 | 9.82 | -0.07 | 15.92 | 6.03 | 12.41 | 2.52 | 7.51 | -2.38 |
| 53 | 9.13 | 8.54 | -0.59 | 11.00 | 1.87 | 13.24 | 4.11 | 8.75 | -0.38 |
| 54 | 9.13 | 7.42 | -1.71 | 9.26 | 0.13 | 10.93 | 1.80 | 8.98 | -0.15 |
| 55 | 9.82 | 7.03 | -2.79 | 11.42 | 1.60 | 13.42 | 3.60 | 13.24 | 3.42 |
| 56 | 9.94 | 10.91 | 0.97 | 14.15 | 4.21 | 16.89 | 6.95 | 6.87 | -3.07 |
| 57 | 5.10 | 7.74 | 2.64 | 11.54 | 6.44 | 8.69 | 3.59 | 8.99 | 3.89 |
| 58 | 10.14 | 11.57 | 1.43 | 13.69 | 3.55 | 14.95 | 4.81 | 9.82 | -0.32 |
| 59 | 9.16 | 8.51 | -0.65 | 9.47 | 0.31 | 8.38 | -0.78 | 7.08 | -2.08 |
| 60 | 9.62 | 10.15 | 0.53 | 12.61 | 2.99 | 7.35 | -2.27 | 5.97 | -3.65 |
| 61 | 9.95 | 9.29 | -0.66 | 9.69 | -0.26 | 12.52 | 2.57 | 10.36 | 0.41 |
| 62 | 9.59 | 9.79 | 0.20 | 17.11 | 7.52 | 12.81 | 3.22 | 9.83 | 0.24 |
| 63 | 9.35 | 10.28 | 0.93 | 10.64 | 1.29 | 8.42 | -0.93 | 5.49 | -3.86 |
| 64 | 10.33 | 6.59 | -3.74 | 13.02 | 2.69 | 10.99 | 0.66 | 8.73 | -1.60 |
| 65 | 8.93 | 11.26 | 2.33 | 12.38 | 3.45 | 10.94 | 2.01 | 6.21 | -2.72 |
| 66 | 9.50 | 7.22 | -2.28 | 11.78 | 2.28 | 6.80 | -2.70 | 4.45 | -5.05 |
| 67 | 9.31 | 9.87 | 0.56 | 12.14 | 2.83 | 9.46 | 0.15 | 6.20 | -3.11 |
| 68 | 9.15 | 12.11 | 2.96 | 17.41 | 8.26 | 12.19 | 3.04 | 9.37 | 0.22 |

| | | | | | | | | | |
|---|---|---|---|---|---|---|---|---|---|
| 69 | 8.94 | 12.20 | 3.26 | 15.71 | 6.77 | 11.23 | 2.29 | 9.35 | 0.41 |
| 70 | 9.34 | 12.24 | 2.90 | 16.35 | 7.01 | 13.18 | 3.84 | 7.71 | -1.63 |
| 71 | 11.14 | 9.66 | -1.48 | 12.58 | 1.44 | 16.83 | 5.69 | 12.94 | 1.80 |
| 72 | 10.69 | 11.40 | 0.71 | 12.87 | 2.18 | 9.54 | -1.15 | 7.08 | -3.61 |
| 73 | 9.40 | 11.12 | 1.72 | 11.83 | 2.43 | 11.75 | 2.35 | 7.12 | -2.28 |
| 74 | 11.52 | 6.74 | -4.78 | 12.53 | 1.01 | 13.10 | 1.58 | 9.52 | -2.00 |
| 75 | 9.86 | 7.60 | -2.26 | 9.31 | -0.55 | 8.03 | -1.83 | 4.05 | -5.81 |
| 76 | 11.58 | 9.11 | -2.47 | 12.90 | 1.32 | 12.05 | 0.47 | 9.27 | -2.31 |
| 77 | 12.08 | 7.31 | -4.77 | 15.69 | 3.61 | 13.80 | 1.72 | 7.90 | -4.18 |
| 78 | 12.02 | 9.52 | -2.50 | 13.84 | 1.82 | 14.47 | 2.45 | 7.50 | -4.52 |
| 79 | 13.22 | 8.12 | -5.10 | 15.90 | 2.68 | 16.66 | 3.44 | 12.40 | -0.82 |
| 80 | 13.84 | 10.08 | -3.76 | 14.48 | 0.64 | 14.47 | 0.63 | 9.28 | -4.56 |
| RMSE(0) | | | 2.50 | | 3.68 | | 3.69 | | 2.69 |

Table S5. Relative conformational energies (RE, kcal/mol) of 76 β-glucose conformers computed using DLPNO-CCSD(T)/CBS and the tested force fields

| Label | DLPNO-CCSD(T)/CBS | PBDPC25 | | CHARMM36 | | GLYCAM06 | | AMOEBA | |
|---|---|---|---|---|---|---|---|---|---|
| | | RE | Δ | RE | Δ | RE | Δ | RE | Δ |
| 81 | 0.25 | 0.50 | 0.25 | -0.79 | -1.04 | -0.99 | -1.24 | -1.88 | -2.13 |
| 82 | 0.02 | 0.68 | 0.66 | 1.09 | 1.07 | 0.31 | 0.29 | 0.19 | 0.17 |
| 83 | 0.00 | 0.00 | 0.00 | 0.00 | 0.00 | 0.00 | 0.00 | 0.00 | 0.00 |
| 84 | 4.17 | 4.73 | 0.56 | 10.45 | 6.28 | 6.32 | 2.15 | 4.32 | 0.15 |
| 85 | 0.82 | 0.26 | -0.56 | 0.49 | -0.33 | 1.17 | 0.35 | 1.94 | 1.12 |
| 86 | 4.09 | 6.14 | 2.05 | 8.77 | 4.68 | 3.49 | -0.6 | 5.82 | 1.73 |
| 87 | 4.31 | 4.66 | 0.35 | 9.50 | 5.19 | 4.78 | 0.47 | 5.02 | 0.71 |
| 88 | 1.20 | 1.09 | -0.11 | -0.10 | -1.30 | 0.39 | -0.81 | 0.10 | -1.10 |
| 89 | 1.15 | 1.20 | 0.05 | 1.64 | 0.49 | 1.95 | 0.8 | 2.35 | 1.20 |
| 90 | 4.58 | 6.93 | 2.35 | 9.19 | 4.61 | 4.01 | -0.57 | 7.87 | 3.29 |
| 91 | 5.33 | 5.07 | -0.26 | 9.80 | 4.47 | 6.34 | 1.01 | 5.39 | 0.06 |
| 92 | 4.42 | 7.41 | 2.99 | 9.61 | 5.19 | 3.98 | -0.44 | 7.14 | 2.72 |
| 93 | 4.90 | 5.71 | 0.81 | 10.86 | 5.96 | 6.42 | 1.52 | 5.35 | 0.45 |
| 94 | 5.34 | 5.17 | -0.17 | 10.57 | 5.23 | 3.16 | -2.18 | 0.94 | -4.40 |
| 95 | 5.03 | 7.30 | 2.27 | 9.89 | 4.86 | 3.75 | -1.28 | 8.83 | 3.80 |
| 96 | 1.99 | 2.47 | 0.48 | 2.50 | 0.51 | 2.60 | 0.61 | 0.60 | -1.39 |
| 97 | 4.24 | 5.79 | 1.55 | 5.93 | 1.69 | 4.02 | -0.22 | 8.24 | 4.00 |
| 98 | 2.48 | 2.67 | 0.19 | 2.19 | -0.29 | 2.95 | 0.47 | 1.74 | -0.74 |
| 99 | 2.41 | 2.08 | -0.33 | 1.73 | -0.68 | 3.35 | 0.94 | 0.56 | -1.85 |
| 100 | 2.97 | 3.11 | 0.14 | 3.77 | 0.80 | 5.87 | 2.9 | 3.89 | 0.92 |
| 101 | 3.08 | 2.53 | -0.55 | 3.89 | 0.81 | 6.31 | 3.23 | 3.24 | 0.16 |
| 102 | 2.99 | 1.84 | -1.15 | 2.26 | -0.73 | 4.31 | 1.32 | 0.22 | -2.77 |
| 103 | 2.52 | 0.62 | -1.90 | 2.61 | 0.09 | 2.40 | -0.12 | 0.24 | -2.28 |
| 104 | 2.42 | 0.30 | -2.12 | 2.97 | 0.55 | 1.64 | -0.78 | -1.63 | -4.05 |
| 105 | 5.56 | 6.68 | 1.12 | 7.88 | 2.32 | 6.19 | 0.63 | 9.05 | 3.49 |
| 106 | 6.33 | 4.96 | -1.37 | 10.35 | 4.02 | 2.56 | -3.77 | 5.22 | -1.11 |
| 107 | 5.86 | 6.63 | 0.77 | 8.27 | 2.41 | 7.72 | 1.86 | 10.48 | 4.62 |

| | | | | | | | | |
|---|---|---|---|---|---|---|---|---|
| 108 | 5.60 | 5.01 | -0.59 | 10.44 | 4.84 | 5.75 | 0.15 | 6.02 | 0.42 |
| 109 | 5.98 | 7.36 | 1.38 | 8.93 | 2.95 | 8.76 | 2.78 | 10.26 | 4.28 |
| 110 | 5.65 | 2.93 | -2.72 | 6.39 | 0.74 | 4.18 | -1.47 | 4.54 | -1.11 |
| 111 | 6.38 | 6.15 | -0.23 | 8.66 | 2.28 | 9.36 | 2.98 | 10.79 | 4.41 |
| 112 | 6.35 | 7.92 | 1.57 | 8.79 | 2.44 | 6.77 | 0.42 | 4.89 | -1.46 |
| 113 | 7.35 | 7.08 | -0.27 | 12.03 | 4.68 | 9.80 | 2.45 | 6.24 | -1.11 |
| 114 | 5.94 | 6.97 | 1.03 | 7.75 | 1.81 | 6.89 | 0.95 | 7.24 | 1.30 |
| 115 | 6.84 | 9.08 | 2.24 | 9.31 | 2.47 | 6.33 | -0.51 | 5.92 | -0.92 |
| 116 | 6.94 | 9.15 | 2.21 | 8.62 | 1.68 | 7.55 | 0.61 | 3.90 | -3.04 |
| 117 | 6.50 | 7.30 | 0.80 | 7.04 | 0.54 | 8.16 | 1.66 | 7.99 | 1.49 |
| 118 | 7.81 | 7.57 | -0.24 | 13.83 | 6.02 | 10.46 | 2.65 | 6.64 | -1.17 |
| 119 | 7.12 | 8.04 | 0.92 | 10.07 | 2.95 | 5.80 | -1.32 | 2.82 | -4.30 |
| 120 | 8.01 | 11.08 | 3.07 | 10.73 | 2.72 | 14.44 | 6.43 | 8.17 | 0.16 |
| 121 | 7.43 | 9.10 | 1.67 | 12.92 | 5.49 | 11.1 | 3.67 | 6.12 | -1.31 |
| 122 | 7.89 | 8.14 | 0.25 | 10.24 | 2.35 | 12.57 | 4.68 | 12.36 | 4.47 |
| 123 | 8.56 | 6.39 | -2.17 | 11.73 | 3.17 | 11.2 | 2.64 | 8.55 | -0.01 |
| 124 | 7.46 | 9.21 | 1.75 | 13.07 | 5.61 | 11.67 | 4.21 | 7.35 | -0.11 |
| 125 | 6.80 | 9.70 | 2.90 | 13.86 | 7.06 | 11.00 | 4.2 | 10.23 | 3.43 |
| 126 | 7.51 | 5.27 | -2.24 | 10.72 | 3.21 | 8.05 | 0.54 | 9.61 | 2.10 |
| 127 | 7.66 | 9.24 | 1.58 | 11.79 | 4.13 | 9.90 | 2.24 | 5.76 | -1.91 |
| 128 | 7.87 | 9.37 | 1.50 | 13.05 | 5.18 | 14.84 | 6.97 | 8.45 | 0.58 |
| 129 | 6.99 | 9.46 | 2.47 | 12.67 | 5.68 | 10.74 | 3.75 | 8.32 | 1.33 |
| 130 | 8.04 | 7.32 | -0.72 | 11.88 | 3.84 | 11.44 | 3.4 | 7.79 | -0.25 |
| 131 | 7.48 | 10.68 | 3.20 | 11.67 | 4.19 | 14.74 | 7.26 | 8.89 | 1.41 |
| 132 | 8.52 | 11.41 | 2.89 | 13.56 | 5.04 | 14.64 | 6.12 | 10.71 | 2.19 |
| 133 | 8.33 | 9.87 | 1.54 | 14.63 | 6.3 | 16.41 | 8.08 | 9.49 | 1.16 |
| 134 | 7.10 | 10.06 | 2.96 | 10.68 | 3.58 | 9.53 | 2.43 | 3.10 | -4.00 |
| 135 | 9.92 | 9.14 | -0.78 | 13.83 | 3.91 | 9.44 | -0.48 | 9.69 | -0.23 |
| 136 | 7.38 | 11.98 | 4.60 | 10.50 | 3.12 | 13.57 | 6.19 | 9.82 | 2.44 |
| 137 | 7.50 | 11.81 | 4.31 | 11.40 | 3.9 | 13.33 | 5.83 | 9.90 | 2.40 |
| 138 | 9.04 | 10.65 | 1.61 | 14.26 | 5.22 | 16.63 | 7.59 | 11.66 | 2.62 |
| 139 | 7.70 | 7.24 | -0.46 | 8.57 | 0.87 | 9.43 | 1.73 | 10.40 | 2.70 |
| 140 | 8.01 | 5.92 | -2.09 | 8.91 | 0.90 | 11.61 | 3.6 | 10.85 | 2.84 |
| 141 | 7.98 | 11.30 | 3.32 | 13.71 | 5.73 | 11.38 | 3.4 | 6.11 | -1.87 |
| 142 | 8.06 | 11.17 | 3.11 | 11.46 | 3.40 | 15.73 | 7.67 | 9.36 | 1.30 |
| 143 | 8.04 | 9.65 | 1.61 | 13.71 | 5.67 | 11.98 | 3.94 | 8.00 | -0.04 |
| 144 | 8.83 | 9.78 | 0.95 | 13.27 | 4.44 | 16.65 | 7.82 | 14.13 | 5.30 |
| 145 | 8.25 | 6.03 | -2.22 | 10.23 | 1.98 | 11.76 | 3.51 | 10.73 | 2.48 |
| 146 | 9.42 | 10.23 | 0.81 | 13.56 | 4.14 | 11.43 | 2.01 | 7.62 | -1.80 |
| 147 | 10.05 | 12.97 | 2.92 | 14.00 | 3.95 | 17.18 | 7.13 | 9.82 | -0.23 |
| 148 | 9.89 | 11.62 | 1.73 | 15.23 | 5.34 | 12.83 | 2.94 | 7.43 | -2.46 |
| 149 | 10.46 | 10.97 | 0.51 | 17.47 | 7.01 | 17.06 | 6.6 | 10.98 | 0.52 |
| 150 | 10.34 | 9.32 | -1.02 | 14.57 | 4.23 | 14.11 | 3.77 | 6.83 | -3.51 |
| 151 | 9.67 | 12.73 | 3.06 | 13.69 | 4.02 | 14.09 | 4.42 | 10.92 | 1.25 |
| 152 | 10.12 | 8.64 | -1.48 | 12.04 | 1.92 | 11.77 | 1.65 | 7.18 | -2.94 |

| | | | | | | | | | |
|---|---|---|---|---|---|---|---|---|---|
| 153 | 10.31 | 14.19 | 3.88 | 12.24 | 1.93 | 12.82 | 2.51 | 6.79 | -3.52 |
| 154 | 11.86 | 10.99 | -0.87 | 18.92 | 7.06 | 15.41 | 3.55 | 8.55 | -3.31 |
| 155 | 10.71 | 14.87 | 4.16 | 13.62 | 2.91 | 14.63 | 3.92 | 9.03 | -1.68 |
| 156 | 11.45 | 13.70 | 2.25 | 16.78 | 5.33 | 15.73 | 4.28 | 10.26 | -1.19 |
| RMSE(0) | | | 1.94 | | 3.90 | | 3.58 | | 2.39 |

Table S6. Relative conformational energies (RE, kcal/mol) of 223 α-maltoses conformers computed using DLPNO-CCSD(T)/CBS and the tested force fields

| Label | DLPNO-CCSD(T)/CBS | PBDPC25 | | CHARMM36 | | GLYCAM06 | | AMOEBA | |
|---|---|---|---|---|---|---|---|---|---|
| | | RE | Δ | RE | Δ | RE | Δ | RE | Δ |
| 1 | -0.40 | -0.31 | 0.09 | -2.57 | -2.17 | 0.25 | 0.65 | 2.47 | 2.87 |
| 2 | 0.00 | 0.00 | 0.00 | 0.00 | 0.00 | 0.00 | 0.00 | 0.00 | 0.00 |
| 3 | 0.01 | -1.22 | -1.23 | -2.46 | -2.47 | -1.17 | -1.18 | -0.15 | -0.16 |
| 4 | -0.07 | 0.80 | 0.87 | -3.47 | -3.40 | -1.27 | -1.20 | 4.37 | 4.44 |
| 5 | -0.23 | 1.47 | 1.70 | -2.23 | -2.00 | -2.92 | -2.69 | 3.01 | 3.24 |
| 6 | 1.76 | 0.67 | -1.09 | -1.85 | -3.61 | -2.08 | -3.84 | 3.92 | 2.16 |
| 7 | 0.94 | 1.18 | 0.24 | 0.84 | -0.10 | 1.79 | 0.85 | 2.39 | 1.45 |
| 8 | 2.51 | 1.66 | -0.85 | -0.06 | -2.57 | 0.47 | -2.04 | 1.11 | -1.40 |
| 9 | 2.45 | 3.40 | 0.95 | -0.94 | -3.39 | 4.04 | 1.59 | 2.12 | -0.33 |
| 10 | 3.47 | 3.78 | 0.31 | 4.09 | 0.62 | 4.47 | 1.00 | 4.41 | 0.94 |
| 11 | 1.99 | 2.13 | 0.14 | 1.01 | -0.98 | 2.15 | 0.16 | 2.95 | 0.96 |
| 12 | 3.42 | 2.56 | -0.86 | 2.48 | -0.94 | 4.32 | 0.90 | 3.88 | 0.46 |
| 13 | 1.85 | 4.58 | 2.73 | 2.02 | 0.17 | 5.52 | 3.67 | 5.43 | 3.58 |
| 14 | 3.89 | 5.02 | 1.13 | 1.99 | -1.90 | 3.36 | -0.53 | 4.99 | 1.10 |
| 15 | 5.80 | 7.06 | 1.26 | 8.95 | 3.15 | 10.27 | 4.47 | -1.28 | -7.08 |
| 16 | 5.71 | 7.35 | 1.64 | 8.12 | 2.41 | 3.91 | -1.80 | 5.25 | -0.46 |
| 17 | 4.97 | 7.04 | 2.07 | 12.12 | 7.15 | 12.06 | 7.09 | 7.30 | 2.33 |
| 18 | 4.16 | 5.28 | 1.12 | 4.33 | 0.17 | -0.04 | -4.20 | 0.02 | -4.14 |
| 19 | 5.18 | 5.55 | 0.37 | 2.31 | -2.87 | 5.59 | 0.41 | 7.09 | 1.91 |
| 20 | 6.89 | 10.93 | 4.04 | 14.26 | 7.37 | 16.46 | 9.57 | 4.80 | -2.09 |
| 21 | 4.18 | -0.56 | -4.74 | 3.31 | -0.87 | 4.75 | 0.57 | -1.65 | -5.83 |
| 22 | 5.03 | 9.14 | 4.11 | 8.85 | 3.82 | 9.65 | 4.62 | 7.36 | 2.33 |
| 23 | 4.32 | 4.96 | 0.64 | 3.85 | -0.47 | 5.91 | 1.59 | 7.86 | 3.54 |
| 24 | 5.86 | 5.57 | -0.29 | 3.73 | -2.13 | 2.71 | -3.15 | 2.60 | -3.26 |
| 25 | 7.04 | 11.59 | 4.55 | 12.53 | 5.49 | 9.08 | 2.04 | 9.52 | 2.48 |
| 26 | 5.31 | 9.55 | 4.24 | 11.57 | 6.26 | 14.24 | 8.93 | 8.77 | 3.46 |
| 27 | 7.15 | 6.82 | -0.33 | 7.71 | 0.56 | 3.52 | -3.63 | 9.31 | 2.16 |
| 28 | 7.06 | 9.91 | 2.85 | 10.48 | 3.42 | 9.72 | 2.66 | 11.92 | 4.86 |
| 29 | 7.60 | 12.18 | 4.58 | 8.72 | 1.12 | 10.27 | 2.67 | 9.34 | 1.74 |
| 30 | 6.81 | 9.48 | 2.67 | 12.18 | 5.37 | 11.81 | 5.00 | 6.70 | -0.11 |
| 31 | 5.29 | 4.01 | -1.28 | 3.78 | -1.51 | -2.11 | -7.40 | -0.22 | -5.51 |
| 32 | 4.58 | 4.42 | -0.16 | 4.45 | -0.13 | 2.43 | -2.15 | 0.11 | -4.47 |
| 33 | 6.30 | 9.81 | 3.51 | 9.84 | 3.54 | 5.26 | -1.04 | -0.68 | -6.98 |
| 34 | 9.62 | 11.40 | 1.78 | 16.26 | 6.64 | 17.54 | 7.92 | 7.63 | -1.99 |
| 35 | 5.28 | 9.92 | 4.64 | 13.22 | 7.94 | 11.40 | 6.12 | 6.35 | 1.07 |

| | | | | | | | | | |
|---|---|---|---|---|---|---|---|---|---|
| 36 | 8.37 | 9.94 | 1.57 | 13.89 | 5.52 | 9.48 | 1.11 | 11.48 | 3.11 |
| 37 | 6.47 | 6.01 | -0.46 | 5.39 | -1.08 | 6.64 | 0.17 | 1.06 | -5.41 |
| 38 | 7.55 | 9.57 | 2.02 | 11.87 | 4.32 | 7.17 | -0.38 | 6.61 | -0.94 |
| 39 | 7.10 | 10.03 | 2.93 | 4.91 | -2.19 | 7.15 | 0.05 | 6.63 | -0.47 |
| 40 | 5.65 | 6.59 | 0.94 | 5.59 | -0.06 | 8.85 | 3.20 | 4.59 | -1.06 |
| 41 | 7.65 | 13.35 | 5.70 | 8.31 | 0.66 | 9.57 | 1.92 | 13.05 | 5.40 |
| 42 | 5.87 | 5.68 | -0.19 | 4.42 | -1.45 | 7.99 | 2.12 | 5.93 | 0.06 |
| 43 | 7.80 | 8.72 | 0.92 | 14.73 | 6.93 | 10.43 | 2.63 | 8.79 | 0.99 |
| 44 | 8.34 | 9.13 | 0.79 | 13.94 | 5.60 | 15.19 | 6.85 | 9.65 | 1.31 |
| 45 | 6.59 | 9.00 | 2.41 | 5.61 | -0.98 | 8.15 | 1.56 | 5.50 | -1.09 |
| 46 | 7.58 | 7.55 | -0.03 | 14.06 | 6.48 | 14.34 | 6.76 | 8.30 | 0.72 |
| 47 | 7.91 | 5.31 | -2.60 | 6.87 | -1.04 | 3.27 | -4.64 | 5.04 | -2.87 |
| 48 | 7.14 | 10.73 | 3.59 | 13.43 | 6.29 | 15.91 | 8.77 | 10.72 | 3.58 |
| 49 | 7.12 | 7.84 | 0.72 | 6.19 | -0.93 | 9.28 | 2.16 | 10.64 | 3.52 |
| 50 | 9.22 | 10.91 | 1.69 | 11.36 | 2.14 | 11.58 | 2.36 | 11.94 | 2.72 |
| 51 | 8.72 | 9.27 | 0.55 | 11.44 | 2.72 | 11.74 | 3.02 | 14.30 | 5.58 |
| 52 | 7.07 | 5.04 | -2.03 | 6.69 | -0.38 | 5.95 | -1.12 | 2.59 | -4.48 |
| 53 | 7.57 | 9.88 | 2.31 | 10.42 | 2.85 | 8.77 | 1.20 | 7.49 | -0.08 |
| 54 | 6.37 | 5.65 | -0.72 | 4.03 | -2.34 | 3.02 | -3.35 | -1.25 | -7.62 |
| 55 | 6.81 | 6.75 | -0.06 | 14.78 | 7.97 | 12.09 | 5.28 | 7.05 | 0.24 |
| 56 | 7.20 | 9.67 | 2.47 | 12.98 | 5.78 | 8.63 | 1.43 | 8.43 | 1.23 |
| 57 | 8.50 | 10.42 | 1.92 | 8.50 | 0.00 | 11.34 | 2.84 | 5.30 | -3.20 |
| 58 | 6.47 | 9.03 | 2.56 | 7.85 | 1.38 | 6.35 | -0.12 | 2.13 | -4.34 |
| 59 | 9.51 | 10.70 | 1.19 | 12.68 | 3.17 | 14.55 | 5.04 | 11.73 | 2.22 |
| 60 | 9.17 | 10.29 | 1.12 | 14.41 | 5.24 | 17.49 | 8.32 | 7.63 | -1.54 |
| 61 | 7.89 | 3.67 | -4.22 | 9.49 | 1.60 | 8.57 | 0.68 | 3.21 | -4.68 |
| 62 | 8.41 | 5.47 | -2.94 | 8.02 | -0.39 | 9.19 | 0.78 | 1.90 | -6.51 |
| 63 | 9.40 | 12.34 | 2.94 | 14.76 | 5.36 | 13.23 | 3.83 | 11.53 | 2.13 |
| 64 | 7.21 | 5.25 | -1.96 | 10.14 | 2.93 | 6.16 | -1.05 | 0.88 | -6.33 |
| 65 | 8.78 | 9.85 | 1.07 | 7.20 | -1.58 | 9.80 | 1.02 | 9.71 | 0.93 |
| 66 | 8.66 | 6.77 | -1.89 | 8.82 | 0.16 | 5.76 | -2.90 | 3.11 | -5.55 |
| 67 | 7.48 | 7.10 | -0.38 | 9.18 | 1.70 | 11.81 | 4.33 | 8.00 | 0.52 |
| 68 | 8.92 | 9.73 | 0.81 | 15.80 | 6.88 | 16.81 | 7.89 | 8.06 | -0.86 |
| 69 | 8.24 | 9.08 | 0.84 | 10.62 | 2.38 | 13.45 | 5.21 | 11.18 | 2.94 |
| 70 | 8.22 | 12.31 | 4.09 | 9.30 | 1.08 | 13.58 | 5.36 | 12.28 | 4.06 |
| 71 | 7.69 | 12.36 | 4.67 | 13.59 | 5.90 | 11.54 | 3.85 | 9.87 | 2.18 |
| 72 | 7.23 | 8.17 | 0.94 | 6.52 | -0.71 | 12.12 | 4.89 | 6.32 | -0.91 |
| 73 | 7.01 | 8.78 | 1.77 | 12.32 | 5.31 | 12.76 | 5.75 | 8.70 | 1.69 |
| 74 | 7.02 | 11.37 | 4.35 | 7.32 | 0.30 | 10.71 | 3.69 | 6.88 | -0.14 |
| 75 | 9.32 | 13.71 | 4.39 | 9.90 | 0.58 | 11.16 | 1.84 | 14.12 | 4.80 |
| 76 | 6.84 | 7.46 | 0.62 | 9.43 | 2.59 | 9.53 | 2.69 | 2.85 | -3.99 |
| 77 | 8.47 | 9.72 | 1.25 | 11.45 | 2.98 | 11.76 | 3.29 | 9.54 | 1.07 |
| 78 | 9.06 | 9.81 | 0.75 | 9.20 | 0.14 | 9.41 | 0.35 | 5.40 | -3.66 |
| 79 | 8.68 | 8.13 | -0.55 | 11.10 | 2.42 | 8.11 | -0.57 | 5.88 | -2.80 |
| 80 | 9.20 | 7.66 | -1.54 | 12.57 | 3.37 | 10.88 | 1.68 | 9.29 | 0.09 |

| | | | | | | | | | |
|---|---|---|---|---|---|---|---|---|---|
| 81 | 8.51 | 5.69 | -2.82 | 9.03 | 0.52 | 10.27 | 1.76 | 6.59 | -1.92 |
| 82 | 9.43 | 9.62 | 0.19 | 10.84 | 1.41 | 10.50 | 1.07 | 9.74 | 0.31 |
| 83 | 7.14 | 7.92 | 0.78 | 8.30 | 1.16 | 15.33 | 8.19 | 10.57 | 3.43 |
| 84 | 7.53 | 11.57 | 4.04 | 9.79 | 2.26 | 6.96 | -0.57 | 7.53 | 0.00 |
| 85 | 7.03 | 11.04 | 4.01 | 12.78 | 5.75 | 11.25 | 4.22 | 4.72 | -2.31 |
| 86 | 8.42 | 10.11 | 1.69 | 7.70 | -0.72 | 7.95 | -0.47 | 8.46 | 0.04 |
| 87 | 9.55 | 11.39 | 1.84 | 15.59 | 6.04 | 17.63 | 8.08 | 12.53 | 2.98 |
| 88 | 7.60 | 6.70 | -0.90 | 7.67 | 0.07 | 2.78 | -4.82 | -0.46 | -8.06 |
| 89 | 8.00 | 7.36 | -0.64 | 9.03 | 1.03 | 5.45 | -2.55 | -0.13 | -8.13 |
| 90 | 8.08 | 7.18 | -0.90 | 12.82 | 4.74 | 8.09 | 0.01 | 4.64 | -3.44 |
| 91 | 9.15 | 10.05 | 0.90 | 10.20 | 1.05 | 11.71 | 2.56 | 7.76 | -1.39 |
| 92 | 7.85 | 9.75 | 1.90 | 7.88 | 0.03 | 10.06 | 2.21 | 6.82 | -1.03 |
| 93 | 8.42 | 8.05 | -0.37 | 11.41 | 2.99 | 6.36 | -2.06 | 4.87 | -3.55 |
| 94 | 7.41 | 12.42 | 5.01 | 13.09 | 5.68 | 11.22 | 3.81 | 11.43 | 4.02 |
| 95 | 9.95 | 8.64 | -1.31 | 14.06 | 4.11 | 12.96 | 3.01 | 12.69 | 2.74 |
| 96 | 9.98 | 11.72 | 1.74 | 10.08 | 0.10 | 13.73 | 3.75 | 10.10 | 0.12 |
| 97 | 9.76 | 9.10 | -0.66 | 12.12 | 2.36 | 8.72 | -1.04 | 2.27 | -7.49 |
| 98 | 9.44 | 10.87 | 1.43 | 7.84 | -1.60 | 12.50 | 3.06 | 10.12 | 0.68 |
| 99 | 11.54 | 8.53 | -3.01 | 10.69 | -0.85 | 8.93 | -2.61 | 10.62 | -0.92 |
| 100 | 8.67 | 7.47 | -1.20 | 9.86 | 1.19 | 10.03 | 1.36 | 5.54 | -3.13 |
| 101 | 9.58 | 14.33 | 4.75 | 14.11 | 4.53 | 13.89 | 4.31 | 10.44 | 0.86 |
| 102 | 8.15 | 9.88 | 1.73 | 4.79 | -3.36 | 9.86 | 1.71 | 6.25 | -1.90 |
| 103 | 10.30 | 14.44 | 4.14 | 11.96 | 1.66 | 16.30 | 6.00 | 15.27 | 4.97 |
| 104 | 9.91 | 12.87 | 2.96 | 19.29 | 9.38 | 20.64 | 10.73 | 8.24 | -1.67 |
| 105 | 7.69 | 8.71 | 1.02 | 8.52 | 0.83 | 9.47 | 1.78 | 15.01 | 7.32 |
| 106 | 9.70 | 12.26 | 2.56 | 13.61 | 3.91 | 12.92 | 3.22 | 11.40 | 1.70 |
| 107 | 12.84 | 19.70 | 6.86 | 22.69 | 9.85 | 17.68 | 4.84 | 13.03 | 0.19 |
| 108 | 9.60 | 11.99 | 2.39 | 12.90 | 3.30 | 15.96 | 6.36 | 7.71 | -1.89 |
| 109 | 8.94 | 10.84 | 1.90 | 9.50 | 0.56 | 9.92 | 0.98 | 7.00 | -1.94 |
| 110 | 8.54 | 10.58 | 2.04 | 6.68 | -1.86 | 11.44 | 2.90 | 8.22 | -0.32 |
| 111 | 11.45 | 16.46 | 5.01 | 22.69 | 11.24 | 16.04 | 4.59 | 11.61 | 0.16 |
| 112 | 9.77 | 11.62 | 1.85 | 14.14 | 4.37 | 10.35 | 0.58 | 10.74 | 0.97 |
| 113 | 11.14 | 16.73 | 5.59 | 15.72 | 4.58 | 8.95 | -2.19 | 4.33 | -6.81 |
| 114 | 6.99 | 11.45 | 4.46 | 13.51 | 6.52 | 9.76 | 2.77 | 6.73 | -0.26 |
| 115 | 9.13 | 10.33 | 1.20 | 13.15 | 4.02 | 6.53 | -2.60 | 0.19 | -8.94 |
| 116 | 9.92 | 6.28 | -3.64 | 10.62 | 0.70 | 11.50 | 1.58 | 7.38 | -2.54 |
| 117 | 9.25 | 8.23 | -1.02 | 9.33 | 0.08 | 7.18 | -2.07 | 7.55 | -1.70 |
| 118 | 9.22 | 8.52 | -0.70 | 11.19 | 1.97 | 8.53 | -0.69 | 8.73 | -0.49 |
| 119 | 11.42 | 10.95 | -0.47 | 14.68 | 3.26 | 13.73 | 2.31 | 10.58 | -0.84 |
| 120 | 9.10 | 11.80 | 2.70 | 10.60 | 1.50 | 10.71 | 1.61 | 7.04 | -2.06 |
| 121 | 9.60 | 12.10 | 2.50 | 8.49 | -1.11 | 9.75 | 0.15 | 2.26 | -7.34 |
| 122 | 11.15 | 14.44 | 3.29 | 18.86 | 7.71 | 14.99 | 3.84 | 6.01 | -5.14 |
| 123 | 10.00 | 5.18 | -4.82 | 10.59 | 0.59 | 10.27 | 0.27 | 3.68 | -6.32 |
| 124 | 12.59 | 16.30 | 3.71 | 20.67 | 8.08 | 21.38 | 8.79 | 16.21 | 3.62 |
| 125 | 9.79 | 7.39 | -2.40 | 10.02 | 0.23 | 7.91 | -1.88 | 7.59 | -2.20 |

| | | | | | | | | | |
|---|---|---|---|---|---|---|---|---|---|
| 126 | 10.05 | 13.04 | 2.99 | 20.23 | 10.18 | 13.66 | 3.61 | 13.02 | 2.97 |
| 127 | 9.67 | 11.24 | 1.57 | 10.64 | 0.97 | 13.93 | 4.26 | 7.12 | -2.55 |
| 128 | 11.15 | 10.52 | -0.63 | 13.45 | 2.30 | 3.68 | -7.47 | 10.65 | -0.50 |
| 129 | 10.32 | 6.70 | -3.62 | 10.40 | 0.08 | 14.08 | 3.76 | 4.24 | -6.08 |
| 130 | 9.03 | 13.77 | 4.74 | 11.03 | 2.00 | 12.87 | 3.84 | 3.24 | -5.79 |
| 131 | 8.78 | 13.21 | 4.43 | 9.53 | 0.75 | 12.43 | 3.65 | 5.05 | -3.73 |
| 132 | 9.48 | 11.84 | 2.36 | 16.73 | 7.25 | 7.51 | -1.97 | 1.67 | -7.81 |
| 133 | 11.97 | 10.76 | -1.21 | 14.64 | 2.67 | 15.17 | 3.20 | 10.15 | -1.82 |
| 134 | 11.94 | 13.28 | 1.34 | 22.27 | 10.33 | 18.62 | 6.68 | 14.41 | 2.47 |
| 135 | 10.26 | 11.77 | 1.51 | 14.14 | 3.88 | 11.18 | 0.92 | 10.50 | 0.24 |
| 136 | 11.06 | 10.95 | -0.11 | 15.29 | 4.23 | 14.01 | 2.95 | 10.50 | -0.56 |
| 137 | 10.16 | 12.04 | 1.88 | 8.22 | -1.94 | 10.32 | 0.16 | 4.33 | -5.83 |
| 138 | 11.00 | 11.86 | 0.86 | 16.34 | 5.34 | 14.43 | 3.43 | 11.43 | 0.43 |
| 139 | 11.28 | 12.65 | 1.37 | 10.98 | -0.30 | 13.48 | 2.20 | 3.14 | -8.14 |
| 140 | 10.46 | 11.39 | 0.93 | 16.34 | 5.88 | 12.53 | 2.07 | 8.22 | -2.24 |
| 141 | 9.67 | 10.28 | 0.61 | 13.98 | 4.31 | 12.73 | 3.06 | 7.98 | -1.69 |
| 142 | 10.01 | 11.47 | 1.46 | 13.71 | 3.70 | 11.93 | 1.92 | 14.32 | 4.31 |
| 143 | 12.48 | 12.29 | -0.19 | 15.28 | 2.80 | 13.05 | 0.57 | 10.72 | -1.76 |
| 144 | 12.03 | 15.17 | 3.14 | 16.84 | 4.81 | 14.92 | 2.89 | 10.69 | -1.34 |
| 145 | 9.61 | 6.35 | -3.26 | 14.32 | 4.71 | 8.65 | -0.96 | 8.07 | -1.55 |
| 146 | 11.51 | 11.45 | -0.06 | 17.32 | 5.81 | 12.66 | 1.15 | 9.53 | -1.98 |
| 147 | 12.01 | 13.75 | 1.74 | 17.95 | 5.94 | 8.11 | -3.90 | 4.29 | -7.72 |
| 148 | 7.76 | 10.85 | 3.09 | 11.47 | 3.71 | 14.18 | 6.42 | 0.39 | -7.37 |
| 149 | 9.99 | 10.16 | 0.17 | 14.29 | 4.30 | 10.54 | 0.55 | 12.12 | 2.13 |
| 150 | 10.87 | 14.99 | 4.12 | 13.05 | 2.18 | 11.52 | 0.65 | 12.86 | 1.99 |
| 151 | 8.40 | 14.64 | 6.24 | 15.03 | 6.63 | 13.60 | 5.20 | 11.14 | 2.74 |
| 152 | 10.59 | 8.46 | -2.13 | 9.08 | -1.51 | 9.45 | -1.14 | 8.03 | -2.56 |
| 153 | 10.91 | 9.26 | -1.65 | 15.89 | 4.98 | 13.20 | 2.29 | 14.21 | 3.30 |
| 154 | 10.29 | 12.91 | 2.62 | 16.13 | 5.84 | 10.99 | 0.70 | 13.72 | 3.43 |
| 155 | 10.29 | 9.44 | -0.85 | 10.69 | 0.40 | 12.90 | 2.61 | 12.68 | 2.39 |
| 156 | 11.15 | 9.02 | -2.13 | 13.87 | 2.72 | 13.65 | 2.50 | 9.71 | -1.44 |
| 157 | 11.44 | 12.75 | 1.31 | 17.48 | 6.04 | 10.92 | -0.52 | 13.52 | 2.08 |
| 158 | 10.28 | 6.84 | -3.44 | 11.24 | 0.96 | 8.52 | -1.76 | 7.35 | -2.93 |
| 159 | 11.87 | 16.42 | 4.55 | 24.58 | 12.71 | 23.20 | 11.33 | 8.04 | -3.83 |
| 160 | 12.31 | 9.92 | -2.39 | 13.29 | 0.98 | 14.77 | 2.46 | 5.19 | -7.12 |
| 161 | 11.03 | 10.59 | -0.44 | 11.94 | 0.91 | 12.19 | 1.16 | 7.84 | -3.19 |
| 162 | 10.64 | 13.63 | 2.99 | 12.96 | 2.32 | 13.49 | 2.85 | 10.20 | -0.44 |
| 163 | 10.68 | 15.71 | 5.03 | 11.62 | 0.94 | 21.79 | 11.11 | 12.38 | 1.70 |
| 164 | 11.29 | 11.37 | 0.08 | 13.67 | 2.38 | 12.55 | 1.26 | 10.46 | -0.83 |
| 165 | 10.90 | 14.32 | 3.42 | 18.89 | 7.99 | 14.09 | 3.19 | 15.84 | 4.94 |
| 166 | 10.12 | 10.48 | 0.36 | 13.28 | 3.16 | 10.83 | 0.71 | 11.51 | 1.39 |
| 167 | 8.78 | 8.23 | -0.55 | 10.40 | 1.62 | 5.14 | -3.64 | 3.77 | -5.01 |
| 168 | 11.24 | 8.68 | -2.56 | 13.11 | 1.87 | 10.84 | -0.40 | 5.64 | -5.60 |
| 169 | 11.56 | 9.79 | -1.77 | 12.42 | 0.86 | 11.57 | 0.01 | 11.85 | 0.29 |
| 170 | 12.19 | 16.66 | 4.47 | 22.63 | 10.44 | 17.69 | 5.50 | 13.83 | 1.64 |

| | | | | | | | | | |
|---|---|---|---|---|---|---|---|---|---|
| 171 | 11.67 | 13.88 | 2.21 | 24.10 | 12.43 | 21.58 | 9.91 | 10.21 | -1.46 |
| 172 | 9.24 | 12.31 | 3.07 | 11.05 | 1.81 | 11.81 | 2.57 | 10.95 | 1.71 |
| 173 | 12.30 | 12.51 | 0.21 | 16.12 | 3.82 | 17.08 | 4.78 | 11.97 | -0.33 |
| 174 | 11.92 | 15.71 | 3.79 | 19.53 | 7.61 | 15.97 | 4.05 | 9.07 | -2.85 |
| 175 | 11.61 | 12.79 | 1.18 | 15.32 | 3.71 | 13.52 | 1.91 | 11.39 | -0.22 |
| 176 | 11.95 | 13.28 | 1.33 | 7.50 | -4.45 | 11.57 | -0.38 | 10.82 | -1.13 |
| 177 | 10.02 | 14.10 | 4.08 | 13.71 | 3.69 | 14.10 | 4.08 | 11.63 | 1.61 |
| 178 | 10.01 | 10.57 | 0.56 | 14.71 | 4.70 | 8.94 | -1.07 | -0.70 | -10.71 |
| 179 | 12.83 | 11.10 | -1.73 | 18.58 | 5.75 | 14.72 | 1.89 | 9.44 | -3.39 |
| 180 | 9.89 | 14.92 | 5.03 | 13.79 | 3.90 | 15.11 | 5.22 | 9.09 | -0.80 |
| 181 | 11.50 | 11.78 | 0.28 | 16.44 | 4.94 | 10.25 | -1.25 | -0.86 | -12.36 |
| 182 | 11.88 | 16.78 | 4.90 | 15.84 | 3.96 | 16.39 | 4.51 | 7.33 | -4.55 |
| 183 | 10.70 | 13.09 | 2.39 | 17.19 | 6.49 | 10.01 | -0.69 | 4.21 | -6.49 |
| 184 | 11.12 | 13.80 | 2.68 | 14.24 | 3.12 | 14.56 | 3.44 | 11.11 | -0.01 |
| 185 | 11.14 | 16.22 | 5.08 | 13.08 | 1.94 | 15.63 | 4.49 | 9.09 | -2.05 |
| 186 | 13.15 | 13.86 | 0.71 | 17.44 | 4.29 | 16.08 | 2.93 | 15.30 | 2.15 |
| 187 | 9.67 | 10.12 | 0.45 | 13.30 | 3.63 | 15.20 | 5.53 | 3.95 | -5.72 |
| 188 | 10.48 | 15.73 | 5.25 | 18.05 | 7.57 | 15.25 | 4.77 | 13.55 | 3.07 |
| 189 | 15.64 | 19.76 | 4.12 | 25.57 | 9.93 | 15.61 | -0.03 | 14.85 | -0.79 |
| 190 | 13.27 | 14.36 | 1.09 | 20.18 | 6.91 | 17.48 | 4.21 | 18.23 | 4.96 |
| 191 | 13.02 | 10.10 | -2.92 | 15.60 | 2.58 | 16.45 | 3.43 | 11.35 | -1.67 |
| 192 | 11.48 | 9.60 | -1.88 | 15.23 | 3.75 | 11.49 | 0.01 | 7.52 | -3.96 |
| 193 | 12.52 | 13.89 | 1.37 | 14.14 | 1.62 | 19.58 | 7.06 | 14.41 | 1.89 |
| 194 | 11.00 | 17.10 | 6.10 | 22.09 | 11.09 | 18.56 | 7.56 | 11.86 | 0.86 |
| 195 | 11.47 | 9.93 | -1.54 | 13.61 | 2.14 | 10.71 | -0.76 | 8.53 | -2.94 |
| 196 | 12.43 | 13.77 | 1.34 | 22.75 | 10.32 | 15.57 | 3.14 | 11.51 | -0.92 |
| 197 | 15.44 | 16.70 | 1.26 | 18.93 | 3.49 | 8.23 | -7.21 | 6.78 | -8.66 |
| 198 | 11.35 | 9.59 | -1.76 | 16.49 | 5.14 | 16.06 | 4.71 | 9.22 | -2.13 |
| 199 | 12.22 | 17.03 | 4.81 | 19.06 | 6.84 | 17.89 | 5.67 | 11.90 | -0.32 |
| 200 | 11.91 | 10.69 | -1.22 | 15.29 | 3.38 | 10.92 | -0.99 | 13.18 | 1.27 |
| 201 | 14.35 | 14.76 | 0.41 | 20.75 | 6.40 | 20.92 | 6.57 | 11.75 | -2.60 |
| 202 | 11.30 | 9.98 | -1.32 | 12.48 | 1.18 | 11.88 | 0.58 | 10.32 | -0.98 |
| 203 | 13.58 | 10.91 | -2.67 | 14.25 | 0.67 | 20.55 | 6.97 | 12.01 | -1.57 |
| 204 | 13.96 | 10.97 | -2.99 | 20.51 | 6.55 | 13.80 | -0.16 | 12.91 | -1.05 |
| 205 | 12.66 | 10.69 | -1.97 | 12.14 | -0.52 | 11.62 | -1.04 | 7.99 | -4.67 |
| 206 | 12.96 | 17.36 | 4.40 | 17.67 | 4.71 | 17.02 | 4.06 | 7.23 | -5.73 |
| 207 | 13.30 | 8.04 | -5.26 | 13.56 | 0.26 | 17.35 | 4.05 | 4.02 | -9.28 |
| 208 | 14.86 | 14.71 | -0.15 | 12.16 | -2.70 | 21.36 | 6.50 | 12.01 | -2.85 |
| 209 | 14.24 | 13.16 | -1.08 | 14.45 | 0.21 | 15.82 | 1.58 | 17.53 | 3.29 |
| 210 | 14.41 | 21.87 | 7.46 | 25.54 | 11.13 | 29.03 | 14.62 | 8.89 | -5.52 |
| 211 | 13.92 | 14.24 | 0.32 | 16.15 | 2.23 | 9.48 | -4.44 | 5.79 | -8.13 |
| 212 | 16.97 | 24.44 | 7.47 | 31.09 | 14.12 | 34.69 | 17.72 | 19.64 | 2.67 |
| 213 | 13.26 | 11.84 | -1.42 | 14.67 | 1.41 | 11.36 | -1.90 | 9.97 | -3.29 |
| 214 | 10.28 | 11.07 | 0.79 | 10.77 | 0.49 | 9.62 | -0.66 | 3.19 | -7.09 |
| 215 | 13.75 | 14.08 | 0.33 | 21.47 | 7.72 | 25.06 | 11.31 | 15.43 | 1.68 |

| | | | | | | | | | |
|---|---|---|---|---|---|---|---|---|---|
| 216 | 15.38 | 18.27 | 2.89 | 17.52 | 2.14 | 11.60 | -3.78 | 13.33 | -2.05 |
| 217 | 16.00 | 16.11 | 0.11 | 24.61 | 8.61 | 20.61 | 4.61 | 2.88 | -13.12 |
| 218 | 13.32 | 13.20 | -0.12 | 20.13 | 6.81 | 15.95 | 2.63 | 20.39 | 7.07 |
| 219 | 12.66 | 14.79 | 2.13 | 19.89 | 7.23 | 15.41 | 2.75 | 18.61 | 5.95 |
| 220 | 16.11 | 17.33 | 1.22 | 19.09 | 2.98 | 19.66 | 3.55 | 7.73 | -8.38 |
| 221 | 14.17 | 14.15 | -0.02 | 11.94 | -2.23 | 17.28 | 3.11 | 8.03 | -6.14 |
| 222 | 16.25 | 16.68 | 0.43 | 23.70 | 7.45 | 18.08 | 1.83 | 12.81 | -3.44 |
| 223 | 13.16 | 10.82 | -2.34 | 14.20 | 1.04 | 17.80 | 4.64 | 15.66 | 2.50 |
| RMSE(0) | | | 2.69 | | 4.60 | | 4.29 | | 3.94 |

Table S7. Relative conformational energies (RE, kcal/mol) of 90 xylose conformers computed using CCSD(T)/6-311+G(d,p) and the tested force fields

| Label | CCSD(T)/ | PBDPC25 | | CHARMM36 | | GLYCAM06 | | AMOEBA | |
|---|---|---|---|---|---|---|---|---|---|
| | 6-311+G(d,p) | RE | Δ | RE | Δ | RE | Δ | RE | Δ |
| 1 | 12.50 | 11.85 | -0.65 | 16.24 | 3.74 | 17.46 | 4.96 | 13.63 | 1.13 |
| 2 | 1.00 | 1.25 | 0.25 | 3.74 | 2.74 | 1.56 | 0.56 | 3.52 | 2.52 |
| 3 | 1.10 | 2.36 | 1.26 | 3.78 | 2.68 | 2.24 | 1.14 | 5.75 | 4.65 |
| 4 | 1.80 | 2.99 | 1.19 | 4.93 | 3.13 | 3.19 | 1.39 | 5.11 | 3.31 |
| 5 | 2.30 | 3.38 | 1.08 | 5.91 | 3.61 | 2.30 | 0.00 | 4.33 | 2.03 |
| 6 | 6.70 | 5.65 | -1.05 | 8.41 | 1.71 | 7.72 | 1.02 | 8.43 | 1.73 |
| 7 | 7.50 | 10.32 | 2.82 | 11.21 | 3.71 | 11.07 | 3.57 | 11.46 | 3.96 |
| 8 | 7.70 | 10.24 | 2.54 | 12.16 | 4.46 | 10.42 | 2.72 | 10.32 | 2.62 |
| 9 | 8.20 | 9.52 | 1.32 | 9.12 | 0.92 | 10.72 | 2.52 | 11.20 | 3.00 |
| 10 | 8.80 | 11.74 | 2.94 | 14.28 | 5.48 | 14.37 | 5.57 | 13.13 | 4.33 |
| 11 | 8.90 | 8.67 | -0.23 | 9.27 | 0.37 | 13.99 | 5.09 | 9.58 | 0.68 |
| 12 | 4.10 | 3.50 | -0.60 | 5.57 | 1.47 | 6.08 | 1.98 | 6.55 | 2.45 |
| 13 | 7.40 | 7.41 | 0.01 | 11.49 | 4.09 | 9.02 | 1.62 | 7.61 | 0.21 |
| 14 | 7.90 | 8.96 | 1.06 | 15.03 | 7.13 | 12.36 | 4.46 | 11.37 | 3.47 |
| 15 | 0.00 | 0.00 | 0.00 | 0.00 | 0.00 | 0.00 | 0.00 | 0.00 | 0.00 |
| 16 | 0.80 | 0.86 | 0.06 | 0.67 | -0.13 | 1.77 | 0.97 | 1.45 | 0.65 |
| 17 | 8.70 | 9.60 | 0.90 | 11.48 | 2.78 | 14.37 | 5.67 | 11.07 | 2.37 |
| 18 | 10.20 | 10.47 | 0.27 | 14.19 | 3.99 | 14.05 | 3.85 | 11.60 | 1.40 |
| 19 | 10.60 | 10.52 | -0.08 | 18.82 | 8.22 | 13.24 | 2.64 | 11.88 | 1.28 |
| 20 | 10.90 | 10.61 | -0.29 | 18.76 | 7.86 | 14.62 | 3.72 | 13.19 | 2.29 |
| 21 | 6.70 | 5.61 | -1.09 | 8.50 | 1.80 | 7.74 | 1.04 | 8.45 | 1.75 |
| 22 | 7.80 | 8.22 | 0.42 | 14.94 | 7.14 | 10.64 | 2.84 | 8.30 | 0.50 |
| 23 | 7.80 | 8.70 | 0.90 | 14.05 | 6.25 | 10.57 | 2.77 | 8.63 | 0.83 |
| 24 | 8.20 | 10.77 | 2.57 | 14.95 | 6.75 | 12.25 | 4.05 | 12.79 | 4.59 |
| 25 | 8.20 | 9.69 | 1.49 | 13.81 | 5.61 | 14.94 | 6.74 | 11.28 | 3.08 |
| 26 | 9.20 | 12.02 | 2.82 | 15.65 | 6.45 | 13.13 | 3.93 | 11.32 | 2.12 |
| 27 | 10.00 | 9.95 | -0.05 | 12.12 | 2.12 | 11.41 | 1.41 | 12.61 | 2.61 |
| 28 | 10.50 | 9.89 | -0.61 | 14.57 | 4.07 | 12.44 | 1.94 | 11.94 | 1.44 |
| 29 | 12.80 | 10.21 | -2.59 | 14.70 | 1.90 | 16.81 | 4.01 | 12.74 | -0.06 |
| 30 | 17.10 | 12.09 | -5.01 | 19.70 | 2.60 | 22.09 | 4.99 | 17.00 | -0.10 |
| 31 | 16.50 | 12.38 | -4.12 | 22.85 | 6.35 | 22.93 | 6.43 | 15.18 | -1.32 |

| | | | | | | | | | |
|---|---|---|---|---|---|---|---|---|---|
| 32 | 11.20 | 12.15 | 0.95 | 16.89 | 5.69 | 13.66 | 2.46 | 15.26 | 4.06 |
| 33 | 15.00 | 13.64 | -1.36 | 22.79 | 7.79 | 16.55 | 1.55 | 16.86 | 1.86 |
| 34 | 15.80 | 15.19 | -0.61 | 22.95 | 7.15 | 16.58 | 0.78 | 17.42 | 1.62 |
| 35 | 15.50 | 13.95 | -1.55 | 20.45 | 4.95 | 15.93 | 0.43 | 17.67 | 2.17 |
| 36 | 15.80 | 14.34 | -1.46 | 22.56 | 6.76 | 18.39 | 2.59 | 17.26 | 1.46 |
| 37 | 13.60 | 14.41 | 0.81 | 24.46 | 10.86 | 16.06 | 2.46 | 18.55 | 4.95 |
| 38 | 13.10 | 13.13 | 0.03 | 18.97 | 5.87 | 16.34 | 3.24 | 16.91 | 3.81 |
| 39 | 13.10 | 13.18 | 0.08 | 17.16 | 4.06 | 15.77 | 2.67 | 16.32 | 3.22 |
| 40 | 13.70 | 12.69 | -1.01 | 21.43 | 7.73 | 14.24 | 0.54 | 14.77 | 1.07 |
| 41 | 10.10 | 7.65 | -2.45 | 10.92 | 0.82 | 15.68 | 5.58 | 11.48 | 1.38 |
| 42 | 10.80 | 9.49 | -1.31 | 14.84 | 4.04 | 11.66 | 0.86 | 12.22 | 1.42 |
| 43 | 14.40 | 12.56 | -1.84 | 18.68 | 4.28 | 17.48 | 3.08 | 15.90 | 1.50 |
| 44 | 11.50 | 12.84 | 1.34 | 19.12 | 7.62 | 8.79 | -2.71 | 12.39 | 0.89 |
| 45 | 12.20 | 14.90 | 2.70 | 20.71 | 8.51 | 9.58 | -2.62 | 13.60 | 1.40 |
| 46 | 12.30 | 13.43 | 1.13 | 19.67 | 7.37 | 9.67 | -2.63 | 14.31 | 2.01 |
| 47 | 12.60 | 14.92 | 2.32 | 19.99 | 7.39 | 10.27 | -2.33 | 13.82 | 1.22 |
| 48 | 12.90 | 13.46 | 0.56 | 21.46 | 8.56 | 12.82 | -0.08 | 15.45 | 2.55 |
| 49 | 13.80 | 14.17 | 0.37 | 21.50 | 7.70 | 13.14 | -0.66 | 13.61 | -0.19 |
| 50 | 14.10 | 14.60 | 0.50 | 21.60 | 7.50 | 14.50 | 0.40 | 16.15 | 2.05 |
| 51 | 13.20 | 15.24 | 2.04 | 23.08 | 9.88 | 12.29 | -0.91 | 15.83 | 2.63 |
| 52 | 14.50 | 15.60 | 1.10 | 23.91 | 9.41 | 10.59 | -3.91 | 13.94 | -0.56 |
| 53 | 15.60 | 15.87 | 0.27 | 23.25 | 7.65 | 11.26 | -4.34 | 14.88 | -0.72 |
| 54 | 11.10 | 10.79 | -0.31 | 15.28 | 4.18 | 9.92 | -1.18 | 13.70 | 2.60 |
| 55 | 11.90 | 10.64 | -1.26 | 16.00 | 4.10 | 10.58 | -1.32 | 12.45 | 0.55 |
| 56 | 11.80 | 11.37 | -0.43 | 16.13 | 4.33 | 12.69 | 0.89 | 15.58 | 3.78 |
| 57 | 15.40 | 14.00 | -1.40 | 20.35 | 4.95 | 14.38 | -1.02 | 16.65 | 1.25 |
| 58 | 11.30 | 11.37 | 0.07 | 19.26 | 7.96 | 13.20 | 1.90 | 14.99 | 3.69 |
| 59 | 11.70 | 11.49 | -0.21 | 19.26 | 7.56 | 12.29 | 0.59 | 13.67 | 1.97 |
| 60 | 11.90 | 11.42 | -0.48 | 18.73 | 6.83 | 11.52 | -0.38 | 11.97 | 0.07 |
| 61 | 13.30 | 13.13 | -0.17 | 19.64 | 6.34 | 14.64 | 1.34 | 14.39 | 1.09 |
| 62 | 15.20 | 13.39 | -1.81 | 27.52 | 12.32 | 16.78 | 1.58 | 17.41 | 2.21 |
| 63 | 15.00 | 11.11 | -3.89 | 20.40 | 5.40 | 16.79 | 1.79 | 15.83 | 0.83 |
| 64 | 15.50 | 11.06 | -4.44 | 19.22 | 3.72 | 15.87 | 0.36 | 14.00 | -1.50 |
| 65 | 17.20 | 12.27 | -4.93 | 26.77 | 9.57 | 19.07 | 1.87 | 18.14 | 0.94 |
| 66 | 15.30 | 10.34 | -4.96 | 21.73 | 6.43 | 13.76 | -1.54 | 14.25 | -1.05 |
| 67 | 16.30 | 11.69 | -4.61 | 23.41 | 7.11 | 17.29 | 0.99 | 17.76 | 1.46 |
| 68 | 13.70 | 11.27 | -2.43 | 16.83 | 3.13 | 15.61 | 1.91 | 15.79 | 2.09 |
| 69 | 10.40 | 12.06 | 1.66 | 16.40 | 6.00 | 11.09 | 0.69 | 10.96 | 0.56 |
| 70 | 11.00 | 12.94 | 1.94 | 15.28 | 4.28 | 10.98 | -0.02 | 11.56 | 0.56 |
| 71 | 10.90 | 13.16 | 2.26 | 14.97 | 4.07 | 12.43 | 1.53 | 12.42 | 1.52 |
| 72 | 11.80 | 11.17 | -0.63 | 15.03 | 3.23 | 14.15 | 2.35 | 13.43 | 1.63 |
| 73 | 10.40 | 5.91 | -4.49 | 11.88 | 1.48 | 12.45 | 2.05 | 9.44 | -0.96 |
| 74 | 15.00 | 13.06 | -1.94 | 23.49 | 8.49 | 16.86 | 1.86 | 17.03 | 2.03 |
| 75 | 15.70 | 13.22 | -2.48 | 23.10 | 7.40 | 17.38 | 1.68 | 17.93 | 2.23 |
| 76 | 18.00 | 15.20 | -2.80 | 25.63 | 7.63 | 22.47 | 4.47 | 21.18 | 3.18 |

| | | | | | | | | | |
|---|---|---|---|---|---|---|---|---|---|
| 77 | 9.10 | 8.72 | -0.38 | 12.74 | 3.64 | 6.95 | -2.15 | 9.96 | 0.86 |
| 78 | 16.10 | 13.70 | -2.40 | 21.02 | 4.92 | 15.34 | -0.76 | 17.43 | 1.33 |
| 79 | 9.80 | 8.53 | -1.27 | 14.19 | 4.39 | 12.60 | 2.80 | 10.85 | 1.05 |
| 80 | 12.50 | 10.37 | -2.13 | 18.27 | 5.77 | 10.75 | -1.75 | 14.04 | 1.54 |
| 81 | 16.60 | 12.50 | -4.10 | 22.22 | 5.62 | 18.65 | 2.05 | 16.98 | 0.38 |
| 82 | 14.50 | 11.47 | -3.03 | 21.07 | 6.57 | 16.48 | 1.98 | 14.56 | 0.06 |
| 83 | 14.90 | 13.50 | -1.40 | 23.84 | 8.94 | 16.42 | 1.52 | 16.24 | 1.34 |
| 84 | 15.40 | 13.36 | -2.04 | 22.86 | 7.46 | 16.36 | 0.96 | 17.56 | 2.16 |
| 85 | 15.80 | 12.26 | -3.54 | 21.87 | 6.07 | 18.77 | 2.97 | 16.14 | 0.34 |
| 86 | 16.00 | 13.40 | -2.60 | 21.96 | 5.96 | 16.94 | 0.94 | 18.47 | 2.47 |
| 87 | 17.20 | 13.40 | -3.80 | 21.66 | 4.46 | 21.35 | 4.15 | 16.94 | -0.26 |
| 88 | 17.20 | 13.80 | -3.40 | 23.80 | 6.60 | 17.98 | 0.78 | 17.07 | -0.13 |
| 89 | 17.00 | 13.29 | -3.71 | 21.86 | 4.86 | 18.06 | 1.06 | 17.77 | 0.77 |
| 90 | 9.80 | 12.19 | 2.39 | 15.35 | 5.55 | 9.75 | -0.05 | 10.13 | 0.33 |
| RMSE(0) | | | 2.17 | | 6.00 | | 2.71 | | 2.11 |

Table S8. Relative conformational energies (RE, kcal/mol) of 168 mannose conformers computed using CCSD(T)/6-311+G(d,p) and the tested force fields

| Label | CCSD(T /6-311+G(d,p) | PBDPC25 | | CHARMM36 | | GLYCAM06 | | AMOEBA | |
|---|---|---|---|---|---|---|---|---|---|
| | | RE | Δ | RE | Δ | RE | Δ | RE | Δ |
| 1 | 10.00 | 12.53 | 2.53 | 15.08 | 5.08 | 13.84 | 3.84 | 2.60 | -7.40 |
| 2 | 11.10 | 13.40 | 2.30 | 16.84 | 5.74 | 17.03 | 5.93 | 7.79 | -3.31 |
| 3 | 2.80 | 4.80 | 2.00 | 4.42 | 1.62 | 2.61 | -0.19 | -0.87 | -3.67 |
| 4 | 3.00 | 6.07 | 3.07 | 4.24 | 1.24 | 4.31 | 1.31 | 4.14 | 1.14 |
| 5 | 3.30 | 4.51 | 1.21 | 5.85 | 2.55 | 3.83 | 0.53 | 4.03 | 0.73 |
| 6 | 3.50 | 5.22 | 1.72 | 5.82 | 2.32 | 4.44 | 0.94 | 5.52 | 2.02 |
| 7 | 4.00 | 6.48 | 2.48 | 6.11 | 2.11 | 3.79 | -0.21 | 5.35 | 1.35 |
| 8 | 3.60 | 4.64 | 1.04 | 6.75 | 3.15 | 3.20 | -0.40 | 0.99 | -2.61 |
| 9 | 3.80 | 4.30 | 0.50 | 7.23 | 3.43 | 3.08 | -0.72 | -0.30 | -4.10 |
| 10 | 4.80 | 7.59 | 2.79 | 6.66 | 1.86 | 5.17 | 0.37 | 7.46 | 2.66 |
| 11 | 9.90 | 8.13 | -1.77 | 12.78 | 2.88 | 6.99 | -2.91 | 7.23 | -2.67 |
| 12 | 10.60 | 8.27 | -2.33 | 15.43 | 4.83 | 7.86 | -2.74 | 9.11 | -1.49 |
| 13 | 3.10 | 8.50 | 5.40 | 7.17 | 4.07 | 8.42 | 5.32 | 5.71 | 2.61 |
| 14 | 3.50 | 7.34 | 3.84 | 7.36 | 3.86 | 8.65 | 5.15 | 2.80 | -0.70 |
| 15 | 3.70 | 6.26 | 2.56 | 6.41 | 2.71 | 5.70 | 2.00 | -0.71 | -4.41 |
| 16 | 4.20 | 7.74 | 3.54 | 6.40 | 2.20 | 8.24 | 4.04 | 0.40 | -3.80 |
| 17 | 4.50 | 7.89 | 3.39 | 7.29 | 2.79 | 10.30 | 5.80 | 3.97 | -0.53 |
| 18 | 5.50 | 10.65 | 5.15 | 5.65 | 0.15 | 9.89 | 4.39 | 7.01 | 1.51 |
| 19 | 5.90 | 11.71 | 5.81 | 5.59 | -0.31 | 11.67 | 5.77 | 6.67 | 0.77 |
| 20 | 6.10 | 9.69 | 3.59 | 5.16 | -0.94 | 10.25 | 4.15 | 7.45 | 1.35 |
| 21 | 7.10 | 12.26 | 5.16 | 7.48 | 0.38 | 11.79 | 4.69 | 8.07 | 0.97 |
| 22 | 5.80 | 11.03 | 5.23 | 4.71 | -1.09 | 10.27 | 4.47 | 5.89 | 0.09 |
| 23 | 7.40 | 11.89 | 4.49 | 9.88 | 2.48 | 10.97 | 3.57 | 6.11 | -1.29 |
| 24 | 8.20 | 6.85 | -1.35 | 10.04 | 1.84 | 7.30 | -0.90 | 7.89 | -0.31 |
| 25 | 9.00 | 7.94 | -1.06 | 11.73 | 2.73 | 7.71 | -1.29 | 7.12 | -1.88 |

| | | | | | | | | | |
|---|---|---|---|---|---|---|---|---|---|
| 26 | 9.40 | 9.74 | 0.34 | 14.60 | 5.20 | 9.87 | 0.47 | 5.83 | -3.57 |
| 27 | 4.70 | 6.58 | 1.88 | 9.68 | 4.98 | 12.27 | 7.57 | 5.91 | 1.21 |
| 28 | 5.70 | 7.62 | 1.92 | 9.99 | 4.29 | 13.09 | 7.39 | 8.02 | 2.32 |
| 29 | 6.90 | 7.94 | 1.04 | 12.89 | 5.99 | 8.99 | 2.09 | 1.98 | -4.92 |
| 30 | 7.50 | 8.40 | 0.90 | 13.07 | 5.57 | 9.79 | 2.29 | 3.40 | -4.10 |
| 31 | 7.80 | 7.71 | -0.09 | 11.85 | 4.05 | 10.43 | 2.63 | 4.36 | -3.44 |
| 32 | 0.00 | 0.00 | 0.00 | 0.00 | 0.00 | 0.00 | 0.00 | 0.00 | 0.00 |
| 33 | 0.60 | -1.05 | -1.65 | -0.16 | -0.76 | -2.02 | -2.62 | -2.72 | -3.32 |
| 34 | 0.70 | -1.87 | -2.57 | -1.52 | -2.22 | -1.92 | -2.62 | -2.66 | -3.36 |
| 35 | 0.70 | -1.18 | -1.88 | -0.06 | -0.76 | -1.32 | -2.02 | -2.02 | -2.72 |
| 36 | 0.60 | -0.85 | -1.45 | -1.89 | -2.49 | -2.70 | -3.30 | -4.59 | -5.19 |
| 37 | 10.60 | 7.74 | -2.86 | 12.00 | 1.40 | 10.31 | -0.29 | 5.84 | -4.76 |
| 38 | 6.30 | 7.09 | 0.79 | 10.01 | 3.71 | 10.84 | 4.54 | 5.86 | -0.44 |
| 39 | 6.80 | 10.40 | 3.60 | 8.59 | 1.79 | 8.02 | 1.22 | 3.58 | -3.22 |
| 40 | 7.20 | 10.32 | 3.12 | 7.32 | 0.12 | 9.74 | 2.54 | 2.66 | -4.54 |
| 41 | 6.70 | 10.03 | 3.33 | 7.93 | 1.23 | 7.57 | 0.87 | 0.48 | -6.22 |
| 42 | 5.30 | 5.24 | -0.06 | 5.48 | 0.18 | 4.53 | -0.77 | 2.98 | -2.32 |
| 43 | 7.10 | 5.35 | -1.75 | 9.95 | 2.85 | 6.82 | -0.28 | 8.71 | 1.61 |
| 44 | 6.90 | 6.16 | -0.74 | 10.30 | 3.40 | 8.43 | 1.53 | 10.88 | 3.98 |
| 45 | 6.80 | 8.80 | 2.00 | 9.91 | 3.11 | 7.85 | 1.05 | 8.87 | 2.07 |
| 46 | 6.70 | 6.29 | -0.41 | 9.42 | 2.72 | 8.76 | 2.06 | 9.31 | 2.61 |
| 47 | 5.50 | 7.07 | 1.57 | 5.03 | -0.47 | 5.29 | -0.21 | 5.95 | 0.45 |
| 48 | 12.00 | 9.34 | -2.66 | 15.22 | 3.22 | 11.22 | -0.78 | 5.84 | -6.16 |
| 49 | 6.70 | 9.67 | 2.97 | 13.30 | 6.60 | 16.76 | 10.06 | 9.51 | 2.81 |
| 50 | 7.10 | 9.50 | 2.40 | 12.26 | 5.16 | 16.65 | 9.55 | 10.34 | 3.24 |
| 51 | 8.00 | 11.22 | 3.22 | 11.95 | 3.95 | 14.68 | 6.68 | 6.84 | -1.16 |
| 52 | 7.10 | 10.60 | 3.50 | 11.50 | 4.40 | 11.05 | 3.95 | 9.26 | 2.16 |
| 53 | 7.00 | 10.26 | 3.26 | 8.45 | 1.45 | 9.14 | 2.14 | 4.74 | -2.26 |
| 54 | 7.30 | 10.07 | 2.77 | 8.97 | 1.67 | 8.95 | 1.65 | 4.49 | -2.81 |
| 55 | 7.90 | 10.49 | 2.59 | 9.80 | 1.90 | 10.48 | 2.58 | 8.13 | 0.23 |
| 56 | 8.20 | 11.23 | 3.03 | 12.26 | 4.06 | 12.83 | 4.63 | 10.65 | 2.45 |
| 57 | 8.10 | 10.51 | 2.41 | 10.16 | 2.06 | 10.68 | 2.58 | 5.25 | -2.85 |
| 58 | 8.50 | 10.89 | 2.39 | 11.63 | 3.13 | 12.63 | 4.13 | 10.06 | 1.56 |
| 59 | 8.80 | 11.31 | 2.51 | 9.71 | 0.91 | 12.30 | 3.50 | 8.71 | -0.09 |
| 60 | 11.30 | 13.65 | 2.35 | 12.19 | 0.89 | 16.50 | 5.20 | 10.21 | -1.09 |
| 61 | 10.30 | 11.07 | 0.77 | 10.44 | 0.14 | 14.25 | 3.95 | 9.45 | -0.85 |
| 62 | 13.10 | 11.97 | -1.13 | 20.45 | 7.35 | 10.69 | -2.41 | 7.42 | -5.68 |
| 63 | 14.20 | 14.37 | 0.17 | 22.16 | 7.96 | 14.58 | 0.38 | 13.90 | -0.30 |
| 64 | 14.80 | 16.01 | 1.21 | 21.79 | 6.99 | 14.44 | -0.36 | 11.25 | -3.55 |
| 65 | 14.10 | 16.85 | 2.75 | 21.81 | 7.71 | 13.69 | -0.41 | 13.69 | -0.41 |
| 66 | 10.20 | 13.77 | 3.57 | 14.90 | 4.70 | 10.16 | -0.04 | 11.50 | 1.30 |
| 67 | 10.50 | 11.71 | 1.21 | 15.89 | 5.39 | 12.25 | 1.75 | 13.35 | 2.85 |
| 68 | 10.20 | 10.29 | 0.09 | 16.99 | 6.79 | 7.17 | -3.03 | 6.49 | -3.71 |
| 69 | 10.60 | 10.11 | -0.49 | 16.07 | 5.47 | 9.25 | -1.35 | 7.44 | -3.16 |
| 70 | 11.30 | 13.04 | 1.74 | 14.94 | 3.64 | 11.33 | 0.03 | 10.08 | -1.22 |

| | | | | | | | | | | |
|---|---|---|---|---|---|---|---|---|---|---|
| 71 | 11.70 | 13.47 | 1.77 | 15.52 | 3.82 | 11.24 | -0.46 | 10.74 | -0.96 |
| 72 | 11.80 | 12.96 | 1.16 | 17.48 | 5.68 | 12.62 | 0.82 | 14.94 | 3.14 |
| 73 | 12.20 | 11.15 | -1.05 | 18.72 | 6.52 | 11.65 | -0.55 | 6.28 | -5.92 |
| 74 | 12.30 | 10.75 | -1.55 | 20.34 | 8.04 | 13.57 | 1.27 | 10.53 | -1.77 |
| 75 | 12.60 | 12.07 | -0.53 | 16.92 | 4.32 | 10.96 | -1.64 | 5.77 | -6.83 |
| 76 | 12.90 | 10.52 | -2.38 | 20.89 | 7.99 | 14.40 | 1.50 | 10.56 | -2.34 |
| 77 | 13.30 | 12.72 | -0.58 | 16.31 | 3.01 | 12.18 | -1.12 | 11.99 | -1.31 |
| 78 | 12.20 | 10.57 | -1.63 | 19.04 | 6.84 | 12.50 | 0.30 | 7.72 | -4.48 |
| 79 | 15.30 | 14.64 | -0.66 | 23.04 | 7.74 | 16.34 | 1.04 | 15.41 | 0.11 |
| 80 | 13.20 | 18.96 | 5.76 | 18.83 | 5.63 | 19.64 | 6.44 | 12.33 | -0.87 |
| 81 | 13.90 | 14.43 | 0.53 | 17.86 | 3.96 | 22.35 | 8.45 | 13.12 | -0.78 |
| 82 | 10.70 | 11.39 | 0.69 | 10.06 | -0.64 | 15.70 | 5.00 | 7.01 | -3.69 |
| 83 | 14.70 | 12.27 | -2.43 | 16.21 | 1.51 | 7.95 | -6.75 | 6.92 | -7.78 |
| 84 | 14.80 | 11.47 | -3.33 | 18.00 | 3.20 | 13.43 | -1.38 | 11.34 | -3.46 |
| 85 | 15.90 | 15.16 | -0.74 | 19.83 | 3.93 | 13.86 | -2.04 | 9.85 | -6.05 |
| 86 | 16.30 | 14.08 | -2.22 | 19.36 | 3.06 | 13.63 | -2.67 | 11.77 | -4.53 |
| 87 | 16.40 | 11.06 | -5.34 | 20.88 | 4.48 | 10.88 | -5.52 | 8.98 | -7.42 |
| 88 | 15.90 | 14.65 | -1.25 | 18.96 | 3.06 | 14.23 | -1.67 | 10.45 | -5.45 |
| 89 | 16.40 | 15.68 | -0.72 | 19.05 | 2.65 | 15.19 | -1.21 | 11.35 | -5.05 |
| 90 | 16.80 | 15.47 | -1.33 | 17.91 | 1.11 | 15.56 | -1.24 | 14.85 | -1.95 |
| 91 | 10.00 | 7.22 | -2.78 | 11.21 | 1.21 | 7.80 | -2.20 | 6.96 | -3.04 |
| 92 | 11.80 | 7.44 | -4.36 | 14.72 | 2.92 | 8.49 | -3.31 | 6.57 | -5.23 |
| 93 | 11.60 | 8.08 | -3.52 | 13.73 | 2.13 | 10.89 | -0.71 | 9.00 | -2.60 |
| 94 | 8.80 | 8.95 | 0.15 | 9.91 | 1.11 | 5.62 | -3.18 | 6.35 | -2.45 |
| 95 | 8.50 | 8.98 | 0.48 | 9.66 | 1.16 | 7.13 | -1.37 | 6.87 | -1.63 |
| 96 | 10.20 | 11.35 | 1.15 | 16.74 | 6.54 | 8.95 | -1.25 | 4.91 | -5.29 |
| 97 | 11.00 | 11.31 | 0.31 | 15.08 | 4.08 | 8.32 | -2.68 | 7.03 | -3.97 |
| 98 | 11.80 | 10.67 | -1.13 | 14.40 | 2.60 | 8.36 | -3.44 | 10.63 | -1.17 |
| 99 | 18.40 | 18.42 | 0.02 | 26.19 | 7.79 | 22.99 | 4.59 | 15.82 | -2.58 |
| 100 | 13.50 | 16.33 | 2.83 | 22.39 | 8.89 | 11.11 | -2.39 | 9.46 | -4.04 |
| 101 | 9.60 | 10.07 | 0.47 | 15.87 | 6.27 | 11.59 | 1.99 | 6.72 | -2.88 |
| 102 | 10.90 | 11.17 | 0.27 | 16.66 | 5.76 | 13.38 | 2.48 | 8.52 | -2.38 |
| 103 | 11.80 | 11.91 | 0.11 | 18.22 | 6.42 | 10.77 | -1.03 | 9.05 | -2.75 |
| 104 | 12.10 | 11.91 | -0.19 | 17.39 | 5.29 | 10.81 | -1.29 | 9.50 | -2.60 |
| 105 | 12.20 | 12.42 | 0.22 | 20.28 | 8.08 | 13.93 | 1.73 | 13.65 | 1.45 |
| 106 | 12.90 | 14.41 | 1.51 | 18.32 | 5.42 | 11.28 | -1.62 | 8.18 | -4.72 |
| 107 | 13.50 | 14.05 | 0.55 | 16.31 | 2.81 | 10.76 | -2.74 | 7.19 | -6.31 |
| 108 | 13.30 | 14.92 | 1.62 | 18.15 | 4.85 | 11.74 | -1.56 | 8.19 | -5.11 |
| 109 | 12.30 | 15.00 | 2.70 | 19.95 | 7.65 | 16.14 | 3.84 | 13.56 | 1.26 |
| 110 | 12.40 | 14.01 | 1.61 | 20.16 | 7.76 | 15.20 | 2.80 | 11.40 | -1.00 |
| 111 | 13.60 | 17.48 | 3.88 | 22.62 | 9.02 | 12.59 | -1.01 | 11.28 | -2.32 |
| 112 | 12.00 | 10.53 | -1.47 | 18.19 | 6.19 | 13.49 | 1.49 | 8.43 | -3.57 |
| 113 | 8.70 | 11.26 | 2.56 | 14.80 | 6.10 | 9.62 | 0.92 | 9.23 | 0.53 |
| 114 | 10.20 | 11.76 | 1.56 | 15.63 | 5.43 | 6.30 | -3.90 | 4.67 | -5.53 |
| 115 | 10.40 | 13.30 | 2.90 | 15.17 | 4.77 | 8.73 | -1.67 | 6.92 | -3.48 |

| | | | | | | | | | |
|---|---|---|---|---|---|---|---|---|---|
| 116 | 10.00 | 11.51 | 1.51 | 14.00 | 4.00 | 10.26 | 0.26 | 8.35 | -1.65 |
| 117 | 14.00 | 15.76 | 1.76 | 21.76 | 7.76 | 20.15 | 6.15 | 17.90 | 3.90 |
| 118 | 14.70 | 13.84 | -0.86 | 19.42 | 4.72 | 17.62 | 2.92 | 11.50 | -3.20 |
| 119 | 15.30 | 14.38 | -0.92 | 21.55 | 6.25 | 17.59 | 2.29 | 10.43 | -4.87 |
| 120 | 15.80 | 22.38 | 6.58 | 23.05 | 7.25 | 22.09 | 6.29 | 13.10 | -2.70 |
| 121 | 17.30 | 22.85 | 5.55 | 22.20 | 4.90 | 20.08 | 2.78 | 18.38 | 1.08 |
| 122 | 13.00 | 19.81 | 6.81 | 20.34 | 7.34 | 16.00 | 3.00 | 10.38 | -2.62 |
| 123 | 14.90 | 16.73 | 1.83 | 20.71 | 5.81 | 19.33 | 4.43 | 13.56 | -1.34 |
| 124 | 14.10 | 11.33 | -2.77 | 14.77 | 0.67 | 8.22 | -5.88 | 7.59 | -6.51 |
| 125 | 14.60 | 11.38 | -3.22 | 17.76 | 3.16 | 11.24 | -3.36 | 10.67 | -3.93 |
| 126 | 16.60 | 16.66 | 0.06 | 23.36 | 6.76 | 19.67 | 3.07 | 7.86 | -8.74 |
| 127 | 15.60 | 20.92 | 5.32 | 23.29 | 7.69 | 20.22 | 4.62 | 11.78 | -3.82 |
| 128 | 19.30 | 16.35 | -2.95 | 24.23 | 4.93 | 18.18 | -1.12 | 11.43 | -7.87 |
| 129 | 8.50 | 10.93 | 2.43 | 8.47 | -0.03 | 10.43 | 1.93 | 8.02 | -0.48 |
| 130 | 11.50 | 12.04 | 0.54 | 11.36 | -0.14 | 14.91 | 3.41 | 5.82 | -5.68 |
| 131 | 7.60 | 7.98 | 0.38 | 11.42 | 3.82 | 11.40 | 3.80 | 10.24 | 2.64 |
| 132 | 7.70 | 5.94 | -1.76 | 9.80 | 2.10 | 9.23 | 1.53 | 8.62 | 0.92 |
| 133 | 8.50 | 9.44 | 0.94 | 13.25 | 4.75 | 11.47 | 2.97 | 10.13 | 1.63 |
| 134 | 11.50 | 10.74 | -0.76 | 16.21 | 4.71 | 14.71 | 3.21 | 9.45 | -2.05 |
| 135 | 11.80 | 8.54 | -3.26 | 13.92 | 2.12 | 10.67 | -1.13 | 10.25 | -1.55 |
| 136 | 12.40 | 11.53 | -0.87 | 16.37 | 3.97 | 17.35 | 4.95 | 13.58 | 1.18 |
| 137 | 13.10 | 8.82 | -4.28 | 16.13 | 3.03 | 13.32 | 0.22 | 11.92 | -1.18 |
| 138 | 13.20 | 9.41 | -3.79 | 16.32 | 3.12 | 13.15 | -0.05 | 6.96 | -6.24 |
| 139 | 8.30 | 9.04 | 0.74 | 13.35 | 5.05 | 6.90 | -1.40 | 7.90 | -0.40 |
| 140 | 9.20 | 7.80 | -1.40 | 13.43 | 4.23 | 6.59 | -2.61 | 8.58 | -0.62 |
| 141 | 8.90 | 9.84 | 0.94 | 13.73 | 4.83 | 8.01 | -0.89 | 6.32 | -2.58 |
| 142 | 9.60 | 11.51 | 1.91 | 17.38 | 7.78 | 12.48 | 2.88 | 10.16 | 0.56 |
| 143 | 9.90 | 10.86 | 0.96 | 14.13 | 4.23 | 10.24 | 0.34 | 7.65 | -2.25 |
| 144 | 10.40 | 12.28 | 1.88 | 15.41 | 5.01 | 9.55 | -0.85 | 5.62 | -4.78 |
| 145 | 10.40 | 11.88 | 1.48 | 16.07 | 5.67 | 10.70 | 0.30 | 6.92 | -3.48 |
| 146 | 11.20 | 11.98 | 0.78 | 16.67 | 5.47 | 10.99 | -0.21 | 5.07 | -6.13 |
| 147 | 11.90 | 10.29 | -1.61 | 15.79 | 3.89 | 16.72 | 4.82 | 6.56 | -5.34 |
| 148 | 11.60 | 10.41 | -1.19 | 17.39 | 5.79 | 17.72 | 6.12 | 8.22 | -3.38 |
| 149 | 12.00 | 9.83 | -2.17 | 18.30 | 6.30 | 16.63 | 4.63 | 6.19 | -5.81 |
| 150 | 13.00 | 11.05 | -1.95 | 14.98 | 1.98 | 17.41 | 4.41 | 8.02 | -4.98 |
| 151 | 13.40 | 12.30 | -1.10 | 14.09 | 0.69 | 15.13 | 1.73 | 5.03 | -8.37 |
| 152 | 13.80 | 16.10 | 2.30 | 20.72 | 6.92 | 18.21 | 4.41 | 14.44 | 0.64 |
| 153 | 13.60 | 16.82 | 3.22 | 21.91 | 8.31 | 18.65 | 5.05 | 13.45 | -0.15 |
| 154 | 14.30 | 14.34 | 0.04 | 22.62 | 8.32 | 16.24 | 1.94 | 8.93 | -5.37 |
| 155 | 15.50 | 13.11 | -2.39 | 24.96 | 9.46 | 17.27 | 1.77 | 9.72 | -5.78 |
| 156 | 15.30 | 16.25 | 0.95 | 24.01 | 8.71 | 22.54 | 7.24 | 17.33 | 2.03 |
| 157 | 15.30 | 16.76 | 1.46 | 23.07 | 7.77 | 22.38 | 7.08 | 15.39 | 0.09 |
| 158 | 14.70 | 17.76 | 3.06 | 22.20 | 7.50 | 20.52 | 5.82 | 14.65 | -0.05 |
| 159 | 14.60 | 16.83 | 2.23 | 22.71 | 8.11 | 22.13 | 7.53 | 14.55 | -0.05 |
| 160 | 15.10 | 16.45 | 1.35 | 21.73 | 6.63 | 22.34 | 7.24 | 18.04 | 2.94 |

| | | | | | | | | | |
|---|---|---|---|---|---|---|---|---|---|
| 161 | 14.50 | 13.97 | -0.53 | 20.46 | 5.96 | 18.81 | 4.31 | 14.31 | -0.19 |
| 162 | 15.30 | 13.59 | -1.71 | 21.73 | 6.43 | 20.17 | 4.87 | 15.49 | 0.19 |
| 163 | 16.00 | 13.72 | -2.28 | 24.44 | 8.44 | 20.69 | 4.69 | 11.63 | -4.37 |
| 164 | 14.80 | 17.80 | 3.00 | 22.95 | 8.15 | 20.48 | 5.68 | 16.68 | 1.88 |
| 165 | 15.50 | 17.98 | 2.48 | 22.87 | 7.37 | 24.02 | 8.52 | 15.74 | 0.24 |
| 166 | 8.70 | 9.79 | 1.09 | 9.05 | 0.35 | 10.82 | 2.12 | 5.12 | -3.58 |
| 167 | 9.00 | 10.40 | 1.40 | 8.83 | -0.17 | 11.00 | 2.00 | 1.99 | -7.01 |
| 168 | 10.40 | 10.90 | 0.50 | 15.86 | 5.46 | 10.75 | 0.35 | 6.35 | -4.05 |
| RMSE(0) | | | 2.48 | | 4.89 | | 3.60 | | 3.54 |

Table S9. Relative conformational energies (RE, kcal/mol) of 9 α-allose conformers computed using CCSD(T)/CBS and the tested force fields

| Label | CCSD(T)/CBS | PBDPC25 | | CHARMM36 | | GLYCAM06 | | AMOEBA | |
|---|---|---|---|---|---|---|---|---|---|
| | | RE | Δ | RE | Δ | RE | Δ | RE | Δ |
| 1 | 0.00 | 0.00 | 0.00 | 0.00 | 0.00 | 0.00 | 0.00 | 0.00 | 0.00 |
| 4 | 0.59 | 2.13 | 1.54 | -0.07 | -0.66 | 0.30 | -0.29 | 1.92 | 1.33 |
| 5 | 0.50 | 3.01 | 2.51 | 1.17 | 0.67 | 1.10 | 0.60 | 2.17 | 1.67 |
| 7 | 0.53 | 0.36 | -0.17 | 0.47 | -0.06 | -0.55 | -1.08 | 0.92 | 0.39 |
| 8 | 1.68 | 1.16 | -0.52 | 0.81 | -0.87 | 2.06 | 0.38 | -0.65 | -2.33 |
| 9 | 1.89 | 1.20 | -0.69 | 4.29 | 2.40 | 2.40 | 0.51 | 4.17 | 2.28 |
| 10 | 2.54 | 4.40 | 1.86 | 1.72 | -0.82 | 2.98 | 0.44 | 2.12 | -0.42 |
| 12 | 2.75 | 1.27 | -1.48 | 2.52 | -0.23 | 1.77 | -0.98 | 0.08 | -2.67 |
| 15 | 5.06 | 1.97 | -3.09 | 4.28 | -0.78 | 2.33 | -2.73 | 3.22 | -1.84 |
| RMSE(0) | | | 1.76 | | 1.04 | | 1.15 | | 1.80 |

Table S10. Relative conformational (RE, kcal/mol) of 6 β-allose conformers computed using CCSD(T)/CBS and the tested force fields

| Label | CCSD(T)/CBS | PBDPC25 | | CHARMM36 | | GLYCAM06 | | AMOEBA | |
|---|---|---|---|---|---|---|---|---|---|
| | | RE | Δ | RE | Δ | RE | Δ | RE | Δ |
| 2 | 0.00 | 0.00 | 0.00 | 0.00 | 0.00 | 0.00 | 0.00 | 0.00 | 0.00 |
| 3 | 0.18 | -0.62 | -0.80 | -1.11 | -1.29 | -0.39 | -0.57 | -0.24 | -0.42 |
| 6 | 0.45 | -0.14 | -0.59 | -1.86 | -2.31 | -1.11 | -1.56 | -2.67 | -3.12 |
| 11 | 2.24 | 0.86 | -1.38 | 0.87 | -1.37 | 2.58 | 0.34 | -2.89 | -5.13 |
| 13 | 3.68 | 1.49 | -2.19 | 3.85 | 0.17 | 3.14 | -0.54 | 1.20 | -2.48 |
| 14 | 4.18 | 4.64 | 0.46 | 1.28 | -2.90 | 2.87 | -1.31 | 4.46 | 0.28 |
| RMSE(0) | | | 1.40 | | 2.08 | | 1.10 | | 3.25 |

Table S11. Dipole moments ($\mu_M$) in Debye of 80 α-glucose conformers computed by B3LYP/aug-cc-pVTZ and tested force fields

| Label | B3LYP /aug-cc-pVTZ | PBDPC25 | | CHARMM36 | | GLYCAM06 | | AMOEBA | |
|---|---|---|---|---|---|---|---|---|---|
| | | $\mu_M$ | Δ | $\mu_M$ | Δ | $\mu_M$ | Δ | $\mu_M$ | Δ |
| 1 | 2.90 | 2.97 | 0.07 | 3.85 | 0.95 | 3.52 | 0.62 | 3.12 | 0.22 |
| 2 | 3.65 | 3.67 | 0.02 | 5.11 | 1.46 | 5.13 | 1.48 | 3.94 | 0.30 |
| 3 | 2.80 | 2.94 | 0.14 | 3.91 | 1.11 | 3.84 | 1.03 | 2.96 | 0.16 |
| 4 | 1.34 | 1.55 | 0.22 | 1.35 | 0.02 | 1.80 | 0.46 | 1.44 | 0.10 |
| 5 | 1.22 | 1.00 | -0.22 | 0.92 | -0.30 | 1.66 | 0.44 | 1.12 | -0.10 |
| 6 | 2.68 | 2.39 | -0.29 | 3.33 | 0.65 | 3.25 | 0.56 | 2.59 | -0.09 |
| 7 | 2.58 | 2.68 | 0.10 | 3.44 | 0.86 | 3.66 | 1.08 | 2.91 | 0.33 |
| 8 | 2.16 | 2.34 | 0.18 | 2.89 | 0.73 | 3.41 | 1.26 | 2.49 | 0.33 |
| 9 | 2.61 | 2.89 | 0.28 | 3.21 | 0.60 | 3.46 | 0.85 | 2.95 | 0.35 |
| 10 | 2.03 | 2.05 | 0.02 | 2.49 | 0.46 | 3.05 | 1.02 | 2.30 | 0.27 |
| 11 | 3.06 | 3.19 | 0.13 | 4.19 | 1.14 | 4.06 | 1.00 | 3.26 | 0.20 |
| 12 | 0.97 | 0.97 | 0.00 | 1.43 | 0.46 | 1.15 | 0.18 | 1.04 | 0.07 |
| 13 | 2.93 | 3.41 | 0.47 | 4.18 | 1.25 | 4.38 | 1.44 | 3.14 | 0.20 |
| 14 | 2.49 | 2.35 | -0.14 | 3.54 | 1.05 | 3.56 | 1.08 | 2.85 | 0.36 |
| 15 | 3.52 | 3.70 | 0.18 | 4.77 | 1.25 | 4.36 | 0.84 | 4.00 | 0.48 |
| 16 | 2.14 | 2.21 | 0.07 | 2.24 | 0.10 | 2.45 | 0.30 | 2.46 | 0.31 |
| 17 | 3.78 | 3.36 | -0.42 | 4.32 | 0.54 | 4.76 | 0.98 | 3.86 | 0.08 |
| 18 | 5.09 | 4.56 | -0.53 | 6.26 | 1.17 | 6.28 | 1.19 | 4.95 | -0.15 |
| 19 | 2.31 | 1.69 | -0.63 | 2.32 | 0.00 | 2.53 | 0.22 | 2.37 | 0.06 |
| 20 | 2.32 | 2.29 | -0.03 | 2.90 | 0.58 | 2.83 | 0.52 | 2.67 | 0.35 |
| 21 | 1.97 | 1.56 | -0.41 | 2.28 | 0.31 | 2.74 | 0.77 | 1.78 | -0.18 |
| 22 | 1.27 | 0.99 | -0.28 | 2.03 | 0.76 | 1.46 | 0.19 | 1.06 | -0.22 |
| 23 | 2.02 | 2.06 | 0.05 | 2.68 | 0.67 | 3.07 | 1.06 | 2.52 | 0.50 |
| 24 | 4.69 | 4.32 | -0.37 | 5.92 | 1.23 | 6.26 | 1.57 | 4.52 | -0.17 |
| 25 | 3.64 | 3.24 | -0.40 | 4.07 | 0.44 | 4.49 | 0.85 | 3.85 | 0.22 |
| 26 | 2.35 | 2.31 | -0.04 | 2.33 | -0.02 | 2.87 | 0.52 | 2.61 | 0.26 |
| 27 | 3.94 | 3.76 | -0.17 | 5.17 | 1.23 | 4.78 | 0.84 | 4.34 | 0.40 |
| 28 | 4.85 | 4.71 | -0.14 | 6.47 | 1.62 | 6.42 | 1.57 | 4.91 | 0.06 |
| 29 | 4.43 | 4.36 | -0.07 | 5.87 | 1.44 | 5.43 | 1.00 | 4.81 | 0.38 |
| 30 | 2.30 | 2.64 | 0.34 | 3.55 | 1.25 | 3.86 | 1.56 | 2.93 | 0.63 |

| | | | | | | | | | | |
|---|---|---|---|---|---|---|---|---|---|---|
| 31 | 4.96 | 4.72 | -0.24 | 6.50 | 1.54 | 6.20 | 1.24 | 5.36 | 0.40 |
| 32 | 3.27 | 2.84 | -0.43 | 4.31 | 1.04 | 4.65 | 1.38 | 3.31 | 0.04 |
| 33 | 2.27 | 2.04 | -0.23 | 2.56 | 0.29 | 3.11 | 0.84 | 2.37 | 0.09 |
| 34 | 3.20 | 2.86 | -0.34 | 3.67 | 0.47 | 3.79 | 0.59 | 3.36 | 0.16 |
| 35 | 1.51 | 1.36 | -0.16 | 2.09 | 0.57 | 1.97 | 0.45 | 1.31 | -0.21 |
| 36 | 3.93 | 3.51 | -0.42 | 4.79 | 0.86 | 5.40 | 1.47 | 4.30 | 0.37 |
| 37 | 2.66 | 2.37 | -0.29 | 3.11 | 0.45 | 2.99 | 0.33 | 2.84 | 0.17 |
| 38 | 3.46 | 3.35 | -0.12 | 4.84 | 1.37 | 4.83 | 1.37 | 3.74 | 0.28 |
| 39 | 4.25 | 4.06 | -0.19 | 5.71 | 1.47 | 5.56 | 1.32 | 4.10 | -0.15 |
| 40 | 3.34 | 3.16 | -0.18 | 4.49 | 1.15 | 4.93 | 1.59 | 3.73 | 0.38 |
| 41 | 4.65 | 4.42 | -0.23 | 6.05 | 1.40 | 5.64 | 1.00 | 4.58 | -0.07 |
| 42 | 2.55 | 2.36 | -0.19 | 3.36 | 0.81 | 3.38 | 0.83 | 2.60 | 0.05 |
| 43 | 2.22 | 1.87 | -0.35 | 2.10 | -0.12 | 2.60 | 0.38 | 2.24 | 0.02 |
| 44 | 1.78 | 1.97 | 0.19 | 2.43 | 0.66 | 2.79 | 1.01 | 2.14 | 0.37 |
| 45 | 1.35 | 0.87 | -0.47 | 0.74 | -0.61 | 1.54 | 0.19 | 1.63 | 0.28 |
| 46 | 4.67 | 4.39 | -0.27 | 6.03 | 1.37 | 5.83 | 1.16 | 5.19 | 0.52 |
| 47 | 4.92 | 4.70 | -0.21 | 6.33 | 1.41 | 6.32 | 1.40 | 5.29 | 0.38 |
| 48 | 3.81 | 3.72 | -0.09 | 4.50 | 0.69 | 4.71 | 0.89 | 3.99 | 0.17 |
| 49 | 2.32 | 2.50 | 0.18 | 3.48 | 1.16 | 3.85 | 1.53 | 2.21 | -0.11 |
| 50 | 2.96 | 3.05 | 0.09 | 4.10 | 1.14 | 3.78 | 0.82 | 3.05 | 0.09 |
| 51 | 4.38 | 4.15 | -0.23 | 5.64 | 1.26 | 5.92 | 1.54 | 4.65 | 0.27 |
| 52 | 2.50 | 2.72 | 0.22 | 3.06 | 0.55 | 3.42 | 0.92 | 2.79 | 0.29 |
| 53 | 3.88 | 3.90 | 0.02 | 5.06 | 1.17 | 4.93 | 1.05 | 4.06 | 0.17 |
| 54 | 2.31 | 2.56 | 0.25 | 3.12 | 0.81 | 3.01 | 0.70 | 2.54 | 0.23 |
| 55 | 2.98 | 3.23 | 0.25 | 3.81 | 0.83 | 3.71 | 0.74 | 3.37 | 0.39 |
| 56 | 1.17 | 0.87 | -0.30 | 1.68 | 0.51 | 1.81 | 0.64 | 1.15 | -0.03 |
| 57 | 2.68 | 2.62 | -0.06 | 3.47 | 0.79 | 3.72 | 1.03 | 2.92 | 0.23 |
| 58 | 4.04 | 3.92 | -0.12 | 5.19 | 1.15 | 5.38 | 1.35 | 4.09 | 0.06 |
| 59 | 2.81 | 2.81 | 0.00 | 3.63 | 0.82 | 3.90 | 1.09 | 2.67 | -0.15 |
| 60 | 2.44 | 1.92 | -0.52 | 2.89 | 0.44 | 2.10 | -0.34 | 2.31 | -0.13 |
| 61 | 2.84 | 2.88 | 0.04 | 4.15 | 1.31 | 3.86 | 1.01 | 3.19 | 0.34 |
| 62 | 3.33 | 2.80 | -0.53 | 3.64 | 0.32 | 3.78 | 0.46 | 3.30 | -0.03 |
| 63 | 3.55 | 3.45 | -0.11 | 5.05 | 1.50 | 4.74 | 1.19 | 4.01 | 0.46 |
| 64 | 1.91 | 1.69 | -0.22 | 2.50 | 0.59 | 2.92 | 1.01 | 2.06 | 0.15 |
| 65 | 2.15 | 2.57 | 0.42 | 3.48 | 1.33 | 3.78 | 1.63 | 2.49 | 0.34 |
| 66 | 3.19 | 2.84 | -0.35 | 4.21 | 1.02 | 3.61 | 0.42 | 3.31 | 0.12 |
| 67 | 2.46 | 2.67 | 0.21 | 3.68 | 1.22 | 3.93 | 1.47 | 2.92 | 0.46 |
| 68 | 2.69 | 3.00 | 0.30 | 4.26 | 1.57 | 4.16 | 1.47 | 3.10 | 0.41 |
| 69 | 3.97 | 3.92 | -0.05 | 5.47 | 1.50 | 5.53 | 1.55 | 3.93 | -0.05 |
| 70 | 3.02 | 3.22 | 0.20 | 4.39 | 1.36 | 4.48 | 1.45 | 3.29 | 0.26 |
| 71 | 3.42 | 3.36 | -0.05 | 5.40 | 1.99 | 5.40 | 1.98 | 3.91 | 0.49 |
| 72 | 1.47 | 1.29 | -0.17 | 1.88 | 0.41 | 1.56 | 0.09 | 1.28 | -0.18 |
| 73 | 4.12 | 4.00 | -0.12 | 5.24 | 1.12 | 5.58 | 1.47 | 3.97 | -0.15 |
| 74 | 1.21 | 0.99 | -0.22 | 1.30 | 0.09 | 1.32 | 0.11 | 1.30 | 0.09 |
| 75 | 1.61 | 1.62 | 0.02 | 2.36 | 0.76 | 2.94 | 1.33 | 1.70 | 0.09 |

| | | | | | | | | | |
|---|---|---|---|---|---|---|---|---|---|
| 76 | 2.60 | 2.58 | -0.02 | 3.69 | 1.08 | 3.52 | 0.91 | 2.88 | 0.28 |
| 77 | 2.22 | 1.87 | -0.35 | 3.49 | 1.27 | 2.89 | 0.67 | 2.14 | -0.07 |
| 78 | 2.69 | 2.39 | -0.30 | 4.03 | 1.34 | 4.38 | 1.69 | 2.64 | -0.05 |
| 79 | 3.47 | 3.30 | -0.17 | 4.66 | 1.18 | 5.29 | 1.81 | 3.61 | 0.13 |
| 80 | 2.70 | 2.79 | 0.09 | 3.64 | 0.95 | 3.49 | 0.80 | 2.88 | 0.18 |
| RMSE | | | 0.27 | | 1.01 | | 1.08 | | 0.27 |

Table S12. Dipole moments ($\mu_M$) in Debye of 76 β-glucose conformers computed by B3LYP/aug-cc-pVTZ and tested force fields

| Label | B3LYP /aug-cc-pVTZ | PBDPC25 | | CHARMM36 | | GLYCAM06 | | AMOEBA | |
|---|---|---|---|---|---|---|---|---|---|
| | | $\mu_M$ | Δ | $\mu_M$ | Δ | $\mu_M$ | Δ | $\mu_M$ | Δ |
| 81 | 2.97 | 2.88 | -0.08 | 3.54 | 0.57 | 3.73 | 0.76 | 3.27 | 0.30 |
| 82 | 3.49 | 3.39 | -0.10 | 4.97 | 1.48 | 4.97 | 1.49 | 3.81 | 0.32 |
| 83 | 2.91 | 2.90 | -0.01 | 3.65 | 0.74 | 4.10 | 1.19 | 3.17 | 0.26 |
| 84 | 2.47 | 2.28 | -0.20 | 3.01 | 0.53 | 3.13 | 0.66 | 3.06 | 0.58 |
| 85 | 3.21 | 3.33 | 0.11 | 4.29 | 1.08 | 4.83 | 1.62 | 3.65 | 0.43 |
| 86 | 2.39 | 2.31 | -0.08 | 3.09 | 0.70 | 2.81 | 0.42 | 2.18 | -0.21 |
| 87 | 1.86 | 1.86 | 0.00 | 2.73 | 0.87 | 2.58 | 0.72 | 1.37 | -0.49 |
| 88 | 3.29 | 3.36 | 0.07 | 4.18 | 0.88 | 4.61 | 1.31 | 3.72 | 0.43 |
| 89 | 2.34 | 2.56 | 0.23 | 3.33 | 0.99 | 3.75 | 1.42 | 2.63 | 0.29 |
| 90 | 2.63 | 2.34 | -0.29 | 3.14 | 0.51 | 3.13 | 0.51 | 2.19 | -0.44 |
| 91 | 1.33 | 1.44 | 0.11 | 1.88 | 0.56 | 2.02 | 0.70 | 1.98 | 0.65 |
| 92 | 4.56 | 4.61 | 0.05 | 6.26 | 1.70 | 6.18 | 1.61 | 4.36 | -0.20 |
| 93 | 3.59 | 3.30 | -0.28 | 4.54 | 0.95 | 4.46 | 0.87 | 3.74 | 0.15 |
| 94 | 1.30 | 1.42 | 0.12 | 2.11 | 0.80 | 1.97 | 0.67 | 0.79 | -0.51 |
| 95 | 4.64 | 4.47 | -0.17 | 6.12 | 1.48 | 6.26 | 1.62 | 4.48 | -0.16 |
| 96 | 2.47 | 2.46 | 0.00 | 3.81 | 1.34 | 3.36 | 0.89 | 2.62 | 0.15 |
| 97 | 2.91 | 2.88 | -0.04 | 3.89 | 0.98 | 4.19 | 1.27 | 3.15 | 0.24 |
| 98 | 1.62 | 1.89 | 0.28 | 2.28 | 0.67 | 2.34 | 0.72 | 1.65 | 0.04 |
| 99 | 2.14 | 2.23 | 0.09 | 2.96 | 0.82 | 2.54 | 0.40 | 2.24 | 0.10 |
| 100 | 2.29 | 2.57 | 0.28 | 3.10 | 0.81 | 3.17 | 0.88 | 2.48 | 0.19 |
| 101 | 2.75 | 2.86 | 0.11 | 3.46 | 0.71 | 3.51 | 0.76 | 2.74 | -0.01 |
| 102 | 2.32 | 2.11 | -0.21 | 3.06 | 0.74 | 2.84 | 0.52 | 2.14 | -0.18 |
| 103 | 1.94 | 1.88 | -0.06 | 2.82 | 0.88 | 2.73 | 0.79 | 2.00 | 0.06 |
| 104 | 0.68 | 0.72 | 0.04 | 0.65 | -0.03 | 1.21 | 0.53 | 1.09 | 0.41 |
| 105 | 2.31 | 2.80 | 0.49 | 3.74 | 1.43 | 3.51 | 1.20 | 2.45 | 0.14 |
| 106 | 2.67 | 2.34 | -0.33 | 2.90 | 0.24 | 2.89 | 0.23 | 2.94 | 0.27 |
| 107 | 2.94 | 3.16 | 0.22 | 4.72 | 1.78 | 4.19 | 1.25 | 2.58 | -0.36 |
| 108 | 1.01 | 0.91 | -0.10 | 1.31 | 0.30 | 1.08 | 0.07 | 0.07 | -0.94 |
| 109 | 0.11 | 0.35 | 0.24 | 0.51 | 0.40 | 1.06 | 0.95 | 0.80 | 0.69 |
| 110 | 3.20 | 2.86 | -0.34 | 3.70 | 0.50 | 4.47 | 1.27 | 4.24 | 1.04 |
| 111 | 3.44 | 3.22 | -0.22 | 4.57 | 1.13 | 4.90 | 1.47 | 3.66 | 0.22 |
| 112 | 1.16 | 1.05 | -0.11 | 1.64 | 0.48 | 2.23 | 1.07 | 1.85 | 0.68 |
| 113 | 3.56 | 3.32 | -0.25 | 4.79 | 1.23 | 4.82 | 1.25 | 3.71 | 0.15 |
| 114 | 3.90 | 3.80 | -0.11 | 4.88 | 0.97 | 5.38 | 1.48 | 4.44 | 0.54 |

| | | | | | | | | | |
|---|---|---|---|---|---|---|---|---|---|
| 115 | 1.25 | 1.37 | 0.12 | 1.91 | 0.66 | 2.01 | 0.76 | 1.37 | 0.12 |
| 116 | 1.82 | 1.83 | 0.01 | 2.59 | 0.78 | 2.20 | 0.38 | 2.07 | 0.26 |
| 117 | 2.95 | 2.90 | -0.05 | 3.87 | 0.92 | 4.11 | 1.16 | 3.18 | 0.23 |
| 118 | 1.84 | 1.44 | -0.40 | 2.55 | 0.71 | 2.19 | 0.35 | 1.76 | -0.08 |
| 119 | 2.59 | 2.22 | -0.37 | 3.57 | 0.98 | 3.19 | 0.60 | 3.08 | 0.49 |
| 120 | 1.27 | 1.02 | -0.25 | 1.49 | 0.21 | 1.76 | 0.48 | 1.89 | 0.61 |
| 121 | 1.14 | 1.18 | 0.04 | 1.52 | 0.38 | 1.63 | 0.49 | 1.19 | 0.05 |
| 122 | 1.96 | 2.10 | 0.14 | 2.79 | 0.83 | 2.38 | 0.42 | 1.35 | -0.62 |
| 123 | 1.83 | 1.54 | -0.29 | 2.16 | 0.32 | 2.71 | 0.88 | 1.82 | -0.01 |
| 124 | 2.67 | 2.30 | -0.37 | 3.10 | 0.43 | 3.36 | 0.69 | 2.83 | 0.16 |
| 125 | 2.57 | 2.43 | -0.14 | 3.59 | 1.02 | 3.12 | 0.55 | 3.13 | 0.56 |
| 126 | 0.68 | 0.73 | 0.05 | 0.84 | 0.15 | 1.24 | 0.56 | 1.30 | 0.61 |
| 127 | 1.61 | 1.32 | -0.28 | 2.47 | 0.86 | 1.98 | 0.37 | 1.39 | -0.22 |
| 128 | 2.41 | 2.32 | -0.08 | 2.73 | 0.32 | 2.80 | 0.39 | 2.71 | 0.31 |
| 129 | 3.84 | 3.55 | -0.29 | 4.92 | 1.08 | 5.35 | 1.50 | 4.00 | 0.16 |
| 130 | 3.21 | 2.93 | -0.28 | 3.75 | 0.54 | 4.45 | 1.23 | 3.36 | 0.15 |
| 131 | 1.10 | 0.97 | -0.12 | 1.40 | 0.30 | 0.51 | -0.59 | 1.50 | 0.40 |
| 132 | 2.77 | 3.05 | 0.28 | 3.60 | 0.83 | 3.47 | 0.69 | 3.28 | 0.50 |
| 133 | 3.48 | 3.51 | 0.03 | 4.72 | 1.24 | 4.86 | 1.38 | 4.08 | 0.60 |
| 134 | 4.96 | 4.55 | -0.40 | 6.29 | 1.33 | 6.50 | 1.55 | 4.80 | -0.15 |
| 135 | 2.46 | 2.46 | 0.00 | 3.21 | 0.75 | 3.06 | 0.60 | 2.43 | -0.03 |
| 136 | 3.29 | 3.28 | -0.01 | 4.55 | 1.26 | 4.71 | 1.42 | 3.96 | 0.67 |
| 137 | 3.89 | 3.95 | 0.06 | 5.82 | 1.94 | 5.72 | 1.84 | 4.32 | 0.44 |
| 138 | 2.47 | 2.48 | 0.01 | 3.80 | 1.33 | 3.25 | 0.78 | 2.87 | 0.40 |
| 139 | 2.52 | 2.67 | 0.14 | 3.69 | 1.17 | 3.63 | 1.10 | 2.89 | 0.37 |
| 140 | 2.64 | 2.73 | 0.09 | 3.53 | 0.89 | 3.27 | 0.63 | 2.67 | 0.03 |
| 141 | 4.26 | 3.75 | -0.52 | 4.98 | 0.72 | 5.44 | 1.18 | 4.68 | 0.42 |
| 142 | 2.87 | 2.72 | -0.15 | 3.38 | 0.51 | 3.54 | 0.67 | 3.75 | 0.88 |
| 143 | 3.94 | 3.47 | -0.47 | 5.46 | 1.52 | 5.22 | 1.28 | 4.01 | 0.07 |
| 144 | 2.46 | 2.32 | -0.15 | 3.70 | 1.24 | 3.81 | 1.34 | 2.70 | 0.23 |
| 145 | 2.86 | 2.96 | 0.10 | 3.86 | 1.00 | 3.83 | 0.97 | 3.23 | 0.37 |
| 146 | 2.34 | 2.01 | -0.33 | 2.97 | 0.63 | 2.78 | 0.44 | 2.15 | -0.20 |
| 147 | 0.71 | 1.04 | 0.34 | 1.73 | 1.03 | 1.60 | 0.90 | 0.82 | 0.12 |
| 148 | 2.31 | 2.44 | 0.13 | 3.91 | 1.59 | 3.16 | 0.85 | 2.28 | -0.03 |
| 149 | 3.98 | 3.78 | -0.20 | 5.51 | 1.53 | 5.51 | 1.53 | 3.87 | -0.11 |
| 150 | 1.77 | 1.98 | 0.22 | 2.69 | 0.92 | 2.46 | 0.70 | 2.04 | 0.28 |
| 151 | 2.08 | 2.40 | 0.31 | 3.72 | 1.64 | 3.03 | 0.94 | 1.73 | -0.35 |
| 152 | 2.89 | 2.45 | -0.45 | 3.59 | 0.70 | 3.52 | 0.62 | 2.90 | 0.01 |
| 153 | 1.59 | 1.65 | 0.06 | 2.23 | 0.64 | 2.38 | 0.79 | 1.75 | 0.16 |
| 154 | 3.86 | 3.86 | 0.00 | 5.64 | 1.78 | 5.18 | 1.32 | 3.78 | -0.08 |
| 155 | 0.96 | 1.16 | 0.21 | 1.77 | 0.81 | 2.40 | 1.44 | 1.50 | 0.54 |
| 156 | 3.96 | 3.90 | -0.07 | 5.44 | 1.47 | 5.45 | 1.48 | 4.12 | 0.16 |
| RMSE | | | 0.21 | | 0.99 | | 1.01 | | 0.39 |

Table S13. Dipole moments ($\mu_M$) in Debye of 223 α-maltose conformers computed by B3LYP/aug-cc-pVTZ and tested force fields

| Label | B3LYP /aug-cc-pVTZ | PBDPC25 | | CHARMM36 | | GLYCAM06 | | AMOEBA | |
|---|---|---|---|---|---|---|---|---|---|
| | | $\mu_M$ | Δ | $\mu_M$ | Δ | $\mu_M$ | Δ | $\mu_M$ | Δ |
| 1 | 4.09 | 4.04 | -0.05 | 4.56 | 0.47 | 5.77 | 1.68 | 4.33 | 0.24 |
| 2 | 3.15 | 3.65 | 0.50 | 4.75 | 1.60 | 6.09 | 2.94 | 3.98 | 0.83 |
| 3 | 3.48 | 3.71 | 0.23 | 4.24 | 0.76 | 5.51 | 2.03 | 4.02 | 0.53 |
| 4 | 5.61 | 4.95 | -0.66 | 6.36 | 0.76 | 7.17 | 1.57 | 5.82 | 0.21 |
| 5 | 2.50 | 2.81 | 0.31 | 1.94 | -0.56 | 2.55 | 0.05 | 2.82 | 0.32 |
| 6 | 2.52 | 2.34 | -0.18 | 2.77 | 0.25 | 1.84 | -0.68 | 2.73 | 0.21 |
| 7 | 6.94 | 6.35 | -0.59 | 8.53 | 1.59 | 9.93 | 2.99 | 7.80 | 0.87 |
| 8 | 3.03 | 2.97 | -0.06 | 3.69 | 0.66 | 4.75 | 1.72 | 3.39 | 0.36 |
| 9 | 2.44 | 2.20 | -0.24 | 2.87 | 0.44 | 2.82 | 0.39 | 2.38 | -0.06 |
| 10 | 5.22 | 5.97 | 0.75 | 6.82 | 1.61 | 7.20 | 1.98 | 5.50 | 0.29 |
| 11 | 1.87 | 1.55 | -0.32 | 1.07 | -0.81 | 1.04 | -0.84 | 1.93 | 0.06 |
| 12 | 3.46 | 3.00 | -0.46 | 4.20 | 0.75 | 4.57 | 1.11 | 3.33 | -0.12 |
| 13 | 5.91 | 5.86 | -0.05 | 7.67 | 1.76 | 8.97 | 3.06 | 6.73 | 0.82 |
| 14 | 4.01 | 4.55 | 0.53 | 5.02 | 1.01 | 5.65 | 1.64 | 4.31 | 0.30 |
| 15 | 1.63 | 1.80 | 0.17 | 2.22 | 0.59 | 2.63 | 1.00 | 1.46 | -0.17 |
| 16 | 1.65 | 1.24 | -0.40 | 2.16 | 0.52 | 1.40 | -0.25 | 1.91 | 0.26 |
| 17 | 4.02 | 3.97 | -0.05 | 4.50 | 0.47 | 3.44 | -0.58 | 4.17 | 0.15 |
| 18 | 3.89 | 3.85 | -0.04 | 4.92 | 1.03 | 5.86 | 1.97 | 4.02 | 0.12 |
| 19 | 5.91 | 6.02 | 0.11 | 6.99 | 1.08 | 7.01 | 1.10 | 6.28 | 0.37 |
| 20 | 5.73 | 5.15 | -0.58 | 6.73 | 1.00 | 6.00 | 0.27 | 5.96 | 0.23 |
| 21 | 1.05 | 1.39 | 0.34 | 2.14 | 1.09 | 3.14 | 2.08 | 1.76 | 0.70 |
| 22 | 5.54 | 5.42 | -0.12 | 7.03 | 1.49 | 8.15 | 2.62 | 5.98 | 0.44 |
| 23 | 5.54 | 5.30 | -0.24 | 7.90 | 2.36 | 7.72 | 2.17 | 6.18 | 0.64 |
| 24 | 3.17 | 2.02 | -1.16 | 3.32 | 0.14 | 3.36 | 0.19 | 3.71 | 0.54 |
| 25 | 2.34 | 2.77 | 0.44 | 3.76 | 1.42 | 3.15 | 0.81 | 2.63 | 0.29 |
| 26 | 1.45 | 1.33 | -0.12 | 1.15 | -0.30 | 1.82 | 0.37 | 1.41 | -0.04 |
| 27 | 2.86 | 3.03 | 0.17 | 3.35 | 0.49 | 3.13 | 0.26 | 3.03 | 0.17 |
| 28 | 1.59 | 0.91 | -0.68 | 2.48 | 0.89 | 2.75 | 1.15 | 1.38 | -0.21 |
| 29 | 4.20 | 3.97 | -0.23 | 4.98 | 0.79 | 5.99 | 1.79 | 4.31 | 0.12 |
| 30 | 3.75 | 3.44 | -0.31 | 3.80 | 0.05 | 4.01 | 0.26 | 4.43 | 0.68 |
| 31 | 5.19 | 3.99 | -1.20 | 5.11 | -0.08 | 6.40 | 1.21 | 5.94 | 0.75 |
| 32 | 6.64 | 6.22 | -0.42 | 8.23 | 1.59 | 9.59 | 2.95 | 6.74 | 0.10 |
| 33 | 3.60 | 3.09 | -0.51 | 4.19 | 0.59 | 4.36 | 0.76 | 4.04 | 0.44 |
| 34 | 2.71 | 2.92 | 0.21 | 2.92 | 0.21 | 2.68 | -0.03 | 1.99 | -0.72 |
| 35 | 2.57 | 3.09 | 0.52 | 4.07 | 1.50 | 5.10 | 2.52 | 3.39 | 0.81 |
| 36 | 3.81 | 4.22 | 0.42 | 5.03 | 1.22 | 4.58 | 0.78 | 4.58 | 0.77 |
| 37 | 0.30 | 0.70 | 0.40 | 0.62 | 0.32 | 1.17 | 0.87 | 0.73 | 0.43 |
| 38 | 4.00 | 4.13 | 0.14 | 4.44 | 0.44 | 5.34 | 1.34 | 4.26 | 0.26 |
| 39 | 1.76 | 2.68 | 0.93 | 2.32 | 0.57 | 3.34 | 1.58 | 1.68 | -0.08 |
| 40 | 6.51 | 5.85 | -0.66 | 8.13 | 1.62 | 9.28 | 2.77 | 6.39 | -0.12 |
| 41 | 6.69 | 5.79 | -0.90 | 7.92 | 1.22 | 8.54 | 1.84 | 7.04 | 0.34 |
| 42 | 4.02 | 4.82 | 0.80 | 5.61 | 1.59 | 6.76 | 2.73 | 4.29 | 0.26 |
| 43 | 7.24 | 6.62 | -0.62 | 8.86 | 1.62 | 9.76 | 2.52 | 7.75 | 0.51 |

| | | | | | | | | | |
|---|---|---|---|---|---|---|---|---|---|
| 44 | 2.29 | 2.05 | -0.23 | 2.57 | 0.28 | 3.43 | 1.14 | 2.80 | 0.51 |
| 45 | 6.26 | 5.43 | -0.83 | 7.51 | 1.25 | 8.54 | 2.29 | 5.97 | -0.29 |
| 46 | 2.38 | 2.23 | -0.14 | 2.11 | -0.26 | 1.81 | -0.57 | 2.60 | 0.23 |
| 47 | 6.93 | 6.75 | -0.18 | 8.40 | 1.48 | 7.64 | 0.71 | 7.84 | 0.92 |
| 48 | 4.41 | 4.36 | -0.04 | 5.79 | 1.38 | 7.09 | 2.68 | 5.33 | 0.92 |
| 49 | 4.06 | 4.26 | 0.20 | 4.77 | 0.71 | 3.80 | -0.26 | 4.39 | 0.33 |
| 50 | 1.56 | 0.91 | -0.65 | 2.35 | 0.79 | 1.40 | -0.17 | 1.74 | 0.18 |
| 51 | 2.43 | 2.41 | -0.02 | 3.38 | 0.95 | 2.90 | 0.46 | 2.64 | 0.21 |
| 52 | 4.02 | 3.72 | -0.30 | 4.75 | 0.74 | 3.44 | -0.58 | 4.37 | 0.35 |
| 53 | 2.76 | 2.91 | 0.15 | 3.58 | 0.82 | 4.20 | 1.44 | 2.98 | 0.22 |
| 54 | 4.90 | 4.76 | -0.14 | 6.32 | 1.42 | 7.61 | 2.71 | 5.14 | 0.24 |
| 55 | 6.52 | 6.13 | -0.39 | 7.35 | 0.83 | 7.14 | 0.62 | 7.15 | 0.63 |
| 56 | 5.69 | 5.83 | 0.14 | 7.81 | 2.12 | 8.57 | 2.88 | 6.28 | 0.59 |
| 57 | 2.86 | 3.40 | 0.55 | 4.15 | 1.29 | 5.36 | 2.51 | 3.39 | 0.53 |
| 58 | 6.23 | 5.37 | -0.86 | 7.43 | 1.20 | 8.68 | 2.45 | 6.88 | 0.65 |
| 59 | 5.62 | 5.30 | -0.32 | 7.10 | 1.48 | 7.29 | 1.66 | 6.12 | 0.50 |
| 60 | 4.87 | 4.85 | -0.02 | 6.52 | 1.65 | 6.21 | 1.34 | 4.75 | -0.12 |
| 61 | 6.12 | 6.49 | 0.38 | 8.49 | 2.37 | 8.37 | 2.26 | 5.96 | -0.16 |
| 62 | 3.29 | 3.20 | -0.08 | 4.28 | 1.00 | 3.28 | -0.01 | 2.83 | -0.46 |
| 63 | 3.09 | 3.13 | 0.04 | 3.97 | 0.88 | 4.82 | 1.73 | 3.43 | 0.35 |
| 64 | 1.56 | 2.20 | 0.63 | 2.69 | 1.13 | 3.96 | 2.39 | 1.93 | 0.37 |
| 65 | 5.80 | 5.95 | 0.15 | 7.25 | 1.44 | 7.13 | 1.32 | 6.25 | 0.44 |
| 66 | 6.91 | 6.45 | -0.47 | 8.95 | 2.03 | 9.31 | 2.39 | 6.86 | -0.05 |
| 67 | 2.75 | 2.81 | 0.06 | 4.60 | 1.85 | 4.20 | 1.46 | 3.11 | 0.36 |
| 68 | 8.81 | 7.94 | -0.87 | 10.20 | 1.39 | 9.84 | 1.03 | 9.16 | 0.35 |
| 69 | 6.49 | 5.53 | -0.96 | 7.08 | 0.59 | 8.04 | 1.55 | 6.22 | -0.27 |
| 70 | 3.82 | 3.85 | 0.03 | 5.45 | 1.63 | 5.20 | 1.38 | 3.79 | -0.04 |
| 71 | 4.22 | 4.24 | 0.02 | 5.51 | 1.29 | 6.18 | 1.96 | 4.44 | 0.22 |
| 72 | 5.53 | 5.37 | -0.16 | 7.60 | 2.07 | 8.70 | 3.17 | 5.53 | 0.00 |
| 73 | 1.57 | 1.48 | -0.09 | 2.02 | 0.45 | 2.21 | 0.63 | 1.43 | -0.14 |
| 74 | 6.95 | 6.34 | -0.61 | 8.48 | 1.52 | 9.81 | 2.86 | 7.68 | 0.73 |
| 75 | 6.02 | 5.24 | -0.78 | 7.36 | 1.34 | 7.71 | 1.69 | 6.25 | 0.23 |
| 76 | 3.11 | 2.66 | -0.45 | 3.90 | 0.79 | 4.87 | 1.76 | 3.44 | 0.32 |
| 77 | 2.29 | 1.67 | -0.61 | 2.15 | -0.14 | 2.48 | 0.19 | 2.77 | 0.48 |
| 78 | 3.80 | 3.93 | 0.13 | 5.02 | 1.22 | 5.62 | 1.82 | 4.05 | 0.25 |
| 79 | 5.46 | 5.79 | 0.33 | 7.92 | 2.46 | 7.22 | 1.76 | 5.34 | -0.12 |
| 80 | 7.33 | 6.36 | -0.97 | 8.17 | 0.84 | 9.08 | 1.75 | 7.50 | 0.16 |
| 81 | 3.62 | 3.31 | -0.31 | 4.54 | 0.92 | 5.15 | 1.53 | 3.99 | 0.37 |
| 82 | 4.84 | 5.15 | 0.31 | 6.52 | 1.68 | 5.44 | 0.60 | 5.01 | 0.17 |
| 83 | 2.11 | 2.34 | 0.22 | 3.14 | 1.03 | 3.38 | 1.26 | 2.32 | 0.20 |
| 84 | 1.56 | 1.27 | -0.30 | 2.16 | 0.60 | 2.15 | 0.58 | 1.83 | 0.27 |
| 85 | 6.55 | 6.38 | -0.18 | 7.81 | 1.26 | 8.58 | 2.03 | 7.05 | 0.49 |
| 86 | 3.39 | 3.14 | -0.25 | 3.84 | 0.44 | 3.70 | 0.30 | 3.66 | 0.27 |
| 87 | 4.32 | 3.70 | -0.61 | 3.83 | -0.49 | 3.79 | -0.53 | 4.19 | -0.13 |
| 88 | 2.95 | 2.96 | 0.01 | 4.07 | 1.12 | 5.06 | 2.11 | 3.22 | 0.27 |

| | | | | | | | | | |
|---|---|---|---|---|---|---|---|---|---|
| 89 | 4.63 | 4.65 | 0.02 | 6.50 | 1.87 | 6.39 | 1.77 | 4.76 | 0.13 |
| 90 | 3.58 | 3.80 | 0.23 | 4.85 | 1.27 | 6.09 | 2.51 | 3.82 | 0.25 |
| 91 | 5.25 | 5.45 | 0.20 | 6.86 | 1.61 | 5.65 | 0.40 | 5.70 | 0.45 |
| 92 | 1.78 | 2.51 | 0.74 | 2.98 | 1.21 | 3.63 | 1.86 | 2.36 | 0.58 |
| 93 | 3.01 | 3.44 | 0.43 | 4.70 | 1.68 | 3.70 | 0.68 | 3.50 | 0.48 |
| 94 | 5.27 | 5.22 | -0.04 | 6.76 | 1.50 | 7.75 | 2.49 | 5.70 | 0.44 |
| 95 | 3.69 | 3.39 | -0.30 | 5.38 | 1.69 | 3.72 | 0.03 | 3.87 | 0.18 |
| 96 | 2.90 | 3.82 | 0.91 | 4.47 | 1.57 | 4.40 | 1.50 | 3.27 | 0.37 |
| 97 | 1.70 | 0.83 | -0.87 | 2.02 | 0.31 | 2.37 | 0.67 | 2.45 | 0.74 |
| 98 | 4.75 | 4.74 | -0.01 | 6.07 | 1.33 | 5.17 | 0.42 | 5.44 | 0.69 |
| 99 | 4.57 | 4.19 | -0.38 | 6.03 | 1.46 | 6.65 | 2.08 | 5.35 | 0.78 |
| 100 | 6.20 | 5.65 | -0.55 | 7.29 | 1.10 | 6.16 | -0.04 | 6.33 | 0.13 |
| 101 | 1.06 | 0.91 | -0.16 | 1.62 | 0.56 | 1.89 | 0.83 | 1.16 | 0.09 |
| 102 | 5.05 | 4.35 | -0.70 | 5.95 | 0.90 | 7.28 | 2.22 | 5.09 | 0.04 |
| 103 | 1.65 | 0.91 | -0.74 | 2.39 | 0.75 | 2.99 | 1.34 | 1.41 | -0.24 |
| 104 | 3.99 | 3.92 | -0.07 | 4.57 | 0.58 | 4.52 | 0.53 | 4.24 | 0.24 |
| 105 | 4.77 | 4.12 | -0.65 | 4.49 | -0.28 | 5.05 | 0.28 | 5.11 | 0.35 |
| 106 | 2.48 | 1.74 | -0.75 | 2.70 | 0.22 | 3.48 | 1.00 | 1.72 | -0.77 |
| 107 | 6.83 | 6.13 | -0.69 | 8.23 | 1.40 | 8.42 | 1.59 | 8.10 | 1.27 |
| 108 | 3.74 | 4.05 | 0.31 | 4.62 | 0.88 | 5.41 | 1.67 | 4.26 | 0.52 |
| 109 | 5.36 | 5.13 | -0.23 | 6.56 | 1.20 | 7.66 | 2.30 | 5.95 | 0.59 |
| 110 | 5.17 | 4.93 | -0.24 | 6.51 | 1.34 | 7.18 | 2.01 | 5.00 | -0.17 |
| 111 | 6.72 | 6.60 | -0.12 | 8.91 | 2.19 | 9.14 | 2.42 | 7.58 | 0.87 |
| 112 | 5.35 | 5.00 | -0.35 | 6.14 | 0.79 | 6.64 | 1.29 | 5.35 | 0.00 |
| 113 | 5.52 | 4.67 | -0.84 | 5.82 | 0.30 | 7.18 | 1.66 | 5.87 | 0.35 |
| 114 | 4.29 | 4.69 | 0.39 | 6.81 | 2.52 | 6.71 | 2.42 | 4.58 | 0.29 |
| 115 | 4.13 | 4.56 | 0.42 | 5.42 | 1.29 | 6.43 | 2.29 | 4.32 | 0.19 |
| 116 | 1.76 | 1.56 | -0.19 | 1.06 | -0.70 | 2.73 | 0.97 | 1.94 | 0.18 |
| 117 | 2.71 | 1.87 | -0.84 | 3.61 | 0.90 | 3.48 | 0.77 | 3.11 | 0.41 |
| 118 | 1.87 | 2.03 | 0.16 | 2.62 | 0.75 | 3.63 | 1.75 | 1.74 | -0.13 |
| 119 | 2.74 | 2.86 | 0.12 | 3.31 | 0.57 | 4.32 | 1.57 | 2.49 | -0.25 |
| 120 | 5.55 | 6.09 | 0.55 | 7.67 | 2.12 | 7.70 | 2.15 | 5.85 | 0.31 |
| 121 | 3.78 | 3.91 | 0.12 | 5.25 | 1.47 | 6.14 | 2.36 | 4.16 | 0.38 |
| 122 | 3.43 | 2.58 | -0.85 | 3.58 | 0.15 | 3.44 | 0.01 | 3.87 | 0.44 |
| 123 | 2.04 | 1.34 | -0.69 | 3.61 | 1.58 | 3.00 | 0.97 | 2.47 | 0.44 |
| 124 | 4.63 | 4.56 | -0.07 | 5.84 | 1.21 | 5.56 | 0.93 | 4.45 | -0.17 |
| 125 | 3.01 | 2.22 | -0.79 | 3.83 | 0.82 | 3.87 | 0.86 | 3.42 | 0.41 |
| 126 | 0.49 | 0.52 | 0.03 | 0.58 | 0.09 | 0.26 | -0.23 | 0.72 | 0.23 |
| 127 | 4.90 | 4.13 | -0.77 | 5.72 | 0.81 | 5.13 | 0.23 | 5.03 | 0.13 |
| 128 | 10.40 | 9.03 | -1.37 | 11.14 | 0.74 | 11.04 | 0.64 | 11.20 | 0.80 |
| 129 | 1.14 | 1.03 | -0.11 | 0.69 | -0.45 | 1.19 | 0.05 | 0.92 | -0.22 |
| 130 | 3.16 | 3.75 | 0.59 | 4.82 | 1.66 | 4.71 | 1.55 | 3.06 | -0.10 |
| 131 | 5.46 | 5.59 | 0.13 | 7.43 | 1.98 | 8.14 | 2.68 | 5.93 | 0.48 |
| 132 | 4.93 | 4.75 | -0.18 | 7.13 | 2.21 | 7.04 | 2.11 | 5.48 | 0.55 |
| 133 | 5.07 | 5.13 | 0.06 | 6.49 | 1.42 | 7.18 | 2.11 | 5.34 | 0.27 |

| | | | | | | | | | |
|---|---|---|---|---|---|---|---|---|---|
| 134 | 6.02 | 5.63 | -0.40 | 6.48 | 0.45 | 5.72 | -0.30 | 6.13 | 0.10 |
| 135 | 2.64 | 2.80 | 0.15 | 2.82 | 0.18 | 3.07 | 0.43 | 3.39 | 0.75 |
| 136 | 3.23 | 3.37 | 0.14 | 4.35 | 1.11 | 5.26 | 2.03 | 3.47 | 0.23 |
| 137 | 5.52 | 5.57 | 0.05 | 7.50 | 1.98 | 7.92 | 2.41 | 5.55 | 0.03 |
| 138 | 4.68 | 4.55 | -0.13 | 5.99 | 1.32 | 6.31 | 1.63 | 5.13 | 0.45 |
| 139 | 3.52 | 4.14 | 0.63 | 5.36 | 1.84 | 5.98 | 2.46 | 3.74 | 0.22 |
| 140 | 2.57 | 2.34 | -0.23 | 3.03 | 0.46 | 3.80 | 1.23 | 2.84 | 0.26 |
| 141 | 2.70 | 2.37 | -0.33 | 2.60 | -0.09 | 3.43 | 0.74 | 2.86 | 0.16 |
| 142 | 5.30 | 4.66 | -0.64 | 6.84 | 1.54 | 7.12 | 1.82 | 5.30 | 0.01 |
| 143 | 6.14 | 5.92 | -0.23 | 8.72 | 2.58 | 8.57 | 2.42 | 6.66 | 0.51 |
| 144 | 3.78 | 3.57 | -0.21 | 4.53 | 0.74 | 4.29 | 0.51 | 3.87 | 0.09 |
| 145 | 4.88 | 4.89 | 0.01 | 6.07 | 1.20 | 5.50 | 0.62 | 5.34 | 0.47 |
| 146 | 4.17 | 4.40 | 0.23 | 5.14 | 0.97 | 6.02 | 1.84 | 4.47 | 0.30 |
| 147 | 3.50 | 3.68 | 0.18 | 4.51 | 1.00 | 3.65 | 0.14 | 3.77 | 0.26 |
| 148 | 2.95 | 3.78 | 0.83 | 4.79 | 1.84 | 4.56 | 1.61 | 2.61 | -0.34 |
| 149 | 5.69 | 4.93 | -0.75 | 6.21 | 0.52 | 7.19 | 1.50 | 5.76 | 0.07 |
| 150 | 5.34 | 4.64 | -0.71 | 5.79 | 0.45 | 6.56 | 1.22 | 6.12 | 0.77 |
| 151 | 2.06 | 2.58 | 0.52 | 3.37 | 1.31 | 4.42 | 2.36 | 2.59 | 0.53 |
| 152 | 5.53 | 4.88 | -0.64 | 6.64 | 1.12 | 7.92 | 2.39 | 5.65 | 0.12 |
| 153 | 3.09 | 3.04 | -0.05 | 4.33 | 1.24 | 3.29 | 0.20 | 3.41 | 0.32 |
| 154 | 2.46 | 2.55 | 0.09 | 2.57 | 0.11 | 3.22 | 0.77 | 1.77 | -0.68 |
| 155 | 3.40 | 3.02 | -0.39 | 4.27 | 0.87 | 3.68 | 0.28 | 4.25 | 0.85 |
| 156 | 3.78 | 3.61 | -0.18 | 4.46 | 0.67 | 5.54 | 1.76 | 3.95 | 0.16 |
| 157 | 4.08 | 4.58 | 0.50 | 4.81 | 0.73 | 5.50 | 1.42 | 4.46 | 0.38 |
| 158 | 4.30 | 3.34 | -0.96 | 5.34 | 1.04 | 5.05 | 0.75 | 4.70 | 0.40 |
| 159 | 3.32 | 2.98 | -0.35 | 3.66 | 0.34 | 4.20 | 0.88 | 3.16 | -0.16 |
| 160 | 3.85 | 3.05 | -0.80 | 4.18 | 0.33 | 3.78 | -0.07 | 3.93 | 0.08 |
| 161 | 1.09 | 1.00 | -0.09 | 1.05 | -0.04 | 1.61 | 0.52 | 1.97 | 0.88 |
| 162 | 5.40 | 5.28 | -0.12 | 6.76 | 1.36 | 7.82 | 2.41 | 5.95 | 0.54 |
| 163 | 2.77 | 2.93 | 0.16 | 3.74 | 0.97 | 4.45 | 1.68 | 3.02 | 0.24 |
| 164 | 4.01 | 4.54 | 0.52 | 5.88 | 1.86 | 4.70 | 0.69 | 4.80 | 0.79 |
| 165 | 4.25 | 4.70 | 0.44 | 6.21 | 1.95 | 7.04 | 2.79 | 4.99 | 0.73 |
| 166 | 3.67 | 3.19 | -0.48 | 4.55 | 0.88 | 4.75 | 1.08 | 4.18 | 0.51 |
| 167 | 4.40 | 3.31 | -1.09 | 5.18 | 0.78 | 6.09 | 1.69 | 4.91 | 0.50 |
| 168 | 2.49 | 1.88 | -0.61 | 2.59 | 0.10 | 2.83 | 0.34 | 2.49 | -0.01 |
| 169 | 5.96 | 5.13 | -0.82 | 6.44 | 0.48 | 7.40 | 1.44 | 6.28 | 0.32 |
| 170 | 6.30 | 5.96 | -0.34 | 8.39 | 2.09 | 8.88 | 2.57 | 6.56 | 0.26 |
| 171 | 2.73 | 2.52 | -0.21 | 2.96 | 0.23 | 4.32 | 1.59 | 3.32 | 0.59 |
| 172 | 6.06 | 6.06 | 0.00 | 8.16 | 2.10 | 8.29 | 2.23 | 6.12 | 0.06 |
| 173 | 4.57 | 4.86 | 0.28 | 6.05 | 1.47 | 6.75 | 2.18 | 5.12 | 0.54 |
| 174 | 7.27 | 6.71 | -0.56 | 8.57 | 1.30 | 8.49 | 1.22 | 7.76 | 0.49 |
| 175 | 7.95 | 7.63 | -0.32 | 10.13 | 2.18 | 9.95 | 2.01 | 8.83 | 0.88 |
| 176 | 3.43 | 3.75 | 0.31 | 3.61 | 0.18 | 4.57 | 1.13 | 3.41 | -0.03 |
| 177 | 4.43 | 4.95 | 0.52 | 5.94 | 1.51 | 6.30 | 1.88 | 4.70 | 0.27 |
| 178 | 5.25 | 4.67 | -0.58 | 6.37 | 1.12 | 7.70 | 2.46 | 5.48 | 0.23 |

| | | | | | | | | | |
|---|---|---|---|---|---|---|---|---|---|
| 179 | 4.22 | 4.38 | 0.16 | 6.31 | 2.09 | 5.84 | 1.62 | 4.15 | -0.07 |
| 180 | 2.85 | 2.84 | -0.01 | 3.73 | 0.88 | 3.46 | 0.61 | 2.78 | -0.07 |
| 181 | 5.89 | 5.55 | -0.33 | 6.72 | 0.83 | 7.42 | 1.53 | 5.97 | 0.08 |
| 182 | 4.61 | 4.66 | 0.06 | 6.04 | 1.44 | 6.20 | 1.59 | 5.05 | 0.44 |
| 183 | 3.78 | 3.90 | 0.12 | 6.00 | 2.21 | 5.81 | 2.03 | 4.03 | 0.24 |
| 184 | 4.66 | 3.98 | -0.68 | 6.47 | 1.81 | 6.16 | 1.50 | 4.86 | 0.20 |
| 185 | 7.47 | 7.14 | -0.33 | 9.84 | 2.37 | 9.96 | 2.49 | 7.78 | 0.31 |
| 186 | 6.88 | 7.02 | 0.13 | 8.79 | 1.91 | 8.74 | 1.86 | 6.93 | 0.04 |
| 187 | 2.94 | 2.21 | -0.73 | 2.74 | -0.21 | 2.77 | -0.17 | 3.49 | 0.55 |
| 188 | 6.78 | 6.55 | -0.23 | 8.92 | 2.14 | 9.41 | 2.63 | 7.14 | 0.36 |
| 189 | 3.13 | 3.31 | 0.18 | 4.13 | 0.99 | 4.23 | 1.10 | 2.14 | -0.99 |
| 190 | 5.18 | 4.76 | -0.43 | 6.71 | 1.53 | 7.06 | 1.88 | 4.92 | -0.26 |
| 191 | 3.44 | 3.26 | -0.18 | 4.99 | 1.55 | 4.65 | 1.21 | 3.11 | -0.33 |
| 192 | 3.95 | 4.24 | 0.30 | 5.96 | 2.01 | 4.85 | 0.90 | 4.52 | 0.58 |
| 193 | 2.15 | 2.01 | -0.13 | 3.51 | 1.36 | 4.06 | 1.91 | 2.11 | -0.04 |
| 194 | 1.27 | 1.78 | 0.51 | 2.01 | 0.74 | 2.92 | 1.64 | 1.78 | 0.51 |
| 195 | 7.77 | 8.14 | 0.37 | 10.72 | 2.95 | 10.09 | 2.33 | 8.59 | 0.83 |
| 196 | 4.44 | 3.97 | -0.47 | 5.29 | 0.85 | 4.48 | 0.04 | 5.03 | 0.59 |
| 197 | 7.09 | 6.20 | -0.89 | 8.63 | 1.54 | 8.26 | 1.17 | 8.18 | 1.09 |
| 198 | 7.12 | 6.37 | -0.76 | 8.23 | 1.11 | 7.80 | 0.67 | 7.61 | 0.48 |
| 199 | 2.48 | 2.68 | 0.21 | 3.51 | 1.03 | 3.34 | 0.87 | 2.50 | 0.02 |
| 200 | 4.49 | 4.10 | -0.39 | 4.34 | -0.15 | 3.42 | -1.06 | 5.27 | 0.78 |
| 201 | 6.80 | 6.29 | -0.51 | 8.49 | 1.69 | 8.46 | 1.67 | 7.03 | 0.23 |
| 202 | 4.02 | 3.50 | -0.52 | 5.05 | 1.03 | 5.63 | 1.61 | 4.15 | 0.13 |
| 203 | 3.09 | 3.94 | 0.85 | 3.46 | 0.37 | 3.96 | 0.88 | 3.57 | 0.48 |
| 204 | 3.87 | 3.65 | -0.22 | 4.19 | 0.32 | 3.43 | -0.44 | 3.89 | 0.02 |
| 205 | 5.09 | 4.66 | -0.43 | 6.90 | 1.81 | 7.31 | 2.21 | 5.84 | 0.75 |
| 206 | 4.72 | 4.34 | -0.38 | 5.98 | 1.27 | 5.22 | 0.50 | 4.88 | 0.16 |
| 207 | 5.98 | 5.38 | -0.60 | 7.69 | 1.71 | 7.49 | 1.51 | 6.05 | 0.07 |
| 208 | 5.52 | 4.99 | -0.52 | 7.33 | 1.81 | 6.19 | 0.68 | 5.68 | 0.17 |
| 209 | 1.53 | 1.39 | -0.14 | 1.86 | 0.33 | 1.13 | -0.40 | 1.25 | -0.28 |
| 210 | 2.23 | 2.88 | 0.64 | 4.01 | 1.77 | 4.33 | 2.10 | 2.66 | 0.43 |
| 211 | 7.37 | 6.39 | -0.98 | 8.55 | 1.17 | 8.42 | 1.05 | 8.28 | 0.91 |
| 212 | 2.36 | 2.15 | -0.22 | 3.25 | 0.88 | 3.10 | 0.74 | 2.80 | 0.43 |
| 213 | 4.64 | 4.37 | -0.26 | 6.90 | 2.26 | 6.11 | 1.47 | 5.11 | 0.47 |
| 214 | 2.06 | 1.03 | -1.03 | 1.28 | -0.77 | 1.01 | -1.05 | 1.68 | -0.38 |
| 215 | 4.94 | 4.76 | -0.18 | 6.38 | 1.44 | 7.22 | 2.28 | 5.31 | 0.37 |
| 216 | 4.00 | 3.81 | -0.19 | 5.55 | 1.55 | 4.67 | 0.66 | 4.92 | 0.91 |
| 217 | 3.88 | 3.88 | 0.00 | 4.82 | 0.94 | 4.68 | 0.80 | 4.47 | 0.59 |
| 218 | 4.94 | 4.40 | -0.54 | 5.72 | 0.78 | 6.48 | 1.54 | 4.90 | -0.04 |
| 219 | 1.13 | 1.21 | 0.08 | 1.36 | 0.22 | 2.67 | 1.54 | 1.24 | 0.11 |
| 220 | 4.17 | 3.72 | -0.45 | 4.86 | 0.69 | 5.20 | 1.03 | 4.53 | 0.36 |
| 221 | 2.69 | 2.61 | -0.08 | 2.77 | 0.08 | 3.90 | 1.21 | 2.71 | 0.02 |
| 222 | 6.17 | 6.20 | 0.03 | 8.82 | 2.65 | 8.35 | 2.18 | 7.47 | 1.30 |
| 223 | 2.51 | 2.24 | -0.27 | 2.53 | 0.03 | 3.95 | 1.45 | 2.73 | 0.22 |

| RMSE | | 0.48 | 1.27 | 1.61 | 0.45 |
|---|---|---|---|---|---|

Table S14. Dipole moments in Debye ($\mu_M$) of 90 β-xylose conformers computed by B3LYP/aug-cc-pVTZ and tested force fields

| Label | B3LYP /aug-cc-pVTZ | PBDPC25 | | CHARMM36 | | GLYCAM06 | | AMOEBA | |
|---|---|---|---|---|---|---|---|---|---|
| | | $\mu_M$ | Δ | $\mu_M$ | Δ | $\mu_M$ | Δ | $\mu_M$ | Δ |
| 1 | 1.97 | 2.20 | 0.23 | 3.79 | 1.82 | 3.10 | 1.13 | 0.96 | -1.01 |
| 2 | 1.81 | 1.70 | -0.11 | 2.13 | 0.32 | 2.79 | 0.98 | 2.89 | 1.08 |
| 3 | 3.54 | 3.47 | -0.07 | 4.64 | 1.09 | 5.04 | 1.49 | 4.12 | 0.58 |
| 4 | 2.28 | 2.11 | -0.17 | 2.88 | 0.60 | 2.78 | 0.50 | 2.34 | 0.06 |
| 5 | 3.97 | 3.86 | -0.12 | 5.33 | 1.36 | 4.99 | 1.02 | 3.59 | -0.39 |
| 6 | 1.49 | 1.43 | -0.05 | 2.49 | 1.00 | 1.54 | 0.05 | 1.04 | -0.45 |
| 7 | 1.32 | 1.63 | 0.31 | 2.18 | 0.86 | 2.27 | 0.94 | 1.99 | 0.67 |
| 8 | 2.74 | 2.82 | 0.09 | 4.30 | 1.57 | 3.84 | 1.11 | 2.48 | -0.26 |
| 9 | 2.69 | 2.59 | -0.10 | 3.71 | 1.02 | 3.57 | 0.88 | 2.87 | 0.18 |
| 10 | 1.61 | 1.63 | 0.03 | 2.47 | 0.87 | 2.60 | 0.99 | 1.75 | 0.14 |
| 11 | 2.40 | 2.54 | 0.14 | 3.39 | 0.99 | 4.10 | 1.70 | 3.27 | 0.87 |
| 12 | 2.17 | 2.27 | 0.10 | 2.99 | 0.82 | 2.94 | 0.77 | 2.82 | 0.65 |
| 13 | 2.69 | 2.49 | -0.19 | 3.49 | 0.81 | 3.36 | 0.68 | 2.64 | -0.05 |
| 14 | 4.87 | 4.64 | -0.23 | 6.83 | 1.96 | 6.60 | 1.73 | 5.19 | 0.32 |
| 15 | 1.80 | 1.75 | -0.05 | 2.47 | 0.67 | 1.94 | 0.13 | 1.86 | 0.05 |
| 16 | 1.39 | 1.78 | 0.39 | 1.85 | 0.46 | 2.46 | 1.07 | 1.56 | 0.17 |
| 17 | 2.14 | 2.18 | 0.04 | 3.20 | 1.06 | 4.19 | 2.05 | 2.74 | 0.60 |
| 18 | 2.44 | 2.58 | 0.15 | 3.72 | 1.28 | 3.83 | 1.39 | 2.36 | -0.08 |
| 19 | 2.19 | 1.91 | -0.28 | 3.33 | 1.14 | 3.04 | 0.85 | 2.39 | 0.20 |
| 20 | 2.81 | 2.68 | -0.13 | 3.87 | 1.06 | 4.32 | 1.51 | 3.12 | 0.30 |
| 21 | 1.50 | 1.44 | -0.06 | 2.50 | 1.00 | 1.55 | 0.05 | 1.06 | -0.44 |
| 22 | 3.29 | 3.04 | -0.25 | 4.55 | 1.26 | 4.28 | 0.99 | 3.50 | 0.22 |
| 23 | 1.53 | 1.05 | -0.48 | 1.78 | 0.25 | 1.51 | -0.01 | 1.43 | -0.10 |
| 24 | 1.68 | 1.65 | -0.02 | 2.20 | 0.52 | 2.11 | 0.44 | 2.07 | 0.40 |
| 25 | 1.85 | 1.53 | -0.32 | 2.41 | 0.56 | 1.70 | -0.15 | 1.57 | -0.28 |
| 26 | 2.78 | 2.66 | -0.12 | 3.87 | 1.09 | 3.61 | 0.83 | 3.02 | 0.24 |
| 27 | 1.91 | 1.98 | 0.07 | 2.74 | 0.83 | 3.02 | 1.11 | 2.71 | 0.80 |
| 28 | 3.09 | 2.97 | -0.12 | 4.63 | 1.54 | 4.34 | 1.25 | 3.30 | 0.21 |
| 29 | 2.18 | 2.31 | 0.12 | 2.60 | 0.42 | 3.37 | 1.19 | 3.13 | 0.95 |
| 30 | 2.54 | 2.48 | -0.06 | 3.42 | 0.88 | 3.15 | 0.61 | 2.24 | -0.30 |
| 31 | 3.47 | 3.60 | 0.12 | 5.28 | 1.81 | 4.78 | 1.31 | 2.88 | -0.60 |
| 32 | 2.89 | 2.63 | -0.26 | 3.80 | 0.91 | 3.95 | 1.07 | 3.57 | 0.68 |
| 33 | 2.13 | 1.91 | -0.21 | 2.84 | 0.71 | 3.00 | 0.88 | 3.16 | 1.03 |
| 34 | 2.13 | 1.96 | -0.17 | 2.62 | 0.49 | 3.35 | 1.22 | 3.58 | 1.45 |
| 35 | 1.94 | 1.92 | -0.02 | 2.95 | 1.01 | 2.80 | 0.86 | 2.57 | 0.63 |
| 36 | 2.09 | 2.04 | -0.05 | 3.38 | 1.29 | 2.69 | 0.60 | 1.53 | -0.56 |
| 37 | 0.98 | 1.27 | 0.29 | 1.73 | 0.75 | 2.34 | 1.36 | 2.30 | 1.32 |
| 38 | 4.32 | 4.10 | -0.21 | 5.96 | 1.64 | 6.11 | 1.79 | 4.85 | 0.53 |
| 39 | 3.57 | 3.46 | -0.11 | 4.91 | 1.35 | 4.50 | 0.94 | 3.10 | -0.47 |

| | | | | | | | | | |
|---|---|---|---|---|---|---|---|---|---|
| 40 | 1.12 | 0.68 | -0.44 | 1.13 | 0.01 | 1.42 | 0.30 | 2.20 | 1.08 |
| 41 | 2.78 | 2.63 | -0.16 | 4.03 | 1.24 | 5.06 | 2.27 | 3.93 | 1.15 |
| 42 | 1.93 | 2.04 | 0.11 | 2.74 | 0.81 | 3.53 | 1.60 | 2.89 | 0.96 |
| 43 | 1.61 | 2.11 | 0.50 | 2.60 | 0.99 | 2.65 | 1.04 | 2.07 | 0.46 |
| 44 | 1.63 | 1.45 | -0.18 | 1.83 | 0.20 | 2.33 | 0.70 | 2.04 | 0.42 |
| 45 | 3.86 | 3.78 | -0.08 | 5.29 | 1.43 | 4.74 | 0.88 | 3.13 | -0.73 |
| 46 | 3.13 | 3.06 | -0.07 | 4.17 | 1.04 | 4.36 | 1.23 | 3.02 | -0.12 |
| 47 | 2.47 | 2.22 | -0.25 | 3.18 | 0.71 | 2.63 | 0.16 | 1.94 | -0.54 |
| 48 | 1.84 | 1.70 | -0.14 | 1.92 | 0.08 | 2.29 | 0.45 | 2.68 | 0.84 |
| 49 | 2.70 | 2.62 | -0.08 | 3.42 | 0.72 | 3.31 | 0.61 | 3.23 | 0.52 |
| 50 | 3.40 | 3.33 | -0.06 | 4.14 | 0.75 | 4.50 | 1.10 | 4.26 | 0.86 |
| 51 | 2.75 | 2.49 | -0.26 | 3.70 | 0.95 | 3.71 | 0.95 | 2.75 | 0.00 |
| 52 | 2.27 | 2.46 | 0.19 | 2.99 | 0.73 | 2.93 | 0.66 | 2.52 | 0.25 |
| 53 | 2.64 | 2.71 | 0.07 | 3.29 | 0.65 | 3.70 | 1.05 | 3.34 | 0.70 |
| 54 | 2.88 | 3.00 | 0.12 | 3.82 | 0.94 | 4.47 | 1.59 | 3.34 | 0.46 |
| 55 | 1.81 | 1.68 | -0.13 | 3.08 | 1.27 | 2.15 | 0.34 | 1.22 | -0.59 |
| 56 | 2.45 | 2.49 | 0.04 | 3.81 | 1.36 | 3.87 | 1.42 | 2.34 | -0.11 |
| 57 | 2.03 | 1.99 | -0.04 | 2.53 | 0.51 | 3.40 | 1.37 | 2.34 | 0.32 |
| 58 | 0.88 | 0.91 | 0.03 | 1.07 | 0.19 | 1.98 | 1.10 | 2.08 | 1.20 |
| 59 | 1.35 | 1.40 | 0.05 | 1.82 | 0.47 | 2.15 | 0.80 | 2.12 | 0.76 |
| 60 | 2.78 | 2.84 | 0.06 | 3.97 | 1.19 | 3.80 | 1.02 | 3.14 | 0.36 |
| 61 | 1.95 | 1.88 | -0.07 | 2.55 | 0.60 | 2.65 | 0.69 | 2.61 | 0.66 |
| 62 | 1.31 | 1.20 | -0.12 | 1.75 | 0.43 | 2.47 | 1.15 | 2.15 | 0.83 |
| 63 | 2.00 | 1.86 | -0.14 | 2.66 | 0.66 | 3.74 | 1.74 | 3.35 | 1.35 |
| 64 | 2.48 | 2.51 | 0.03 | 3.54 | 1.06 | 4.10 | 1.61 | 3.60 | 1.12 |
| 65 | 2.76 | 2.52 | -0.24 | 3.94 | 1.18 | 4.16 | 1.40 | 2.88 | 0.12 |
| 66 | 0.74 | 0.67 | -0.06 | 1.20 | 0.46 | 1.22 | 0.49 | 1.24 | 0.51 |
| 67 | 1.92 | 2.05 | 0.13 | 2.97 | 1.05 | 3.85 | 1.93 | 3.24 | 1.32 |
| 68 | 2.20 | 2.13 | -0.07 | 3.04 | 0.84 | 3.17 | 0.97 | 2.54 | 0.33 |
| 69 | 1.76 | 1.64 | -0.13 | 2.42 | 0.66 | 2.53 | 0.76 | 1.45 | -0.32 |
| 70 | 3.88 | 3.66 | -0.22 | 5.53 | 1.65 | 4.83 | 0.95 | 3.11 | -0.78 |
| 71 | 2.57 | 2.13 | -0.44 | 3.54 | 0.97 | 2.82 | 0.25 | 1.88 | -0.69 |
| 72 | 2.51 | 2.16 | -0.35 | 3.06 | 0.55 | 3.11 | 0.61 | 2.98 | 0.47 |
| 73 | 2.32 | 2.79 | 0.48 | 3.54 | 1.22 | 3.35 | 1.04 | 2.27 | -0.05 |
| 74 | 3.16 | 3.20 | 0.04 | 4.89 | 1.73 | 4.71 | 1.55 | 3.58 | 0.42 |
| 75 | 2.68 | 2.70 | 0.02 | 3.90 | 1.22 | 4.26 | 1.58 | 2.99 | 0.31 |
| 76 | 4.52 | 4.82 | 0.30 | 6.50 | 1.98 | 6.79 | 2.27 | 4.68 | 0.16 |
| 77 | 1.91 | 2.23 | 0.32 | 2.77 | 0.86 | 2.50 | 0.59 | 2.29 | 0.39 |
| 78 | 1.52 | 1.26 | -0.26 | 2.18 | 0.66 | 2.14 | 0.62 | 1.59 | 0.07 |
| 79 | 1.73 | 1.54 | -0.20 | 2.11 | 0.38 | 2.09 | 0.36 | 1.49 | -0.24 |
| 80 | 1.31 | 1.46 | 0.14 | 1.53 | 0.22 | 2.11 | 0.80 | 1.43 | 0.11 |
| 81 | 3.46 | 3.18 | -0.27 | 4.86 | 1.41 | 4.76 | 1.30 | 3.64 | 0.18 |
| 82 | 1.75 | 1.67 | -0.09 | 2.51 | 0.76 | 2.77 | 1.02 | 2.06 | 0.30 |
| 83 | 2.02 | 1.91 | -0.11 | 3.20 | 1.18 | 2.89 | 0.87 | 2.23 | 0.21 |
| 84 | 1.98 | 1.79 | -0.19 | 3.31 | 1.33 | 2.75 | 0.77 | 2.28 | 0.30 |

| | | | | | | | | | |
|---|---|---|---|---|---|---|---|---|---|
| 85 | 2.00 | 2.00 | 0.00 | 3.07 | 1.07 | 4.05 | 2.05 | 2.26 | 0.26 |
| 86 | 0.48 | 0.62 | 0.14 | 0.76 | 0.28 | 1.00 | 0.52 | 0.73 | 0.25 |
| 87 | 2.94 | 3.36 | 0.41 | 4.08 | 1.13 | 4.42 | 1.47 | 2.98 | 0.03 |
| 88 | 2.21 | 2.09 | -0.12 | 3.47 | 1.27 | 2.96 | 0.75 | 2.22 | 0.01 |
| 89 | 1.69 | 1.27 | -0.42 | 2.50 | 0.81 | 1.99 | 0.30 | 1.71 | 0.01 |
| 90 | 0.34 | 0.56 | 0.22 | 0.48 | 0.15 | 1.24 | 0.90 | 0.96 | 0.62 |
| RMSE | | | 0.20 | | 1.02 | | 1.11 | | 0.61 |

Table S15. Dipole moments ($\mu_M$) in Debye of 168 β-mannose conformers computed by B3LYP/aug-cc-pVTZ and tested force fields

| Label | B3LYP /aug-cc-pVTZ | PBDPC25 | | CHARMM36 | | GLYCAM06 | | AMOEBA | |
|---|---|---|---|---|---|---|---|---|---|
| | | $\mu_M$ | Δ | $\mu_M$ | Δ | $\mu_M$ | Δ | $\mu_M$ | Δ |
| 1 | 1.51 | 1.63 | 0.12 | 2.90 | 1.39 | 1.78 | 0.27 | 1.64 | 0.13 |
| 2 | 4.92 | 5.06 | 0.14 | 7.06 | 2.15 | 6.93 | 2.01 | 5.91 | 0.99 |
| 3 | 1.17 | 1.06 | -0.11 | 1.75 | 0.58 | 1.07 | -0.10 | 1.76 | 0.59 |
| 4 | 1.46 | 1.26 | -0.20 | 2.33 | 0.87 | 1.53 | 0.07 | 1.26 | -0.20 |
| 5 | 1.65 | 1.57 | -0.08 | 2.36 | 0.71 | 2.34 | 0.69 | 2.08 | 0.43 |
| 6 | 2.48 | 2.09 | -0.38 | 3.45 | 0.98 | 3.14 | 0.66 | 2.70 | 0.22 |
| 7 | 1.96 | 1.97 | 0.01 | 2.62 | 0.66 | 2.33 | 0.37 | 1.98 | 0.01 |
| 8 | 2.86 | 2.52 | -0.34 | 3.79 | 0.93 | 3.73 | 0.88 | 3.43 | 0.57 |
| 9 | 3.16 | 2.84 | -0.32 | 4.03 | 0.87 | 4.20 | 1.04 | 3.92 | 0.76 |
| 10 | 4.12 | 4.15 | 0.03 | 5.82 | 1.70 | 5.71 | 1.60 | 4.30 | 0.18 |
| 11 | 2.11 | 2.11 | 0.00 | 2.55 | 0.44 | 2.60 | 0.49 | 3.07 | 0.96 |
| 12 | 2.28 | 2.50 | 0.22 | 3.61 | 1.33 | 2.35 | 0.07 | 2.02 | -0.26 |
| 13 | 2.29 | 2.55 | 0.26 | 2.70 | 0.41 | 2.62 | 0.33 | 2.79 | 0.50 |
| 14 | 2.00 | 2.11 | 0.11 | 1.86 | -0.14 | 2.99 | 1.00 | 3.37 | 1.37 |
| 15 | 3.32 | 2.75 | -0.58 | 3.48 | 0.16 | 3.74 | 0.42 | 3.87 | 0.55 |
| 16 | 1.88 | 1.43 | -0.46 | 1.44 | -0.45 | 2.39 | 0.50 | 2.86 | 0.98 |
| 17 | 2.08 | 2.21 | 0.13 | 2.78 | 0.70 | 2.89 | 0.81 | 2.91 | 0.83 |
| 18 | 1.43 | 1.33 | -0.10 | 2.37 | 0.94 | 2.63 | 1.20 | 1.27 | -0.16 |
| 19 | 2.74 | 2.72 | -0.02 | 4.13 | 1.39 | 4.39 | 1.64 | 2.59 | -0.15 |
| 20 | 2.39 | 2.22 | -0.17 | 3.21 | 0.82 | 4.09 | 1.70 | 3.06 | 0.67 |
| 21 | 1.36 | 1.66 | 0.30 | 2.66 | 1.30 | 2.62 | 1.25 | 1.81 | 0.45 |
| 22 | 2.42 | 2.27 | -0.15 | 3.13 | 0.71 | 4.00 | 1.58 | 3.16 | 0.73 |
| 23 | 2.58 | 2.68 | 0.10 | 4.24 | 1.66 | 3.46 | 0.88 | 2.13 | -0.45 |
| 24 | 2.72 | 2.86 | 0.14 | 4.12 | 1.40 | 3.39 | 0.67 | 2.58 | -0.14 |
| 25 | 3.73 | 3.63 | -0.09 | 5.22 | 1.49 | 5.22 | 1.49 | 4.31 | 0.59 |
| 26 | 3.08 | 3.19 | 0.11 | 4.26 | 1.18 | 4.61 | 1.53 | 3.59 | 0.51 |
| 27 | 3.25 | 3.15 | -0.10 | 4.45 | 1.20 | 3.94 | 0.69 | 3.76 | 0.51 |
| 28 | 4.05 | 3.80 | -0.25 | 5.61 | 1.56 | 5.14 | 1.09 | 4.70 | 0.65 |
| 29 | 3.17 | 3.05 | -0.11 | 4.56 | 1.40 | 3.01 | -0.16 | 3.29 | 0.12 |
| 30 | 3.69 | 3.62 | -0.07 | 5.68 | 1.99 | 4.43 | 0.74 | 3.95 | 0.26 |
| 31 | 2.38 | 2.48 | 0.09 | 3.69 | 1.30 | 2.84 | 0.46 | 2.47 | 0.09 |
| 32 | 2.12 | 2.46 | 0.34 | 3.24 | 1.12 | 2.74 | 0.62 | 2.46 | 0.34 |
| 33 | 3.28 | 3.32 | 0.03 | 4.99 | 1.71 | 4.23 | 0.94 | 3.16 | -0.13 |

| | | | | | | | | | |
|---|---|---|---|---|---|---|---|---|---|
| 34 | 2.63 | 2.70 | 0.08 | 3.48 | 0.86 | 3.58 | 0.96 | 2.62 | -0.01 |
| 35 | 2.94 | 3.09 | 0.15 | 3.32 | 0.39 | 4.14 | 1.21 | 3.41 | 0.47 |
| 36 | 2.56 | 2.60 | 0.03 | 3.30 | 0.74 | 3.02 | 0.45 | 2.65 | 0.08 |
| 37 | 2.55 | 2.91 | 0.36 | 3.67 | 1.12 | 3.34 | 0.79 | 2.41 | -0.14 |
| 38 | 2.28 | 2.21 | -0.07 | 3.51 | 1.22 | 3.35 | 1.07 | 2.77 | 0.49 |
| 39 | 1.60 | 1.79 | 0.19 | 2.27 | 0.67 | 2.08 | 0.48 | 2.57 | 0.97 |
| 40 | 0.93 | 0.97 | 0.04 | 1.80 | 0.87 | 0.29 | -0.64 | 1.48 | 0.55 |
| 41 | 2.62 | 2.24 | -0.38 | 2.92 | 0.30 | 3.38 | 0.76 | 2.46 | -0.16 |
| 42 | 1.89 | 1.77 | -0.12 | 2.40 | 0.52 | 3.32 | 1.43 | 3.00 | 1.12 |
| 43 | 3.39 | 3.27 | -0.12 | 4.73 | 1.34 | 4.33 | 0.94 | 3.38 | -0.01 |
| 44 | 3.70 | 3.90 | 0.19 | 4.60 | 0.90 | 4.80 | 1.10 | 4.20 | 0.50 |
| 45 | 3.77 | 3.77 | 0.00 | 5.28 | 1.51 | 4.70 | 0.94 | 3.28 | -0.48 |
| 46 | 3.47 | 3.77 | 0.30 | 4.72 | 1.25 | 4.11 | 0.63 | 3.26 | -0.21 |
| 47 | 2.37 | 2.23 | -0.13 | 3.14 | 0.77 | 2.71 | 0.35 | 2.20 | -0.16 |
| 48 | 3.44 | 3.35 | -0.09 | 4.82 | 1.37 | 4.66 | 1.21 | 3.57 | 0.13 |
| 49 | 3.51 | 3.63 | 0.12 | 4.82 | 1.31 | 4.62 | 1.12 | 3.82 | 0.31 |
| 50 | 2.62 | 2.68 | 0.06 | 3.78 | 1.16 | 3.32 | 0.70 | 3.11 | 0.49 |
| 51 | 3.52 | 3.41 | -0.11 | 4.55 | 1.03 | 5.06 | 1.54 | 4.17 | 0.65 |
| 52 | 2.77 | 2.83 | 0.06 | 3.33 | 0.56 | 4.06 | 1.29 | 3.72 | 0.95 |
| 53 | 4.22 | 4.22 | -0.01 | 5.22 | 1.00 | 6.03 | 1.81 | 4.66 | 0.44 |
| 54 | 2.82 | 2.92 | 0.11 | 3.62 | 0.80 | 4.31 | 1.49 | 3.92 | 1.10 |
| 55 | 2.43 | 2.54 | 0.11 | 3.25 | 0.82 | 3.63 | 1.20 | 3.14 | 0.71 |
| 56 | 4.35 | 4.28 | -0.07 | 5.62 | 1.27 | 6.43 | 2.08 | 5.43 | 1.08 |
| 57 | 1.44 | 1.38 | -0.06 | 1.56 | 0.12 | 2.35 | 0.91 | 2.31 | 0.87 |
| 58 | 3.20 | 3.26 | 0.06 | 4.19 | 0.99 | 4.89 | 1.69 | 4.06 | 0.87 |
| 59 | 2.53 | 3.05 | 0.52 | 3.41 | 0.88 | 3.33 | 0.79 | 2.73 | 0.20 |
| 60 | 1.12 | 1.05 | -0.07 | 1.72 | 0.60 | 1.19 | 0.07 | 1.73 | 0.61 |
| 61 | 3.85 | 4.16 | 0.31 | 5.00 | 1.15 | 5.26 | 1.41 | 4.45 | 0.60 |
| 62 | 2.77 | 2.23 | -0.54 | 2.66 | -0.11 | 3.25 | 0.47 | 3.57 | 0.80 |
| 63 | 2.09 | 1.86 | -0.23 | 2.78 | 0.69 | 2.81 | 0.72 | 2.39 | 0.30 |
| 64 | 3.43 | 3.13 | -0.29 | 4.55 | 1.13 | 5.04 | 1.61 | 3.80 | 0.37 |
| 65 | 1.96 | 1.88 | -0.08 | 2.84 | 0.88 | 2.29 | 0.33 | 1.45 | -0.51 |
| 66 | 0.93 | 0.84 | -0.09 | 1.52 | 0.58 | 1.74 | 0.81 | 0.79 | -0.14 |
| 67 | 1.37 | 1.11 | -0.26 | 1.88 | 0.51 | 2.85 | 1.48 | 2.18 | 0.81 |
| 68 | 4.46 | 4.20 | -0.25 | 5.23 | 0.78 | 5.75 | 1.29 | 5.19 | 0.73 |
| 69 | 2.09 | 2.11 | 0.02 | 2.49 | 0.40 | 2.38 | 0.29 | 2.91 | 0.82 |
| 70 | 4.71 | 4.55 | -0.17 | 6.30 | 1.59 | 6.98 | 2.27 | 5.26 | 0.54 |
| 71 | 1.48 | 1.37 | -0.11 | 2.51 | 1.03 | 2.40 | 0.92 | 1.31 | -0.17 |
| 72 | 4.00 | 3.84 | -0.16 | 4.73 | 0.72 | 5.36 | 1.36 | 5.24 | 1.24 |
| 73 | 0.64 | 0.57 | -0.07 | 1.49 | 0.84 | 1.57 | 0.92 | 1.38 | 0.74 |
| 74 | 3.70 | 3.61 | -0.09 | 4.50 | 0.80 | 4.44 | 0.74 | 4.40 | 0.70 |
| 75 | 2.01 | 1.68 | -0.33 | 2.29 | 0.28 | 3.30 | 1.29 | 2.83 | 0.83 |
| 76 | 2.02 | 1.96 | -0.06 | 2.20 | 0.18 | 1.58 | -0.44 | 2.36 | 0.34 |
| 77 | 4.18 | 4.19 | 0.01 | 5.13 | 0.95 | 6.27 | 2.09 | 5.50 | 1.31 |
| 78 | 4.12 | 4.09 | -0.03 | 6.24 | 2.12 | 5.32 | 1.20 | 3.48 | -0.65 |

| | | | | | | | | | |
|---|---|---|---|---|---|---|---|---|---|
| 79 | 0.88 | 0.90 | 0.02 | 1.44 | 0.56 | 0.95 | 0.07 | 1.16 | 0.28 |
| 80 | 4.92 | 5.32 | 0.40 | 6.64 | 1.72 | 6.57 | 1.65 | 5.11 | 0.19 |
| 81 | 2.79 | 3.09 | 0.30 | 4.48 | 1.69 | 4.10 | 1.31 | 2.33 | -0.46 |
| 82 | 1.78 | 1.22 | -0.55 | 2.35 | 0.57 | 1.80 | 0.02 | 1.81 | 0.03 |
| 83 | 1.88 | 2.01 | 0.13 | 2.46 | 0.58 | 3.05 | 1.17 | 2.26 | 0.38 |
| 84 | 3.81 | 4.03 | 0.22 | 5.43 | 1.63 | 5.29 | 1.48 | 3.63 | -0.18 |
| 85 | 3.96 | 4.10 | 0.14 | 5.47 | 1.51 | 5.72 | 1.76 | 4.29 | 0.33 |
| 86 | 2.78 | 2.75 | -0.03 | 2.87 | 0.09 | 3.37 | 0.58 | 3.36 | 0.58 |
| 87 | 1.26 | 1.06 | -0.21 | 1.52 | 0.26 | 2.30 | 1.03 | 1.88 | 0.61 |
| 88 | 4.48 | 4.74 | 0.26 | 6.67 | 2.19 | 6.61 | 2.13 | 4.45 | -0.03 |
| 89 | 3.42 | 3.69 | 0.26 | 4.82 | 1.40 | 4.52 | 1.09 | 3.48 | 0.05 |
| 90 | 1.60 | 1.41 | -0.19 | 2.04 | 0.45 | 2.31 | 0.71 | 1.70 | 0.11 |
| 91 | 3.33 | 3.03 | -0.30 | 3.86 | 0.53 | 4.71 | 1.39 | 3.54 | 0.22 |
| 92 | 2.95 | 3.29 | 0.35 | 4.64 | 1.70 | 4.43 | 1.48 | 3.01 | 0.06 |
| 93 | 4.67 | 4.31 | -0.36 | 5.88 | 1.21 | 6.80 | 2.13 | 4.98 | 0.31 |
| 94 | 2.88 | 2.41 | -0.47 | 3.00 | 0.12 | 4.34 | 1.46 | 3.60 | 0.71 |
| 95 | 1.04 | 0.83 | -0.22 | 0.80 | -0.24 | 2.11 | 1.07 | 1.86 | 0.81 |
| 96 | 3.46 | 3.72 | 0.26 | 4.79 | 1.32 | 5.24 | 1.78 | 3.71 | 0.24 |
| 97 | 1.34 | 1.02 | -0.33 | 0.97 | -0.37 | 1.74 | 0.40 | 1.94 | 0.60 |
| 98 | 3.11 | 3.25 | 0.14 | 3.73 | 0.62 | 3.93 | 0.83 | 3.52 | 0.41 |
| 99 | 2.32 | 2.44 | 0.12 | 3.95 | 1.63 | 3.08 | 0.75 | 1.90 | -0.43 |
| 100 | 2.76 | 2.37 | -0.39 | 3.97 | 1.21 | 3.19 | 0.43 | 3.25 | 0.49 |
| 101 | 3.00 | 2.80 | -0.20 | 3.57 | 0.57 | 3.73 | 0.73 | 3.54 | 0.54 |
| 102 | 4.22 | 4.13 | -0.09 | 5.62 | 1.40 | 5.58 | 1.37 | 4.44 | 0.22 |
| 103 | 3.60 | 3.43 | -0.18 | 4.66 | 1.06 | 4.99 | 1.39 | 5.04 | 1.44 |
| 104 | 2.11 | 1.86 | -0.25 | 2.43 | 0.32 | 2.71 | 0.60 | 3.27 | 1.16 |
| 105 | 4.64 | 4.32 | -0.31 | 6.00 | 1.37 | 6.51 | 1.87 | 6.07 | 1.43 |
| 106 | 1.69 | 1.53 | -0.17 | 2.27 | 0.58 | 1.12 | -0.57 | 1.59 | -0.10 |
| 107 | 2.01 | 1.64 | -0.37 | 2.32 | 0.31 | 2.61 | 0.60 | 2.25 | 0.24 |
| 108 | 1.87 | 1.74 | -0.12 | 2.05 | 0.18 | 2.40 | 0.54 | 2.91 | 1.05 |
| 109 | 3.69 | 3.39 | -0.30 | 5.19 | 1.50 | 4.63 | 0.94 | 4.09 | 0.40 |
| 110 | 3.29 | 3.00 | -0.29 | 4.44 | 1.15 | 3.88 | 0.59 | 3.76 | 0.47 |
| 111 | 2.65 | 2.34 | -0.30 | 4.28 | 1.63 | 3.28 | 0.63 | 2.61 | -0.03 |
| 112 | 3.21 | 3.44 | 0.23 | 4.90 | 1.68 | 3.72 | 0.51 | 3.40 | 0.18 |
| 113 | 2.88 | 3.09 | 0.21 | 3.70 | 0.81 | 3.50 | 0.62 | 3.42 | 0.54 |
| 114 | 1.99 | 1.84 | -0.14 | 2.46 | 0.47 | 2.87 | 0.88 | 2.01 | 0.02 |
| 115 | 0.34 | 0.47 | 0.13 | 0.96 | 0.61 | 0.80 | 0.45 | 0.77 | 0.43 |
| 116 | 3.10 | 3.16 | 0.05 | 4.31 | 1.20 | 3.74 | 0.64 | 3.35 | 0.24 |
| 117 | 4.54 | 4.61 | 0.07 | 5.79 | 1.25 | 5.11 | 0.57 | 4.55 | 0.01 |
| 118 | 2.57 | 2.55 | -0.02 | 3.04 | 0.47 | 3.52 | 0.96 | 2.90 | 0.33 |
| 119 | 2.78 | 2.66 | -0.12 | 2.53 | -0.25 | 3.75 | 0.97 | 3.73 | 0.94 |
| 120 | 1.79 | 1.89 | 0.10 | 2.85 | 1.06 | 1.97 | 0.18 | 1.39 | -0.39 |
| 121 | 3.89 | 3.98 | 0.09 | 5.59 | 1.71 | 5.40 | 1.52 | 3.65 | -0.23 |
| 122 | 2.70 | 3.07 | 0.38 | 3.91 | 1.21 | 3.29 | 0.59 | 2.29 | -0.41 |
| 123 | 4.20 | 4.48 | 0.28 | 5.90 | 1.70 | 5.60 | 1.40 | 3.93 | -0.27 |

| | | | | | | | | | |
|---|---|---|---|---|---|---|---|---|---|
| 124 | 1.98 | 2.20 | 0.22 | 3.18 | 1.20 | 2.77 | 0.79 | 1.74 | -0.24 |
| 125 | 3.51 | 3.65 | 0.15 | 4.19 | 0.69 | 5.05 | 1.54 | 4.04 | 0.53 |
| 126 | 2.78 | 3.22 | 0.44 | 4.63 | 1.85 | 3.75 | 0.97 | 2.60 | -0.18 |
| 127 | 2.00 | 2.52 | 0.52 | 3.73 | 1.73 | 3.12 | 1.12 | 1.72 | -0.28 |
| 128 | 2.49 | 2.95 | 0.46 | 4.73 | 2.24 | 3.96 | 1.47 | 2.40 | -0.10 |
| 129 | 2.41 | 2.47 | 0.06 | 3.05 | 0.64 | 3.53 | 1.12 | 3.29 | 0.88 |
| 130 | 2.08 | 2.24 | 0.16 | 3.27 | 1.19 | 2.86 | 0.78 | 2.32 | 0.24 |
| 131 | 3.28 | 3.71 | 0.44 | 4.48 | 1.20 | 3.99 | 0.71 | 3.17 | -0.11 |
| 132 | 1.62 | 1.59 | -0.03 | 1.70 | 0.09 | 1.85 | 0.24 | 1.97 | 0.35 |
| 133 | 5.68 | 5.86 | 0.18 | 7.39 | 1.71 | 7.11 | 1.43 | 5.78 | 0.10 |
| 134 | 5.57 | 5.65 | 0.08 | 7.51 | 1.94 | 7.82 | 2.25 | 6.18 | 0.61 |
| 135 | 2.07 | 2.28 | 0.21 | 2.69 | 0.62 | 3.03 | 0.96 | 2.20 | 0.13 |
| 136 | 2.28 | 2.43 | 0.15 | 3.39 | 1.12 | 3.19 | 0.92 | 2.45 | 0.18 |
| 137 | 0.96 | 1.07 | 0.11 | 1.53 | 0.58 | 2.34 | 1.38 | 1.33 | 0.38 |
| 138 | 3.11 | 3.11 | 0.00 | 4.79 | 1.68 | 4.15 | 1.04 | 3.12 | 0.01 |
| 139 | 3.43 | 3.62 | 0.19 | 5.19 | 1.76 | 4.49 | 1.06 | 3.08 | -0.35 |
| 140 | 3.21 | 3.19 | -0.02 | 4.50 | 1.29 | 4.21 | 1.00 | 3.45 | 0.24 |
| 141 | 2.67 | 2.93 | 0.26 | 2.68 | 0.01 | 4.06 | 1.39 | 3.60 | 0.93 |
| 142 | 3.61 | 3.78 | 0.17 | 5.71 | 2.10 | 4.70 | 1.09 | 3.68 | 0.07 |
| 143 | 2.55 | 2.84 | 0.30 | 3.25 | 0.70 | 3.65 | 1.10 | 3.10 | 0.55 |
| 144 | 1.29 | 1.54 | 0.25 | 2.51 | 1.21 | 1.35 | 0.06 | 1.51 | 0.22 |
| 145 | 1.87 | 2.15 | 0.28 | 3.05 | 1.18 | 2.81 | 0.94 | 2.34 | 0.48 |
| 146 | 3.92 | 3.69 | -0.23 | 5.01 | 1.09 | 5.13 | 1.22 | 4.03 | 0.12 |
| 147 | 1.98 | 2.09 | 0.11 | 2.85 | 0.87 | 1.85 | -0.13 | 2.00 | 0.02 |
| 148 | 3.42 | 3.43 | 0.01 | 5.11 | 1.69 | 4.44 | 1.02 | 3.76 | 0.33 |
| 149 | 4.84 | 4.80 | -0.04 | 6.83 | 1.99 | 6.48 | 1.64 | 5.30 | 0.46 |
| 150 | 3.26 | 3.40 | 0.14 | 4.69 | 1.43 | 4.43 | 1.17 | 3.46 | 0.20 |
| 151 | 4.83 | 4.79 | -0.04 | 6.46 | 1.63 | 6.70 | 1.87 | 5.15 | 0.32 |
| 152 | 3.53 | 3.62 | 0.09 | 4.61 | 1.08 | 4.25 | 0.72 | 3.70 | 0.17 |
| 153 | 3.82 | 4.04 | 0.22 | 4.66 | 0.83 | 5.09 | 1.27 | 4.41 | 0.59 |
| 154 | 4.58 | 4.47 | -0.12 | 5.74 | 1.16 | 6.13 | 1.55 | 5.02 | 0.43 |
| 155 | 1.54 | 1.33 | -0.22 | 2.08 | 0.54 | 2.14 | 0.60 | 1.79 | 0.24 |
| 156 | 3.98 | 4.29 | 0.31 | 5.34 | 1.35 | 4.54 | 0.56 | 4.17 | 0.19 |
| 157 | 3.68 | 3.81 | 0.12 | 4.89 | 1.20 | 4.10 | 0.41 | 3.79 | 0.11 |
| 158 | 3.03 | 3.30 | 0.27 | 4.23 | 1.20 | 3.84 | 0.81 | 3.59 | 0.56 |
| 159 | 3.81 | 3.59 | -0.22 | 4.56 | 0.75 | 5.34 | 1.53 | 4.55 | 0.74 |
| 160 | 3.98 | 4.28 | 0.30 | 4.99 | 1.01 | 5.41 | 1.42 | 4.10 | 0.12 |
| 161 | 4.29 | 4.31 | 0.03 | 5.68 | 1.39 | 5.47 | 1.18 | 4.15 | -0.14 |
| 162 | 2.23 | 2.20 | -0.03 | 2.68 | 0.45 | 2.39 | 0.16 | 1.84 | -0.39 |
| 163 | 3.93 | 3.79 | -0.13 | 5.33 | 1.40 | 5.19 | 1.26 | 3.94 | 0.01 |
| 164 | 3.38 | 3.65 | 0.28 | 4.65 | 1.27 | 4.07 | 0.69 | 3.80 | 0.42 |
| 165 | 2.65 | 2.36 | -0.29 | 3.41 | 0.76 | 3.37 | 0.72 | 3.32 | 0.67 |
| 166 | 1.49 | 1.78 | 0.29 | 2.52 | 1.03 | 1.37 | -0.12 | 1.84 | 0.35 |
| 167 | 2.91 | 2.33 | -0.59 | 3.63 | 0.71 | 3.58 | 0.67 | 2.78 | -0.14 |
| 168 | 2.56 | 2.59 | 0.03 | 4.12 | 1.56 | 2.88 | 0.32 | 2.47 | -0.09 |

| | | | | |
|---|---|---|---|---|
| RMSE | 0.23 | 1.14 | 1.10 | 0.54 |

Table S16. Dipole moments ($\mu_M$ in D) of 15 α/β-allose conformers computed by B3LYP/aug-cc-pVTZ and force field methods

| Label | B3LYP | PBDPC25 | | CHARMM36 | | GLYCAM06 | | AMOEBA | |
|---|---|---|---|---|---|---|---|---|---|
| | /aug-cc-pVTZ | $\mu_M$ | Δ | $\mu_M$ | Δ | $\mu_M$ | Δ | $\mu_M$ | Δ |
| 1 | 3.10 | 3.14 | 0.04 | 3.50 | 0.40 | 4.14 | 1.04 | 3.01 | -0.09 |
| 2 | 3.74 | 3.70 | -0.04 | 4.94 | 1.19 | 5.78 | 2.04 | 3.94 | 0.20 |
| 3 | 3.12 | 3.25 | 0.13 | 3.87 | 0.75 | 4.53 | 1.41 | 3.22 | 0.10 |
| 4 | 2.30 | 2.09 | -0.21 | 3.00 | 0.70 | 3.57 | 1.27 | 2.61 | 0.31 |
| 5 | 3.65 | 3.35 | -0.30 | 4.90 | 1.25 | 5.87 | 2.22 | 3.84 | 0.19 |
| 6 | 3.22 | 3.22 | 0.00 | 3.79 | 0.57 | 4.32 | 1.10 | 3.40 | 0.18 |
| 7 | 2.57 | 2.74 | 0.17 | 3.08 | 0.51 | 3.42 | 0.85 | 2.92 | 0.35 |
| 8 | 3.11 | 3.19 | 0.08 | 3.94 | 0.83 | 4.07 | 0.95 | 4.14 | 1.03 |
| 9 | 3.27 | 3.43 | 0.15 | 4.57 | 1.30 | 4.08 | 0.81 | 2.39 | -0.88 |
| 10 | 2.45 | 2.40 | -0.04 | 3.22 | 0.77 | 4.62 | 2.17 | 3.83 | 1.38 |
| 11 | 3.32 | 3.13 | -0.19 | 3.70 | 0.38 | 3.38 | 0.06 | 3.01 | -0.31 |
| 12 | 4.13 | 4.10 | -0.03 | 5.14 | 1.01 | 5.34 | 1.21 | 4.23 | 0.10 |
| 13 | 1.91 | 2.22 | 0.32 | 3.24 | 1.33 | 2.64 | 0.74 | 2.11 | 0.20 |
| 14 | 2.65 | 2.32 | -0.33 | 3.55 | 0.90 | 4.12 | 1.47 | 2.86 | 0.21 |
| 15 | 2.58 | 2.20 | -0.39 | 3.67 | 1.08 | 3.20 | 0.62 | 3.00 | 0.42 |
| RMSE | | | 0.18 | | 0.82 | | 1.18 | | 0.48 |

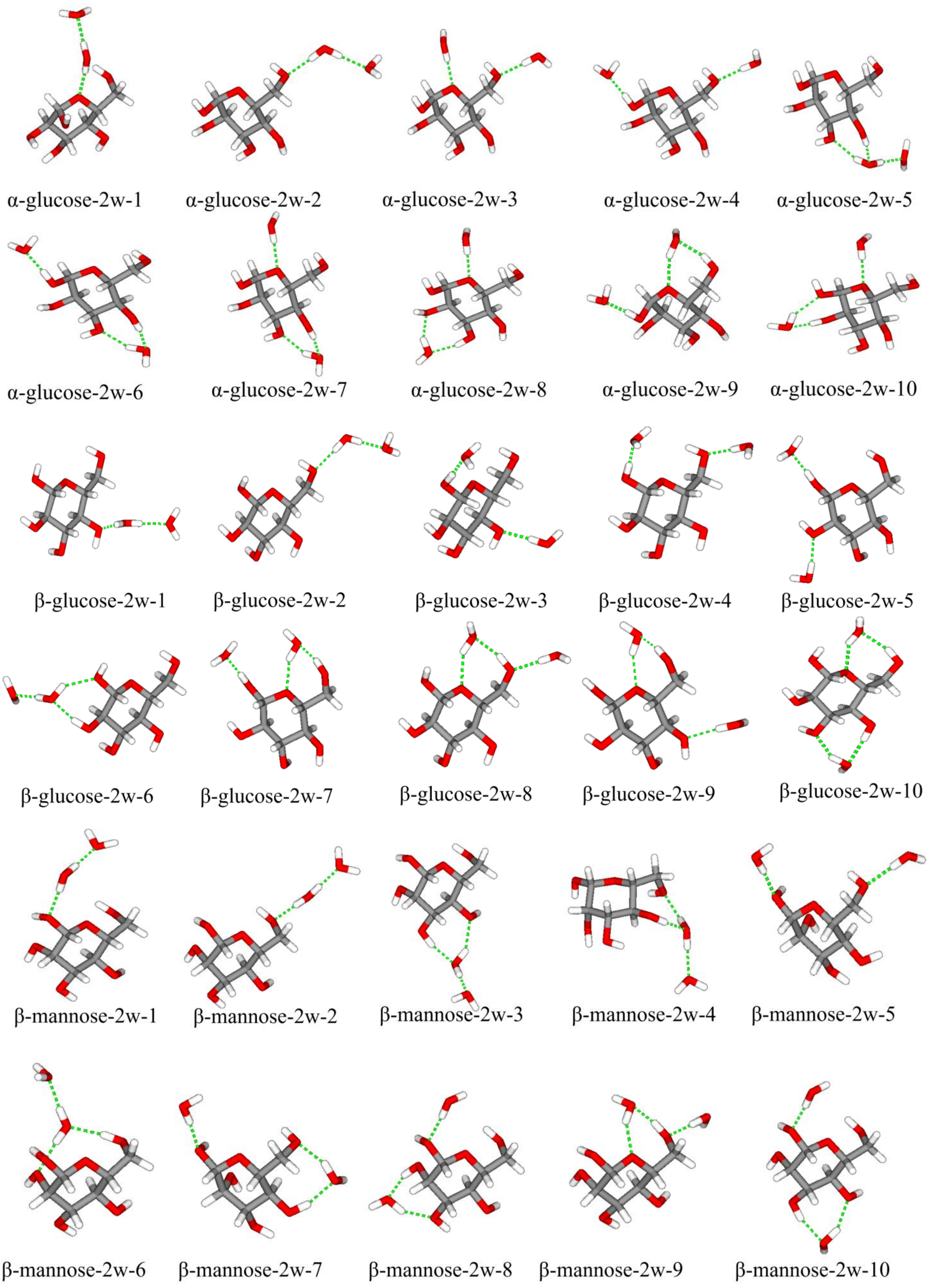


Figure S2 Representative structures of carbohydrate–water–water trimers

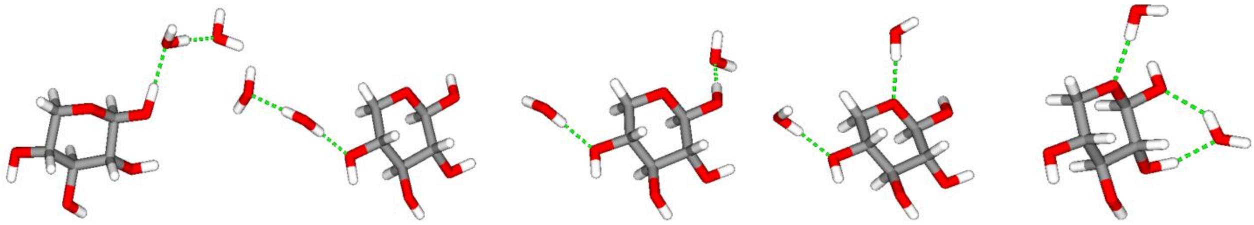

β-xylose-2w-1 β-xylose-2w-2 β-xylose-2w-3 β-xylose-2w-4 β-xylose-2w-5

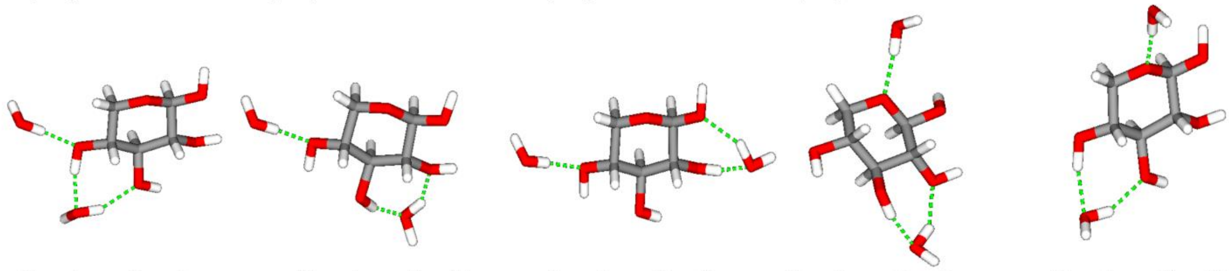

β-xylose-2w-6 β-xylose-2w-7 β-xylose-2w-8 β-xylose-2w-9 β-xylose-2w-10

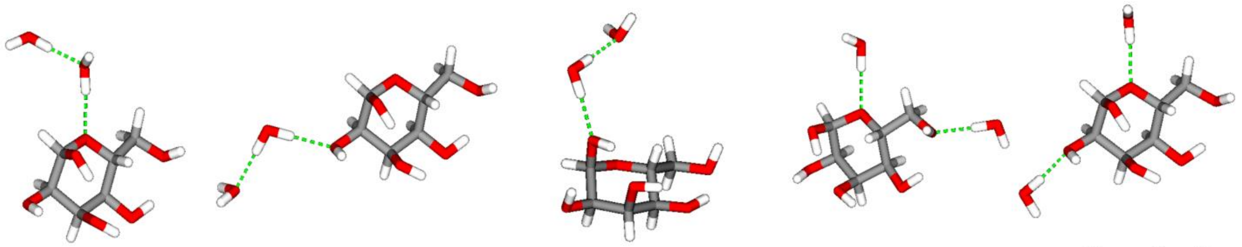

α-allose-2w-1 α-allose-2w-2 α-allose-2w-3 α-allose-2w-4 α-allose-2w-5

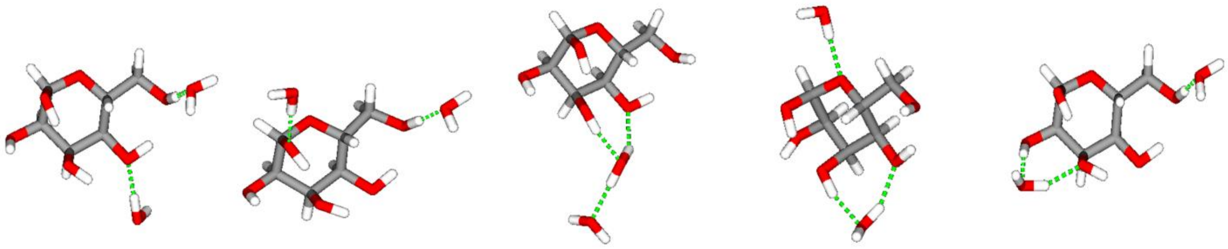

α-allose-2w-6 α-allose-2w-7 α-allose-2w-8 α-allose-2w-9 α-allose-2w-10

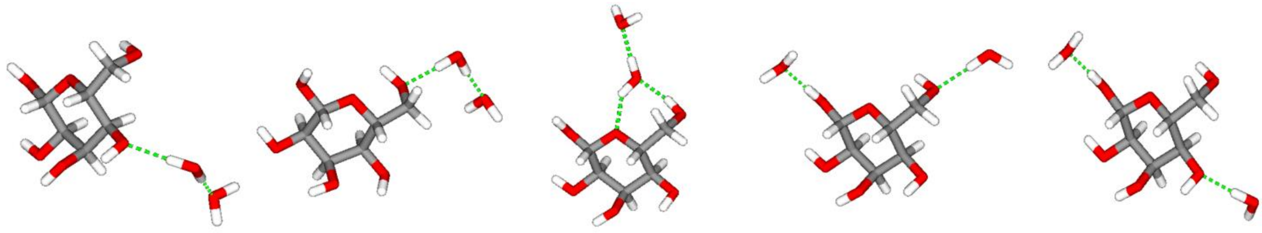

β-allose-2w-1 β-allose-2w-2 β-allose-2w-3 β-allose-2w-4 β-allose-2w-5

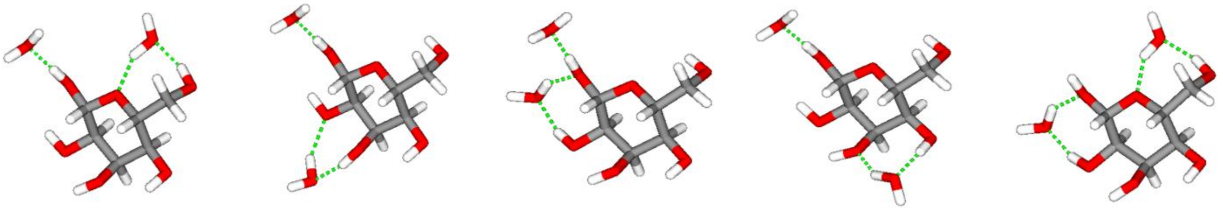

β-allose-2w-6 β-allose-2w-7 β-allose-2w-8 β-allose-2w-9 β-allose-2w-10

Figure S2 Continued

Table S17 The interaction energies (kcal/mol) of carbohydrate-water-water trimers in solution-phase under CPCM implicit solvation

| Trimers | $IE_{2b}$ | | | $IE_{3b}$ | | | $IE_{tot}$ | | |
|---|---|---|---|---|---|---|---|---|---|
| | QM[a] | PBDPC25[b] | Δ | QM[a] | PBDPC25[b] | Δ | QM[a] | PBDPC25[b] | Δ |
| α-glucose-2w-1 | -9.84 | -10.03 | -0.19 | 0.36 | 0.23 | -0.13 | -9.48 | -9.80 | -0.32 |
| α-glucose-2w-2 | -10.31 | -11.33 | -1.02 | 0.49 | 0.54 | 0.05 | -9.82 | -10.79 | -0.97 |
| α-glucose-2w-3 | -10.08 | -9.75 | 0.33 | 0.04 | 0.01 | -0.03 | -10.04 | -9.74 | 0.30 |
| α-glucose-2w-4 | -12.35 | -12.96 | -0.61 | -0.02 | -0.03 | -0.01 | -12.37 | -12.99 | -0.62 |
| α-glucose-2w-5 | -13.66 | -14.63 | -0.97 | -0.15 | 0.03 | 0.18 | -13.81 | -14.60 | -0.79 |
| α-glucose-2w-6 | -15.28 | -16.80 | -1.52 | -0.02 | -0.02 | 0.00 | -15.30 | -16.82 | -1.52 |
| α-glucose-2w-7 | -13.07 | -12.96 | 0.11 | -0.04 | -0.05 | -0.01 | -13.11 | -13.01 | 0.10 |
| α-glucose-2w-8 | -13.02 | -13.04 | -0.02 | 0.02 | -0.04 | -0.06 | -13.00 | -13.08 | -0.08 |
| α-glucose-2w-9 | -14.89 | -16.01 | -1.12 | -0.10 | -0.15 | -0.05 | -14.99 | -16.16 | -1.17 |
| α-glucose-2w-10 | -12.20 | -13.11 | -0.91 | 0.01 | -0.08 | -0.09 | -12.19 | -13.19 | -1.00 |
| β-glucose-2w-1 | -9.98 | -11.42 | -1.44 | 0.43 | 0.40 | -0.03 | -9.55 | -11.02 | -1.47 |
| β-glucose-2w-2 | -10.14 | -11.17 | -1.03 | 0.48 | 0.54 | 0.06 | -9.66 | -10.63 | -0.97 |
| β-glucose-2w-3 | -12.16 | -13.69 | -1.53 | -0.03 | 0.01 | 0.04 | -12.19 | -13.68 | -1.49 |
| β-glucose-2w-4 | -12.47 | -12.80 | -0.33 | -0.03 | -0.06 | -0.03 | -12.50 | -12.86 | -0.36 |
| β-glucose-2w-5 | -12.22 | -13.32 | -1.10 | -0.09 | -0.09 | 0.00 | -12.31 | -13.41 | -1.10 |
| β-glucose-2w-6 | -12.87 | -14.09 | -1.22 | -0.32 | -0.18 | 0.14 | -13.19 | -14.27 | -1.08 |
| β-glucose-2w-7 | -15.43 | -16.68 | -1.25 | -0.10 | -0.16 | -0.06 | -15.53 | -16.84 | -1.31 |
| β-glucose-2w-8 | -13.25 | -13.49 | -0.24 | -0.55 | -0.60 | -0.05 | -13.80 | -14.09 | -0.29 |
| β-glucose-2w-9 | -12.91 | -12.06 | 0.85 | 0.02 | 0.02 | 0.00 | -12.89 | -12.04 | 0.85 |
| β-glucose-2w-10 | -15.85 | -16.35 | -0.50 | -0.01 | 0.03 | 0.04 | -15.86 | -16.32 | -0.46 |
| β-mannose-2w-1 | -9.78 | -11.49 | -1.71 | 0.42 | 0.56 | 0.14 | -9.36 | -10.93 | -1.57 |
| β-mannose-2w-2 | -10.25 | -11.15 | -0.90 | 0.48 | 0.55 | 0.07 | -9.77 | -10.60 | -0.83 |
| β-mannose-2w-3 | -13.72 | -16.43 | -2.71 | -0.22 | -0.28 | -0.06 | -13.94 | -16.71 | -2.77 |
| β-mannose-2w-4 | -15.63 | -18.34 | -2.71 | -0.21 | -0.13 | 0.08 | -15.84 | -18.47 | -2.63 |
| β-mannose-2w-5 | -9.95 | -10.82 | -0.87 | 0.01 | 0.02 | 0.01 | -9.94 | -10.80 | -0.86 |
| β-mannose-2w-6 | -15.21 | -17.37 | -2.16 | -0.06 | 0.03 | 0.09 | -15.27 | -17.34 | -2.07 |
| β-mannose-2w-7 | -14.99 | -17.25 | -2.26 | -0.01 | -0.03 | -0.02 | -15.00 | -17.28 | -2.28 |
| β-mannose-2w-8 | -13.51 | -15.82 | -2.31 | -0.05 | -0.10 | -0.05 | -13.56 | -15.92 | -2.36 |
| β-mannose-2w-9 | -13.76 | -15.49 | -1.73 | -0.57 | -0.61 | -0.04 | -14.33 | -16.10 | -1.77 |
| β-mannose-2w-10 | -13.04 | -15.07 | -2.03 | -0.01 | -0.02 | -0.01 | -13.05 | -15.09 | -2.04 |
| β-xylose-2w-1 | -12.27 | -13.94 | -1.67 | -0.86 | -0.73 | 0.13 | -13.13 | -14.67 | -1.54 |
| β-xylose-2w-2 | -10.02 | -10.30 | -0.28 | 0.45 | 0.57 | 0.12 | -9.57 | -9.73 | -0.16 |
| β-xylose-2w-3 | -11.97 | -13.37 | -1.40 | -0.03 | 0.02 | 0.05 | -12.00 | -13.35 | -1.35 |
| β-xylose-2w-4 | -10.00 | -10.22 | -0.22 | 0.05 | 0.01 | -0.04 | -9.95 | -10.21 | -0.26 |
| β-xylose-2w-5 | -12.75 | -14.71 | -1.96 | 0.01 | -0.02 | -0.03 | -12.74 | -14.73 | -1.99 |
| β-xylose-2w-6 | -13.38 | -14.54 | -1.16 | -0.48 | -0.57 | -0.09 | -13.86 | -15.11 | -1.25 |
| β-xylose-2w-7 | -13.50 | -15.13 | -1.63 | -0.05 | -0.05 | 0.00 | -13.55 | -15.18 | -1.63 |
| β-xylose-2w-8 | -12.87 | -14.59 | -1.72 | -0.03 | -0.02 | 0.01 | -12.90 | -14.61 | -1.71 |
| β-xylose-2w-9 | -13.23 | -14.85 | -1.62 | 0.01 | -0.03 | -0.04 | -13.22 | -14.88 | -1.66 |
| β-xylose-2w-10 | -13.34 | -14.63 | -1.29 | -0.03 | 0.00 | 0.03 | -13.37 | -14.63 | -1.26 |

| | | | | | | | | | |
|---|---|---|---|---|---|---|---|---|---|
| α-allose-2w-1 | -10.20 | -11.08 | -0.88 | -0.49 | -0.25 | 0.24 | -10.69 | -11.33 | -0.64 |
| α-allose-2w-2 | -9.76 | -11.09 | -1.33 | 0.43 | 0.53 | 0.10 | -9.33 | -10.56 | -1.23 |
| α-allose-2w-3 | -9.99 | -11.38 | -1.39 | 0.45 | 0.66 | 0.21 | -9.54 | -10.72 | -1.18 |
| α-allose-2w-4 | -9.62 | -9.47 | 0.15 | 0.05 | -0.02 | -0.07 | -9.57 | -9.49 | 0.08 |
| α-allose-2w-5 | -9.85 | -9.98 | -0.13 | 0.06 | -0.02 | -0.08 | -9.79 | -10.00 | -0.21 |
| α-allose-2w-6 | -12.08 | -13.54 | -1.46 | -0.08 | -0.14 | -0.06 | -12.16 | -13.68 | -1.52 |
| α-allose-2w-7 | -11.71 | -14.11 | -2.40 | -0.01 | -0.03 | -0.02 | -11.72 | -14.14 | -2.42 |
| α-allose-2w-8 | -14.68 | -17.07 | -2.39 | -0.27 | -0.10 | 0.17 | -14.95 | -17.17 | -2.22 |
| α-allose-2w-9 | -14.26 | -16.11 | -1.85 | 0.01 | -0.06 | -0.07 | -14.25 | -16.17 | -1.92 |
| α-allose-2w-10 | -14.13 | -15.88 | -1.75 | 0.00 | 0.00 | 0.00 | -14.13 | -15.88 | -1.75 |
| β-allose-2w-1 | -10.21 | -11.23 | -1.02 | 0.47 | 0.63 | 0.16 | -9.74 | -10.60 | -0.86 |
| β-allose-2w-2 | -10.21 | -10.89 | -0.68 | 0.49 | 0.59 | 0.10 | -9.72 | -10.30 | -0.58 |
| β-allose-2w-3 | -13.39 | -15.40 | -2.01 | -0.15 | -0.14 | 0.01 | -13.54 | -15.54 | -2.00 |
| β-allose-2w-4 | -15.33 | -17.07 | -1.74 | -0.13 | -0.16 | -0.03 | -15.46 | -17.23 | -1.77 |
| β-allose-2w-5 | -16.30 | -17.50 | -1.20 | 0.05 | 0.01 | -0.04 | -16.25 | -17.49 | -1.24 |
| β-allose-2w-6 | -12.21 | -13.78 | -1.57 | -0.02 | -0.03 | -0.01 | -12.23 | -13.81 | -1.58 |
| β-allose-2w-7 | -13.51 | -15.29 | -1.78 | 0.03 | -0.02 | -0.05 | -13.48 | -15.31 | -1.83 |
| β-allose-2w-8 | -13.63 | -13.45 | 0.18 | -0.56 | -0.60 | -0.04 | -14.19 | -14.05 | 0.14 |
| β-allose-2w-9 | -16.65 | -18.43 | -1.78 | 0.02 | 0.01 | -0.01 | -16.63 | -18.42 | -1.79 |
| β-allose-2w-10 | -12.15 | -12.85 | -0.70 | -0.02 | 0.00 | 0.02 | -12.17 | -12.85 | -0.68 |
| RMSE | | | 1.43 | | | 0.08 | | | 1.42 |
| MAE | | | 2.71 | | | 0.24 | | | 2.77 |
| $R^2$ | | | 0.9873 | | | 0.9166 | | | 0.9877 |

[a] QM reference: DLPNO-CCSD(T)/CBS with CPCM implicit solvation model

[b] the gas-phase parameterization and functional form were retained, while AM1 atomic charges were evaluated under CPCM implicit solvation instead of in vacuum

Table S18 Conformational energies (kcal/mol) and Molecular dipole moments (D) of disaccharides

| Monomers | Conformational energies | | | Molecular dipole moments | | |
|---|---|---|---|---|---|---|
| | QM[a] | PBDPC25[c] | Δ | QM[b] | PBDPC25[c] | Δ |
| Lactose01 | 0.00 | 0.00 | 0.00 | 6.18 | 4.70 | -1.48 |
| Lactose02 | 3.47 | 3.54 | 0.07 | 6.24 | 5.33 | -0.91 |
| Lactose03 | 7.36 | 6.99 | -0.37 | 1.58 | 1.27 | -0.31 |
| Lactose04 | 8.12 | 7.40 | -0.72 | 7.67 | 6.33 | -1.34 |
| Lactose05 | 9.07 | 4.07 | -5.00 | 6.57 | 5.01 | -1.56 |
| Lactose06 | 10.04 | 9.82 | -0.22 | 6.18 | 5.28 | -0.90 |
| Lactose07 | 10.31 | 11.56 | 1.25 | 8.38 | 6.91 | -1.47 |
| Lactose08 | 10.87 | 8.09 | -2.78 | 5.45 | 4.76 | -0.69 |
| Lactose09 | 12.75 | 14.04 | 1.29 | 7.19 | 5.66 | -1.53 |
| Lactose10 | 15.1 | 13.94 | -1.16 | 4.12 | 3.13 | -0.99 |
| Maltose01 | 0.00 | 0.00 | 0.00 | 5.22 | 4.34 | -0.88 |
| Maltose02 | 2.17 | 1.50 | -0.67 | 8.26 | 6.51 | -1.75 |
| Maltose03 | 2.95 | 3.54 | 0.59 | 1.57 | 1.38 | -0.19 |
| Maltose04 | 5.00 | 7.68 | 2.68 | 5.82 | 5.12 | -0.70 |
| Maltose05 | 5.64 | 8.22 | 2.58 | 3.94 | 3.66 | -0.28 |
| Maltose06 | 6.84 | 10.64 | 3.80 | 3.41 | 2.60 | -0.81 |
| Maltose07 | 8.06 | 5.89 | -2.17 | 5.85 | 4.63 | -1.22 |
| Maltose08 | 8.5 | 3.90 | -4.60 | 3.46 | 2.38 | -1.08 |
| Maltose09 | 8.59 | 8.20 | -0.39 | 4.27 | 3.49 | -0.78 |
| Maltose10 | 10.89 | 4.01 | -6.88 | 4.97 | 3.82 | -1.15 |
| Sucrose01 | 0.00 | 0.00 | 0.00 | 1.36 | 0.91 | -0.45 |
| Sucrose02 | 2.28 | 5.65 | 3.37 | 6.44 | 6.01 | -0.43 |
| Sucrose03 | 4.06 | -0.02 | -4.08 | 4.40 | 3.89 | -0.51 |
| Sucrose04 | 7.51 | 6.91 | -0.60 | 2.75 | 2.57 | -0.18 |
| Sucrose05 | 7.94 | 5.59 | -2.35 | 6.81 | 5.01 | -1.80 |
| Sucrose06 | 10.42 | 7.73 | -2.69 | 3.15 | 2.57 | -0.58 |
| Sucrose07 | 10.79 | 7.80 | -2.99 | 7.26 | 6.02 | -1.24 |
| Sucrose08 | 11.56 | 8.08 | -3.48 | 2.60 | 1.44 | -1.16 |
| Sucrose09 | 13.18 | 10.13 | -3.05 | 3.81 | 2.42 | -1.39 |
| Sucrose10 | 14.81 | 11.75 | -3.06 | 6.64 | 4.76 | -1.88 |
| RMSE | | | 2.86 | | | 1.10 |
| MAE | | | 6.88 | | | 1.88 |
| $R^2$ | | | 0.9025 | | | 0.9587 |

[a] QM reference: RIJCOSX-DLPNO-CCSD(T)/CBS energies including SMD implicit solvation, taken from *J. Phys. Chem. A* 2020,124,3,582-590

[b] QM reference: B3LYP/aug-cc-pVTZ dipole moments computed using CPCM implicit solvation model.

[c] the gas-phase parameterization and functional form were retained, while AM1 atomic charges were evaluated under CPCM implicit solvation instead of in vacuum

Table S19. Cartesian coordinates (Å) for six carbohydrate–$(H_2O)_{20}$ clusters

| α-glucose-20w | | | | β-mannose-20w | | | |
|---|---|---|---|---|---|---|---|
| C | 0.872978 | -2.893967 | -0.617923 | C | -2.746903 | -0.337758 | 0.321973 |
| C | 0.688021 | -1.892039 | -1.780281 | H | -3.386093 | -0.449763 | -0.561823 |
| C | 1.20186 | -0.495733 | -1.400702 | O | -2.88917 | -1.500152 | 1.105121 |
| C | 0.609022 | -0.05543 | -0.058127 | H | -2.11457 | -1.511006 | 1.720877 |
| C | 0.73261 | -1.138482 | 1.023168 | C | -0.726973 | 0.811712 | -0.566339 |
| C | -0.060855 | -0.802462 | 2.290207 | H | -0.921977 | 0.898649 | -1.64638 |
| O | 2.225992 | -3.191228 | -0.333046 | O | -1.378497 | -0.33971 | -0.03933 |
| O | 1.276119 | -2.354536 | -2.982473 | C | -3.177954 | 0.941239 | 1.043514 |
| O | 2.65187 | -0.556965 | -1.404091 | H | -4.275395 | 0.959913 | 1.064146 |
| O | 1.260292 | 1.152856 | 0.368356 | O | -2.680728 | 0.951119 | 2.389407 |
| O | 0.204597 | -2.385225 | 0.539039 | H | -2.743959 | 1.876926 | 2.681167 |
| O | 0.519469 | 0.336216 | 2.952508 | C | -2.683055 | 2.191962 | 0.309002 |
| H | 2.783339 | -2.492177 | -0.706927 | H | -3.181232 | 2.242113 | -0.668843 |
| H | 2.191134 | -2.036114 | -3.006057 | O | -3.0653 | 3.278965 | 1.148366 |
| H | 2.976079 | 0.363856 | -1.571024 | H | -2.940258 | 4.130745 | 0.680303 |
| H | 1.060057 | 1.276848 | 1.313792 | C | -1.163714 | 2.135254 | 0.096161 |
| H | 1.316007 | 0.01955 | 3.44637 | H | -0.673716 | 2.236284 | 1.070182 |
| H | 0.372285 | -3.839708 | -0.834525 | O | -0.788797 | 3.230992 | -0.756041 |
| H | -0.387713 | -1.814329 | -1.961758 | H | 0.184507 | 3.347225 | -0.770669 |
| H | 0.889289 | 0.209219 | -2.177055 | C | 0.748267 | 0.475164 | -0.327111 |
| H | -0.458212 | 0.13933 | -0.222765 | H | 0.96085 | -0.490482 | -0.799193 |
| H | 1.785872 | -1.273114 | 1.294718 | H | 1.393015 | 1.225295 | -0.784461 |
| H | -0.063252 | -1.670104 | 2.958893 | O | 1.074206 | 0.427674 | 1.060357 |
| H | -1.09035 | -0.543506 | 2.032024 | H | 0.557636 | -0.280334 | 1.506343 |
| O | 5.575682 | 1.831021 | 0.160356 | O | -2.346681 | 5.449258 | -0.449069 |
| H | 5.827432 | 2.318508 | 0.953479 | H | -2.863485 | 5.69531 | -1.225354 |
| H | 5.436144 | 0.896646 | 0.424183 | H | -1.690524 | 4.777517 | -0.739765 |
| O | 4.901124 | -0.882935 | 0.347045 | O | 2.777603 | -3.975646 | 0.167141 |
| H | 5.562211 | -1.309127 | -0.239002 | H | 3.45553 | -3.730847 | 0.833483 |
| H | 4.089043 | -0.77522 | -0.188816 | H | 2.963864 | -3.386058 | -0.597181 |
| O | -5.053453 | 1.156288 | 2.595154 | O | -0.420426 | -2.24667 | -2.906087 |
| H | -4.873409 | 0.226748 | 2.324011 | H | -0.771223 | -2.504669 | -2.013849 |
| H | -5.99397 | 1.305067 | 2.443498 | H | -0.348557 | -3.085868 | -3.381928 |
| O | 6.765769 | -2.024525 | -1.378436 | O | -3.304414 | -4.169341 | -2.700394 |
| H | 7.215004 | -2.865603 | -1.226339 | H | -4.048587 | -4.742503 | -2.484073 |
| H | 7.397191 | -1.447975 | -1.827727 | H | -2.770181 | -4.066462 | -1.882676 |
| O | -1.417408 | 0.556694 | -3.426911 | O | 3.634297 | -2.144361 | -1.754268 |
| H | -1.259302 | 1.524753 | -3.208315 | H | 3.072291 | -1.505165 | -2.225247 |
| H | -1.224254 | 0.443498 | -4.365275 | H | 4.311537 | -1.641356 | -1.262744 |
| O | -6.884146 | -3.546318 | -2.162603 | O | 3.313927 | 5.160092 | 0.748496 |
| H | -6.679804 | -4.459141 | -2.395645 | H | 2.995126 | 5.925784 | 1.240883 |
| H | -6.068621 | -3.186156 | -1.760827 | H | 3.214255 | 4.38715 | 1.333511 |

| | | | | | | | |
|---|---|---|---|---|---|---|---|
| O | -4.708049 | -1.529435 | 1.882995 | O | 5.541441 | 1.297967 | 1.127831 |
| H | -3.816624 | -1.926322 | 1.712006 | H | 6.16618 | 2.029686 | 1.068447 |
| H | -5.166102 | -2.164156 | 2.44891 | H | 4.632841 | 1.69622 | 1.167049 |
| O | 1.435577 | 4.1495 | -2.400017 | O | -0.979662 | -1.80034 | 5.095569 |
| H | 1.240352 | 4.668871 | -1.593999 | H | -0.563109 | -1.317369 | 5.818436 |
| H | 2.150717 | 3.532882 | -2.159853 | H | -1.885526 | -1.422454 | 4.983006 |
| O | -3.762683 | -0.097602 | -2.360433 | O | 4.810719 | -2.985619 | 1.818769 |
| H | -2.91381 | 0.097717 | -2.847149 | H | 5.181954 | -2.294119 | 1.230313 |
| H | -4.469899 | 0.351956 | -2.838596 | H | 4.585159 | -2.543597 | 2.646053 |
| O | -2.604277 | 0.342335 | 0.233484 | O | 1.991344 | 3.508321 | -1.077635 |
| H | -3.099509 | 0.350747 | -0.604491 | H | 2.455685 | 4.341305 | -0.876343 |
| H | -2.716886 | 1.223915 | 0.635324 | H | 2.171151 | 3.202948 | -1.994972 |
| O | 0.33648 | 5.419232 | -0.118462 | O | -4.127266 | 0.683572 | -2.547077 |
| H | -0.564681 | 5.032478 | -0.109474 | H | -4.871398 | 1.034678 | -3.049706 |
| H | 0.64601 | 5.411028 | 0.794609 | H | -3.825503 | -0.115 | -3.040029 |
| O | -1.032555 | 2.512584 | 3.647771 | O | -3.126549 | -1.556755 | -3.808332 |
| H | -0.488198 | 1.706393 | 3.504761 | H | -2.166606 | -1.574338 | -3.650838 |
| H | -1.300093 | 2.507922 | 4.574421 | H | -3.41911 | -2.438263 | -3.501387 |
| O | 2.680786 | -0.831539 | 4.093577 | O | 5.689651 | -1.090977 | -0.066518 |
| H | 2.760105 | -1.152146 | 4.999145 | H | 6.577005 | -1.248808 | -0.412101 |
| H | 3.096526 | -1.510894 | 3.504527 | H | 5.686128 | -0.174938 | 0.320106 |
| O | 3.198048 | 2.107048 | -1.29818 | O | -0.367688 | -1.720833 | 2.419095 |
| H | 2.549065 | 1.991469 | -0.569971 | H | -0.415127 | -1.762804 | 3.401214 |
| H | 4.070627 | 2.218975 | -0.864255 | H | -0.047063 | -2.589621 | 2.101592 |
| O | -1.059884 | 3.082672 | -2.79461 | O | -1.603779 | -3.378881 | -0.681233 |
| H | -0.136431 | 3.433184 | -2.731095 | H | -2.086678 | -2.749438 | -0.102176 |
| H | -1.517381 | 3.392977 | -1.992273 | H | -0.912495 | -3.77421 | -0.09793 |
| O | -2.996163 | 2.748251 | 1.714693 | O | -3.40792 | -0.733469 | 4.455199 |
| H | -3.812403 | 2.313931 | 2.054142 | H | -3.220718 | -0.162076 | 3.675395 |
| H | -2.33029 | 2.709384 | 2.438265 | H | -4.070124 | -1.37128 | 4.161633 |
| O | -4.603218 | -2.35745 | -0.951373 | O | 3.051313 | 2.342239 | 1.215315 |
| H | -4.290342 | -1.62827 | -1.52904 | H | 2.405573 | 1.646892 | 1.472004 |
| H | -4.923823 | -1.926694 | -0.137957 | H | 2.743954 | 2.614293 | 0.323186 |
| O | 3.799241 | -2.563983 | 2.319544 | O | 2.083256 | 2.197117 | -3.503566 |
| H | 3.213233 | -3.095064 | 1.755873 | H | 1.431093 | 2.505571 | -4.144666 |
| H | 4.314458 | -2.010044 | 1.695214 | H | 1.952364 | 1.227861 | -3.434584 |
| O | -2.641555 | -3.072364 | 0.988232 | O | 1.782089 | -0.644032 | -3.4426 |
| H | -1.711405 | -2.827938 | 0.83953 | H | 0.970229 | -1.162458 | -3.219226 |
| H | -3.087725 | -3.023088 | 0.120043 | H | 2.088691 | -0.988666 | -4.291911 |
| O | -2.228662 | 4.28276 | -0.375661 | O | 0.357107 | -4.135847 | 1.087913 |
| H | -2.890545 | 4.957518 | -0.571974 | H | 0.298566 | -4.99422 | 1.525671 |
| H | -2.580901 | 3.753391 | 0.384744 | H | 1.299405 | -4.067084 | 0.720407 |

| | β-glucose-20w | | | | α-allose-20w | | |
|---|---|---|---|---|---|---|---|
| C | 0.740249 | -1.331883 | -1.652735 | C | 0.872978 | -2.893967 | -0.617923 |
| C | -0.289587 | -1.271399 | -0.525604 | C | 0.688021 | -1.892039 | -1.780281 |
| C | -1.488558 | -0.388113 | -0.868386 | C | 1.20186 | -0.495733 | -1.400702 |
| C | -1.013408 | 0.968094 | -1.419226 | C | 0.609022 | -0.05543 | -0.058127 |
| C | 0.029323 | 0.790697 | -2.537161 | C | 0.73261 | -1.138482 | 1.023168 |
| C | 0.649949 | 2.109642 | -2.997244 | C | -0.060855 | -0.802462 | 2.290207 |
| O | 1.883697 | -1.935272 | -1.095938 | O | 2.225992 | -3.191228 | -0.333046 |
| O | -0.726417 | -2.583597 | -0.169373 | O | 1.276119 | -2.354536 | -2.982473 |
| O | -2.343144 | -0.975622 | -1.848985 | O | 2.65187 | -0.556965 | -1.404091 |
| O | -2.146582 | 1.69097 | -1.912449 | O | 1.260292 | 1.152856 | 0.368356 |
| O | 1.112077 | -0.031792 | -2.072433 | O | 0.204597 | -2.385225 | 0.539039 |
| O | 1.356013 | 2.778868 | -1.963869 | O | 0.519469 | 0.336216 | 2.952508 |
| H | 2.527869 | -2.212974 | -1.770296 | H | 2.783339 | -2.492177 | -0.706927 |
| H | 0.000664 | -3.040496 | 0.29552 | H | 2.191134 | -2.036114 | -3.006057 |
| H | -3.092107 | -1.377775 | -1.360013 | H | 2.976079 | 0.363856 | -1.571024 |
| H | -2.699145 | 1.021158 | -2.35593 | H | 1.060057 | 1.276848 | 1.313792 |
| H | 2.257617 | 2.384047 | -1.841397 | H | 1.316007 | 0.01955 | 3.44637 |
| H | 0.363984 | -1.918607 | -2.504687 | H | 0.372285 | -3.839708 | -0.834525 |
| H | 0.211289 | -0.801594 | 0.329628 | H | -0.387713 | -1.814329 | -1.961758 |
| H | -2.056112 | -0.214357 | 0.050652 | H | 0.889289 | 0.209219 | -2.177055 |
| H | -0.572962 | 1.557253 | -0.611881 | H | -0.458212 | 0.13933 | -0.222765 |
| H | -0.455334 | 0.314113 | -3.405662 | H | 1.785872 | -1.273114 | 1.294718 |
| H | -0.154529 | 2.770005 | -3.333631 | H | -0.063252 | -1.670104 | 2.958893 |
| H | 1.316985 | 1.910501 | -3.846586 | H | -1.09035 | -0.543506 | 2.032024 |
| O | -3.153077 | 1.622095 | 2.415726 | O | 5.575682 | 1.831021 | 0.160356 |
| H | -3.497987 | 2.179464 | 3.123506 | H | 5.827432 | 2.318508 | 0.953479 |
| H | -3.504495 | 1.998258 | 1.546201 | H | 5.436144 | 0.896646 | 0.424183 |
| O | -0.444242 | 1.050036 | 2.034593 | O | 4.901124 | -0.882935 | 0.347045 |
| H | -1.340049 | 1.397501 | 2.227595 | H | 5.562211 | -1.309127 | -0.239002 |
| H | -0.439153 | 0.131366 | 2.369822 | H | 4.089043 | -0.77522 | -0.188816 |
| O | 3.897168 | 1.987287 | -1.51405 | O | -5.053453 | 1.156288 | 2.595154 |
| H | 4.268536 | 1.145152 | -1.836978 | H | -4.873409 | 0.226748 | 2.324011 |
| H | 4.001015 | 1.959678 | -0.543178 | H | -5.99397 | 1.305067 | 2.443498 |
| O | 1.885133 | 2.300764 | 2.679553 | O | 6.765769 | -2.024525 | -1.378436 |
| H | 1.763717 | 3.277796 | 2.595089 | H | 7.215004 | -2.865603 | -1.226339 |
| H | 1.022101 | 1.903039 | 2.400523 | H | 7.397191 | -1.447975 | -1.827727 |
| O | 2.165935 | -0.43395 | 3.81583 | O | -1.417408 | 0.556694 | -3.426911 |
| H | 2.124886 | 0.538223 | 3.829994 | H | -1.259302 | 1.524753 | -3.208315 |
| H | 2.546448 | -0.648342 | 2.94285 | H | -1.224254 | 0.443498 | -4.365275 |
| O | 5.284757 | -0.431974 | -2.204406 | O | -6.884146 | -3.546318 | -2.162603 |
| H | 5.040043 | -1.37418 | -2.13693 | H | -6.679804 | -4.459141 | -2.395645 |
| H | 6.051256 | -0.378191 | -2.786259 | H | -6.068621 | -3.186156 | -1.760827 |

| | | | | | | | |
|---|---|---|---|---|---|---|---|
| O | -3.966476 | 2.567836 | 0.126557 | O | -4.708049 | -1.529435 | 1.882995 |
| H | -3.321313 | 2.402671 | -0.58989 | H | -3.816624 | -1.926322 | 1.712006 |
| H | -4.84001 | 2.253012 | -0.203231 | H | -5.166102 | -2.164156 | 2.44891 |
| O | -0.262652 | -1.585052 | 3.125813 | O | 1.435577 | 4.1495 | -2.400017 |
| H | 0.513271 | -1.23013 | 3.618259 | H | 1.240352 | 4.668871 | -1.593999 |
| H | 0.106893 | -2.31806 | 2.605041 | H | 2.150717 | 3.532882 | -2.159853 |
| O | 4.02751 | 1.640465 | 1.302445 | O | -3.762683 | -0.097602 | -2.360433 |
| H | 3.203137 | 1.938545 | 1.802136 | H | -2.91381 | 0.097717 | -2.847149 |
| H | 4.788074 | 1.997099 | 1.777576 | H | -4.469899 | 0.351956 | -2.838596 |
| O | -6.395223 | 1.644258 | -0.784334 | O | -2.604277 | 0.342335 | 0.233484 |
| H | -7.190084 | 2.051861 | -0.421705 | H | -3.099509 | 0.350747 | -0.604491 |
| H | -6.533962 | 0.666223 | -0.738565 | H | -2.716886 | 1.223915 | 0.635324 |
| O | -2.854813 | -4.287225 | -0.78838 | O | 0.33648 | 5.419232 | -0.118462 |
| H | -2.690341 | -5.113051 | -0.319295 | H | -0.564681 | 5.032478 | -0.109474 |
| H | -2.043046 | -3.741318 | -0.700285 | H | 0.64601 | 5.411028 | 0.794609 |
| O | 3.957145 | -3.475897 | 0.939672 | O | -1.032555 | 2.512584 | 3.647771 |
| H | 4.621621 | -3.826492 | 1.546445 | H | -0.488198 | 1.706393 | 3.504761 |
| H | 3.883213 | -2.493959 | 1.122221 | H | -1.300093 | 2.507922 | 4.574421 |
| O | -3.256722 | -1.203896 | 2.403123 | O | 2.680786 | -0.831539 | 4.093577 |
| H | -2.370558 | -1.445809 | 2.720918 | H | 2.760105 | -1.152146 | 4.999145 |
| H | -3.319487 | -0.231112 | 2.500771 | H | 3.096526 | -1.510894 | 3.504527 |
| O | 1.579449 | 4.948884 | 2.106051 | O | 3.198048 | 2.107048 | -1.29818 |
| H | 0.898495 | 5.494044 | 2.516969 | H | 2.549065 | 1.991469 | -0.569971 |
| H | 1.430988 | 5.00968 | 1.128437 | H | 4.070627 | 2.218975 | -0.864255 |
| O | 3.283353 | -0.957462 | 1.153211 | O | -1.059884 | 3.082672 | -2.79461 |
| H | 2.730467 | -1.022932 | 0.345721 | H | -0.136431 | 3.433184 | -2.731095 |
| H | 3.729843 | -0.07667 | 1.132546 | H | -1.517381 | 3.392977 | -1.992273 |
| O | -6.69904 | -1.058563 | -0.654478 | O | -2.996163 | 2.748251 | 1.714693 |
| H | -6.985958 | -1.498963 | -1.46317 | H | -3.812403 | 2.313931 | 2.054142 |
| H | -5.84182 | -1.481161 | -0.39888 | H | -2.33029 | 2.709384 | 2.438265 |
| O | 4.333623 | -3.104266 | -1.811359 | O | -4.603218 | -2.35745 | -0.951373 |
| H | 4.300109 | -3.381676 | -0.867122 | H | -4.290342 | -1.62827 | -1.52904 |
| H | 4.525511 | -3.8913 | -2.335268 | H | -4.923823 | -1.926694 | -0.137957 |
| O | -4.234411 | -2.037728 | -0.041585 | O | 3.799241 | -2.563983 | 2.319544 |
| H | -3.89533 | -1.779339 | 0.851914 | H | 3.213233 | -3.095064 | 1.755873 |
| H | -3.966265 | -2.971259 | -0.180729 | H | 4.314458 | -2.010044 | 1.695214 |
| O | 1.170116 | 5.106304 | -0.576604 | O | -2.641555 | -3.072364 | 0.988232 |
| H | 1.773428 | 5.685023 | -1.057699 | H | -1.711405 | -2.827938 | 0.83953 |
| H | 1.15254 | 4.2603 | -1.087843 | H | -3.087725 | -3.023088 | 0.120043 |
| O | 1.146281 | -3.809559 | 1.582969 | O | -2.228662 | 4.28276 | -0.375661 |
| H | 2.098916 | -3.773486 | 1.352781 | H | -2.890545 | 4.957518 | -0.571974 |
| H | 0.993657 | -4.672317 | 1.987727 | H | -2.580901 | 3.753391 | 0.384744 |

| β-xylose-20w | | | | β-allose-20w | | | |
|---|---|---|---|---|---|---|---|
| C | -0.408441 | -0.397754 | -0.67346 | C | 0.740249 | -1.331883 | -1.652735 |
| H | -0.176435 | -1.425986 | -0.355361 | C | -0.289587 | -1.271399 | -0.525604 |
| O | -0.896407 | -0.401734 | -1.984121 | C | -1.488558 | -0.388113 | -0.868386 |
| H | -1.366108 | -1.260776 | -2.129018 | C | -1.013408 | 0.968094 | -1.419226 |
| C | -0.9674 | 0.046073 | 1.566374 | C | 0.029323 | 0.790697 | -2.537161 |
| H | -0.836383 | -1.010427 | 1.834841 | C | 0.649949 | 2.109642 | -2.997244 |
| H | -1.774144 | 0.464959 | 2.16799 | O | 1.883697 | -1.935272 | -1.095938 |
| O | -1.380379 | 0.157119 | 0.19951 | O | -0.726417 | -2.583597 | -0.169373 |
| C | 0.857852 | 0.45918 | -0.632477 | O | -2.343144 | -0.975622 | -1.848985 |
| H | 0.59588 | 1.490491 | -0.902472 | O | -2.146582 | 1.69097 | -1.912449 |
| O | 1.819508 | -0.059817 | -1.558921 | O | 1.112077 | -0.031792 | -2.072433 |
| H | 1.34806 | -0.252768 | -2.384547 | O | 1.356013 | 2.778868 | -1.963869 |
| C | 1.419715 | 0.404663 | 0.788469 | H | 2.527869 | -2.212974 | -1.770296 |
| H | 1.722867 | -0.630454 | 0.999048 | H | 0.000664 | -3.040496 | 0.29552 |
| O | 2.537491 | 1.272346 | 1.002934 | H | -3.092107 | -1.377775 | -1.360013 |
| H | 3.329507 | 0.86145 | 0.591552 | H | -2.699145 | 1.021158 | -2.35593 |
| C | 0.339612 | 0.811058 | 1.797694 | H | 2.257617 | 2.384047 | -1.841397 |
| H | 0.14613 | 1.886378 | 1.70146 | H | 0.363984 | -1.918607 | -2.504687 |
| O | 0.777137 | 0.519808 | 3.13163 | H | 0.211289 | -0.801594 | 0.329628 |
| H | 1.613284 | 0.990909 | 3.264175 | H | -2.056112 | -0.214357 | 0.050652 |
| O | 5.933716 | 1.848471 | -1.702303 | H | -0.572962 | 1.557253 | -0.611881 |
| H | 6.784525 | 2.131655 | -1.348244 | H | -0.455334 | 0.314113 | -3.405662 |
| H | 5.378555 | 2.668255 | -1.792921 | H | -0.154529 | 2.770005 | -3.333631 |
| O | -4.536722 | -1.105119 | -2.388175 | H | 1.316985 | 1.910501 | -3.846586 |
| H | -4.108085 | -0.207423 | -2.528238 | O | -3.153077 | 1.622095 | 2.415726 |
| H | -5.296283 | -1.152986 | -2.981247 | H | -3.497987 | 2.179464 | 3.123506 |
| O | -4.157707 | 2.998977 | 1.412149 | H | -3.504495 | 1.998258 | 1.546201 |
| H | -4.290721 | 2.028405 | 1.632151 | O | -0.444242 | 1.050036 | 2.034593 |
| H | -4.983932 | 3.44082 | 1.644503 | H | -1.340049 | 1.397501 | 2.227595 |
| O | -0.245607 | -3.684363 | 1.337156 | H | -0.439153 | 0.131366 | 2.369822 |
| H | 0.475757 | -3.80503 | 0.677108 | O | 3.897168 | 1.987287 | -1.51405 |
| H | 0.152483 | -3.258659 | 2.132534 | H | 4.268536 | 1.145152 | -1.836978 |
| O | -4.472013 | 0.468268 | 2.0556 | H | 4.001015 | 1.959678 | -0.543178 |
| H | -4.959892 | 0.193146 | 2.84007 | O | 1.885133 | 2.300764 | 2.679553 |
| H | -4.522162 | -0.293109 | 1.406138 | H | 1.763717 | 3.277796 | 2.595089 |
| O | -3.144621 | 1.109823 | -2.743745 | H | 1.022101 | 1.903039 | 2.400523 |
| H | -3.254926 | 1.923948 | -2.212582 | O | 2.165935 | -0.43395 | 3.81583 |
| H | -2.267706 | 0.75842 | -2.486421 | H | 2.124886 | 0.538223 | 3.829994 |
| O | 4.380457 | 4.033571 | -1.893473 | H | 2.546448 | -0.648342 | 2.94285 |
| H | 4.061814 | 4.303336 | -2.762279 | O | 5.284757 | -0.431974 | -2.204406 |
| H | 3.591954 | 4.038224 | -1.285317 | H | 5.040043 | -1.37418 | -2.13693 |
| O | 4.280896 | -2.67343 | 0.041137 | H | 6.051256 | -0.378191 | -2.786259 |

| | | | | | | | |
|---|---|---|---|---|---|---|---|
| H | 4.986718 | -3.210916 | -0.338973 | O | -3.966476 | 2.567836 | 0.126557 |
| H | 4.452865 | -1.733583 | -0.228837 | H | -3.321313 | 2.402671 | -0.58989 |
| O | -2.617978 | -3.586883 | 0.332982 | H | -4.84001 | 2.253012 | -0.203231 |
| H | -1.708724 | -3.589317 | 0.78448 | O | -0.262652 | -1.585052 | 3.125813 |
| H | -2.987707 | -4.465376 | 0.487701 | H | 0.513271 | -1.23013 | 3.618259 |
| O | -2.254045 | -2.744698 | -2.250327 | H | 0.106893 | -2.31806 | 2.605041 |
| H | -2.383628 | -3.1262 | -1.346394 | O | 4.02751 | 1.640465 | 1.302445 |
| H | -3.11668 | -2.345923 | -2.492997 | H | 3.203137 | 1.938545 | 1.802136 |
| O | 2.339857 | 3.886807 | -0.166647 | H | 4.788074 | 1.997099 | 1.777576 |
| H | 2.382218 | 3.025399 | 0.291868 | O | -6.395223 | 1.644258 | -0.784334 |
| H | 1.387535 | 4.081762 | -0.294045 | H | -7.190084 | 2.051861 | -0.421705 |
| O | 4.372463 | -0.068085 | -0.627658 | H | -6.533962 | 0.666223 | -0.738565 |
| H | 3.593073 | -0.047946 | -1.222332 | O | -2.854813 | -4.287225 | -0.78838 |
| H | 5.036066 | 0.567573 | -1.001638 | H | -2.690341 | -5.113051 | -0.319295 |
| O | 1.042855 | -2.402615 | 3.41514 | H | -2.043046 | -3.741318 | -0.700285 |
| H | 0.956214 | -1.432928 | 3.451601 | O | 3.957145 | -3.475897 | 0.939672 |
| H | 1.997082 | -2.584122 | 3.291636 | H | 4.621621 | -3.826492 | 1.546445 |
| O | 1.669983 | -3.894152 | -0.63279 | H | 3.883213 | -2.493959 | 1.122221 |
| H | 2.511281 | -3.412732 | -0.54482 | O | -3.256722 | -1.203896 | 2.403123 |
| H | 1.308481 | -3.736469 | -1.527176 | H | -2.370558 | -1.445809 | 2.720918 |
| O | -0.436459 | 4.284066 | -0.221949 | H | -3.319487 | -0.231112 | 2.500771 |
| H | -0.765335 | 4.250961 | 0.698226 | O | 1.579449 | 4.948884 | 2.106051 |
| H | -1.183015 | 3.962901 | -0.754495 | H | 0.898495 | 5.494044 | 2.516969 |
| O | -4.67136 | -1.607741 | 0.426554 | H | 1.430988 | 5.00968 | 1.128437 |
| H | -3.937802 | -2.250431 | 0.513452 | O | 3.283353 | -0.957462 | 1.153211 |
| H | -4.722878 | -1.396859 | -0.527169 | H | 2.730467 | -1.022932 | 0.345721 |
| O | -1.626311 | 3.854024 | 2.302289 | H | 3.729843 | -0.07667 | 1.132546 |
| H | -2.557343 | 3.596515 | 2.141798 | O | -6.69904 | -1.058563 | -0.654478 |
| H | -1.607015 | 4.383291 | 3.107799 | H | -6.985958 | -1.498963 | -1.46317 |
| O | -3.173778 | 3.451919 | -1.165014 | H | -5.84182 | -1.481161 | -0.39888 |
| H | -3.566452 | 3.292092 | -0.276003 | O | 4.333623 | -3.104266 | -1.811359 |
| H | -3.609304 | 4.235273 | -1.523714 | H | 4.300109 | -3.381676 | -0.867122 |
| O | 3.739741 | -2.87588 | 2.786802 | H | 4.525511 | -3.8913 | -2.335268 |
| H | 4.523645 | -2.63799 | 3.294023 | O | -4.234411 | -2.037728 | -0.041585 |
| H | 3.983746 | -2.828279 | 1.838048 | H | -3.89533 | -1.779339 | 0.851914 |
| O | 0.2897 | -3.608474 | -3.109519 | H | -3.966265 | -2.971259 | -0.180729 |
| H | 0.305149 | -4.403785 | -3.654909 | O | 1.170116 | 5.106304 | -0.576604 |
| H | -0.653615 | -3.392672 | -2.963577 | H | 1.773428 | 5.685023 | -1.057699 |
| | | | | H | 1.15254 | 4.2603 | -1.087843 |
| | | | | O | 1.146281 | -3.809559 | 1.582969 |
| | | | | H | 2.098916 | -3.773486 | 1.352781 |
| | | | | H | 0.993657 | -4.672317 | 1.987727 |

Table S20. Cartesian coordinates (Å) of 60 carbohydrate-water-water trimers.

| | | | | | | | |
|---|---|---|---|---|---|---|---|
| | α-glucose-2w-1 | | | | β-glucose-2w-1 | | |
| C | -0.942469 | 2.050905 | 0.719375 | C | -1.010905 | 1.81391 | 0.381034 |
| C | 0.085551 | 0.924669 | 0.733244 | C | 0.047637 | 0.882999 | -0.197193 |
| C | 1.317347 | 1.204136 | -0.135827 | C | -0.072818 | -0.567444 | 0.286445 |
| C | 2.219873 | -0.025175 | -0.186140 | C | 1.104441 | -1.403352 | -0.237469 |
| C | 1.432767 | -1.249605 | -0.665376 | C | 2.43948 | -0.752575 | 0.124801 |
| C | 0.176992 | -1.450536 | 0.195171 | C | 2.431253 | 0.714722 | -0.330551 |
| O | -0.597135 | -0.255687 | 0.242495 | O | 1.316151 | 1.401159 | 0.23621 |
| O | 0.616170 | -1.858246 | 1.472589 | O | 3.604236 | 1.305624 | 0.142603 |
| O | 2.244812 | -2.419864 | -0.677568 | O | 3.484519 | -1.47388 | -0.514338 |
| O | 3.297637 | 0.282152 | -1.072944 | O | 0.982606 | -2.756731 | 0.201989 |
| O | 1.999663 | 2.321669 | 0.432969 | O | -1.305332 | -1.122857 | -0.182869 |
| O | -1.420660 | 2.360434 | -0.589503 | O | -0.872379 | 3.142404 | -0.11981 |
| H | -0.475481 | 2.960233 | 1.102856 | H | -0.94528 | 1.80229 | 1.477954 |
| H | -1.773440 | 1.780886 | 1.383576 | H | -2.004846 | 1.470798 | 0.088479 |
| H | 0.402052 | 0.757053 | 1.770474 | H | -0.007974 | 0.909703 | -1.296241 |
| H | 0.988424 | 1.444799 | -1.156133 | H | -0.060604 | -0.574735 | 1.386718 |
| H | 2.614580 | -0.215303 | 0.821927 | H | 1.029865 | -1.462237 | -1.329826 |
| H | 1.119111 | -1.087032 | -1.700732 | H | 2.577629 | -0.757553 | 1.217057 |
| H | -0.487585 | -2.196701 | -0.248643 | H | 2.37327 | 0.772083 | -1.428368 |
| H | -0.110561 | -2.281347 | 1.951459 | H | 3.778653 | 2.121814 | -0.347504 |
| H | 2.330747 | -2.724549 | 0.240311 | H | 4.321796 | -1.04451 | -0.284061 |
| H | 3.906331 | -0.470863 | -1.081154 | H | 1.158198 | -2.806953 | 1.154352 |
| H | 2.834167 | 2.426379 | -0.047859 | H | -1.21971 | -2.088787 | -0.12157 |
| H | -1.839148 | 1.567807 | -0.954784 | H | 0.025028 | 3.440812 | 0.08965 |
| O | -3.359775 | -0.986025 | 0.977512 | O | -3.868373 | -0.689597 | 1.118109 |
| H | -3.917464 | -0.810459 | 0.194787 | H | -4.537016 | -0.562881 | 0.417931 |
| H | -2.464379 | -0.691644 | 0.738668 | H | -3.014633 | -0.796276 | 0.660798 |
| O | -5.111476 | -0.523161 | -1.219762 | O | -5.867247 | -0.310982 | -0.891409 |
| H | -4.912518 | -0.953319 | -2.062919 | H | -6.522186 | -1.019391 | -0.960765 |
| H | -5.309102 | 0.397503 | -1.440818 | H | -6.380164 | 0.505142 | -0.810886 |
| | α-glucose-2w-2 | | | | β-glucose-2w-2 | | |
| C | -1.282802 | -0.896313 | 0.902713 | C | -1.365413 | 0.430361 | -0.572499 |
| C | 0.192721 | -0.536159 | 0.789248 | C | -0.011592 | 0.235229 | 0.094369 |
| C | 0.452076 | 0.905380 | 0.333907 | C | 1.019742 | 1.297032 | -0.306139 |
| C | 1.947186 | 1.116692 | 0.110380 | C | 2.395973 | 0.957755 | 0.288746 |
| C | 2.495107 | 0.079361 | -0.876398 | C | 2.806405 | -0.475085 | -0.05471 |
| C | 2.137693 | -1.342350 | -0.417500 | C | 1.673765 | -1.442667 | 0.319781 |
| O | 0.746685 | -1.459975 | -0.170352 | O | 0.461348 | -1.05423 | -0.325421 |
| O | 2.917053 | -1.607530 | 0.732320 | O | 2.036037 | -2.710037 | -0.140328 |
| O | 3.900050 | 0.230861 | -1.061630 | O | 3.994855 | -0.790785 | 0.660037 |
| O | 2.117378 | 2.446927 | -0.383950 | O | 3.360491 | 1.935849 | -0.103493 |
| O | -0.048195 | 1.776969 | 1.347950 | O | 0.568509 | 2.563242 | 0.164825 |
| O | -1.965551 | -0.798500 | -0.353547 | O | -2.296736 | -0.574654 | -0.155607 |
| H | -1.778619 | -0.202078 | 1.583237 | H | -1.246129 | 0.412144 | -1.663755 |
| H | -1.371789 | -1.912576 | 1.305216 | H | -1.787984 | 1.39207 | -0.278298 |
| H | 0.660335 | -0.692394 | 1.770330 | H | -0.141578 | 0.24408 | 1.187485 |
| H | -0.077273 | 1.086530 | -0.611892 | H | 1.099631 | 1.310204 | -1.404673 |
| H | 2.465839 | 1.013150 | 1.074135 | H | 2.334597 | 1.052923 | 1.379242 |
| H | 2.039864 | 0.249255 | -1.856623 | H | 2.971809 | -0.568093 | -1.139277 |
| H | 2.345315 | -2.072343 | -1.204806 | H | 1.51949 | -1.445619 | 1.409795 |
| H | 2.943892 | -2.561269 | 0.893903 | H | 1.509014 | -3.38418 | 0.312099 |
| H | 4.337913 | -0.160051 | -0.288208 | H | 4.238629 | -1.701808 | 0.438581 |
| H | 3.065834 | 2.606679 | -0.496657 | H | 3.562005 | 1.830227 | -1.04583 |

| | | | | | | | |
|---|---|---|---|---|---|---|---|
| H | 0.216742 | 2.678560 | 1.112621 | H | 1.297249 | 3.191855 | 0.046091 |
| H | -1.507452 | -1.373679 | -0.985154 | H | -1.897128 | -1.439659 | -0.334804 |
| O | -4.784394 | -1.283712 | -0.337814 | O | -4.992726 | -0.410044 | -1.097395 |
| H | -3.828132 | -1.086121 | -0.344491 | H | -5.557236 | -0.206695 | -0.327712 |
| H | -5.240903 | -0.422551 | -0.386395 | H | -4.078419 | -0.455753 | -0.757752 |
| O | -6.185889 | 1.213568 | -0.486497 | O | -6.684233 | 0.190217 | 1.138472 |
| H | -6.684287 | 1.457711 | 0.305717 | H | -7.161467 | 1.029887 | 1.086407 |
| H | -6.816257 | 1.282654 | -1.216878 | H | -7.356123 | -0.473781 | 1.346405 |
| | α-glucose-2w-3 | | | | β-glucose-2w-3 | | |
| C | 1.932051 | -0.042982 | 0.785948 | C | -1.025247 | -2.165751 | -1.051773 |
| C | 0.409570 | -0.018164 | 0.751280 | C | -0.560282 | -0.920923 | -0.305656 |
| C | -0.212272 | -1.312561 | 0.212021 | C | -1.353032 | 0.339161 | -0.674672 |
| C | -1.723574 | -1.154180 | 0.072776 | C | -0.747056 | 1.581253 | -0.003617 |
| C | -2.057305 | 0.061879 | -0.798160 | C | 0.751946 | 1.676464 | -0.286785 |
| C | -1.364550 | 1.318954 | -0.254003 | C | 1.436162 | 0.345704 | 0.062597 |
| O | 0.036399 | 1.092971 | -0.100336 | O | 0.813147 | -0.720308 | -0.666290 |
| O | -1.992021 | 1.641057 | 0.966112 | O | 2.766746 | 0.433685 | -0.316821 |
| O | -3.464364 | 0.252673 | -0.907455 | O | 1.297228 | 2.739139 | 0.486185 |
| O | -2.220625 | -2.361586 | -0.506588 | O | -1.479270 | 2.750450 | -0.376913 |
| O | 0.116935 | -2.357114 | 1.126674 | O | -2.713656 | 0.167825 | -0.262813 |
| O | 2.510551 | -0.286100 | -0.502643 | O | -0.280857 | -3.319368 | -0.664325 |
| H | 2.260681 | -0.860102 | 1.430084 | H | -0.940037 | -1.993122 | -2.133848 |
| H | 2.302385 | 0.902144 | 1.199606 | H | -2.070579 | -2.370317 | -0.812789 |
| H | 0.045401 | 0.164120 | 1.770058 | H | -0.635089 | -1.099201 | 0.778496 |
| H | 0.214342 | -1.530219 | -0.776674 | H | -1.320258 | 0.465776 | -1.766756 |
| H | -2.158545 | -1.017383 | 1.072962 | H | -0.894545 | 1.497256 | 1.079314 |
| H | -1.695367 | -0.119064 | -1.814493 | H | 0.920777 | 1.857019 | -1.360037 |
| H | -1.436235 | 2.146096 | -0.965165 | H | 1.339599 | 0.139851 | 1.140985 |
| H | -1.820128 | 2.566278 | 1.191922 | H | 3.306635 | -0.213530 | 0.192109 |
| H | -3.770993 | 0.647969 | -0.075302 | H | 2.254997 | 2.738913 | 0.337586 |
| H | -3.184170 | -2.290196 | -0.572017 | H | -1.279709 | 2.976714 | -1.298244 |
| H | -0.360219 | -3.151609 | 0.844524 | H | -3.165601 | 1.016210 | -0.397548 |
| H | 2.259950 | 0.434819 | -1.098870 | H | 0.656027 | -3.123325 | -0.813647 |
| O | 1.437180 | 3.635884 | 0.051133 | O | 4.360560 | -1.312822 | 1.076222 |
| H | 1.621726 | 3.893566 | -0.861675 | H | 4.151797 | -2.255913 | 1.020119 |
| H | 0.984493 | 2.774595 | -0.009753 | H | 4.492019 | -1.129676 | 2.017111 |
| O | 5.236569 | -0.992126 | -0.676442 | O | -3.598859 | -0.897626 | 2.240145 |
| H | 5.261799 | -1.920798 | -0.940757 | H | -3.879451 | -1.806470 | 2.071448 |
| H | 4.286375 | -0.763160 | -0.617264 | H | -3.271843 | -0.563436 | 1.382007 |
| | α-glucose-2w-4 | | | | β-glucose-2w-4 | | |
| C | 2.064625 | 0.687872 | 0.699403 | C | -1.802551 | -1.351668 | 0.992651 |
| C | 0.625546 | 0.192482 | 0.642211 | C | -0.623197 | -0.810297 | 0.196919 |
| C | 0.493547 | -1.309633 | 0.360818 | C | 0.614373 | -1.713674 | 0.259947 |
| C | -0.977892 | -1.671344 | 0.182386 | C | 1.802146 | -1.042171 | -0.447310 |
| C | -1.619481 | -0.806944 | -0.908719 | C | 2.029800 | 0.367819 | 0.096037 |
| C | -1.390956 | 0.684930 | -0.617720 | C | 0.717680 | 1.166419 | 0.044456 |
| O | -0.003465 | 0.942929 | -0.413469 | O | -0.302640 | 0.465679 | 0.768964 |
| O | -2.176130 | 1.021595 | 0.494557 | O | 0.935525 | 2.394712 | 0.649221 |
| O | -3.009278 | -1.098186 | -1.042395 | O | 3.033052 | 1.006125 | -0.685385 |
| O | -1.035968 | -3.061298 | -0.149824 | O | 2.959119 | -1.878505 | -0.376463 |
| O | 1.069892 | -2.012617 | 1.462540 | O | 0.297391 | -2.956770 | -0.359778 |
| O | 2.746224 | 0.509084 | -0.549825 | O | -2.937861 | -0.482116 | 0.899314 |
| H | 2.622876 | 0.118751 | 1.444530 | H | -1.514279 | -1.479050 | 2.043953 |
| H | 2.067943 | 1.746815 | 0.984238 | H | -2.112233 | -2.317684 | 0.591249 |
| H | 0.144589 | 0.420315 | 1.602747 | H | -0.926159 | -0.680358 | -0.853789 |

| | | | | | | | |
|---|---|---|---|---|---|---|---|
| H | 1.034915 | -1.553370 | -0.563990 | H | 0.874939 | -1.875640 | 1.317999 |
| H | -1.499936 | -1.499770 | 1.134534 | H | 1.576471 | -0.974814 | -1.518124 |
| H | -1.160791 | -1.052025 | -1.871411 | H | 2.340312 | 0.317369 | 1.151655 |
| H | -1.655322 | 1.295919 | -1.486633 | H | 0.388995 | 1.287687 | -1.000390 |
| H | -2.349132 | 1.990246 | 0.502428 | H | 0.287613 | 3.055888 | 0.314201 |
| H | -3.463527 | -0.629850 | -0.322421 | H | 3.114882 | 1.914985 | -0.358654 |
| H | -1.968246 | -3.309526 | -0.234053 | H | 3.305565 | -1.873981 | 0.528788 |
| H | 0.874535 | -2.953484 | 1.339967 | H | 1.126609 | -3.449982 | -0.457401 |
| H | 2.231685 | 0.962469 | -1.234981 | H | -2.650000 | 0.407050 | 1.157925 |
| O | -2.746234 | 3.710941 | 0.565686 | O | -0.801765 | 4.313200 | -0.263627 |
| H | -2.002483 | 4.329377 | 0.543738 | H | -1.750212 | 4.136991 | -0.188957 |
| H | -3.373781 | 4.034138 | -0.095977 | H | -0.664034 | 4.621851 | -1.170209 |
| O | 5.532590 | 0.850998 | -0.718277 | O | -4.644450 | -0.592173 | -1.343155 |
| H | 5.745059 | 1.751009 | -0.439414 | H | -4.119461 | -0.409839 | -2.133161 |
| H | 4.558225 | 0.777237 | -0.645513 | H | -4.015473 | -0.540262 | -0.594029 |
| | α-glucose-2w-5 | | | | β-glucose-2w-5 | | |
| C | -0.661718 | 2.773230 | 0.338370 | C | -2.690918 | -1.672187 | 0.329687 |
| C | -0.996689 | 1.294494 | 0.500591 | C | -1.423781 | -0.999454 | -0.182194 |
| C | 0.221697 | 0.366916 | 0.355805 | C | -0.150821 | -1.809447 | 0.098559 |
| C | -0.241905 | -1.093040 | 0.335614 | C | 1.097115 | -1.009769 | -0.310698 |
| C | -1.328522 | -1.310742 | -0.723559 | C | 1.078449 | 0.380731 | 0.320758 |
| C | -2.480234 | -0.315612 | -0.541584 | C | -0.255518 | 1.075844 | 0.014994 |
| O | -1.988375 | 1.010547 | -0.509334 | O | -1.332439 | 0.264227 | 0.493622 |
| O | -3.153750 | -0.679489 | 0.648297 | O | -0.267861 | 2.292431 | 0.680587 |
| O | -1.788408 | -2.661311 | -0.713247 | O | 2.163007 | 1.147778 | -0.206136 |
| O | 0.882828 | -1.938401 | 0.046229 | O | 2.285021 | -1.748965 | -0.021921 |
| O | 1.080068 | 0.644258 | 1.454999 | O | -0.223423 | -3.031072 | -0.630844 |
| O | -0.145316 | 3.085478 | -0.956524 | O | -3.855652 | -0.907253 | 0.020845 |
| H | 0.109535 | 3.048860 | 1.059613 | H | -2.606531 | -1.830316 | 1.414066 |
| H | -1.564618 | 3.362697 | 0.544769 | H | -2.812645 | -2.642104 | -0.156012 |
| H | -1.433936 | 1.147092 | 1.497076 | H | -1.517003 | -0.831414 | -1.266505 |
| H | 0.728961 | 0.587086 | -0.594266 | H | -0.101347 | -2.018484 | 1.179017 |
| H | -0.637802 | -1.346814 | 1.328563 | H | 1.097939 | -0.902197 | -1.401624 |
| H | -0.891666 | -1.148889 | -1.713556 | H | 1.169314 | 0.302330 | 1.414545 |
| H | -3.161797 | -0.344653 | -1.396467 | H | -0.371585 | 1.214151 | -1.072168 |
| H | -4.027935 | -0.264933 | 0.664412 | H | -0.895840 | 2.910903 | 0.240904 |
| H | -2.393706 | -2.756297 | 0.040000 | H | 2.048676 | 2.057327 | 0.112947 |
| H | 0.554551 | -2.847121 | -0.032070 | H | 2.409583 | -1.797742 | 0.938282 |
| H | 1.949694 | 0.210338 | 1.299728 | H | 0.644108 | -3.459258 | -0.566320 |
| H | -0.804138 | 2.804610 | -1.608444 | H | -3.727166 | -0.021298 | 0.390901 |
| O | 3.333254 | -0.871905 | 0.934266 | O | -1.959370 | 4.088233 | -0.515418 |
| H | 3.958010 | -0.576005 | 0.239649 | H | -2.907342 | 3.898142 | -0.477754 |
| H | 2.701815 | -1.481756 | 0.509902 | H | -1.774478 | 4.313094 | -1.438150 |
| O | 5.182482 | 0.074361 | -0.953248 | O | 4.951989 | 0.514220 | 0.054355 |
| H | 5.790707 | -0.569266 | -1.342494 | H | 3.987898 | 0.657046 | -0.023754 |
| H | 4.824288 | 0.575278 | -1.699333 | H | 5.339072 | 1.398723 | 0.084272 |
| | α-glucose-2w-6 | | | | β-glucose-2w-6 | | |
| C | -0.085972 | 2.789279 | -0.154437 | C | 3.246931 | -1.000601 | 0.766541 |
| C | 0.111829 | 1.288847 | -0.343247 | C | 2.124969 | -0.372272 | -0.049421 |
| C | -1.162758 | 0.463581 | -0.094358 | C | 1.734950 | 1.030241 | 0.426089 |
| C | -0.816779 | -1.027883 | -0.119661 | C | 0.517513 | 1.538265 | -0.360127 |
| C | 0.339723 | -1.346490 | 0.834806 | C | -0.642832 | 0.534567 | -0.338379 |
| C | 1.549728 | -0.448925 | 0.550016 | C | -0.115806 | -0.859929 | -0.729812 |
| O | 1.154457 | 0.917761 | 0.578446 | O | 0.984618 | -1.232388 | 0.096995 |
| O | 2.087544 | -0.840783 | -0.685378 | O | -1.134594 | -1.800918 | -0.520315 |

| | | | | | | | |
|---|---|---|---|---|---|---|---|
| O | 0.685410 | -2.729430 | 0.760344 | O | -1.639945 | 0.984519 | -1.236432 |
| O | -1.974431 | -1.789359 | 0.268026 | O | 0.145304 | 2.839611 | 0.099670 |
| O | -2.085356 | 0.822322 | -1.118262 | O | 2.848734 | 1.900513 | 0.241485 |
| O | -0.450963 | 3.133822 | 1.183390 | O | 3.598913 | -2.293175 | 0.274328 |
| H | -0.895857 | 3.133007 | -0.800260 | H | 2.943115 | -1.055910 | 1.821331 |
| H | 0.839343 | 3.304136 | -0.444545 | H | 4.142151 | -0.380498 | 0.693339 |
| H | 0.447375 | 1.111337 | -1.373832 | H | 2.425685 | -0.333290 | -1.107855 |
| H | -1.568106 | 0.717306 | 0.895188 | H | 1.474276 | 0.975293 | 1.494959 |
| H | -0.533288 | -1.302035 | -1.144875 | H | 0.821235 | 1.691233 | -1.402713 |
| H | 0.008702 | -1.170535 | 1.862742 | H | -1.041794 | 0.455221 | 0.685630 |
| H | 2.294059 | -0.535939 | 1.348087 | H | 0.203894 | -0.859687 | -1.783118 |
| H | 3.033733 | -0.576548 | -0.737356 | H | -0.894099 | -2.634072 | -0.951378 |
| H | 1.231682 | -2.839212 | -0.035690 | H | -2.475419 | 0.496301 | -1.053697 |
| H | -1.707835 | -2.720129 | 0.321202 | H | -0.268432 | 2.765442 | 0.973125 |
| H | -2.961526 | 0.432738 | -0.916619 | H | 2.531931 | 2.805949 | 0.383896 |
| H | 0.232822 | 2.778361 | 1.770172 | H | 2.795631 | -2.834490 | 0.277691 |
| O | 4.751699 | -0.177390 | -0.880246 | O | -3.780960 | -0.663907 | -0.703128 |
| H | 4.993443 | 0.749573 | -0.744489 | H | -3.130268 | -1.352907 | -0.488094 |
| H | 5.361204 | -0.689453 | -0.330367 | H | -4.338377 | -0.536226 | 0.093803 |
| O | -4.390264 | -0.635386 | -0.407020 | O | -5.455985 | -0.211503 | 1.489625 |
| H | -4.903540 | -1.015117 | -1.132660 | H | -5.792411 | -0.987326 | 1.959520 |
| H | -3.711614 | -1.300381 | -0.169820 | H | -5.120148 | 0.381334 | 2.176381 |
| | α-glucose-2w-7 | | | | β-glucose-2w-7 | | |
| C | 1.172213 | -2.271709 | 0.331797 | C | 0.011873 | 2.656527 | 0.029887 |
| C | 0.630563 | -0.862500 | 0.548855 | C | -0.332706 | 1.216987 | -0.346763 |
| C | -0.828182 | -0.695927 | 0.090359 | C | -1.756022 | 0.825975 | 0.078544 |
| C | -1.231679 | 0.780413 | 0.151884 | C | -2.025244 | -0.661804 | -0.199204 |
| C | -0.232341 | 1.656582 | -0.611712 | C | -0.934353 | -1.539436 | 0.411136 |
| C | 1.196925 | 1.412206 | -0.118492 | C | 0.444709 | -1.041453 | -0.044405 |
| O | 1.508650 | 0.024274 | -0.188621 | O | 0.595282 | 0.340167 | 0.321651 |
| O | 1.275981 | 1.919590 | 1.194628 | O | 1.420093 | -1.779130 | 0.608849 |
| O | -0.590232 | 3.033437 | -0.524230 | O | -1.139531 | -2.884921 | -0.002609 |
| O | -2.536528 | 0.933517 | -0.429541 | O | -3.346039 | -1.011522 | 0.222181 |
| O | -1.613203 | -1.505553 | 0.957916 | O | -2.682200 | 1.636961 | -0.639714 |
| O | 1.137889 | -2.682494 | -1.035219 | O | 1.244380 | 3.104966 | -0.521492 |
| H | 0.548828 | -2.976444 | 0.884444 | H | 0.012107 | 2.749717 | 1.125319 |
| H | 2.194454 | -2.325327 | 0.727588 | H | -0.771340 | 3.306859 | -0.366396 |
| H | 0.695210 | -0.626923 | 1.618682 | H | -0.227231 | 1.096907 | -1.435547 |
| H | -0.918656 | -1.043504 | -0.947949 | H | -1.857496 | 1.009063 | 1.160180 |
| H | -1.266643 | 1.090585 | 1.204997 | H | -2.033584 | -0.815174 | -1.284783 |
| H | -0.276637 | 1.400522 | -1.674374 | H | -0.962403 | -1.466702 | 1.509612 |
| H | 1.926089 | 1.897668 | -0.772668 | H | 0.540833 | -1.129614 | -1.137673 |
| H | 2.200987 | 2.072559 | 1.434320 | H | 2.221610 | -1.864534 | 0.042672 |
| H | -0.333729 | 3.347312 | 0.358135 | H | -0.407708 | -3.410090 | 0.355217 |
| H | -2.740956 | 1.881060 | -0.451715 | H | -3.379389 | -1.036385 | 1.190689 |
| H | -2.522894 | -1.574341 | 0.598927 | H | -3.565908 | 1.270059 | -0.481569 |
| H | 1.689834 | -2.075150 | -1.548404 | H | 1.976253 | 2.684914 | -0.025969 |
| O | 4.392445 | -0.298151 | -0.117532 | O | 3.011445 | 1.567737 | 1.065932 |
| H | 4.678397 | -0.382211 | -1.036653 | H | 3.832947 | 1.229956 | 0.684705 |
| H | 3.420204 | -0.231446 | -0.154435 | H | 2.335514 | 0.878481 | 0.913518 |
| O | -4.166992 | -1.291823 | -0.193373 | O | 3.659031 | -2.131614 | -0.920899 |
| H | -4.924128 | -1.266622 | 0.406936 | H | 4.195641 | -1.360293 | -1.151593 |
| H | -3.879457 | -0.364495 | -0.316215 | H | 3.540930 | -2.625079 | -1.744735 |
| | α-glucose-2w-8 | | | | β-glucose-2w-8 | | |
| C | -2.551712 | 1.361053 | 0.343557 | C | -1.818086 | -0.708171 | 0.327362 |

| | | | | | | | |
|---|---|---|---|---|---|---|---|
| C | -1.228048 | 0.630875 | 0.543928 | C | -0.425606 | -0.445084 | -0.236694 |
| C | -0.012402 | 1.402772 | 0.027511 | C | 0.622685 | -1.414261 | 0.330779 |
| C | 1.263076 | 0.568126 | 0.151651 | C | 2.032843 | -1.042586 | -0.155102 |
| C | 1.082261 | -0.787375 | -0.548715 | C | 2.339789 | 0.429164 | 0.122166 |
| C | -0.208261 | -1.485999 | -0.086064 | C | 1.211192 | 1.299142 | -0.447587 |
| O | -1.331749 | -0.620652 | -0.181818 | O | -0.041443 | 0.898550 | 0.117827 |
| O | 0.021852 | -1.921794 | 1.237878 | O | 1.455898 | 2.621830 | -0.074143 |
| O | 2.196124 | -1.665365 | -0.333694 | O | 3.582564 | 0.758408 | -0.483708 |
| O | 2.302423 | 1.350280 | -0.429439 | O | 2.997788 | -1.941077 | 0.394131 |
| O | 0.099039 | 2.605841 | 0.786488 | O | 0.286528 | -2.733573 | -0.087653 |
| O | -2.813126 | 1.695204 | -1.019249 | O | -2.833463 | 0.058442 | -0.321056 |
| H | -2.517645 | 2.303886 | 0.893080 | H | -1.817013 | -0.515418 | 1.408362 |
| H | -3.362987 | 0.748446 | 0.756923 | H | -2.059354 | -1.760705 | 0.165571 |
| H | -1.106878 | 0.421516 | 1.614162 | H | -0.457900 | -0.537887 | -1.332385 |
| H | -0.170369 | 1.642824 | -1.033050 | H | 0.596820 | -1.350426 | 1.430352 |
| H | 1.469753 | 0.397821 | 1.218249 | H | 2.082340 | -1.213153 | -1.236886 |
| H | 1.023940 | -0.623963 | -1.628362 | H | 2.378075 | 0.608282 | 1.207834 |
| H | -0.446690 | -2.329920 | -0.738931 | H | 1.163495 | 1.198344 | -1.541441 |
| H | -0.602821 | -2.624702 | 1.466963 | H | 1.058033 | 3.226287 | -0.716578 |
| H | 2.052060 | -2.096986 | 0.525915 | H | 3.761401 | 1.692798 | -0.300855 |
| H | 3.173012 | 0.928434 | -0.268149 | H | 3.109838 | -1.757368 | 1.339304 |
| H | 0.951804 | 3.004286 | 0.553879 | H | 1.034149 | -3.307141 | 0.141614 |
| H | -2.877836 | 0.874675 | -1.528330 | H | -2.780086 | 0.986034 | -0.001157 |
| O | -3.803841 | -2.151961 | -0.185935 | O | -2.255833 | 2.550720 | 0.758635 |
| H | -3.945865 | -2.427927 | -1.100992 | H | -2.415435 | 3.361410 | 0.256549 |
| H | -2.984279 | -1.623936 | -0.199074 | H | -1.322519 | 2.312159 | 0.604994 |
| O | 4.603531 | -0.221529 | -0.290667 | O | -5.434801 | -0.944126 | -0.256045 |
| H | 3.946533 | -0.947178 | -0.299500 | H | -5.649236 | -1.210573 | -1.159218 |
| H | 5.134762 | -0.344199 | 0.507515 | H | -4.517426 | -0.595247 | -0.295494 |
| | | α-glucose-2w-9 | | | | β-glucose-2w-9 | |
| C | 1.517677 | 1.883382 | 0.991778 | C | -1.608252 | 1.507331 | -0.084057 |
| C | 0.707799 | 0.604552 | 0.781906 | C | -0.512326 | 0.513380 | -0.463924 |
| C | 1.538605 | -0.587645 | 0.294568 | C | -0.744096 | -0.887880 | 0.123274 |
| C | 0.630212 | -1.789893 | 0.053563 | C | 0.438887 | -1.815393 | -0.194994 |
| C | -0.502709 | -1.431838 | -0.914081 | C | 1.761714 | -1.188952 | 0.245402 |
| C | -1.257632 | -0.186741 | -0.423995 | C | 1.871180 | 0.225343 | -0.340543 |
| O | -0.336088 | 0.883035 | -0.183051 | O | 0.739112 | 1.004887 | 0.058049 |
| O | -1.976619 | -0.546908 | 0.720488 | O | 3.018509 | 0.819325 | 0.187853 |
| O | -1.392091 | -2.532130 | -1.094001 | O | 2.827318 | -2.008464 | -0.214483 |
| O | 1.448868 | -2.841014 | -0.467123 | O | 0.204142 | -3.114585 | 0.348852 |
| O | 2.517598 | -0.877809 | 1.293906 | O | -1.943359 | -1.443807 | -0.427735 |
| O | 2.290405 | 2.289174 | -0.132740 | O | -1.477813 | 2.761000 | -0.741749 |
| H | 2.218886 | 1.702539 | 1.810317 | H | -1.618109 | 1.632809 | 1.007839 |
| H | 0.829118 | 2.679938 | 1.304074 | H | -2.570806 | 1.087442 | -0.384983 |
| H | 0.240040 | 0.349078 | 1.741808 | H | -0.449662 | 0.451076 | -1.560290 |
| H | 2.033584 | -0.316302 | -0.647932 | H | -0.849454 | -0.798505 | 1.215242 |
| H | 0.199607 | -2.102437 | 1.015503 | H | 0.473089 | -1.975410 | -1.279194 |
| H | -0.075041 | -1.218828 | -1.898390 | H | 1.789764 | -1.094776 | 1.341707 |
| H | -1.921745 | 0.198350 | -1.204510 | H | 1.914214 | 0.184109 | -1.438577 |
| H | -2.731671 | 0.071213 | 0.852130 | H | 3.350879 | 1.496412 | -0.418339 |
| H | -1.978745 | -2.548747 | -0.319706 | H | 3.657903 | -1.602740 | 0.075311 |
| H | 0.892639 | -3.625088 | -0.583251 | H | 0.280693 | -3.081776 | 1.314872 |
| H | 2.947103 | -1.709319 | 1.043631 | H | -1.896030 | -2.404685 | -0.289215 |
| H | 1.689354 | 2.672908 | -0.802061 | H | -0.766438 | 3.266633 | -0.299260 |
| O | 0.168881 | 3.104562 | -1.821084 | O | 0.695778 | 3.755610 | 0.761643 |

| | | | |
|---|---|---|---|
| H | 0.236370 | 2.942122 | -2.771625 |
| H | -0.225463 | 2.298595 | -1.433031 |
| O | -4.130596 | 1.102998 | 1.125345 |
| H | -4.900127 | 0.941411 | 0.561577 |
| H | -3.980495 | 2.058400 | 1.095098 |

α-glucose-2w-10

| | | | |
|---|---|---|---|
| C | 2.661533 | -0.699091 | 0.697958 |
| C | 1.169740 | -0.392973 | 0.617214 |
| C | 0.332809 | -1.555937 | 0.069761 |
| C | -1.116506 | -1.125255 | -0.140759 |
| C | -1.176555 | 0.124483 | -1.027634 |
| C | -0.298098 | 1.240673 | -0.438485 |
| O | 1.032401 | 0.759890 | -0.252491 |
| O | -0.858795 | 1.688161 | 0.781824 |
| O | -2.508799 | 0.531371 | -1.291991 |
| O | -1.795734 | -2.224557 | -0.751257 |
| O | 0.425602 | -2.624404 | 1.012510 |
| O | 3.231863 | -1.069905 | -0.556678 |
| H | 2.809252 | -1.547577 | 1.368842 |
| H | 3.180342 | 0.171085 | 1.119877 |
| H | 0.815839 | -0.132398 | 1.622575 |
| H | 0.747559 | -1.871277 | -0.897263 |
| H | -1.569246 | -0.910771 | 0.837743 |
| H | -0.744821 | -0.125984 | -2.002312 |
| H | -0.201712 | 2.069946 | -1.144205 |
| H | -0.425590 | 2.512988 | 1.047110 |
| H | -2.966161 | 0.768273 | -0.455066 |
| H | -2.698371 | -1.939909 | -0.958456 |
| H | -0.150755 | -3.338466 | 0.702907 |
| H | 3.135456 | -0.325921 | -1.168084 |
| O | 2.879798 | 2.996747 | -0.151145 |
| H | 3.165947 | 3.153658 | -1.060623 |
| H | 2.287666 | 2.223287 | -0.195836 |
| O | -3.628124 | 1.216717 | 1.193475 |
| H | -4.280976 | 1.928599 | 1.237390 |
| H | -2.767666 | 1.620901 | 1.403100 |

α-allose-2w-1

| | | | |
|---|---|---|---|
| C | -0.349771 | -1.313716 | 0.323878 |
| C | 0.730255 | -1.724139 | -0.700132 |
| C | 1.935136 | -0.785132 | -0.617030 |
| C | 1.491954 | 0.673268 | -0.764449 |
| C | 0.357999 | 1.015542 | 0.220699 |
| C | -0.259945 | 2.391316 | -0.051099 |
| O | 0.027667 | -1.544723 | 1.654344 |
| O | 1.097947 | -3.088912 | -0.560732 |
| O | 2.549199 | -1.003691 | 0.666128 |
| O | 2.648956 | 1.474001 | -0.528101 |
| O | -0.712434 | 0.059567 | 0.085409 |
| O | 0.733241 | 3.424867 | 0.044175 |
| H | 0.968970 | -1.306883 | 1.756570 |
| H | 1.585340 | -3.194200 | 0.270184 |
| H | 3.199318 | -0.296184 | 0.804788 |
| H | 2.341399 | 2.390277 | -0.396923 |
| H | 0.812843 | 3.705829 | 0.966742 |
| H | -1.272152 | -1.871148 | 0.153173 |

| | | | |
|---|---|---|---|
| H | 1.349045 | 4.356086 | 0.378056 |
| H | 1.046961 | 2.854207 | 0.636982 |
| O | -4.632631 | -0.841536 | 0.345540 |
| H | -4.709654 | -0.934929 | 1.303989 |
| H | -3.693493 | -1.018361 | 0.141153 |

β-glucose-2w-10

| | | | |
|---|---|---|---|
| C | -1.299188 | -2.164843 | 0.093394 |
| C | -0.604943 | -0.891991 | -0.388150 |
| C | 0.840556 | -0.790991 | 0.134346 |
| C | 1.458068 | 0.588024 | -0.186122 |
| C | 0.516327 | 1.742497 | 0.160297 |
| C | -0.877376 | 1.482596 | -0.419637 |
| O | -1.367500 | 0.237958 | 0.079041 |
| O | -1.721206 | 2.502348 | 0.026917 |
| O | 1.066623 | 2.945303 | -0.361252 |
| O | 2.734542 | 0.751548 | 0.456921 |
| O | 1.567907 | -1.844370 | -0.482088 |
| O | -2.557728 | -2.392125 | -0.530285 |
| H | -1.403576 | -2.123957 | 1.186985 |
| H | -0.657547 | -3.012225 | -0.156473 |
| H | -0.591465 | -0.886280 | -1.488207 |
| H | 0.826278 | -0.919789 | 1.228410 |
| H | 1.680275 | 0.623188 | -1.258185 |
| H | 0.400272 | 1.813018 | 1.253516 |
| H | -0.843343 | 1.447564 | -1.518328 |
| H | -2.454424 | 2.621752 | -0.593159 |
| H | 0.458733 | 3.666276 | -0.139068 |
| H | 2.595870 | 0.946119 | 1.396574 |
| H | 2.493729 | -1.855843 | -0.159806 |
| H | -3.213716 | -1.787831 | -0.128562 |
| O | -4.027033 | -0.387683 | 0.824023 |
| H | -4.752772 | 0.078527 | 0.387655 |
| H | -3.223218 | 0.139171 | 0.654297 |
| O | 4.279409 | -1.563670 | 0.186708 |
| H | 4.667891 | -1.897942 | 1.006314 |
| H | 4.016234 | -0.637962 | 0.363390 |

β-allose-2w-1

| | | | |
|---|---|---|---|
| C | -2.440474 | -0.264091 | -0.147508 |
| C | -1.407337 | -1.344720 | -0.470202 |
| C | -0.149637 | -1.158701 | 0.381694 |
| C | 0.370045 | 0.281810 | 0.296406 |
| C | -0.760486 | 1.304505 | 0.533035 |
| C | -0.344590 | 2.744671 | 0.254557 |
| O | -3.507458 | -0.419191 | -1.037274 |
| O | -1.906221 | -2.651642 | -0.199074 |
| O | -0.420937 | -1.402705 | 1.767131 |
| O | 1.430504 | 0.480205 | 1.235462 |
| O | -1.854467 | 1.020659 | -0.357484 |
| O | 0.063428 | 2.948319 | -1.099023 |
| H | -4.278105 | 0.064247 | -0.706674 |
| H | -2.740993 | -2.762628 | -0.677611 |
| H | -0.782831 | -2.296953 | 1.851516 |
| H | 1.192250 | 0.007571 | 2.050226 |
| H | -0.679543 | 2.702554 | -1.669600 |
| H | -2.778658 | -0.341587 | 0.895330 |

| | | | | | | | |
|---|---|---|---|---|---|---|---|
| H | 0.286892 | -1.631894 | -1.695185 | H | -1.141133 | -1.241720 | -1.531766 |
| H | 2.653634 | -1.029320 | -1.409385 | H | 0.628161 | -1.850308 | 0.032784 |
| H | 1.124294 | 0.818195 | -1.791103 | H | 0.794541 | 0.452668 | -0.696387 |
| H | 0.747765 | 0.992690 | 1.246581 | H | -1.101228 | 1.235879 | 1.576766 |
| H | -1.084806 | 2.577656 | 0.643201 | H | 0.511135 | 3.001960 | 0.882368 |
| H | -0.650157 | 2.426825 | -1.072400 | H | -1.179523 | 3.409590 | 0.510729 |
| O | -3.398729 | 0.585901 | 0.798713 | O | 4.208406 | 0.492019 | 0.524756 |
| H | -2.453470 | 0.421090 | 0.601980 | H | 3.257343 | 0.445491 | 0.736457 |
| H | -3.523892 | 0.343214 | 1.725973 | H | 4.373151 | -0.209437 | -0.133587 |
| O | -5.252553 | -0.450501 | -1.015060 | O | 4.724754 | -1.532256 | -1.436633 |
| H | -5.130313 | -1.407053 | -1.066546 | H | 5.272294 | -2.274885 | -1.146061 |
| H | -4.585783 | -0.134011 | -0.367864 | H | 5.143944 | -1.206543 | -2.245275 |
| | α-allose-2w-2 | | | | β-allose-2w-2 | | |
| C | 0.036585 | -1.397775 | 0.216548 | C | -2.219640 | -1.099147 | -0.208064 |
| C | 0.716202 | -0.053517 | -0.122455 | C | -2.478377 | -0.025417 | 0.850424 |
| C | -0.206566 | 1.119872 | 0.211061 | C | -1.803471 | 1.290900 | 0.459489 |
| C | -1.565338 | 0.938955 | -0.471669 | C | -0.324918 | 1.074252 | 0.112930 |
| C | -2.158382 | -0.449960 | -0.163807 | C | -0.153622 | -0.088665 | -0.887631 |
| C | -3.401582 | -0.757205 | -1.003659 | C | 1.298846 | -0.484655 | -1.117473 |
| O | -0.092742 | -1.604995 | 1.603231 | O | -2.754848 | -2.299769 | 0.267823 |
| O | 1.987759 | 0.076085 | 0.509398 | O | -3.870445 | 0.244230 | 0.996050 |
| O | -0.357894 | 1.145367 | 1.642177 | O | -2.412419 | 1.856895 | -0.707007 |
| O | -2.404101 | 1.988917 | 0.010564 | O | 0.252126 | 2.274501 | -0.394441 |
| O | -1.207881 | -1.477894 | -0.478716 | O | -0.811268 | -1.263182 | -0.378166 |
| O | -4.429965 | 0.221992 | -0.778845 | O | 1.929239 | -0.947029 | 0.084511 |
| H | -0.371318 | -0.765983 | 2.016974 | H | -2.849299 | -2.925600 | -0.464450 |
| H | 1.862301 | 0.089826 | 1.470467 | H | -4.325547 | -0.594119 | 1.164532 |
| H | -1.086183 | 1.753930 | 1.846753 | H | -3.361163 | 1.949622 | -0.535952 |
| H | -3.319932 | 1.755082 | -0.229782 | H | -0.390962 | 2.667280 | -1.006298 |
| H | -4.956200 | -0.038681 | -0.009916 | H | 1.412560 | -1.694387 | 0.422096 |
| H | 0.623211 | -2.232777 | -0.170130 | H | -2.669219 | -0.818658 | -1.170797 |
| H | 0.916531 | -0.045139 | -1.196838 | H | -2.049633 | -0.382712 | 1.797613 |
| H | 0.249232 | 2.060659 | -0.122084 | H | -1.882371 | 1.998858 | 1.295038 |
| H | -1.420191 | 1.029865 | -1.558421 | H | 0.220559 | 0.827212 | 1.028005 |
| H | -2.427309 | -0.498949 | 0.900253 | H | -0.591930 | 0.195006 | -1.855952 |
| H | -3.763018 | -1.765368 | -0.778832 | H | 1.866990 | 0.384454 | -1.453612 |
| H | -3.151569 | -0.706386 | -2.067354 | H | 1.343296 | -1.259952 | -1.891784 |
| O | 4.425606 | -1.118510 | -0.458665 | O | 4.742533 | -1.455967 | 0.055826 |
| H | 3.600226 | -0.714426 | -0.134189 | H | 3.785587 | -1.261839 | 0.063313 |
| H | 5.062157 | -0.384994 | -0.557243 | H | 5.189544 | -0.622778 | 0.297504 |
| O | 6.313992 | 1.013788 | -0.758324 | O | 6.198750 | 0.910661 | 0.753710 |
| H | 6.746150 | 1.072921 | -1.621636 | H | 5.928238 | 1.379438 | 1.555355 |
| H | 7.032219 | 1.030213 | -0.110689 | H | 6.271102 | 1.591842 | 0.070895 |
| | α-allose-2w-3 | | | | β-allose-2w-3 | | |
| C | -0.230473 | -1.166036 | -0.879376 | C | -0.545237 | -1.385369 | -0.525542 |
| C | 1.214203 | -1.701151 | -0.776883 | C | -1.588968 | -1.230842 | 0.581064 |
| C | 2.011902 | -0.890294 | 0.246691 | C | -2.274108 | 0.133738 | 0.487027 |
| C | 1.937679 | 0.603900 | -0.080200 | C | -1.245427 | 1.270060 | 0.412238 |
| C | 0.476930 | 1.062412 | -0.255243 | C | -0.171665 | 0.989018 | -0.659107 |
| C | 0.371734 | 2.483615 | -0.816038 | C | 0.968337 | 2.007595 | -0.661098 |
| O | -0.986444 | -1.443307 | 0.283731 | O | 0.129298 | -2.591142 | -0.309272 |
| O | 1.251012 | -3.096455 | -0.512247 | O | -2.609113 | -2.221516 | 0.484651 |
| O | 1.434070 | -1.156486 | 1.538896 | O | -3.070199 | 0.229908 | -0.700510 |
| O | 2.576964 | 1.288509 | 0.997223 | O | -1.897322 | 2.514473 | 0.169407 |
| O | -0.202908 | 0.220699 | -1.199388 | O | 0.400193 | -0.318288 | -0.428029 |

| | | | | | | | |
|---|---|---|---|---|---|---|---|
| O | 1.030515 | 3.427452 | 0.045045 | O | 1.600073 | 2.182398 | 0.600797 |
| H | -0.424630 | -1.289716 | 1.068611 | H | 0.582574 | -2.861209 | -1.120712 |
| H | 0.924257 | -3.250222 | 0.386896 | H | -2.185686 | -3.092514 | 0.466187 |
| H | 1.802826 | -0.510225 | 2.162598 | H | -3.696670 | -0.508607 | -0.703168 |
| H | 2.339923 | 2.231001 | 0.917032 | H | -2.575050 | 2.356950 | -0.507299 |
| H | 0.410612 | 3.724452 | 0.725857 | H | 2.141510 | 1.385783 | 0.794666 |
| H | -0.758543 | -1.635777 | -1.710377 | H | -1.014273 | -1.363682 | -1.518653 |
| H | 1.677997 | -1.570267 | -1.758422 | H | -1.063824 | -1.304308 | 1.544026 |
| H | 3.060104 | -1.214255 | 0.246083 | H | -2.912227 | 0.278003 | 1.368801 |
| H | 2.479011 | 0.776245 | -1.022353 | H | -0.746734 | 1.362556 | 1.380724 |
| H | -0.031701 | 1.023728 | 0.717675 | H | -0.636481 | 1.002457 | -1.656257 |
| H | -0.679730 | 2.750185 | -0.959972 | H | 0.552281 | 2.976745 | -0.948657 |
| H | 0.879996 | 2.537106 | -1.783261 | H | 1.695820 | 1.708379 | -1.428059 |
| O | -3.857628 | -1.410625 | 0.463261 | O | 2.870233 | -0.260983 | 1.022437 |
| H | -4.171230 | -0.488921 | 0.393405 | H | 3.698176 | -0.459114 | 0.537002 |
| H | -2.886243 | -1.370104 | 0.394795 | H | 2.139929 | -0.646523 | 0.507180 |
| O | -4.821582 | 1.279837 | 0.262905 | O | 5.264330 | -0.803111 | -0.344138 |
| H | -5.297503 | 1.606279 | 1.039184 | H | 5.771061 | -0.023126 | -0.610286 |
| H | -5.408977 | 1.448826 | -0.486870 | H | 5.892048 | -1.370899 | 0.124263 |
| | α-allose-2w-4 | | | | β-allose-2w-4 | | |
| C | -1.831248 | 0.932555 | -0.124524 | C | -1.450919 | 0.536399 | -0.136468 |
| C | -2.127693 | -0.348084 | -0.933465 | C | -1.623457 | -0.754184 | -0.940346 |
| C | -1.287689 | -1.517439 | -0.416014 | C | -0.853964 | -1.909156 | -0.298568 |
| C | 0.199579 | -1.150144 | -0.398023 | C | 0.604874 | -1.518500 | -0.031514 |
| C | 0.437578 | 0.181510 | 0.340907 | C | 0.690701 | -0.161918 | 0.699841 |
| C | 1.865923 | 0.706843 | 0.167149 | C | 2.110653 | 0.374948 | 0.820165 |
| O | -2.299723 | 0.879266 | 1.196351 | O | -2.077325 | 1.565784 | -0.826304 |
| O | -3.515280 | -0.650316 | -0.958090 | O | -2.993464 | -1.145420 | -1.009003 |
| O | -1.755542 | -1.812060 | 0.912710 | O | -1.416790 | -2.265533 | 0.970322 |
| O | 0.871478 | -2.234843 | 0.241185 | O | 1.271742 | -2.540724 | 0.705141 |
| O | -0.420392 | 1.204110 | -0.198240 | O | -0.050147 | 0.830270 | -0.030238 |
| O | 2.826482 | -0.244540 | 0.667601 | O | 2.695134 | 0.638368 | -0.464013 |
| H | -2.138617 | -0.017097 | 1.547573 | H | -2.275081 | 2.308635 | -0.211955 |
| H | -3.785875 | -0.933649 | -0.071767 | H | -3.506194 | -0.376304 | -1.300107 |
| H | -1.127405 | -2.432863 | 1.315477 | H | -2.359477 | -2.445069 | 0.838901 |
| H | 1.776713 | -1.940548 | 0.444591 | H | 0.661369 | -2.842492 | 1.396890 |
| H | 2.935405 | -0.122363 | 1.622221 | H | 2.108235 | 1.248796 | -0.936218 |
| H | -2.298815 | 1.799923 | -0.593220 | H | -1.864037 | 0.424674 | 0.877006 |
| H | -1.844519 | -0.149388 | -1.970669 | H | -1.222189 | -0.572070 | -1.947675 |
| H | -1.436820 | -2.395854 | -1.056100 | H | -0.881309 | -2.779651 | -0.967515 |
| H | 0.539528 | -1.044278 | -1.438603 | H | 1.127857 | -1.431338 | -0.988070 |
| H | 0.229930 | 0.047100 | 1.410656 | H | 0.276358 | -0.270132 | 1.713398 |
| H | 1.980277 | 1.669809 | 0.672308 | H | 2.746428 | -0.367045 | 1.306273 |
| H | 2.088244 | 0.838639 | -0.895065 | H | 2.102191 | 1.289227 | 1.425479 |
| O | 0.163660 | 4.025359 | -0.210694 | O | -2.685534 | 3.674462 | 0.834488 |
| H | -0.024008 | 3.069460 | -0.153140 | H | -3.380057 | 3.529429 | 1.492359 |
| H | -0.184542 | 4.404254 | 0.607055 | H | -1.949648 | 4.077483 | 1.316379 |
| O | 5.362598 | -0.413562 | -0.649044 | O | 5.407632 | 1.374246 | -0.573655 |
| H | 5.387697 | -1.280349 | -1.074724 | H | 5.871819 | 0.645609 | -1.005524 |
| H | 4.497853 | -0.371557 | -0.197001 | H | 4.466404 | 1.103075 | -0.542187 |
| | α-allose-2w-5 | | | | β-allose-2w-5 | | |
| C | -0.344857 | 1.381325 | 0.291738 | C | 1.579814 | 0.281690 | -0.130007 |
| C | -1.401199 | 0.401529 | -0.261519 | C | 0.699507 | 1.369350 | -0.749088 |
| C | -1.015783 | -1.045348 | 0.046041 | C | -0.667571 | 1.425103 | -0.066609 |
| C | 0.399840 | -1.341519 | -0.459622 | C | -1.309607 | 0.034000 | -0.009979 |

| | | | | | | | |
|---|---|---|---|---|---|---|---|
| C | 1.405481 | -0.292036 | 0.051552 | C | -0.325294 | -1.019842 | 0.537568 |
| C | 2.779930 | -0.425071 | -0.612534 | C | -0.845082 | -2.449291 | 0.429858 |
| O | -0.327211 | 1.446346 | 1.692458 | O | 2.757633 | 0.209114 | -0.862023 |
| O | -2.707374 | 0.721824 | 0.213247 | O | 1.291173 | 2.659682 | -0.613480 |
| O | -1.090446 | -1.201928 | 1.473905 | O | -0.554792 | 1.869020 | 1.290926 |
| O | 0.727769 | -2.650187 | 0.003314 | O | -2.500148 | 0.072304 | 0.784725 |
| O | 0.934322 | 1.032361 | -0.262917 | O | 0.893422 | -0.974415 | -0.222586 |
| O | 3.336525 | -1.728377 | -0.376224 | O | -1.091441 | -2.844571 | -0.920710 |
| H | -0.440245 | 0.544970 | 2.049318 | H | 3.464801 | -0.209445 | -0.320793 |
| H | -2.760416 | 0.511874 | 1.158569 | H | 2.205829 | 2.599468 | -0.928143 |
| H | -0.685062 | -2.055200 | 1.697501 | H | -0.086944 | 2.717073 | 1.291653 |
| H | 1.689881 | -2.761579 | -0.112432 | H | -2.320881 | 0.636190 | 1.555382 |
| H | 3.814842 | -1.723231 | 0.464897 | H | -0.261311 | -2.745108 | -1.409712 |
| H | -0.532764 | 2.393122 | -0.070566 | H | 1.779635 | 0.496912 | 0.930219 |
| H | -1.436478 | 0.533253 | -1.345680 | H | 0.559622 | 1.116339 | -1.809818 |
| H | -1.724575 | -1.732260 | -0.432915 | H | -1.323418 | 2.109046 | -0.621554 |
| H | 0.385598 | -1.311973 | -1.559275 | H | -1.616019 | -0.262906 | -1.016507 |
| H | 1.520036 | -0.396665 | 1.138197 | H | -0.115128 | -0.805243 | 1.596210 |
| H | 3.450996 | 0.358602 | -0.248361 | H | -1.796496 | -2.531411 | 0.959653 |
| H | 2.679167 | -0.320595 | -1.696710 | H | -0.120972 | -3.124858 | 0.903434 |
| O | 2.626225 | 3.342146 | -0.601122 | O | 4.810497 | -0.908569 | 0.590319 |
| H | 2.079617 | 2.548750 | -0.446017 | H | 5.052715 | -0.468398 | 1.417171 |
| H | 2.672982 | 3.790942 | 0.253267 | H | 4.739102 | -1.848888 | 0.806614 |
| O | -5.037274 | 0.063431 | -1.282668 | O | -5.063815 | 0.298709 | -0.375012 |
| H | -5.126811 | -0.898268 | -1.299226 | H | -5.126676 | 1.157760 | -0.812108 |
| H | -4.227518 | 0.240040 | -0.764441 | H | -4.164698 | 0.267217 | 0.010572 |
| | α-allose-2w-6 | | | | β-allose-2w-6 | | |
| C | 1.815110 | -1.542191 | -0.405775 | C | 1.102791 | -0.495615 | -0.015224 |
| C | 2.352946 | -0.095793 | -0.485019 | C | 0.360337 | -1.445026 | 0.926297 |
| C | 1.361891 | 0.883520 | 0.150842 | C | -1.055386 | -1.722751 | 0.419843 |
| C | -0.020823 | 0.710899 | -0.482939 | C | -1.803511 | -0.417415 | 0.120789 |
| C | -0.473776 | -0.759589 | -0.430966 | C | -0.956616 | 0.531643 | -0.752141 |
| C | -1.766167 | -1.000084 | -1.216957 | C | -1.590563 | 1.908997 | -0.945780 |
| O | 1.818310 | -2.052771 | 0.907032 | O | 2.331755 | -0.187103 | 0.550773 |
| O | 3.657266 | 0.020023 | 0.067040 | O | 1.027057 | -2.701559 | 1.028752 |
| O | 1.320912 | 0.590657 | 1.559177 | O | -1.028729 | -2.467871 | -0.804090 |
| O | -0.930786 | 1.552570 | 0.238540 | O | -3.060357 | -0.693408 | -0.493861 |
| O | 0.526762 | -1.600392 | -1.020803 | O | 0.338974 | 0.716429 | -0.139674 |
| O | -2.820638 | -0.168886 | -0.715798 | O | -1.942272 | 2.570338 | 0.264913 |
| H | 1.509849 | -1.352068 | 1.511816 | H | 2.961057 | 0.108180 | -0.146654 |
| H | 3.605104 | -0.152992 | 1.018971 | H | 1.956404 | -2.528272 | 1.241742 |
| H | 0.576291 | 1.080329 | 1.942904 | H | -0.493670 | -3.261921 | -0.657805 |
| H | -1.831344 | 1.216427 | 0.037551 | H | -2.912025 | -1.389958 | -1.153211 |
| H | -3.319572 | -0.658625 | -0.024129 | H | -1.123507 | 2.854088 | 0.720104 |
| H | 2.435911 | -2.213340 | -1.001763 | H | 1.217325 | -0.940173 | -1.013978 |
| H | 2.453450 | 0.157115 | -1.544062 | H | 0.301589 | -0.956497 | 1.909519 |
| H | 1.710699 | 1.913549 | 0.004616 | H | -1.606907 | -2.290721 | 1.180982 |
| H | 0.037556 | 1.030697 | -1.533324 | H | -2.022945 | 0.089689 | 1.064214 |
| H | -0.640458 | -1.047835 | 0.615894 | H | -0.816704 | 0.085599 | -1.748173 |
| H | -2.040068 | -2.058795 | -1.160374 | H | -2.514692 | 1.777091 | -1.514157 |
| H | -1.610749 | -0.739341 | -2.269035 | H | -0.905811 | 2.524022 | -1.545382 |
| O | -4.195280 | -1.619293 | 1.182454 | O | 0.567051 | 2.968103 | 1.504186 |
| H | -4.152857 | -1.293730 | 2.092669 | H | 1.178683 | 3.650222 | 1.196244 |
| H | -5.134437 | -1.766048 | 1.002126 | H | 0.792962 | 2.153832 | 1.011508 |
| O | -0.786582 | 4.367618 | -0.037435 | O | 4.170085 | 0.616491 | -1.320044 |

| | | | | | | | |
|---|---|---|---|---|---|---|---|
| H | -1.089801 | 4.596426 | -0.925545 | H | 4.436749 | -0.034151 | -1.984662 |
| H | -0.844366 | 3.391452 | 0.014909 | H | 3.980682 | 1.428614 | -1.810639 |
| | α-allose-2w-7 | | | | β-allose-2w-7 | | |
| C | -1.637673 | 0.516304 | -0.778413 | C | 0.158081 | 1.127688 | 0.144828 |
| C | -2.108113 | -0.930654 | -0.515928 | C | 0.944696 | 0.093907 | 0.951421 |
| C | -1.104614 | -1.665916 | 0.375408 | C | 0.787699 | -1.304260 | 0.346791 |
| C | 0.304769 | -1.573524 | -0.216540 | C | -0.706082 | -1.647699 | 0.217089 |
| C | 0.681905 | -0.113064 | -0.526943 | C | -1.467995 | -0.520739 | -0.514277 |
| C | 1.988750 | -0.003608 | -1.319118 | C | -2.981011 | -0.708222 | -0.509845 |
| O | -1.724543 | 1.321675 | 0.384991 | O | 0.276792 | 2.356424 | 0.783352 |
| O | -3.430329 | -0.976267 | 0.002325 | O | 2.333229 | 0.435447 | 1.015976 |
| O | -1.151680 | -1.037585 | 1.671003 | O | 1.330554 | -1.403773 | -0.970991 |
| O | 1.194380 | -2.145790 | 0.741878 | O | -0.862506 | -2.888583 | -0.465797 |
| O | -0.331647 | 0.502679 | -1.338486 | O | -1.222031 | 0.740629 | 0.133540 |
| O | 3.065101 | -0.629140 | -0.612290 | O | -3.531375 | -0.699996 | 0.809247 |
| H | -1.413829 | 0.801064 | 1.151912 | H | 0.080108 | 3.085066 | 0.152126 |
| H | -3.425267 | -0.595526 | 0.893332 | H | 2.393509 | 1.364501 | 1.286839 |
| H | -0.386509 | -1.357594 | 2.176052 | H | 2.308224 | -1.455924 | -0.914809 |
| H | 2.093733 | -1.859654 | 0.484627 | H | -0.194599 | -2.894107 | -1.172369 |
| H | 3.517880 | 0.038635 | -0.051154 | H | -3.264155 | 0.131407 | 1.228483 |
| H | -2.265248 | 0.995993 | -1.530869 | H | 0.523050 | 1.177483 | -0.891463 |
| H | -2.147458 | -1.437921 | -1.483730 | H | 0.522676 | 0.082760 | 1.966483 |
| H | -1.394446 | -2.719476 | 0.474994 | H | 1.270345 | -2.039224 | 1.004620 |
| H | 0.322606 | -2.150923 | -1.153055 | H | -1.138500 | -1.775061 | 1.213486 |
| H | 0.791439 | 0.438371 | 0.416621 | H | -1.126371 | -0.476685 | -1.559327 |
| H | 2.209604 | 1.050021 | -1.520960 | H | -3.228524 | -1.677256 | -0.948214 |
| H | 1.877622 | -0.521868 | -2.277579 | H | -3.438378 | 0.080687 | -1.121384 |
| O | 4.332366 | 1.295616 | 0.910437 | O | -0.236118 | 4.466729 | -0.906334 |
| H | 4.295532 | 1.177730 | 1.870104 | H | 0.410625 | 4.643273 | -1.603894 |
| H | 5.265953 | 1.441036 | 0.702316 | H | -1.100525 | 4.486961 | -1.340472 |
| O | -1.269516 | 4.121833 | 0.346848 | O | 4.059352 | -1.215939 | -0.419097 |
| H | -2.122167 | 4.539269 | 0.524704 | H | 4.643812 | -0.832975 | -1.086968 |
| H | -1.442645 | 3.159459 | 0.358237 | H | 3.743204 | -0.472464 | 0.131647 |
| | α-allose-2w-8 | | | | β-allose-2w-8 | | |
| C | -2.025908 | -1.462312 | 0.116685 | C | -0.817746 | 0.223746 | 0.201708 |
| C | -0.835749 | -1.765630 | -0.824364 | C | -0.599387 | -1.168661 | -0.40811 |
| C | 0.294099 | -0.753527 | -0.591621 | C | 0.773823 | -1.724312 | -0.010805 |
| C | -0.259134 | 0.667775 | -0.769174 | C | 1.902316 | -0.715079 | -0.247456 |
| C | -1.479200 | 0.898704 | 0.140732 | C | 1.543869 | 0.661456 | 0.345535 |
| C | -2.188661 | 2.227172 | -0.139899 | C | 2.533062 | 1.762836 | -0.018882 |
| O | -1.742559 | -1.725327 | 1.469439 | O | -1.988290 | 0.786313 | -0.314706 |
| O | -0.376423 | -3.103010 | -0.685475 | O | -1.559030 | -2.116781 | 0.043145 |
| O | 0.780440 | -1.008429 | 0.731240 | O | 0.804456 | -2.046160 | 1.386487 |
| O | 0.785189 | 1.614649 | -0.514786 | O | 3.132044 | -1.204261 | 0.28549 |
| O | -2.462650 | -0.117523 | -0.101074 | O | 0.269024 | 1.077331 | -0.17174 |
| O | -1.304285 | 3.345434 | 0.051118 | O | 2.614693 | 1.987733 | -1.427807 |
| H | -0.824310 | -1.444335 | 1.648497 | H | -2.256540 | 1.560072 | 0.235518 |
| H | 0.138412 | -3.150475 | 0.135310 | H | -2.428589 | -1.917583 | -0.365425 |
| H | 1.625558 | -0.516765 | 0.886484 | H | 0.042336 | -2.615320 | 1.57141 |
| H | 0.357626 | 2.482942 | -0.383513 | H | 2.932753 | -1.601025 | 1.148571 |
| H | -1.284172 | 3.579618 | 0.989921 | H | 1.723673 | 2.203250 | -1.740428 |
| H | -2.889380 | -2.075195 | -0.148901 | H | -0.862780 | 0.161776 | 1.298985 |
| H | -1.199797 | -1.671028 | -1.850878 | H | -0.634445 | -1.059226 | -1.501504 |
| H | 1.097296 | -0.921476 | -1.321041 | H | 0.968585 | -2.632823 | -0.596366 |
| H | -0.589111 | 0.761482 | -1.815127 | H | 2.053281 | -0.597987 | -1.32446 |

| | | | | | | | |
|---|---|---|---|---|---|---|---|
| H | -1.158583 | 0.875360 | 1.191216 | H | 1.497076 | 0.586832 | 1.442519 |
| H | -3.074459 | 2.315992 | 0.496607 | H | 3.533433 | 1.475092 | 0.31126 |
| H | -2.507065 | 2.262238 | -1.185708 | H | 2.244016 | 2.685405 | 0.501377 |
| O | 2.922105 | 0.619686 | 1.005562 | O | -2.843692 | 2.922962 | 1.161499 |
| H | 2.414303 | 1.224374 | 0.431972 | H | -3.096547 | 2.750309 | 2.079391 |
| H | 3.764846 | 0.419985 | 0.545475 | H | -2.262929 | 3.696372 | 1.191261 |
| O | 5.362867 | -0.036760 | -0.195476 | O | -3.846658 | -1.099646 | -1.184325 |
| H | 6.042843 | 0.651291 | -0.198118 | H | -4.697034 | -1.142596 | -0.726811 |
| H | 5.335841 | -0.376863 | -1.100744 | H | -3.432301 | -0.252393 | -0.921584 |
| | α-allose-2w-9 | | | | β-allose-2w-9 | | |
| C | -1.075332 | -1.520020 | -0.022711 | C | -1.389144 | -0.287927 | -0.121829 |
| C | 0.158771 | -1.784804 | -0.916053 | C | -0.634136 | -1.139046 | -1.146881 |
| C | 1.250322 | -0.744645 | -0.634297 | C | 0.862957 | -1.168167 | -0.830056 |
| C | 0.669795 | 0.660976 | -0.839961 | C | 1.401332 | 0.263595 | -0.688857 |
| C | -0.591166 | 0.871256 | 0.017890 | C | 0.545308 | 1.081660 | 0.302340 |
| C | -1.316255 | 2.182239 | -0.308663 | C | 0.916272 | 2.559799 | 0.354314 |
| O | -0.848960 | -1.772187 | 1.337628 | O | -2.719453 | -0.216635 | -0.516001 |
| O | 0.643010 | -3.110085 | -0.761570 | O | -1.092580 | -2.490970 | -1.130975 |
| O | 1.679595 | -0.987342 | 0.711369 | O | 1.076646 | -1.885477 | 0.399374 |
| O | 1.678801 | 1.634326 | -0.546479 | O | 2.785248 | 0.306779 | -0.374818 |
| O | -1.539583 | -0.177320 | -0.258946 | O | -0.833794 | 1.032491 | -0.109039 |
| O | -0.459476 | 3.316663 | -0.096633 | O | 0.747413 | 3.214487 | -0.904588 |
| H | 0.052683 | -1.470021 | 1.561295 | H | -3.286479 | 0.009723 | 0.255658 |
| H | 1.121588 | -3.152921 | 0.081099 | H | -2.061336 | -2.475394 | -1.161687 |
| H | 2.498048 | -0.476398 | 0.909328 | H | 0.549074 | -2.698579 | 0.352653 |
| H | 1.227459 | 2.495808 | -0.449244 | H | 2.963898 | -0.144103 | 0.476924 |
| H | -0.491970 | 3.572453 | 0.836263 | H | -0.172389 | 3.082901 | -1.178364 |
| H | -1.911941 | -2.150317 | -0.329057 | H | -1.295051 | -0.719811 | 0.885634 |
| H | -0.165621 | -1.694881 | -1.956045 | H | -0.790065 | -0.689543 | -2.137197 |
| H | 2.089828 | -0.893217 | -1.325250 | H | 1.401280 | -1.677545 | -1.639659 |
| H | 0.382806 | 0.743526 | -1.898910 | H | 1.305783 | 0.737042 | -1.671372 |
| H | -0.318041 | 0.859284 | 1.081061 | H | 0.646892 | 0.664412 | 1.315717 |
| H | -2.227963 | 2.265707 | 0.290134 | H | 1.969611 | 2.661909 | 0.622545 |
| H | -1.592239 | 2.196655 | -1.366868 | H | 0.307782 | 3.048773 | 1.126426 |
| O | -4.303837 | 0.061306 | 0.503302 | O | -4.382884 | 0.372312 | 1.595445 |
| H | -3.353578 | -0.007194 | 0.288881 | H | -4.496287 | -0.318726 | 2.263005 |
| H | -4.384604 | -0.260336 | 1.410799 | H | -4.205961 | 1.185472 | 2.088948 |
| O | 3.812835 | 0.724709 | 0.959823 | O | 3.221721 | -1.210335 | 1.992166 |
| H | 3.991807 | 1.132864 | 1.817717 | H | 4.018297 | -1.757829 | 1.982112 |
| H | 3.205846 | 1.328414 | 0.483632 | H | 2.510876 | -1.749409 | 1.591955 |
| | α-allose-2w-10 | | | | β-allose-2w-10 | | |
| C | 1.125775 | -1.501431 | 0.618685 | C | -0.908646 | 0.374608 | 0.764940 |
| C | 1.813060 | -0.879641 | -0.617158 | C | -1.286068 | -0.541419 | -0.406929 |
| C | 1.088604 | 0.398658 | -1.053430 | C | -0.271141 | -1.683712 | -0.535940 |
| C | -0.400150 | 0.107710 | -1.272938 | C | 1.177394 | -1.184067 | -0.530241 |
| C | -1.015917 | -0.591327 | -0.045015 | C | 1.428532 | -0.222559 | 0.646115 |
| C | -2.441364 | -1.084534 | -0.313655 | C | 2.811363 | 0.427653 | 0.627344 |
| O | 1.292468 | -0.743719 | 1.794043 | O | -1.730110 | 1.514617 | 0.742660 |
| O | 3.210938 | -0.725063 | -0.450813 | O | -2.558048 | -1.147924 | -0.227932 |
| O | 1.230355 | 1.400937 | -0.019021 | O | -0.390476 | -2.592061 | 0.566656 |
| O | -1.031930 | 1.364520 | -1.521855 | O | 2.083602 | -2.284595 | -0.497865 |
| O | -0.248479 | -1.748991 | 0.310883 | O | 0.437283 | 0.829631 | 0.603526 |
| O | -3.289628 | -0.000018 | -0.707720 | O | 3.141082 | 1.075479 | -0.596294 |
| H | 1.197471 | 0.199432 | 1.571420 | H | -1.655023 | 1.980853 | 1.588450 |
| H | 3.423018 | 0.027436 | 0.139554 | H | -3.260195 | -0.478485 | -0.377698 |

| | | | |
|---|---|---|---|
| H | 0.555921 | 2.079471 | -0.196004 |
| H | -1.991088 | 1.219264 | -1.393276 |
| H | -3.720509 | 0.380017 | 0.089499 |
| H | 1.542730 | -2.488794 | 0.825193 |
| H | 1.690564 | -1.603948 | -1.429291 |
| H | 1.532676 | 0.781797 | -1.980392 |
| H | -0.497272 | -0.552682 | -2.147008 |
| H | -1.039625 | 0.117222 | 0.794273 |
| H | -2.831552 | -1.584426 | 0.579399 |
| H | -2.427801 | -1.808522 | -1.135322 |
| O | -4.498697 | 0.994241 | 1.570698 |
| H | -4.322993 | 1.922565 | 1.779569 |
| H | -5.460712 | 0.897553 | 1.607294 |
| O | 3.881828 | 1.700054 | 0.843763 |
| H | 3.985891 | 1.807500 | 1.798873 |
| H | 2.968401 | 1.973817 | 0.637473 |

β-mannose-2w-1

| | | | |
|---|---|---|---|
| C | 0.240869 | -1.191181 | -0.519307 |
| H | 0.306503 | -1.296989 | -1.609363 |
| O | 1.274419 | -1.956002 | 0.030765 |
| H | 1.071601 | -2.093286 | 0.972171 |
| C | -0.510086 | 1.096220 | -0.651642 |
| H | -0.561154 | 1.043276 | -1.751152 |
| O | 0.473844 | 0.170332 | -0.159568 |
| C | -1.127009 | -1.677858 | -0.018443 |
| H | -1.325421 | -2.665079 | -0.456760 |
| O | -1.025052 | -1.786175 | 1.400636 |
| H | -1.905509 | -1.991679 | 1.748445 |
| C | -2.231711 | -0.708481 | -0.438902 |
| H | -2.343003 | -0.761225 | -1.532822 |
| O | -3.435706 | -1.145840 | 0.189784 |
| H | -4.105106 | -0.459733 | 0.045288 |
| C | -1.886614 | 0.731400 | -0.065787 |
| H | -1.853329 | 0.831036 | 1.023663 |
| O | -2.927720 | 1.551723 | -0.609892 |
| H | -3.060877 | 2.322489 | -0.042638 |
| C | -0.012408 | 2.485335 | -0.266291 |
| H | 0.954141 | 2.659468 | -0.756191 |
| H | -0.716168 | 3.241580 | -0.622407 |
| O | 0.097894 | 2.660520 | 1.146194 |
| H | 0.709847 | 1.987894 | 1.479502 |
| O | 4.057667 | -1.501512 | -0.645163 |
| H | 4.341225 | -0.628531 | -0.312685 |
| H | 3.119694 | -1.591689 | -0.398980 |
| O | 4.938153 | 1.047544 | 0.313465 |
| H | 5.612308 | 1.018414 | 1.006393 |
| H | 5.288989 | 1.646734 | -0.359996 |

β-mannose-2w-2

| | | | |
|---|---|---|---|
| C | 1.823405 | 1.569318 | -0.585333 |
| H | 2.070119 | 1.615593 | -1.653801 |
| O | 1.972149 | 2.854907 | -0.071146 |
| H | 2.023985 | 2.778977 | 0.896408 |
| C | 0.127127 | -0.109524 | -0.943995 |
| H | 0.385388 | -0.177320 | -2.013169 |
| O | 0.447741 | 1.195972 | -0.439251 |

| | | | |
|---|---|---|---|
| H | -1.316421 | -2.872770 | 0.617370 |
| H | 1.729374 | -2.929521 | 0.135134 |
| H | 2.611201 | 1.894809 | -0.673565 |
| H | -0.999942 | -0.155395 | 1.722222 |
| H | -1.259363 | 0.063930 | -1.324160 |
| H | -0.466182 | -2.220678 | -1.473578 |
| H | 1.372540 | -0.644556 | -1.461067 |
| H | 1.326675 | -0.766576 | 1.596573 |
| H | 3.553456 | -0.360293 | 0.778807 |
| H | 2.877275 | 1.125624 | 1.472919 |
| O | 1.268007 | 3.184594 | -0.707235 |
| H | 1.363537 | 3.976493 | -0.161240 |
| H | 0.708990 | 2.566121 | -0.198665 |
| O | -4.212943 | 1.061041 | -0.571191 |
| H | -5.050630 | 1.132834 | -0.093876 |
| H | -3.551082 | 1.538328 | -0.037970 |

β-xylose-2w-1

| | | | |
|---|---|---|---|
| C | 0.332953 | -0.201150 | -0.284919 |
| H | 0.640926 | -0.250553 | 0.772926 |
| O | 1.422056 | 0.016332 | -1.114462 |
| H | 2.222370 | -0.432305 | -0.745850 |
| C | -1.409109 | -1.753394 | 0.186163 |
| H | -1.108980 | -1.812989 | 1.243248 |
| H | -1.771785 | -2.733943 | -0.127708 |
| O | -0.284770 | -1.447003 | -0.641203 |
| C | -0.671970 | 0.939107 | -0.493049 |
| H | -0.923041 | 0.974232 | -1.562866 |
| O | -0.133049 | 2.190519 | -0.069366 |
| H | 0.667116 | 2.358206 | -0.588836 |
| C | -1.938548 | 0.689510 | 0.321572 |
| H | -1.683717 | 0.736774 | 1.391215 |
| O | -2.962818 | 1.643867 | 0.032365 |
| H | -2.634887 | 2.522167 | 0.273478 |
| C | -2.504263 | -0.696485 | 0.017439 |
| H | -2.859597 | -0.709944 | -1.022902 |
| O | -3.572018 | -1.032356 | 0.902165 |
| H | -4.230938 | -0.324331 | 0.850489 |
| O | 3.607542 | -1.173944 | -0.039722 |
| H | 4.041853 | -1.839444 | -0.589643 |
| H | 4.313093 | -0.556079 | 0.258447 |
| O | 5.469824 | 0.646653 | 0.881714 |
| H | 6.109650 | 0.324772 | 1.532091 |
| H | 5.989413 | 1.125363 | 0.220740 |

β-xylose-2w-2

| | | | |
|---|---|---|---|
| C | 2.014773 | -1.216520 | 0.193343 |
| H | 1.878161 | -1.387753 | 1.272924 |
| O | 3.117435 | -1.922487 | -0.291695 |
| H | 3.121644 | -2.813948 | 0.085225 |
| C | -0.337435 | -0.991919 | -0.069577 |
| H | -0.504351 | -1.151278 | 1.005831 |
| H | -1.165558 | -1.438967 | -0.621750 |
| O | 0.849752 | -1.661678 | -0.503181 |
| C | 2.253505 | 0.272756 | -0.085390 |
| H | 2.447877 | 0.389869 | -1.160611 |
| O | 3.342685 | 0.782561 | 0.679278 |

| | | | |
|---|---|---|---|
| C | 2.746589 | 0.567858 | 0.125539 |
| H | 3.784403 | 0.800323 | -0.149397 |
| O | 2.559398 | 0.760453 | 1.527082 |
| H | 3.053257 | 0.067914 | 1.990660 |
| C | 2.423165 | -0.864266 | -0.300602 |
| H | 2.702085 | -0.982509 | -1.359027 |
| O | 3.215193 | -1.733943 | 0.507043 |
| H | 2.911665 | -2.639882 | 0.343149 |
| C | 0.932354 | -1.166559 | -0.165742 |
| H | 0.646381 | -1.134909 | 0.890226 |
| O | 0.733300 | -2.481737 | -0.698424 |
| H | 0.021649 | -2.922452 | -0.215854 |
| C | -1.384146 | -0.250984 | -0.815898 |
| H | -1.867238 | 0.515267 | -1.433864 |
| H | -1.700830 | -1.233515 | -1.172277 |
| O | -1.823708 | -0.146449 | 0.543758 |
| H | -1.523276 | 0.707990 | 0.889010 |
| O | -4.519271 | -0.838634 | 1.202657 |
| H | -3.615473 | -0.567558 | 0.952203 |
| H | -5.123986 | -0.275385 | 0.683522 |
| O | -6.329200 | 0.796296 | -0.306014 |
| H | -6.948904 | 0.317229 | -0.873573 |
| H | -6.874993 | 1.400263 | 0.216327 |

β-mannose-2w-3

| | | | |
|---|---|---|---|
| C | 1.775104 | -1.257701 | -0.926094 |
| H | 1.414799 | -1.295852 | -1.962569 |
| O | 2.895742 | -2.082727 | -0.847786 |
| H | 3.052624 | -2.270625 | 0.092807 |
| C | 1.136296 | 1.047293 | -0.704070 |
| H | 0.681402 | 1.028345 | -1.708095 |
| O | 2.190608 | 0.077496 | -0.625375 |
| C | 0.669495 | -1.716670 | 0.032647 |
| H | 0.266892 | -2.670001 | -0.335884 |
| O | 1.286070 | -1.913342 | 1.305998 |
| H | 0.578412 | -2.086989 | 1.945140 |
| C | -0.477320 | -0.702888 | 0.118803 |
| H | -1.020396 | -0.718669 | -0.838650 |
| O | -1.322609 | -1.148016 | 1.172280 |
| H | -2.194230 | -0.694003 | 1.099677 |
| C | 0.050434 | 0.719643 | 0.340171 |
| H | 0.474659 | 0.796098 | 1.345757 |
| O | -1.057492 | 1.629621 | 0.205028 |
| H | -0.887576 | 2.419581 | 0.735841 |
| C | 1.800670 | 2.407454 | -0.513424 |
| H | 2.533016 | 2.552419 | -1.317995 |
| H | 1.054632 | 3.202267 | -0.587348 |
| O | 2.420980 | 2.544992 | 0.765260 |
| H | 3.077518 | 1.838149 | 0.851224 |
| O | -3.551406 | 0.414183 | 0.826675 |
| H | -4.180335 | 0.207961 | 0.103236 |
| H | -2.915415 | 1.064937 | 0.476943 |
| O | -5.351305 | -0.201026 | -1.234280 |
| H | -5.528007 | -1.143159 | -1.365661 |
| H | -6.220770 | 0.217312 | -1.165018 |

β-mannose-2w-4

| | | | |
|---|---|---|---|
| H | 4.155540 | 0.352371 | 0.377252 |
| C | 1.015557 | 1.080625 | 0.302220 |
| H | 0.887424 | 1.022587 | 1.393656 |
| O | 1.117160 | 2.447955 | -0.095471 |
| H | 1.854420 | 2.851498 | 0.384923 |
| C | -0.230134 | 0.505917 | -0.366646 |
| H | -0.152946 | 0.656086 | -1.452031 |
| O | -1.413904 | 1.142940 | 0.125664 |
| H | -1.277154 | 2.101531 | 0.069256 |
| O | -3.991131 | 0.552149 | -1.038512 |
| H | -3.122503 | 0.720260 | -0.628462 |
| H | -4.504916 | 0.041691 | -0.384038 |
| O | -5.622696 | -0.931031 | 0.785299 |
| H | -5.625446 | -0.644547 | 1.709103 |
| H | -5.484191 | -1.887841 | 0.813967 |

β-xylose-2w-3

| | | | |
|---|---|---|---|
| C | 1.426151 | -0.106873 | -0.035068 |
| H | 1.548112 | -0.263442 | 1.049538 |
| O | 2.650864 | -0.124873 | -0.686592 |
| H | 3.241634 | -0.793147 | -0.271362 |
| C | -0.673954 | -1.221357 | 0.073424 |
| H | -0.565284 | -1.380166 | 1.156729 |
| H | -1.193807 | -2.080498 | -0.354169 |
| O | 0.604637 | -1.159098 | -0.560943 |
| C | 0.756920 | 1.246822 | -0.306542 |
| H | 0.694627 | 1.378936 | -1.396091 |
| O | 1.489995 | 2.317801 | 0.283804 |
| H | 2.375624 | 2.322922 | -0.108220 |
| C | -0.647861 | 1.273753 | 0.291854 |
| H | -0.564317 | 1.226278 | 1.387994 |
| O | -1.376006 | 2.445259 | -0.080814 |
| H | -0.925800 | 3.216410 | 0.292856 |
| C | -1.453374 | 0.070213 | -0.189263 |
| H | -1.635748 | 0.171318 | -1.267607 |
| O | -2.704950 | -0.013361 | 0.504016 |
| H | -3.125306 | 0.859681 | 0.458069 |
| O | -4.628028 | -1.993387 | -0.178002 |
| H | -3.951070 | -1.332945 | 0.072369 |
| H | -4.682052 | -2.603016 | 0.569280 |
| O | 4.298296 | -1.962962 | 0.536065 |
| H | 4.508865 | -2.755185 | 0.021917 |
| H | 5.148763 | -1.617524 | 0.842008 |

β-xylose-2w-4

| | | | |
|---|---|---|---|
| C | 1.668939 | 0.081759 | -0.410751 |
| H | 1.668472 | -0.019643 | -1.506197 |
| O | 2.939311 | 0.389965 | 0.070935 |
| H | 3.384863 | 0.996894 | -0.537256 |
| C | -0.569050 | 0.923971 | -0.492879 |
| H | -0.596467 | 0.820438 | -1.586352 |
| H | -1.140288 | 1.808679 | -0.208611 |
| O | 0.774743 | 1.143947 | -0.041995 |
| C | 1.213334 | -1.222309 | 0.255442 |
| H | 1.290854 | -1.092290 | 1.343739 |
| O | 2.000912 | -2.327526 | -0.175695 |
| H | 2.903194 | -2.208713 | 0.154226 |

| | | | |
|---|---|---|---|
| C | 2.607154 | 0.337022 | 0.101943 |
| H | 3.136063 | 0.576143 | -0.830099 |
| O | 3.509653 | 0.524935 | 1.146893 |
| H | 3.149327 | 0.073889 | 1.928579 |
| C | 0.525646 | 1.199480 | -0.744955 |
| H | 0.988603 | 1.347534 | -1.734625 |
| O | 1.532598 | 1.267428 | 0.278979 |
| C | 2.094994 | -1.112387 | 0.055062 |
| H | 2.925066 | -1.759054 | -0.261501 |
| O | 1.694591 | -1.454665 | 1.381540 |
| H | 1.207444 | -2.291172 | 1.332814 |
| C | 0.938897 | -1.250438 | -0.937266 |
| H | 1.334016 | -1.116762 | -1.955977 |
| O | 0.412301 | -2.570306 | -0.795145 |
| H | -0.442516 | -2.582014 | -1.253844 |
| C | -0.134116 | -0.192006 | -0.692763 |
| H | -0.554950 | -0.338529 | 0.308786 |
| O | -1.146063 | -0.364769 | -1.678196 |
| H | -2.003684 | -0.092961 | -1.273947 |
| C | -0.426248 | 2.366155 | -0.514630 |
| H | 0.151321 | 3.297777 | -0.502753 |
| H | -1.145610 | 2.416059 | -1.334560 |
| O | -1.200609 | 2.249053 | 0.687363 |
| H | -0.597948 | 2.181043 | 1.442305 |
| O | -3.288969 | 0.596567 | -0.258073 |
| H | -3.768045 | -0.026759 | 0.327501 |
| H | -2.744258 | 1.168983 | 0.316284 |
| O | -4.667054 | -1.201081 | 1.394948 |
| H | -5.631129 | -1.188301 | 1.316624 |
| H | -4.413234 | -2.130121 | 1.303593 |

β-mannose-2w-5

| | | | |
|---|---|---|---|
| C | 1.694609 | 0.532200 | -0.330947 |
| H | 2.053442 | 0.554140 | -1.367329 |
| O | 2.367162 | 1.545950 | 0.359434 |
| H | 2.273893 | 1.367635 | 1.311424 |
| C | -0.538844 | -0.106704 | -0.976370 |
| H | -0.241706 | -0.195452 | -2.033295 |
| O | 0.301971 | 0.846178 | -0.304439 |
| C | 1.970799 | -0.838283 | 0.303769 |
| H | 3.021098 | -1.100936 | 0.119915 |
| O | 1.743081 | -0.687234 | 1.703937 |
| H | 1.840143 | -1.556720 | 2.119521 |
| C | 1.068631 | -1.908454 | -0.311399 |
| H | 1.371762 | -2.057745 | -1.359389 |
| O | 1.277996 | -3.110344 | 0.426992 |
| H | 0.617875 | -3.754409 | 0.128700 |
| C | -0.398187 | -1.481764 | -0.296783 |
| H | -0.749238 | -1.413404 | 0.737615 |
| O | -1.123781 | -2.493423 | -1.005237 |
| H | -2.004221 | -2.591613 | -0.619585 |
| C | -1.946598 | 0.473681 | -0.928373 |
| H | -1.961189 | 1.424634 | -1.473475 |
| H | -2.651247 | -0.210699 | -1.405207 |
| O | -2.403365 | 0.662548 | 0.417096 |
| H | -1.773559 | 1.245327 | 0.868013 |
| C | -0.235527 | -1.529193 | -0.122685 |
| H | -0.278073 | -1.741550 | -1.201434 |
| O | -0.759393 | -2.638334 | 0.605938 |
| H | -0.261026 | -3.430329 | 0.357821 |
| C | -1.133716 | -0.331567 | 0.174135 |
| H | -1.177317 | -0.178375 | 1.260750 |
| O | -2.453309 | -0.548442 | -0.336634 |
| H | -2.744823 | -1.423906 | -0.036852 |
| O | 1.616282 | 3.860853 | 0.287457 |
| H | 1.366214 | 2.920855 | 0.203575 |
| H | 1.860277 | 3.979193 | 1.214633 |
| O | -4.538555 | 1.360390 | -0.002960 |
| H | -4.766602 | 1.398134 | 0.934938 |
| H | -3.813095 | 0.707827 | -0.068916 |

β-xylose-2w-5

| | | | |
|---|---|---|---|
| C | -0.743713 | 0.313558 | 0.645351 |
| H | -0.594942 | 0.251278 | 1.733223 |
| O | -2.099108 | 0.491406 | 0.337214 |
| H | -2.496737 | 1.119382 | 0.958050 |
| C | 1.373498 | 1.408364 | 0.403747 |
| H | 1.558692 | 1.347461 | 1.485220 |
| H | 1.788641 | 2.344382 | 0.027807 |
| O | -0.036384 | 1.453613 | 0.139703 |
| C | -0.231653 | -0.957101 | -0.050503 |
| H | -0.465351 | -0.867939 | -1.121151 |
| O | -0.798401 | -2.139468 | 0.493684 |
| H | -1.713269 | -2.238587 | 0.153214 |
| C | 1.285017 | -1.070290 | 0.127367 |
| H | 1.496993 | -1.239267 | 1.194560 |
| O | 1.817421 | -2.148469 | -0.640470 |
| H | 1.354626 | -2.956115 | -0.371419 |
| C | 1.995995 | 0.207439 | -0.309236 |
| H | 1.873782 | 0.328874 | -1.394827 |
| O | 3.381135 | 0.180168 | 0.028232 |
| H | 3.765809 | -0.617428 | -0.364132 |
| O | -1.238262 | 3.979537 | -0.476733 |
| H | -0.866574 | 3.096112 | -0.292033 |
| H | -1.541490 | 3.943393 | -1.393196 |
| O | -3.431918 | -1.926077 | -0.373781 |
| H | -3.700162 | -2.083512 | -1.289169 |
| H | -3.290744 | -0.965533 | -0.289307 |

β-xylose-2w-6

| | | | |
|---|---|---|---|
| C | 2.119082 | -0.838697 | 0.185375 |
| H | 2.099813 | -1.024915 | 1.270783 |
| O | 3.400356 | -1.029382 | -0.335522 |
| H | 3.791308 | -1.826484 | 0.049929 |
| C | -0.114881 | -1.614572 | 0.019368 |
| H | -0.155290 | -1.807120 | 1.101839 |
| H | -0.702589 | -2.378466 | -0.491615 |
| O | 1.223917 | -1.740393 | -0.463738 |
| C | 1.698803 | 0.599574 | -0.127073 |
| H | 1.785590 | 0.743175 | -1.212975 |
| O | 2.497430 | 1.555280 | 0.567692 |
| H | 3.397100 | 1.520645 | 0.212367 |
| C | 0.252122 | 0.846539 | 0.302865 |

| | | | |
|---|---|---|---|
| O | 2.114974 | 4.275524 | -0.446724 |
| H | 1.184895 | 4.532511 | -0.402746 |
| H | 2.139238 | 3.340464 | -0.165525 |
| O | -5.104182 | 1.301250 | 0.917056 |
| H | -4.168169 | 1.067339 | 0.749129 |
| H | -5.446326 | 0.601272 | 1.487935 |

β-mannose-2w-6

| | | | |
|---|---|---|---|
| C | 0.176436 | -1.327087 | 1.357389 |
| H | 0.881689 | -1.433129 | 2.192273 |
| O | -0.926194 | -2.128121 | 1.653483 |
| H | -1.440766 | -2.243523 | 0.838120 |
| C | 0.772727 | 0.990223 | 1.041610 |
| H | 1.490206 | 0.961005 | 1.877455 |
| O | -0.269945 | 0.025438 | 1.271922 |
| C | 0.850031 | -1.740332 | 0.043870 |
| H | 1.241507 | -2.759864 | 0.145741 |
| O | -0.175136 | -1.716612 | -0.959672 |
| H | 0.220631 | -1.975308 | -1.805116 |
| C | 1.994527 | -0.782015 | -0.277673 |
| H | 2.767105 | -0.900528 | 0.497660 |
| O | 2.524479 | -1.155021 | -1.548816 |
| H | 3.156424 | -0.469539 | -1.813904 |
| C | 1.516676 | 0.667458 | -0.269833 |
| H | 0.841182 | 0.821144 | -1.115913 |
| O | 2.682832 | 1.485540 | -0.425211 |
| H | 2.437125 | 2.308834 | -0.868340 |
| C | 0.100445 | 2.363931 | 1.033569 |
| H | -0.539286 | 2.426444 | 1.925089 |
| H | 0.868643 | 3.138312 | 1.119003 |
| O | -0.633512 | 2.648449 | -0.150774 |
| H | -1.213324 | 1.893699 | -0.399588 |
| O | -2.052239 | 0.552789 | -1.343144 |
| H | -2.960834 | 0.318469 | -1.062976 |
| H | -1.497991 | -0.229627 | -1.167595 |
| O | -4.714243 | 0.028114 | -0.580578 |
| H | -4.883201 | -0.690930 | 0.044212 |
| H | -5.322511 | -0.123133 | -1.317395 |

β-mannose-2w-7

| | | | |
|---|---|---|---|
| C | -1.680364 | 0.537863 | -0.406573 |
| H | -1.940127 | 0.586898 | -1.471532 |
| O | -2.882378 | 0.469612 | 0.307409 |
| H | -2.690976 | 0.704391 | 1.231946 |
| C | 0.304945 | -0.744563 | -0.833359 |
| H | 0.152387 | -0.618857 | -1.917715 |
| O | -0.960464 | -0.668643 | -0.152342 |
| C | -0.872540 | 1.780039 | 0.000768 |
| H | -1.393566 | 2.669932 | -0.377694 |
| O | -0.849056 | 1.801312 | 1.427208 |
| H | -0.215861 | 2.481641 | 1.701934 |
| C | 0.538424 | 1.727935 | -0.589802 |
| H | 0.467423 | 1.859108 | -1.680472 |
| O | 1.273371 | 2.811295 | -0.021685 |
| H | 2.208724 | 2.657677 | -0.227846 |
| C | 1.210506 | 0.385765 | -0.306663 |
| H | 1.319488 | 0.267178 | 0.777345 |
| H | 0.194713 | 0.803517 | 1.400207 |
| O | -0.171503 | 2.144414 | -0.141691 |
| H | 0.469449 | 2.793459 | 0.186661 |
| C | -0.684077 | -0.219815 | -0.275454 |
| H | -0.754604 | -0.077950 | -1.362555 |
| O | -1.984123 | -0.172771 | 0.313002 |
| H | -2.434212 | 0.665012 | 0.055924 |
| O | -3.890021 | -2.243425 | 0.108528 |
| H | -3.206314 | -1.546139 | 0.190843 |
| H | -3.973594 | -2.627895 | 0.990636 |
| O | -2.914852 | 2.324918 | -0.418556 |
| H | -3.442031 | 2.813738 | 0.227713 |
| H | -1.985269 | 2.602119 | -0.287967 |

β-xylose-2w-7

| | | | |
|---|---|---|---|
| C | -0.986122 | 1.728551 | 0.256768 |
| H | -1.065150 | 1.680056 | 1.354419 |
| O | -1.909502 | 2.633841 | -0.272693 |
| H | -1.940792 | 3.427951 | 0.279871 |
| C | 1.336736 | 1.279043 | 0.411604 |
| H | 1.279545 | 1.215612 | 1.508017 |
| H | 2.296710 | 1.718419 | 0.135926 |
| O | 0.326631 | 2.154514 | -0.099685 |
| C | -1.288758 | 0.354592 | -0.357635 |
| H | -1.282166 | 0.455971 | -1.451053 |
| O | -2.570724 | -0.105698 | 0.086623 |
| H | -3.239124 | 0.533471 | -0.202308 |
| C | -0.227181 | -0.663540 | 0.071345 |
| H | -0.325482 | -0.831417 | 1.154896 |
| O | -0.318834 | -1.903014 | -0.621314 |
| H | -1.092807 | -2.404494 | -0.285360 |
| C | 1.170422 | -0.107637 | -0.209303 |
| H | 1.306357 | -0.033275 | -1.296940 |
| O | 2.166799 | -0.976825 | 0.339808 |
| H | 1.929004 | -1.881091 | 0.077701 |
| O | 4.920898 | -0.557307 | -0.209875 |
| H | 3.973605 | -0.681054 | 0.002015 |
| H | 5.353779 | -0.384397 | 0.636115 |
| O | -2.729982 | -2.873496 | 0.395297 |
| H | -3.300513 | -3.396517 | -0.183723 |
| H | -3.005825 | -1.941412 | 0.288461 |

β-xylose-2w-8

| | | | |
|---|---|---|---|
| C | 1.130386 | -1.037009 | 0.431304 |
| H | 1.087081 | -1.088709 | 1.530291 |
| O | 2.305291 | -1.638542 | -0.046416 |
| H | 2.426990 | -2.494992 | 0.389092 |
| C | -1.239052 | -1.249782 | 0.304025 |
| H | -1.325805 | -1.283221 | 1.399847 |
| H | -1.995363 | -1.907303 | -0.127422 |
| O | 0.029113 | -1.757702 | -0.120256 |
| C | 1.093360 | 0.425878 | -0.040017 |
| H | 1.216703 | 0.429354 | -1.132504 |
| O | 2.086231 | 1.227894 | 0.583003 |
| H | 2.951579 | 1.044427 | 0.158874 |
| C | -0.260509 | 1.044037 | 0.318769 |
| H | -0.331534 | 1.106401 | 1.415743 |

| | | | |
|---|---|---|---|
| O | 2.490844 | 0.405067 | -0.929290 |
| H | 3.097128 | -0.142360 | -0.384038 |
| C | 0.844790 | -2.151906 | -0.615270 |
| H | 0.093299 | -2.876060 | -0.949550 |
| H | 1.748660 | -2.291901 | -1.211578 |
| O | 1.219484 | -2.421579 | 0.743709 |
| H | 0.450653 | -2.286944 | 1.316932 |
| O | -4.624530 | -1.765244 | -0.060992 |
| H | -4.127115 | -2.578217 | 0.096172 |
| H | -3.984489 | -1.039765 | 0.074663 |
| O | 3.784562 | -1.354074 | 0.794544 |
| H | 2.951553 | -1.806311 | 1.044939 |
| H | 4.173581 | -1.019182 | 1.613414 |

β-mannose-2w-8

| | | | |
|---|---|---|---|
| C | 0.822873 | -0.974815 | -0.738213 |
| H | 1.029944 | -0.915074 | -1.813762 |
| O | 1.521933 | -2.075260 | -0.233471 |
| H | 1.087082 | -2.315503 | 0.606097 |
| C | 0.710680 | 1.424364 | -0.475728 |
| H | 0.834323 | 1.586438 | -1.558564 |
| O | 1.336065 | 0.189739 | -0.088104 |
| C | -0.684730 | -1.138302 | -0.474229 |
| H | -1.056468 | -1.956416 | -1.105308 |
| O | -0.807524 | -1.479804 | 0.901290 |
| H | -1.697104 | -1.865842 | 1.057021 |
| C | -1.407981 | 0.156669 | -0.854947 |
| H | -1.289055 | 0.299280 | -1.940174 |
| O | -2.802564 | 0.030359 | -0.559634 |
| H | -3.202541 | 0.906628 | -0.674048 |
| C | -0.793950 | 1.362986 | -0.150537 |
| H | -0.933198 | 1.268330 | 0.930594 |
| O | -1.484746 | 2.517157 | -0.645379 |
| H | -1.534222 | 3.187222 | 0.048997 |
| C | 1.465512 | 2.527551 | 0.258475 |
| H | 2.512522 | 2.514196 | -0.070572 |
| H | 1.039417 | 3.500152 | 0.000796 |
| O | 1.380023 | 2.404610 | 1.678020 |
| H | 1.736433 | 1.537151 | 1.919897 |
| O | 4.372611 | -2.035851 | -0.156607 |
| H | 4.662732 | -1.244581 | 0.315356 |
| H | 3.396249 | -1.992575 | -0.158741 |
| O | -3.491561 | -2.103426 | 1.094721 |
| H | -3.882232 | -2.876850 | 0.666255 |
| H | -3.572190 | -1.363459 | 0.460550 |

β-mannose-2w-9

| | | | |
|---|---|---|---|
| C | 1.746647 | 1.073920 | -0.792537 |
| H | 2.030462 | 0.904817 | -1.838403 |
| O | 2.181216 | 2.346970 | -0.437148 |
| H | 2.250749 | 2.374304 | 0.532553 |
| C | -0.298429 | -0.193618 | -1.057473 |
| H | 0.012894 | -0.466595 | -2.078356 |
| O | 0.311314 | 1.063223 | -0.699487 |
| C | 2.363179 | -0.003186 | 0.111898 |
| H | 3.435047 | -0.073000 | -0.117321 |
| O | 2.180590 | 0.446746 | 1.453828 |
| O | -0.409439 | 2.347118 | -0.242511 |
| H | 0.322203 | 2.893157 | 0.081933 |
| C | -1.411749 | 0.186645 | -0.192918 |
| H | -1.408411 | 0.196224 | -1.291057 |
| O | -2.669042 | 0.678000 | 0.289305 |
| H | -2.704633 | 1.629633 | 0.105047 |
| O | -5.111723 | -0.504315 | -0.560033 |
| H | -4.268234 | -0.118337 | -0.249172 |
| H | -5.526141 | -0.883794 | 0.225656 |
| O | 4.337615 | 0.231640 | -0.726609 |
| H | 5.200564 | 0.225723 | -0.290880 |
| H | 3.936033 | -0.639911 | -0.554290 |

β-xylose-2w-9

| | | | |
|---|---|---|---|
| C | 0.863837 | -0.978436 | -0.479873 |
| H | 0.692187 | -0.978828 | -1.566577 |
| O | 1.423401 | -2.183757 | -0.056043 |
| H | 2.063386 | -2.497753 | -0.710874 |
| C | 1.276211 | 1.367723 | -0.567026 |
| H | 1.106802 | 1.380330 | -1.652701 |
| H | 2.055839 | 2.090939 | -0.323966 |
| O | 1.766771 | 0.083664 | -0.153683 |
| C | -0.458497 | -0.787054 | 0.273028 |
| H | -0.256105 | -0.860804 | 1.349729 |
| O | -1.389881 | -1.800460 | -0.123801 |
| H | -1.015214 | -2.664232 | 0.105038 |
| C | -1.061082 | 0.586652 | -0.044048 |
| H | -1.358338 | 0.600143 | -1.103929 |
| O | -2.176645 | 0.909720 | 0.777733 |
| H | -2.952001 | 0.380017 | 0.493789 |
| C | -0.017828 | 1.685819 | 0.180446 |
| H | 0.197847 | 1.741205 | 1.257300 |
| O | -0.494998 | 2.943963 | -0.286653 |
| H | -1.372407 | 3.078101 | 0.103418 |
| O | 4.543791 | -0.244761 | 0.457697 |
| H | 3.586910 | -0.178503 | 0.276380 |
| H | 4.611125 | -0.491909 | 1.389118 |
| O | -4.050683 | -0.955438 | -0.135547 |
| H | -4.678236 | -1.324100 | 0.500535 |
| H | -3.271682 | -1.546281 | -0.130058 |

β-xylose-2w-10

| | | | |
|---|---|---|---|
| C | 1.483027 | 0.769177 | 0.414837 |
| H | 1.476973 | 0.874471 | 1.510011 |
| O | 2.567865 | 1.434559 | -0.153581 |
| H | 3.350221 | 1.327375 | 0.406099 |
| C | 0.497432 | -1.393285 | 0.629595 |
| H | 0.487889 | -1.306541 | 1.725220 |
| H | 0.691834 | -2.432845 | 0.363192 |
| O | 1.574721 | -0.622120 | 0.079914 |
| C | 0.203244 | 1.341650 | -0.199871 |
| H | 0.289837 | 1.253372 | -1.291649 |
| O | -0.001358 | 2.701534 | 0.176230 |
| H | 0.685317 | 3.243239 | -0.238465 |
| C | -1.024610 | 0.565535 | 0.277122 |
| H | -1.165002 | 0.747237 | 1.352401 |
| O | -2.188968 | 1.014256 | -0.433209 |

| | | | |
|---|---|---|---|
| H | 2.494430 | -0.250768 | 2.048117 |
| C | 1.708551 | -1.363899 | -0.128763 |
| H | 1.995202 | -1.714008 | -1.132370 |
| O | 2.222002 | -2.257001 | 0.858061 |
| H | 1.709431 | -3.077887 | 0.801985 |
| C | 0.184776 | -1.274253 | -0.074233 |
| H | -0.132290 | -1.005405 | 0.938189 |
| O | -0.309460 | -2.571894 | -0.423935 |
| H | -1.155838 | -2.723387 | 0.017580 |
| C | -1.813039 | -0.003694 | -1.086303 |
| H | -2.049804 | 0.834485 | -1.753000 |
| H | -2.266787 | -0.905679 | -1.505011 |
| O | -2.395663 | 0.192782 | 0.203374 |
| H | -2.174881 | 1.098372 | 0.516978 |
| O | -1.421661 | 2.727432 | 0.758486 |
| H | -1.195550 | 2.963103 | 1.668452 |
| H | -0.597215 | 2.404907 | 0.344997 |
| O | -5.079271 | -0.484519 | 0.552435 |
| H | -4.132632 | -0.253865 | 0.429892 |
| H | -5.085858 | -1.230923 | 1.165257 |
| | β-mannose-2w-10 | | |
| C | 1.333123 | -0.854974 | -0.482118 |
| H | 1.366167 | -0.908056 | -1.577569 |
| O | 2.530493 | -1.403886 | -0.008340 |
| H | 2.405791 | -1.617507 | 0.932611 |
| C | 0.119530 | 1.213635 | -0.511639 |
| H | 0.040560 | 1.167579 | -1.609743 |
| O | 1.291779 | 0.510381 | -0.073561 |
| C | 0.125268 | -1.631176 | 0.054362 |
| H | 0.112498 | -2.623357 | -0.416106 |
| O | 0.323762 | -1.763558 | 1.461843 |
| H | -0.473833 | -2.170055 | 1.832830 |
| C | -1.197267 | -0.922041 | -0.261790 |
| H | -1.377397 | -0.999679 | -1.344977 |
| O | -2.202088 | -1.627648 | 0.457593 |
| H | -3.083621 | -1.390167 | 0.098366 |
| C | -1.135603 | 0.565687 | 0.106619 |
| H | -1.104149 | 0.667796 | 1.195433 |
| O | -2.321306 | 1.199518 | -0.411191 |
| H | -2.511647 | 1.993060 | 0.107781 |
| C | 0.333772 | 2.671689 | -0.115473 |
| H | 1.230623 | 3.042105 | -0.628165 |
| H | -0.515679 | 3.276808 | -0.441533 |
| O | 0.446759 | 2.850098 | 1.295999 |
| H | 1.196450 | 2.318642 | 1.601748 |
| O | 5.048800 | -0.303234 | -0.772552 |
| H | 5.082908 | 0.623363 | -0.501918 |
| H | 4.171330 | -0.624595 | -0.487683 |
| O | -4.468268 | -0.568770 | -0.766248 |
| H | -3.940223 | 0.253070 | -0.715517 |
| H | -5.272171 | -0.409126 | -0.253670 |
| H | -2.265687 | 1.972202 | -0.307122 |
| C | -0.852143 | -0.942467 | 0.058774 |
| H | -0.870200 | -1.143260 | -1.021995 |
| O | -1.845619 | -1.708387 | 0.726062 |
| H | -2.708801 | -1.577537 | 0.279232 |
| O | 3.992094 | -1.981546 | -0.613376 |
| H | 3.181414 | -1.478368 | -0.405953 |
| H | 4.085028 | -1.932814 | -1.573653 |
| O | -4.213421 | -0.881534 | -0.512805 |
| H | -4.457473 | -1.190077 | -1.395685 |
| H | -3.705560 | -0.054200 | -0.638266 |
